# Ancient balancing selection at *tan* underlies female colour dimorphism in *Drosophila erecta*

**DOI:** 10.1038/ncomms10400

**Published:** 2016-01-18

**Authors:** Amir Yassin, Héloïse Bastide, Henry Chung, Michel Veuille, Jean R. David, John E. Pool

**Affiliations:** 1 Laboratory of Genetics, University of Wisconsin-Madison, Madison, Wisconsin 53706, USA; 2 Howard Hughes Medical Institute and Laboratory of Molecular Biology, University of Wisconsin-Madison, Madison, Wisconsin 53706, USA; 3 Institut Systématique Evolution Biodiversité ISYEB—UMR 7205—CNRS—MNHN—UPMC—EPHE, Ecole Pratique des Hautes Etudes, Paris-Sciences-Lettres, Paris 75005, France; 4 Laboratoire Evolution, Génomes, Comportement, Ecologie (EGCE), CNRS, IRD, University of Paris-Sud, Université Paris-Saclay, Gif-sur-Yvette 91198, France

## Abstract

Dimorphic traits are ubiquitous in nature, but the evolutionary factors leading to dimorphism are largely unclear. We investigate a potential case of sexual mimicry in *Drosophila erecta*, in which females show contrasting resemblance to males. We map the genetic basis of this sex-limited colour dimorphism to a region containing the gene *tan*. We find a striking signal of ancient balancing selection at the ‘male-specific enhancer' of *tan*, with exceptionally high sequence divergence between light and dark alleles, suggesting that this dimorphism has been adaptively maintained for millions of years. Using transgenic reporter assays, we confirm that these enhancer alleles encode expression differences that are predicted to generate this pigmentation dimorphism. These results are compatible with the theoretical prediction that divergent phenotypes maintained by selection can evolve simple genetic architectures.

## Abstract (web summary)

Sexual dimorphism is common in nature. Here, the authors combine population genetics and functional experiments to show that a region containing the gene *tan* contributes to sex-limited colour dimorphism in *Drosophila erecta* and that this dimorphism has likely been adaptively maintained for millions of years.

## Graphical abstract

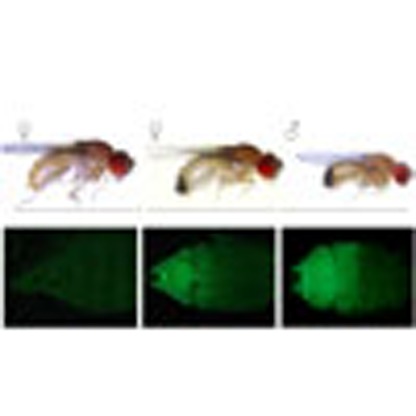

In developing his multifactorial theory of inheritance, R.A. Fisher^1^ showed that genetic variation at just a few loci, together with environmental variance, could lead to continuous variation in a phenotypic trait. And yet, a surprising number of traits in natural populations shows variation that is dichotomous, rather than continuous (for example, sexes, social castes, colour morphs, left–right asymmetry), and these dimorphisms can have either a complex or simple genetic architecture^2^,^3^,^4^. The role of selection in maintaining dimorphic traits has had a long history in evolutionary biology^5^. However, the mechanisms leading to the origin of distinct morphs are largely unclear^6^,^7^,^8^. Theory predicts that long-term frequency-dependent disruptive selection (FDDS) on a continuous polygenic trait, in which disruptive selection eliminates intermediate phenotypes, whereas frequency-dependent selection maintains extreme phenotypes, can lead to the evolution of dimorphic traits with simple genetic architectures^7^, but empirical support for this model is lacking^9^,^10^.

*Drosophila* pigmentation offers unique opportunities to dissect the evolution of genetic architectures, in part due to its well-understood biochemical pathway, as well as the presence of both complex and monogenic systems in related species^11^. Notably, a number of *Drosophila* species have evolved an intriguing form of colour dimorphism, in which some females resemble males while others are visually distinct. A prime example of this female-limited colour dimorphism (FLCD) is found in *D. erecta*, a member of the melanogaster species subgroup. *D. melanogaster* reflects the ancestral condition of this clade, wherein males consistently have dark posterior abdomen, whereas females have a continuously varying abdominal pigmentation (Fig. 1a) that is always lighter than males^12^. A similar continuous distribution is found in *D. orena*, the closest relative of *D. erecta*, but with a lighter mean (Fig. 1a). In *D. erecta*, however, light and dark distinct female morphs coexist in natural populations without intermediate forms, alongside uniformly dark males (Fig. 1a,b). All other cases of FLCD belong to the distant montium species group^13^.

The FLCD found in *D. erecta* and other *Drosophila* species is reminiscent of a classic system of sexual mimicry described in some damselfly species, in which male-mimicking females are believed to benefit from avoiding harassment by males when population densities are high^14^,^15^. Concordantly, Payant^16^ found that evidence for frequency-dependent mating behaviour: when light females of *D. erecta* were common, dark females mated less. Further, it seems plausible that the cost of mating for females may be higher in *D. erecta* than in its non-FLCD relatives, in light of the species' larger and serrated male phallus and the corresponding protective plates found in females^17^. Hence, one hypothesis for FLCD is that male-mimicking females may avoid costly rematings, as has been suggested for multiple damselfly species^14^,^15^.

In this paper, we use introgression mapping to localize the genetic basis of *D. erecta* FLCD to a single locus on the X chromosome containing the melanin synthesis enzyme gene *tan*. Based on the genetic divergence estimates between morphs, we detect a strong signal of ancient balancing selection on the previously identified ‘male-specific enhancer' of this gene. We confirm using transgenic reporters that alleles at this enhancer encode expression differences in females that are predicted to replicate their pigmentation differences. Our results support a hypothesis in which long-term balancing selection on female colour morphs yielded a simple genetic architecture involving the modification of a sexually dimorphic enhancer.

## Results

### Introgression mapping of FLCD in *D. erecta*

Whereas multiple genes affect continuous pigmentation in related species such as *D. melanogaster*^18^,^19^,^20^,^21^, we found FLCD in *D. erecta* to be caused by a single X-linked locus, in agreement with previous studies and observations^16^. When we crossed the dominant allele conferring dark female pigmentation from an inbred dark-female strain into an inbred light-female strain, all F1 flies were dark indistinguishable from their dark parents, indicating complete dominance of the dark allele. Phenotypes of F2 flies did not deviate from the 1:1 ratio expected from a single X-linked locus (680 and 645 dark and light females, respectively; *χ*^2^, *P*=0.34). To identify the causative locus, we introgressed the dark allele into a light background through a series of backcrosses and performed genomic sequence analysis on seventh offspring generation of this introgression heterozygous for the dark allele (line BC7). After 12 generations, we generated an introgression line homozygous for the dark allele from four dark homozygous pairs (line NN) and sequenced its genome as well. The dark pigmentation of these flies did not differ from the parental dark line (pigmentation scores: 18.45±0.23 and 18.85±0.24; Mann–Whitney *U*-test *P*=0.20; Supplementary Fig. 1). We then estimated the proportion of alleles from the dark parent in both BC7 and NN lines (Supplementary Fig. 2). Since this ancestry proportion at the FLCD locus is expected to be 50% and 100% in heterozygous BC7 and homozygous NN females, respectively, the average score of the FLCD locus should be near 75%. Only ten neighbouring windows comprising a 1-Mb interval on chromosome X conformed to this expectation. The 100-kb window with the highest ancestry metric contained the known pigmentation gene *tan* (Fig. 2a), an enzyme that helps govern the transition between light and dark pigment precursors^22^.

### Sequence divergence between morphs at the mapped region

Examining parental strain genome sequences around the *tan* locus, we identified a ∼0.5-kb interval with extreme sequence divergence between the female-light and female-dark alleles (Fig. 2b). This window corresponds to the ‘male-specific enhancer' of *tan* (*t*_MSE), which contributed to the loss of male abdominal pigmentation in another melanogaster subgroup species, *D. santomea*^23^, and has been localized to a ∼150-bp region^24^. At the *t*_MSE, the divergence between *D. erecta* genomes was several times higher than normal (Fig. 2b). Comparing the *t*_MSE region to the rest of the X chromosome, the Hudson–Kreitman–Aguadé (HKA)-like test^25^ confirmed a substantial excess of within-species polymorphism relative to expectations based on divergence between *D. erecta* and its relative *D. orena* (*P*<7 × 10^−19^; Supplementary Fig. 3 and Supplementary Table 1).

### Population genetic analysis of the *t*_MSE-containing locus

The exceptional differentiation between light and dark *D. erecta t*_MSE sequences suggests an ancient divergence time between these alleles, and is most consistent with a history of long-term balancing selection. Consistent with this explanation, when we sequenced a 1-kb region containing the *t*_MSE from 36 wild-caught *D. erecta* males, alleles clustered into two haplogroups similar to our light and dark strain *t*_MSE sequences (Fig. 3a and Supplementary Note 1). These haplogroups were present at intermediate frequencies in both Cameroon and Gabon (Fig. 3b), and no differentiation was observed between sequences from these populations (*F*_ST_=−0.02). Since most variation was found between haplogroups rather than within them, *F*_ST_ between haplogroups showed very high levels of genetic differentiation (Fig. 3c). In contrast, flanking loci 5–10 kb away had low differentiation between *t*_MSE haplogroups, and HKA analysis confirmed a powerful excess of polymorphism at the *t*_MSE-containing locus relative to the flanking loci (*χ*^2^, *P*=4.99 × 10^−4^ and 1.50 × 10^−5^ for up- and downstream loci, respectively; Supplementary Table 2 and Supplementary Notes 2 and 3). Notably among the 135 polymorphic sites of the *t*_MSE-containing locus, 76 nucleotide differences were fixed between the light and dark *D. erecta* alleles, one-third of which (that is, 26) fell within the *t*_MSE sequence (Supplementary Fig. 4).

### Phylogenetic analysis of the *t*_MSE-containing locus

Sequence divergence between haplogroups at the *t*_MSE-containing locus was not only much greater than within-haplogroup variation, but also significantly larger than *t*_MSE divergence between the dark haplogroup and the *D. orena* genome sequence (Fig. 3a). Such incomplete lineage sorting was restricted to an interval of <1 kb centred on the *t*_MSE (Supplementary Fig. 5), suggesting that neither allele introgressed into *D. erecta* in the very recent past. Further evidence against introgression comes from the elevated divergence between *D. orena* and both *D. erecta* haplotypes, and from laboratory experiments showing these species' inability to hybridize^26^. These observations suggest that light and dark *t*_MSE alleles have been present for a time equal to or longer than the divergence between *D. erecta* and *D. orena*.

**Figure 1:**
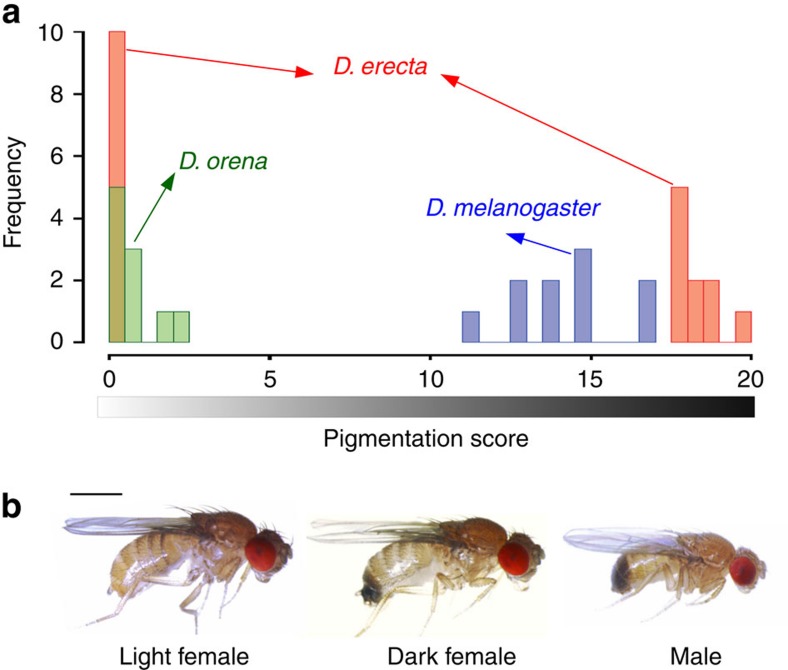
Female-limited colour dimorphism in *D. erecta*. (**a**) Histogram of pigmentation score on the last two abdominal segments in ten females in three melanogaster subgroup species grown under similar conditions. Only *D. erecta* (red) shows a bimodal distribution, whereas both *D. orena* (green) and *D. melanogaster* (blue) show a continuous, unimodal distribution. (**b**) Photomicrographs of male and female *D. erecta* showing the dark (male-like) and light female morphs. Scale bar, 1 mm.

**Figure 2:**
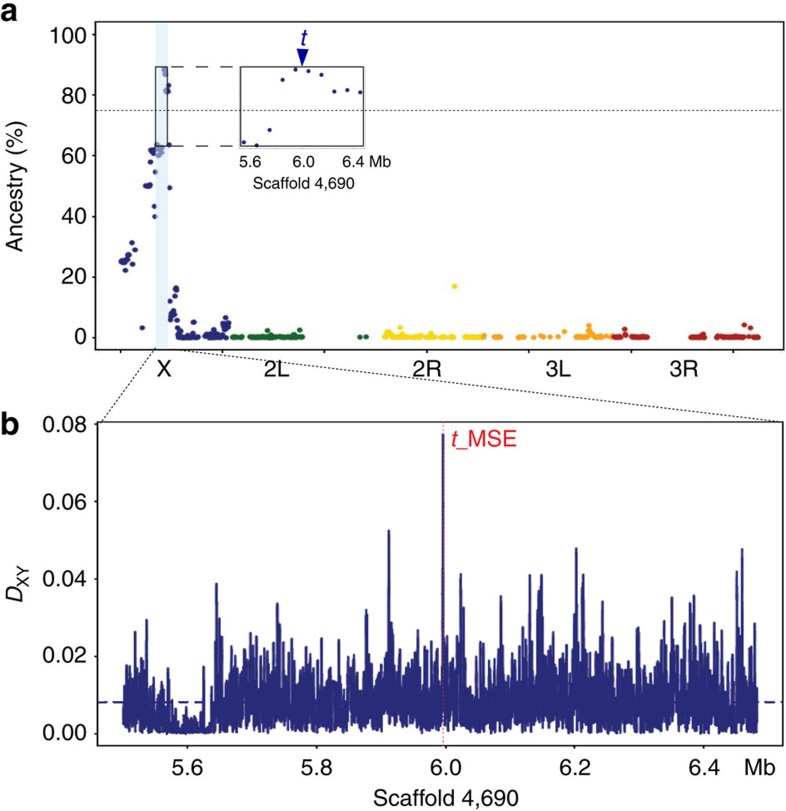
The FLCD locus maps to the *tan* male-specific enhancer (*t*_MSE) region in *D. erecta*. (**a**) Genetic mapping signal indicated by ancestry proportion of the dark parental line averaged between heterozygous dark 7th backcross generation and homozygous dark introgression line. Each dot corresponds to a 100-kb window, and each chromosome arm is given by a different colour. Windows with high identity-by-descent percentage were excluded. Dashed horizontal line indicates 75% ancestry score expected for the dark allele. The inset shows a 1-Mb interval harbouring windows with the highest ancestry score centred on *tan* (*t*). (**b**) Genetic divergence (*D*_xy_) between the two parental dark and light lines at the 1-Mb interval with the highest ancestry score. Dotted red line indicates the *t*_MSE region as delimited by Camino *et al*.^24^.

**Figure 3:**
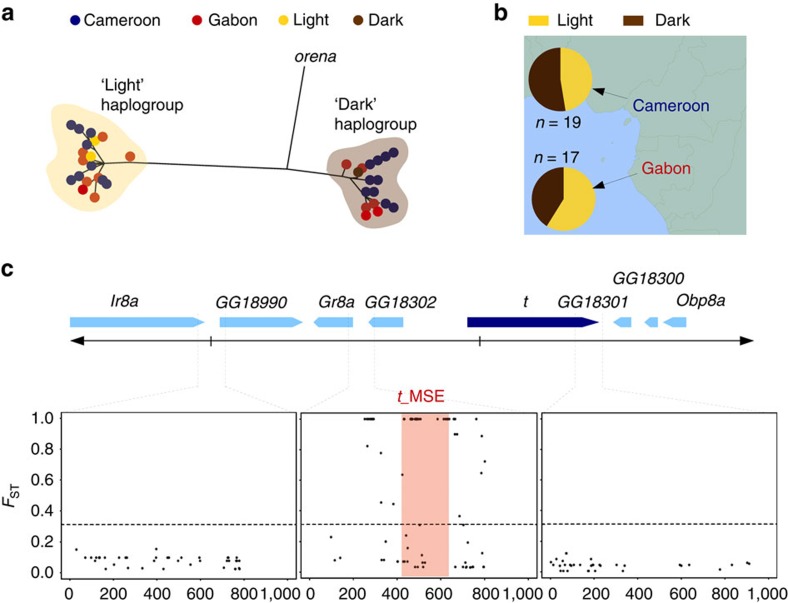
Ongoing balancing selection on the FLCD locus in *D. erecta*. (**a**) Phylogenetic clustering of *D. erecta t*_MSE haplotypes sequenced from the dark parental strain (brown), light parental strain (yellow), Cameroon (blue) and Gabon (red) into dark and light haplogroups. (**b**) Intermediate frequencies of haplotypes belonging to the light (yellow) and dark (brown) haplogroups in Cameroon and Gabon. (**c**) Genetic differentiation (*F*_ST_) at each SNP between the light and dark haplogroups, for the *t*_MSE-containing locus (centre) and two neighbouring loci situated 5- and 10-kb up- and downstream, respectively. The *t*_MSE region as delimited by Camino *et al*.^24^ is highlighted in red.

**Figure 4:**
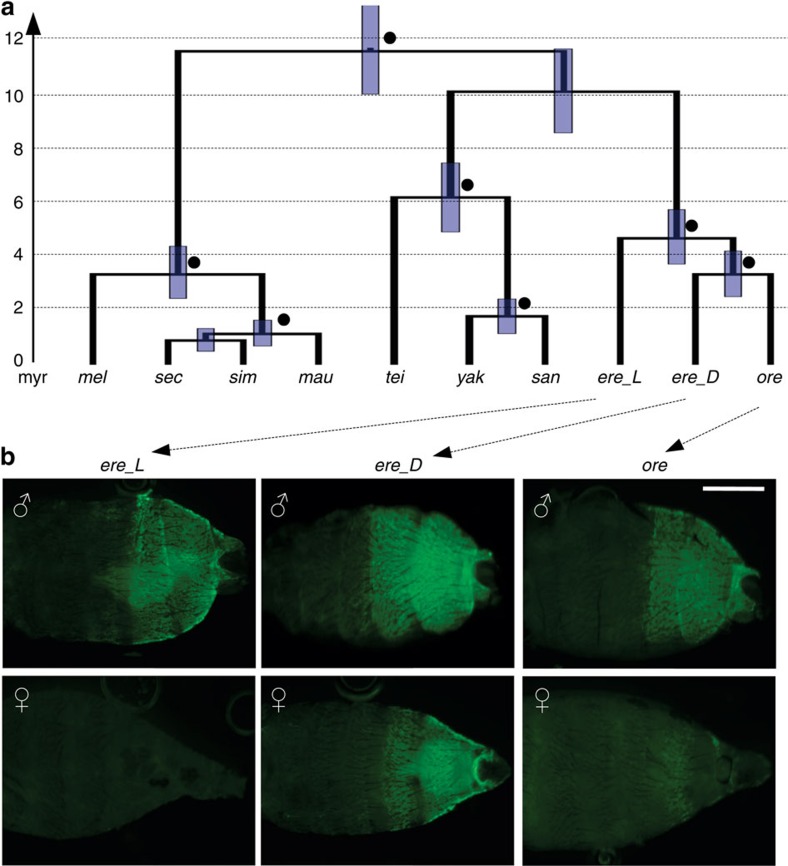
Ancient origin and function of the FLCD locus in *D. erecta*. (**a**) Dated Bayesian phylogeny of a 1-kb region containing *t*_MSE between the nine species of the melanogaster subgroup: *mel*, *melanogaster*; *sim*, *simulans*; *mau*, *mauritiana*; *sec*, *sechellia*; *tei*, *teissieri*; *yak*, *yakuba*; *san*, *santomea*; *ere_L*, ‘light' *erecta*; *ere_D*, ‘dark' *erecta*; *ore*, *orena*. Solid black spheres indicate node posterior probability support >95%. Blue bars indicate 95% confidence intervals of the age of each node. (**b**) EGFP expression at eclosion in males (above) and females (below) driven by the *t*_MSE-containing locus from dark and light *D. erecta* and *D. orena*. Scale bar, 0.5 mm.

The closer relationship of the *D. orena t*_MSE to the dark allele of *D. erecta* was also reflected by a phylogenetic tree constructed with all melanogaster subgroup species using the *t*_MSE-containing locus (Bayesian posterior probability=99.8%; Fig. 4a and Supplementary Note 4). Calibrating our sequence divergence by the estimated age of this clade^27^, the coalescence time between *D. orena* and the dark haplotype of *D. erecta* was estimated at ∼3.4±0.9 million years, whereas the coalescence between dark and light *D. erecta t*_MSE alleles was estimated to be ∼4.7±1.0 million years ago (with both estimates falling within the range between 3.1 and 5.5 million years ago typically estimated for the divergence between *D. erecta* and *D. orena*^27^). The relationship between *D. orena* and the dark *D. erecta* allele is curious because *D. orena* females have light abdomens (Fig. 1a). However, the activity of the *D. orena t*_MSE might be altered by a 44-bp insertion within the enhancer region as defined by Jeong *et al*.^23^ and Camino *et al*.^24^, along with 23 single-nucleotide differences with the dark haplogroup across the *t*_MSE-containing locus (Supplementary Note 4).

### Functional analysis of alleles at the *t*_MSE-containing locus

To investigate the link between *t*_MSE alleles and *tan* expression, we generated reporter constructs in which the *t*_MSE region of dark *D. erecta*, light *D. erecta* or *D. orena* was placed upstream of green fluorescent protein (GFP). Each construct was inserted into the same 51D site of the *D. melanogaster* genome using the ΦC31 integrase system^28^. In transgenic flies, the level of GFP fluorescence in a relevant tissue then permits an assessment of the regulatory element's activity. We found that GFP in the abdominal segments driven by the different *t*_MSE alleles closely mirrored the pigmentation of the studied strains (Fig. 4b). Posterior abdominal segments showed high GFP expression in all males, and in females carrying the dark *D. erecta* construct. Females with the light *D. erecta* construct or the *D. orena* construct showed little or no expression throughout the abdomen, consistent with the light female pigmentation of the source strains. A consistent match between *t*_MSE reporter activity and *tan* protein staining has been reported across a wide range of *Drosophila* species^24^. However, additional experiments will be needed to confirm that an expression change at *tan* is responsible for FLCD, and to identify the causative nucleotide(s).

## Discussion

The above results motivate the hypothesis that the dark-female *t*_MSE allele of *D. erecta* evolved by extending the activity of an otherwise male-specific enhancer into females as well. Increasing *tan* expression in the female abdomen is predicted to increase production of melanic pigments^22^, leading to the male-like pigmentation observed in females of dark *D. erecta* strains. This dark allele may therefore represent a loss of sexual dimorphism at the molecular and phenotypic levels, even as it creates a novel dimorphism among females. Curiously, this same *cis*-regulatory element of *tan* has also underlain the loss of sexual dimorphism in the related species *D. santomea* leading to the evolution of female-like light males^23^ as well as in other sexually monomorphic *Drosophila* species^24^.

In the abdomen of *D. melanogaster*, *tan* is upregulated in males by the Hox genes *Abd-A* and *Abd-B*^24^ and suppressed in females by the transcription factors *bab1* and *bab2* whose expressions are sexually dimorphic^29^. Although the exact binding sites of Bab1 and Bab2 are still unknown, it is possible that the dark haplotype in *D. erecta* involves the loss of such sites. In another *Drosophila* species with monogenic FLCD (*D. kikkawai*), the FLCD locus is still unknown but mapping indicates that it is different from *tan* or *bab*^30^, highlighting the complexity of this trait. Future investigations using recent advances in *Drosophila* molecular biology techniques as well as other species with FLCD will help the precise dissection of the genetic basis of this sexual colour dimorphism.

Although quite rare, female-limited colour variation has been described in at least two other groups of non-drosophilid insects^31^. Aside from the damselfly case referenced above, certain butterflies have both mimetic and non-mimetic female morphs, which vary in frequency geographically due to spatially varying selective pressures^32^,^33^,^34^. These morphs are associated with ∼400 kb chromosomal rearrangements in *Heliconius* butterflies^3^. In *Papilio polytes*, complex pigmentation variation correlates with ∼130 kb inversion-associated alleles of *doublesex*^4^,^35^, a component of the sexual differentiation pathway. In our study, the genetic tools and knowledge of *Drosophila* allowed us to localize the genetic basis of a sex-limited dimorphic trait to a regulatory element of <1 kb, indicating the strong potential of this system for further insights regarding the mechanisms of sex-specific evolution and the origin of dimorphic traits.

Our study provides a rare example of the genetic basis of an ancient balanced polymorphism with clear morphological consequences. Most known examples include genes involved in immunity interactions or mate recognition such as major histocompatibility complexes in vertebrates^36^, blood groups in primates^37^, self-incompatibility in plants^38^ and mating-types in fungi^39^. Balancing selection can involve different mechanisms such as heterozygous advantage, spatially or temporally variable selective pressures, or frequency-dependent selection. In the case of *D. erecta* FLCD, it is not clear why ecological factors would maintain discrete pigmentation morphs at similar frequencies in different populations, and preserve them for millions of years. Payant^16^ conducted extensive experiments on factors maintaining *D. erecta* FLCD in the laboratory. She observed a mating preference for light females when the frequency of the light allele was between 0.5 and 0.7. Although further study is called for, frequency-dependent sexual selection thus represents a plausible explanation for FLCD, especially given the morphological similarity of our case with FLCD in damselflies, a prime model for frequency-dependent sexual selection^14^,^15^,^40^.

The monogenic nature of FLCD and the frequency-dependent mating results cited above suggest that this trait could be consistent with theoretical predictions for FDDS, which can lead to the evolution of dimorphic traits with simple genetic architectures^7^. Disruptive selection would explain the lack of intermediate phenotypes found in nature, whereas evidence from this work is consistent with a role for balancing selection in maintaining *D. erecta* FLCD, potentially due to frequency-dependent sexual selection. FDDS should increase the effect of one or a few loci relative to all others. In agreement with this model, other *Drosophila* species have more modest and continuous pigmentation variation because of several genes including *tan*^18^,^19^,^20^,^41^, whereas in the *D. erecta* lineage *tan*'s role increased to shape a discrete colour dimorphism. These results reflect important steps towards understanding the evolutionary and genetic mechanisms that give rise to dimorphic traits and sex-specific variation in nature.

## Methods

### Laboratory strains and pigmentation phenotyping

We generated several isofemale lines from a mass culture of *D. erecta* collected from Gabon in 2006 and where both colour morphs segregate with no intermediate phenotypes. We then selected two lines that were homozygous either for the dark or light allele. We scored female pigmentation on the last two abdominal tergites from 0 (no pigmentation) to 10 (completely pigmented)^12^ for 10 females from the two parental lines as well as from a mass culture of a French population of *D. melanogaster* collected from Prunay in 2011 and a laboratory line of *D. orena* (strain no. 14021-0245.01 at Drosophila Stock Center, UCSD). Scores were summed over the two tergites and the R software package (http://www.r-project.org) was used to generate histograms illustrating the phenotypic distribution of each line. All lines were raised on standard *Drosophila* medium at 21 °C.

### Generation of introgression lines

The light *D. erecta* line was submitted to inbreeding, then a dark allele of a single male was introgressed into the light line for 12 successive backcrosses. For each backcross, 20 virgin, heterozygous dark females were crossed with 20 males from the parental light strain. After 12 generations, several male and virgin female pairs were established and their F_2_ progeny was checked for the presence of recessive light homozygotes. Four dark homozygous pairs were thus identified and pooled to generate the introgression line (NN) homozygous for the dark allele.

### Preparation of genome libraries

We sequenced the genomes of pooled samples (30 flies) for the parental dark and light lines, dark heterozygous flies from the 7th backcross generation (BC7), dark homozygous flies from the introgression line (NN) and the laboratory line of *D. orena*. Genomic DNA was obtained using chloroform extraction and ethanol precipitation. DNA was then fragmented using Bioruptor sonicator (Diagenode), and paired-end libraries with ∼300 bp inserts were prepared using NEBNext DNA Library Prep Reagent Set for Illumina (New England Biolabs, no. E6000L). Concentration and quality of libraries were assessed using an Agilent 2100 Bioanalyzer (Agilent Technologies, Inc.). They were then sequenced at UW-Madison Biotechnology Center on the Illumina HiSeq 2000 platform with 100 bp read lengths.

### Alignment of genomic reads

For each genome, reads were mapped to the *D. erecta* reference genome r.1.3 obtained from Flybase (http:// www.flybase.org) using default parameters in BWA ver. 0.6.2-r126 (ref. 42), and the BAM files were remapped using Stampy ver. 1.0.21 (ref. 43). Reads were then filtered for a mapping quality of 20 and for proper pairs with samtools ver. 0.1.18 (ref. 44). BAM files were cleaned by removing unmapped reads and sorted by coordinate, and PCR duplicates were marked using Picard ver. 1.109 (http://picard.sourceforge.net). Alignment around indels was then improved using GATK ver. 3.2 (refs 45, 46). The average coverage per genome was about × 17 and × 30 for the parental light and dark lines, respectively, × 44 and × 80 for BC7 and NN introgression lines, respectively, and × 21 for *D. orena*.

### Genome mapping

The PoPoolation2 ver. 1.201 software package^47^ was used to generate a synchronized mpileup file for the five genomes aligned to the *D. erecta* reference. The FLCD locus was identified by estimating the dark strain ancestry proportion in BC7 or NN individuals. For each diallelic single-nucleotide polymorphism (SNP) with a minimum count of ten in all lines, this quantity was calculated as the proportion of sequence reads carrying the allele that is fixed in the dark parental line and absent in the light parental line. Dark strain ancestry proportion was averaged across SNPs within 100 kb windows. As a quality filter, windows with fewer than 1 fixed difference per 1,000 sites between the parental strains were excluded as potentially identical-by-descent between these strains.

Genetic distances (*D*_xy_) were then compared between the two parental lines of *D. erecta* at ten 100-kb windows that conformed to the expected average ancestry score of 75% and centred on *tan*, using a perl script (available upon request from the first author). For each diallelic SNP, with a minimum sequencing coverage of 2, *D*_xy_ was estimated from the formula:


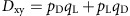


where *p* is the frequency of the allele similar to the allele in the reference *D. erecta* genome, and *q* is the frequency of the alternative allele in each line. Average *D*_xy_ at each SNP±250 SNPs (that is, sliding windows of 501 SNPs) was then estimated.

### HKA-like test

To test for a chromosome-wide signal for balancing selection, we estimated the number of polymorphic SNPs (that is, SNPs with *D*_xy_>0 between the light and dark parental lines) and divergent SNPs (that is, SNPs with *D*_xy_=1 between both lines and *D. orena*) along the X chromosome, that is, scaffolds 4,644 and 4,690 (ref. 48). For each SNP, the observed numbers of polymorphic and divergent SNPs at the SNP±50 SNPs (that is, sliding windows of 101 SNPs) were then compared with their expected numbers estimated from the chromosome-wide pattern using the HKA-like test^25^. Significance levels for deviation from expectations for each SNP-window were estimated using *χ*^2^ test at 1 degree of freedom as implemented in R.

### Single fly DNA extraction

DNA was extracted from 19 and 4 ethanol-preserved male specimens of *D. erecta* collected from Mt. Bafut (Cameroon) and Mboumi, la Lopé and Libreville (Gabon), respectively. The Gabonese population was completed by sequencing 8 males from isofemale lines collected in 2000 and 5 males from the mass culture collected in 2006. Each fly was ground in 49 μl of squishing buffer and 1 μl proteinase K and incubated for 20–30 min at 37 °C followed by 2 min at 95 °C.

### PCR amplification

We amplified the *tan* regulatory region (scaffold_4690:5994939..5995995 of the reference genome rel. 1.3) using ereF1 (5′-ACTGCTCAGCGTCTCCAGAT-3′) and ereR1 (5′-GGCTACGGATCCAGTGGTTA-3′) primers. These primers amplified the entire ∼1-kb interval harbouring the regulatory region. Given the low DNA quality of some of the specimens, we amplified the *t*_MSE region for those specimens using primers ereF2 (5′-TCCAATGCTAAATGAACCGG-3′) and ereR1. We also sequenced two additional loci, locus A (5-kb upstream the regulatory region, scaffold_4690:5989460..5990513) using primers ereFA (5′-AAGAAGCAGAACGCTCCTGA-3′) and ereRA (5′-CCTCACTTCCAGGTGATGCT-3′), and locus B (10-kb downstream the regulatory region, scaffold_4690:6003350..6004399) using primers ereFB (5′-ACAACGTCAAGGAGGAGCAC-3′) and ereRB (5′-TGCGCATACATGGTGAAATC-3′). PCR amplification was performed using Phusion High-Fidelity DNA Polymerase (New England Biolabs, no. M0530L) following the manufacture's protocol. PCR products were directly sequenced on an ABI 3730xl DNA Analyzers using a BigDye (Applied Biosystems) reaction at UW-Madison Biotechnology Center.

### Population genetic analyses

Nucleotide sequences were viewed, manually edited and aligned using MEGA. MEGA was also used to infer the phylogenetic relationships between the haplotypes using neighbour-joining. This tree was used to define the light and dark haplogroups. For each locus, DNA polymorphism was analysed using DnaSP ver. 5.10.1 (ref. 49). We estimated genetic differentiation (*F*_ST_) within and between the two populations and the two haplogroups, as well as genetic divergence (*D*_xy_) between the two haplogroups and between each haplogroup and *D. orena* genome. The HKA test^50^ was conducted in R after counting the number of polymorphic and divergent SNPs at each locus.

### Phylogenetic analysis

Sequences of the 1-kb-long *t*_MSE-containing locus in the nine species of the *melanogaster* subgroup were obtained from GenBank or our sequenced genomes, and aligned using the Muscle algorithm^51^ as implemented in the MEGA ver. 6.06 software package^52^. MEGA was also used to estimate the best substitution model, which was HKY with invariable sites. The BEAST v. 1.8 software package^53^ was used to infer the phylogenetic relationships between the sequences. A run of 10,000,000 generations under the coalescence model was conducted and sampled every 1,000 generations. A burn-in period of 2,500 generations was used. Divergence times were estimated by assuming the most recent common ancestor of the *melanogaster* subgroup to have lived nearly 11 million years ago^27^.

### Functional validation of the *tan* regulatory region

A ∼850-bp region including the *t*_MSE sequence (scaffold_4690:5995162..5996017 of the *D. erecta* reference genome rel. 1.3) was PCR amplified using forward (5′-TTCCGggcgcgccCCATGGAAGCCGAGCACCTGGTAGA-3′) and reverse primers (5′-TTGCCcctgcaggCTACAACGTRGGTCATGTNCAGGG-3′) with *Asc*I and *Sbf*I restriction sites given in small letters^54^. PCR products were cloned into the S3aG vector^55^, and inserted into the 51D site of *D. melanogaster* using ΦC31 integrase-mediated site-specific transgenesis (Best Gene Inc.)^28^. Presence or absence of GFP expression of transgenic males and females were scored at eclosion under an Olympus SZX16 Stereo Microscope equipped with an Olympus DP71 microscope digital camera.

## Additional information

**Accession codes:** Sequences generated in this study have been deposited in the NCBI SRA under accession code SRP057255 and GenBank under accession codes KR811377 to KR811457.

**How to cite this article:** Yassin, A. *et al*. Ancient balancing selection at *tan* underlies female colour dimorphism in *Drosophila erecta*. *Nat. Commun.* 7:10400 doi: 10.1038/ncomms10400 (2016).

## Supplementary Material

Supplementary Information Supplementary Figures 1-5, Supplementary Tables 1-2 and Supplementary Notes 1-4
