# Supplementary material for: Ancient balancing selection at tan underlies female colour dimorphism in Drosophila erecta
Source: Nat Commun. 2016 Jan 18;7:10400. doi: 10.1038/ncomms10400 (PMC4735637; doi:10.1038/ncomms10400)
Supplement: Supplementary Information — Supplementary Figures 1-5, Supplementary Tables 1-2 and Supplementary Notes 1-4 [file ncomms10400-s1.pdf]

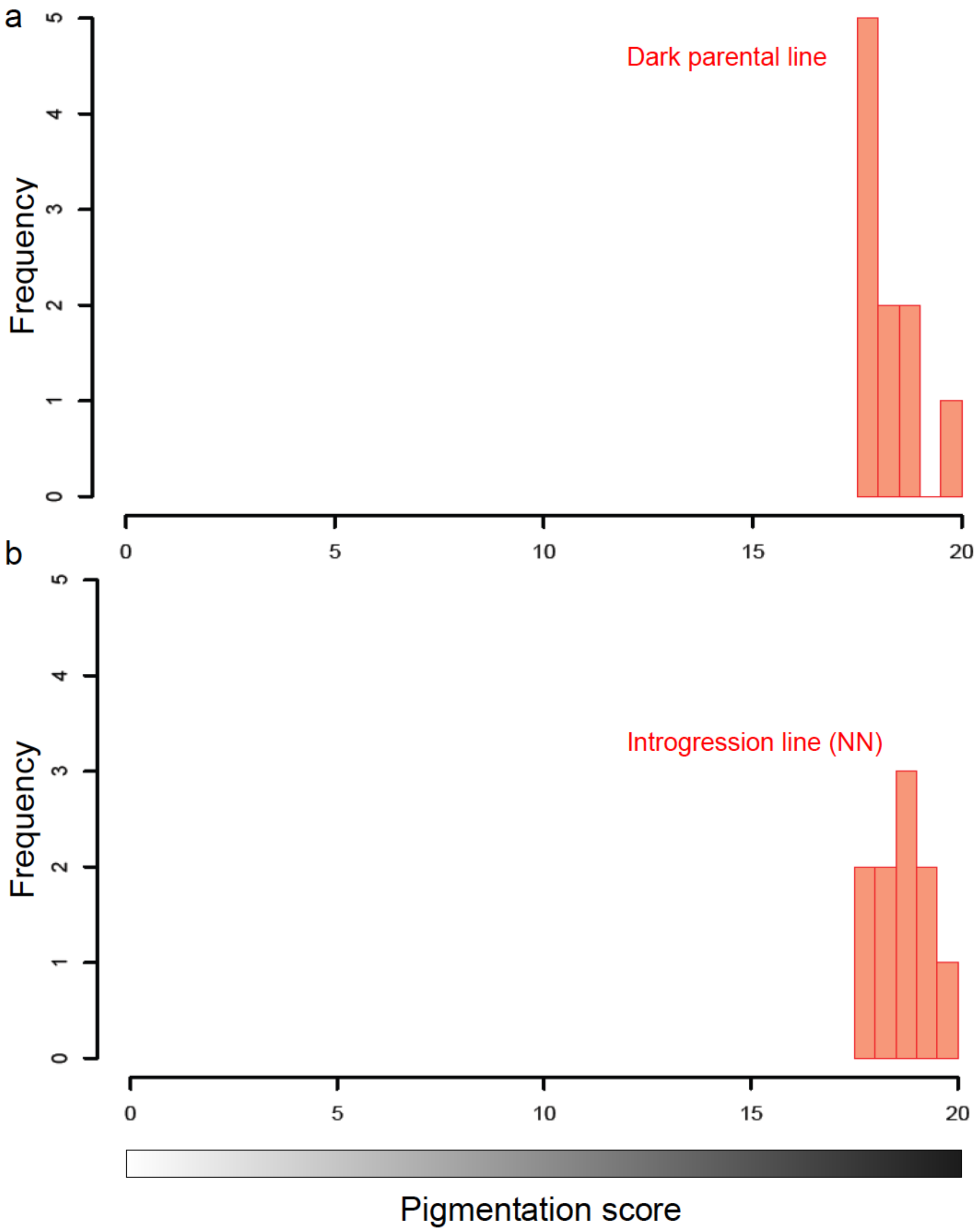

Supplementary Figure 1 | Introgression lines resemble dark parental flies. Histogram of pigmentation score on the last two abdominal segments in 10 females grown under similar conditions from (**a**) dark parental line and (**b**) dark introgression line after 12 generations of backcrosses into a light parental line. No significant difference is found.

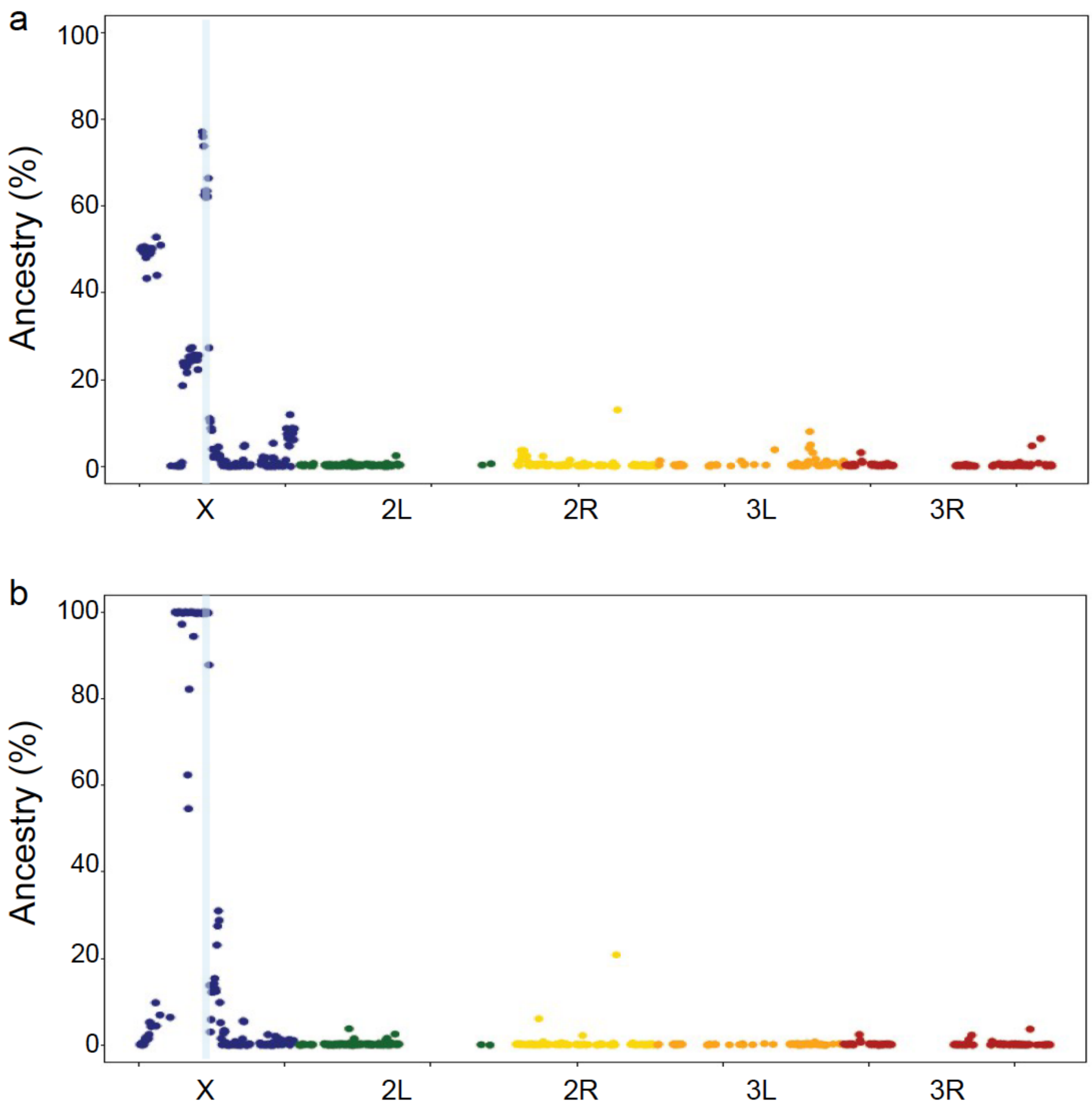

Supplementary Figure 2 | Genetic mapping of the FLCD locus. a,b, Ancestry proportion of the dark parental line in (**a**) heterozygous dark 7th backcross generation and (**b**) flies selected for dark pigmentation and inbred after 12 generations of backcrossing. Each dot corresponds to a 100-kb window, and each chromosome arm is given by a different colour. Windows with identity-by-descent between parental strains were excluded.

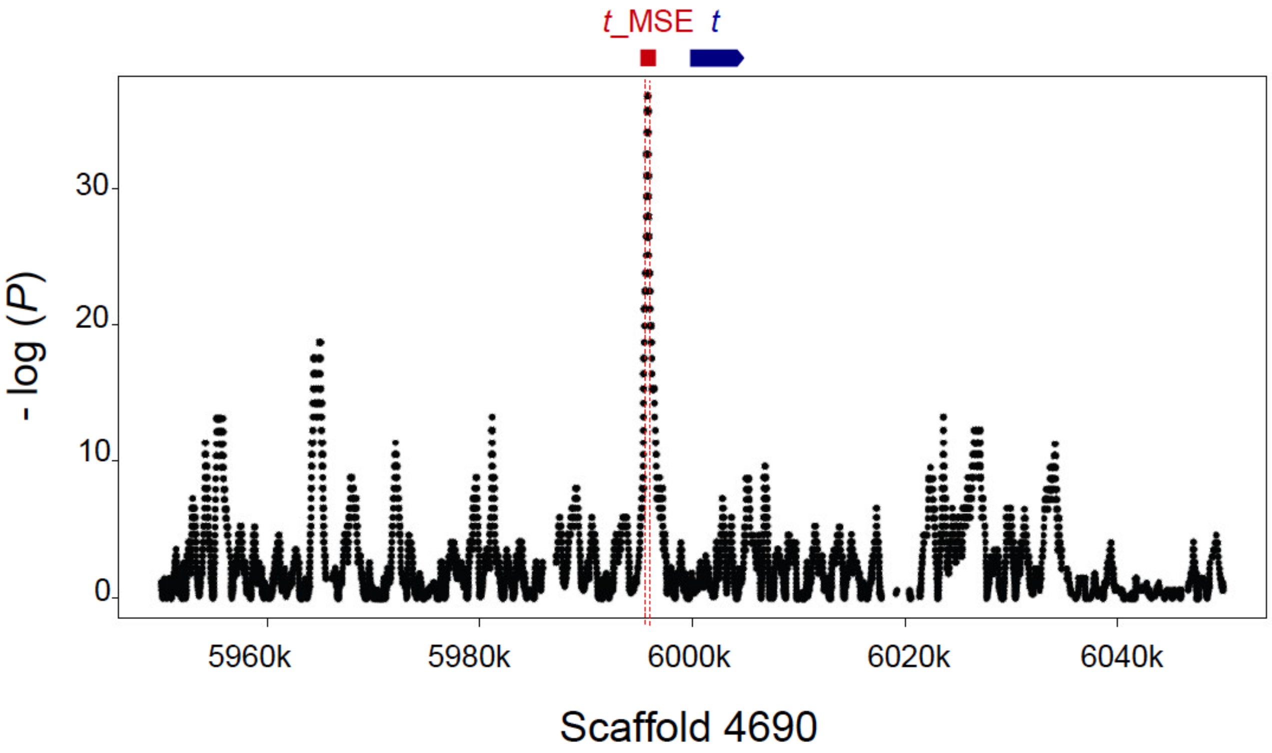

Supplementary Figure 3 | Localised excess polymorphism at the *t*_MSE-containing locus. Sliding HKA-like profile (-log *P*-value of a χ^2 test) in a 100-kb interval of scaffold_4690 of the reference genome centred on *tan*. Each dot corresponds to the -log *P* value of 101 SNPs with the dot position corresponding to the median SNP. The *tan* male-specific-enhancer (*t*_MSE) region is highlighted in red.

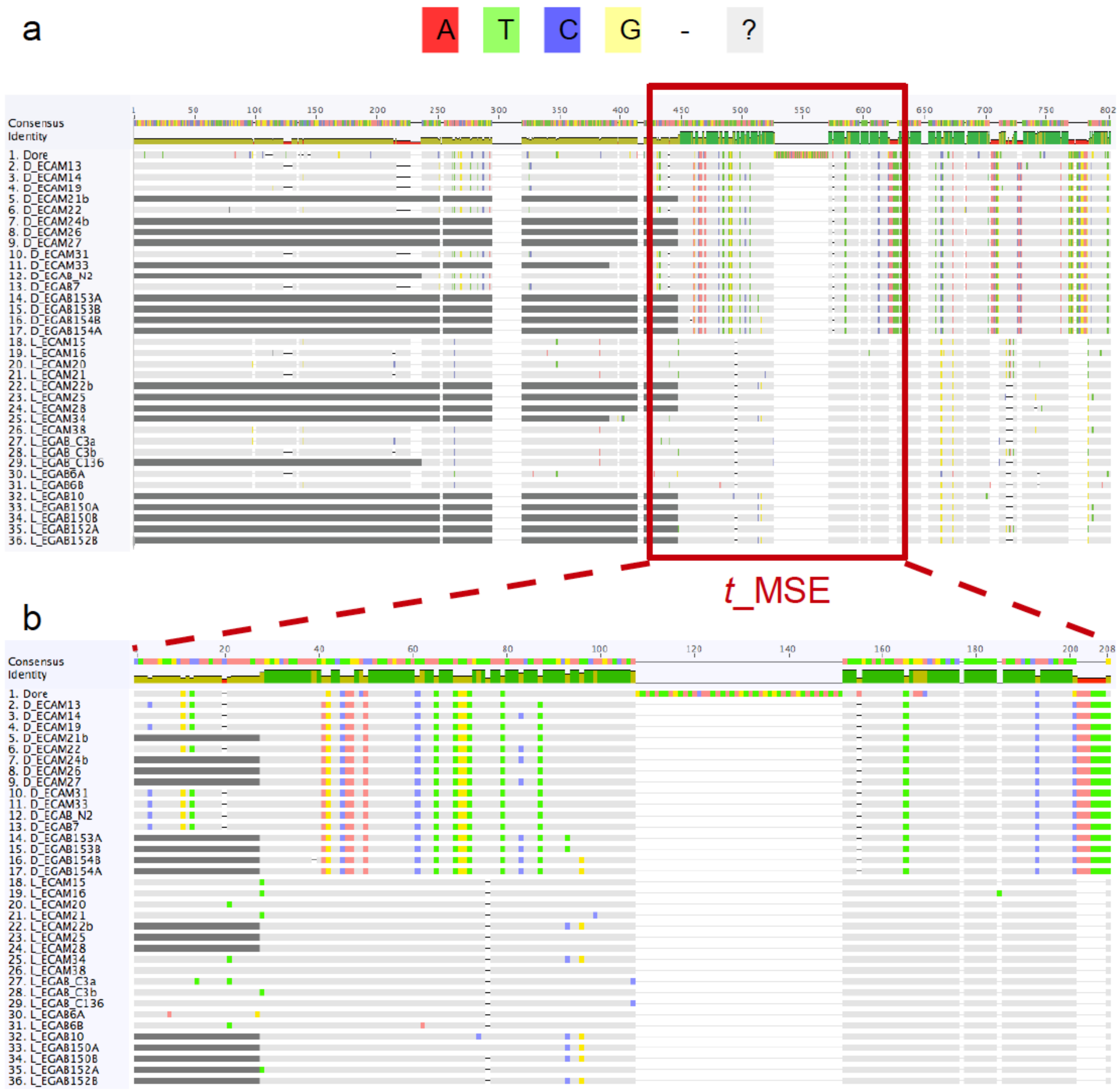

Supplementary Figure 4 | Sequence differences between dark and light haplogroups in 36 wild-caught flies. Alignment at (**a**) the 1 kb long *t*_MSE-containing locus, and (**b**) the ~150 bp region of *t*_MSE. Alleles differing from the consensus sequence are coloured. Dore= D. orena, D_ = dark haplogroup, L_= light haplogroup, ECAM = erecta from Cameroon, EGAB = erecta from Gabon.

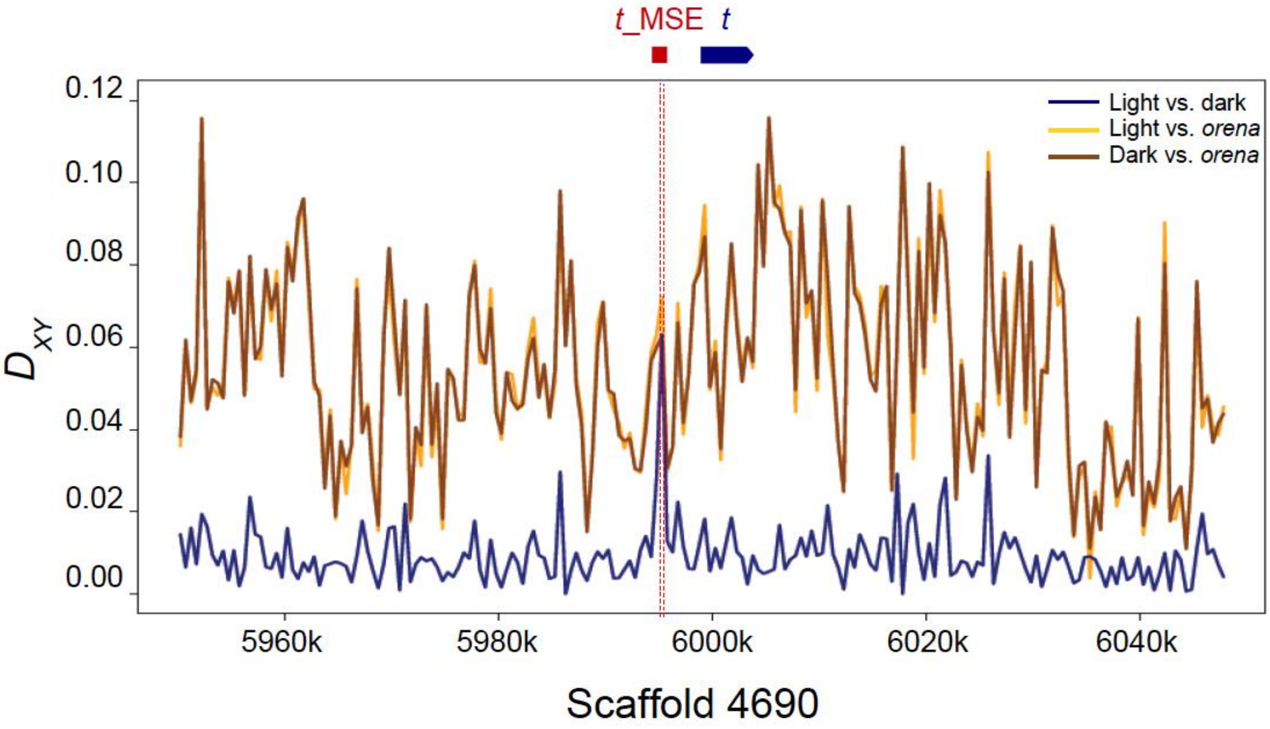

Supplementary Figure 5 | Localised incomplete lineage sorting between *D. erecta* and *D. orena* at the *t*_MSE region. Genetic divergence (D_{xy}) between pools of the two parental dark and light lines (blue) and between the two lines and *D. orena* (brown and orange, respectively) in a 100-kb interval of scaffold_4690 of the reference genome centred on *tan*. The *t*_MSE region is highlighted in red.

Supplementary Table 1 | SNP-based HKA-like test in a 100-kb long window centred on *tan*.

For each SNP, counts of polymorphic and divergent SNPS at the SNP ± 50 SNPs (*i.e.* windows of 101 SNPs). X chromosome-wide expected polymorphism = 50.9459551749531; X chromosome-wide expected divergence = 50.0540448250469. P-value: n.s. = not significant, * < 0.05, ** < 0.01, *** < 0.001.

Scaffold	Location	Polymorphism	Divergence	Chi-square	P-value
4690	5950008	56	45	1.0116974731171	n.s.
4690	5950018	56	45	1.0116974731171	n.s.
4690	5950026	55	46	0.650952919406731	n.s.
4690	5950054	54	47	0.369422463837495	n.s.
4690	5950083	54	47	0.369422463837495	n.s.
4690	5950087	54	47	0.369422463837495	n.s.
4690	5950099	53	48	0.167106106409387	n.s.
4690	5950128	52	49	0.044003847122408	n.s.
4690	5950140	52	49	0.044003847122408	n.s.
4690	5950155	51	50	0.000115685976557532	n.s.
4690	5950172	50	51	0.0354416229718356	n.s.
4690	5950179	49	52	0.149981658108242	n.s.
4690	5950185	50	51	0.0354416229718356	n.s.
4690	5950197	49	52	0.149981658108242	n.s.
4690	5950218	48	53	0.343735791385777	n.s.
4690	5950227	47	54	0.616704022804441	n.s.
4690	5950251	46	55	0.968886352364233	n.s.
4690	5950269	45	56	1.40028278006515	n.s.
4690	5950278	46	55	0.968886352364233	n.s.
4690	5950281	46	55	0.968886352364233	n.s.
4690	5950293	47	54	0.616704022804441	n.s.
4690	5950318	47	54	0.616704022804441	n.s.
4690	5950356	48	53	0.343735791385777	n.s.
4690	5950371	49	52	0.149981658108242	n.s.
4690	5950373	49	52	0.149981658108242	n.s.
4690	5950413	49	52	0.149981658108242	n.s.
4690	5950420	49	52	0.149981658108242	n.s.
4690	5950425	49	52	0.149981658108242	n.s.
4690	5950431	50	51	0.0354416229718356	n.s.
4690	5950443	50	51	0.0354416229718356	n.s.
4690	5950450	49	52	0.149981658108242	n.s.
4690	5950476	49	52	0.149981658108242	n.s.
4690	5950485	50	51	0.0354416229718356	n.s.
4690	5950494	50	51	0.0354416229718356	n.s.
4690	5950508	51	50	0.000115685976557532	n.s.
4690	5950511	52	49	0.044003847122408	n.s.
4690	5950525	52	49	0.044003847122408	n.s.
4690	5950562	52	49	0.044003847122408	n.s.
4690	5950624	52	49	0.044003847122408	n.s.

4690	5950626	53	48	0.167106106409387	n.s.
4690	5950630	54	47	0.369422463837495	n.s.
4690	5950632	53	48	0.167106106409387	n.s.
4690	5950635	53	48	0.167106106409387	n.s.
4690	5950644	54	47	0.369422463837495	n.s.
4690	5950667	54	47	0.369422463837495	n.s.
4690	5950673	55	46	0.650952919406731	n.s.
4690	5950706	56	45	1.0116974731171	n.s.
4690	5950721	57	44	1.45165612496859	n.s.
4690	5950724	56	45	1.0116974731171	n.s.
4690	5950740	56	45	1.0116974731171	n.s.
4690	5950741	56	45	1.0116974731171	n.s.
4690	5950745	56	45	1.0116974731171	n.s.
4690	5950750	56	45	1.0116974731171	n.s.
4690	5950753	55	46	0.650952919406731	n.s.
4690	5950822	54	47	0.369422463837495	n.s.
4690	5950832	55	46	0.650952919406731	n.s.
4690	5950837	55	46	0.650952919406731	n.s.
4690	5950885	54	47	0.369422463837495	n.s.
4690	5950886	53	48	0.167106106409387	n.s.
4690	5950906	54	47	0.369422463837495	n.s.
4690	5950927	53	48	0.167106106409387	n.s.
4690	5950934	52	49	0.044003847122408	n.s.
4690	5950948	52	49	0.044003847122408	n.s.
4690	5950968	51	50	0.000115685976557532	n.s.
4690	5950975	51	50	0.000115685976557532	n.s.
4690	5950978	51	50	0.000115685976557532	n.s.
4690	5950992	51	50	0.000115685976557532	n.s.
4690	5951000	52	49	0.044003847122408	n.s.
4690	5951024	52	49	0.044003847122408	n.s.
4690	5951043	53	48	0.167106106409387	n.s.
4690	5951044	54	47	0.369422463837495	n.s.
4690	5951064	53	48	0.167106106409387	n.s.
4690	5951073	54	47	0.369422463837495	n.s.
4690	5951076	55	46	0.650952919406731	n.s.
4690	5951085	56	45	1.0116974731171	n.s.
4690	5951089	56	45	1.0116974731171	n.s.
4690	5951093	57	44	1.45165612496859	n.s.
4690	5951104	57	44	1.45165612496859	n.s.
4690	5951112	57	44	1.45165612496859	n.s.
4690	5951117	56	45	1.0116974731171	n.s.
4690	5951121	55	46	0.650952919406731	n.s.
4690	5951126	55	46	0.650952919406731	n.s.
4690	5951146	56	45	1.0116974731171	n.s.
4690	5951150	56	45	1.0116974731171	n.s.
4690	5951151	56	45	1.0116974731171	n.s.
4690	5951154	56	45	1.0116974731171	n.s.
4690	5951198	56	45	1.0116974731171	n.s.
4690	5951207	56	45	1.0116974731171	n.s.
4690	5951208	57	44	1.45165612496859	n.s.

4690	5951210	58	43	1.97082887496121	n.s.
4690	5951214	58	43	1.97082887496121	n.s.
4690	5951232	58	43	1.97082887496121	n.s.
4690	5951233	57	44	1.45165612496859	n.s.
4690	5951235	58	43	1.97082887496121	n.s.
4690	5951241	58	43	1.97082887496121	n.s.
4690	5951244	57	44	1.45165612496859	n.s.
4690	5951248	57	44	1.45165612496859	n.s.
4690	5951258	57	44	1.45165612496859	n.s.
4690	5951265	57	44	1.45165612496859	n.s.
4690	5951283	58	43	1.97082887496121	n.s.
4690	5951284	59	42	2.56921572309496	n.s.
4690	5951293	60	41	3.24681666936984	n.s.
4690	5951321	60	41	3.24681666936984	n.s.
4690	5951350	60	41	3.24681666936984	n.s.
4690	5951359	60	41	3.24681666936984	n.s.
4690	5951360	59	42	2.56921572309496	n.s.
4690	5951371	58	43	1.97082887496121	n.s.
4690	5951373	59	42	2.56921572309496	n.s.
4690	5951388	59	42	2.56921572309496	n.s.
4690	5951400	59	42	2.56921572309496	n.s.
4690	5951408	59	42	2.56921572309496	n.s.
4690	5951427	60	41	3.24681666936984	n.s.
4690	5951441	61	40	4.00363171378585	*
4690	5951447	60	41	3.24681666936984	n.s.
4690	5951457	60	41	3.24681666936984	n.s.
4690	5951465	60	41	3.24681666936984	n.s.
4690	5951466	61	40	4.00363171378585	*
4690	5951467	61	40	4.00363171378585	*
4690	5951470	62	39	4.83966085634298	*
4690	5951476	61	40	4.00363171378585	*
4690	5951489	61	40	4.00363171378585	*
4690	5951505	60	41	3.24681666936984	n.s.
4690	5951508	60	41	3.24681666936984	n.s.
4690	5951523	59	42	2.56921572309496	n.s.
4690	5951532	58	43	1.97082887496121	n.s.
4690	5951535	58	43	1.97082887496121	n.s.
4690	5951538	57	44	1.45165612496859	n.s.
4690	5951542	57	44	1.45165612496859	n.s.
4690	5951545	57	44	1.45165612496859	n.s.
4690	5951555	56	45	1.0116974731171	n.s.
4690	5951560	55	46	0.650952919406731	n.s.
4690	5951567	55	46	0.650952919406731	n.s.
4690	5951570	56	45	1.0116974731171	n.s.
4690	5951588	56	45	1.0116974731171	n.s.
4690	5951603	56	45	1.0116974731171	n.s.
4690	5951604	56	45	1.0116974731171	n.s.
4690	5951608	55	46	0.650952919406731	n.s.
4690	5951623	54	47	0.369422463837495	n.s.
4690	5951629	54	47	0.369422463837495	n.s.

4690	5951636	53	48	0.167106106409387	n.s.	
4690	5951637	53	48	0.167106106409387	n.s.	
4690	5951645	53	48	0.167106106409387	n.s.	
4690	5951649	54	47	0.369422463837495	n.s.	
4690	5951678	54	47	0.369422463837495	n.s.	
4690	5951722	54	47	0.369422463837495	n.s.	
4690	5951723	55	46	0.650952919406731	n.s.	
4690	5951734	54	47	0.369422463837495	n.s.	
4690	5951747	54	47	0.369422463837495	n.s.	
4690	5951748	54	47	0.369422463837495	n.s.	
4690	5951763	54	47	0.369422463837495	n.s.	
4690	5951765	55	46	0.650952919406731	n.s.	
4690	5951782	55	46	0.650952919406731	n.s.	
4690	5951788	55	46	0.650952919406731	n.s.	
4690	5951791	55	46	0.650952919406731	n.s.	
4690	5951806	56	45	1.0116974731171	n.s.	
4690	5951840	57	44	1.45165612496859	n.s.	
4690	5951841	57	44	1.45165612496859	n.s.	
4690	5951848	58	43	1.97082887496121	n.s.	
4690	5951854	58	43	1.97082887496121	n.s.	
4690	5951871	59	42	2.56921572309496	n.s.	
4690	5951872	59	42	2.56921572309496	n.s.	
4690	5951915	59	42	2.56921572309496	n.s.	
4690	5951922	59	42	2.56921572309496	n.s.	
4690	5951941	58	43	1.97082887496121	n.s.	
4690	5951950	58	43	1.97082887496121	n.s.	
4690	5951962	57	44	1.45165612496859	n.s.	
4690	5951979	56	45	1.0116974731171	n.s.	
4690	5951987	55	46	0.650952919406731	n.s.	
4690	5951992	54	47	0.369422463837495	n.s.	
4690	5952003	53	48	0.167106106409387	n.s.	
4690	5952008	52	49	0.044003847122408	n.s.	
4690	5952017	51	50	0.000115685976557532		n.s.
4690	5952021	51	50	0.000115685976557532		n.s.
4690	5952035	50	51	0.0354416229718356	n.s.	
4690	5952045	49	52	0.149981658108242	n.s.	
4690	5952051	49	52	0.149981658108242	n.s.	
4690	5952066	48	53	0.343735791385777	n.s.	
4690	5952112	47	54	0.616704022804441	n.s.	
4690	5952114	46	55	0.968886352364233	n.s.	
4690	5952116	45	56	1.40028278006515	n.s.	
4690	5952126	45	56	1.40028278006515	n.s.	
4690	5952127	45	56	1.40028278006515	n.s.	
4690	5952133	44	57	1.9108933059072	n.s.	
4690	5952134	44	57	1.9108933059072	n.s.	
4690	5952137	44	57	1.9108933059072	n.s.	
4690	5952143	44	57	1.9108933059072	n.s.	
4690	5952150	44	57	1.9108933059072	n.s.	
4690	5952157	43	58	2.50071792989038	n.s.	
4690	5952158	43	58	2.50071792989038	n.s.	

4690	5952166	43	58	2.50071792989038	n.s.
4690	5952176	42	59	3.16975665201469	n.s.
4690	5952181	43	58	2.50071792989038	n.s.
4690	5952185	43	58	2.50071792989038	n.s.
4690	5952187	43	58	2.50071792989038	n.s.
4690	5952193	43	58	2.50071792989038	n.s.
4690	5952196	44	57	1.9108933059072	n.s.
4690	5952201	44	57	1.9108933059072	n.s.
4690	5952202	45	56	1.40028278006515	n.s.
4690	5952204	45	56	1.40028278006515	n.s.
4690	5952209	44	57	1.9108933059072	n.s.
4690	5952211	44	57	1.9108933059072	n.s.
4690	5952212	44	57	1.9108933059072	n.s.
4690	5952220	44	57	1.9108933059072	n.s.
4690	5952224	45	56	1.40028278006515	n.s.
4690	5952241	46	55	0.968886352364233	n.s.
4690	5952248	47	54	0.616704022804441	n.s.
4690	5952277	48	53	0.343735791385777	n.s.
4690	5952290	49	52	0.149981658108242	n.s.
4690	5952291	49	52	0.149981658108242	n.s.
4690	5952294	49	52	0.149981658108242	n.s.
4690	5952300	49	52	0.149981658108242	n.s.
4690	5952308	50	51	0.0354416229718356	n.s.
4690	5952321	50	51	0.0354416229718356	n.s.
4690	5952326	50	51	0.0354416229718356	n.s.
4690	5952332	51	50	0.000115685976557532	n.s.
4690	5952360	52	49	0.044003847122408	n.s.
4690	5952369	53	48	0.167106106409387	n.s.
4690	5952371	53	48	0.167106106409387	n.s.
4690	5952378	54	47	0.369422463837495	n.s.
4690	5952396	54	47	0.369422463837495	n.s.
4690	5952397	55	46	0.650952919406731	n.s.
4690	5952403	56	45	1.0116974731171	n.s.
4690	5952405	57	44	1.45165612496859	n.s.
4690	5952410	57	44	1.45165612496859	n.s.
4690	5952419	58	43	1.97082887496121	n.s.
4690	5952421	59	42	2.56921572309496	n.s.
4690	5952432	60	41	3.24681666936984	n.s.
4690	5952450	60	41	3.24681666936984	n.s.
4690	5952475	60	41	3.24681666936984	n.s.
4690	5952485	60	41	3.24681666936984	n.s.
4690	5952486	61	40	4.00363171378585	*
4690	5952489	61	40	4.00363171378585	*
4690	5952506	61	40	4.00363171378585	*
4690	5952511	61	40	4.00363171378585	*
4690	5952512	61	40	4.00363171378585	*
4690	5952514	61	40	4.00363171378585	*
4690	5952565	61	40	4.00363171378585	*
4690	5952574	61	40	4.00363171378585	*
4690	5952589	62	39	4.83966085634298	*

4690	5952644	62	39	4.83966085634298	*
4690	5952651	63	38	5.75490409704125	*
4690	5952663	63	38	5.75490409704125	*
4690	5952674	63	38	5.75490409704125	*
4690	5952685	63	38	5.75490409704125	*
4690	5952689	62	39	4.83966085634298	*
4690	5952717	62	39	4.83966085634298	*
4690	5952719	61	40	4.00363171378585	*
4690	5952727	61	40	4.00363171378585	*
4690	5952738	60	41	3.24681666936984	n.s.
4690	5952744	60	41	3.24681666936984	n.s.
4690	5952747	61	40	4.00363171378585	*
4690	5952753	61	40	4.00363171378585	*
4690	5952754	61	40	4.00363171378585	*
4690	5952755	62	39	4.83966085634298	*
4690	5952760	61	40	4.00363171378585	*
4690	5952764	60	41	3.24681666936984	n.s.
4690	5952773	60	41	3.24681666936984	n.s.
4690	5952800	59	42	2.56921572309496	n.s.
4690	5952803	59	42	2.56921572309496	n.s.
4690	5952824	59	42	2.56921572309496	n.s.
4690	5952827	58	43	1.97082887496121	n.s.
4690	5952838	58	43	1.97082887496121	n.s.
4690	5952843	59	42	2.56921572309496	n.s.
4690	5952852	60	41	3.24681666936984	n.s.
4690	5952854	61	40	4.00363171378585	*
4690	5952861	61	40	4.00363171378585	*
4690	5952870	61	40	4.00363171378585	*
4690	5952878	61	40	4.00363171378585	*
4690	5952887	62	39	4.83966085634298	*
4690	5952902	62	39	4.83966085634298	*
4690	5952908	63	38	5.75490409704125	*
4690	5952911	64	37	6.74936143588064	**
4690	5952920	64	37	6.74936143588064	**
4690	5952921	65	36	7.82303287286116	**
4690	5952926	65	36	7.82303287286116	**
4690	5952938	66	35	8.97591840798281	**
4690	5952941	65	36	7.82303287286116	**
4690	5952944	66	35	8.97591840798281	**
4690	5952947	67	34	10.2080180412456	**
4690	5952962	67	34	10.2080180412456	**
4690	5952983	67	34	10.2080180412456	**
4690	5952993	67	34	10.2080180412456	**
4690	5952996	67	34	10.2080180412456	**
4690	5952997	68	33	11.5193317726495	***
4690	5953007	67	34	10.2080180412456	**
4690	5953009	67	34	10.2080180412456	**
4690	5953049	66	35	8.97591840798281	**
4690	5953053	65	36	7.82303287286116	**
4690	5953061	66	35	8.97591840798281	**

4690	5953063	67	34	10.2080180412456	**
4690	5953074	67	34	10.2080180412456	**
4690	5953082	67	34	10.2080180412456	**
4690	5953093	66	35	8.97591840798281	**
4690	5953094	66	35	8.97591840798281	**
4690	5953130	67	34	10.2080180412456	**
4690	5953133	66	35	8.97591840798281	**
4690	5953136	66	35	8.97591840798281	**
4690	5953151	67	34	10.2080180412456	**
4690	5953164	66	35	8.97591840798281	**
4690	5953175	65	36	7.82303287286116	**
4690	5953185	66	35	8.97591840798281	**
4690	5953208	66	35	8.97591840798281	**
4690	5953220	66	35	8.97591840798281	**
4690	5953223	65	36	7.82303287286116	**
4690	5953234	65	36	7.82303287286116	**
4690	5953235	65	36	7.82303287286116	**
4690	5953241	64	37	6.74936143588064	**
4690	5953255	64	37	6.74936143588064	**
4690	5953258	64	37	6.74936143588064	**
4690	5953270	63	38	5.75490409704125	*
4690	5953273	64	37	6.74936143588064	**
4690	5953280	64	37	6.74936143588064	**
4690	5953292	63	38	5.75490409704125	*
4690	5953294	63	38	5.75490409704125	*
4690	5953313	62	39	4.83966085634298	*
4690	5953315	61	40	4.00363171378585	*
4690	5953319	60	41	3.24681666936984	n.s.
4690	5953320	60	41	3.24681666936984	n.s.
4690	5953345	59	42	2.56921572309496	n.s.
4690	5953347	59	42	2.56921572309496	n.s.
4690	5953362	59	42	2.56921572309496	n.s.
4690	5953378	58	43	1.97082887496121	n.s.
4690	5953379	58	43	1.97082887496121	n.s.
4690	5953385	58	43	1.97082887496121	n.s.
4690	5953410	58	43	1.97082887496121	n.s.
4690	5953428	58	43	1.97082887496121	n.s.
4690	5953430	57	44	1.45165612496859	n.s.
4690	5953474	56	45	1.0116974731171	n.s.
4690	5953482	57	44	1.45165612496859	n.s.
4690	5953485	56	45	1.0116974731171	n.s.
4690	5953489	56	45	1.0116974731171	n.s.
4690	5953491	56	45	1.0116974731171	n.s.
4690	5953500	56	45	1.0116974731171	n.s.
4690	5953506	57	44	1.45165612496859	n.s.
4690	5953511	57	44	1.45165612496859	n.s.
4690	5953519	56	45	1.0116974731171	n.s.
4690	5953520	55	46	0.650952919406731	n.s.
4690	5953521	55	46	0.650952919406731	n.s.
4690	5953530	55	46	0.650952919406731	n.s.

4690	5953550	54	47	0.369422463837495	n.s.
4690	5953561	54	47	0.369422463837495	n.s.
4690	5953573	54	47	0.369422463837495	n.s.
4690	5953579	53	48	0.167106106409387	n.s.
4690	5953581	53	48	0.167106106409387	n.s.
4690	5953592	52	49	0.044003847122408	n.s.
4690	5953597	53	48	0.167106106409387	n.s.
4690	5953602	53	48	0.167106106409387	n.s.
4690	5953609	54	47	0.369422463837495	n.s.
4690	5953612	55	46	0.650952919406731	n.s.
4690	5953626	56	45	1.0116974731171	n.s.
4690	5953633	56	45	1.0116974731171	n.s.
4690	5953648	56	45	1.0116974731171	n.s.
4690	5953650	55	46	0.650952919406731	n.s.
4690	5953654	55	46	0.650952919406731	n.s.
4690	5953659	55	46	0.650952919406731	n.s.
4690	5953664	55	46	0.650952919406731	n.s.
4690	5953670	55	46	0.650952919406731	n.s.
4690	5953676	55	46	0.650952919406731	n.s.
4690	5953685	56	45	1.0116974731171	n.s.
4690	5953707	56	45	1.0116974731171	n.s.
4690	5953718	57	44	1.45165612496859	n.s.
4690	5953789	58	43	1.97082887496121	n.s.
4690	5953801	58	43	1.97082887496121	n.s.
4690	5953824	58	43	1.97082887496121	n.s.
4690	5953846	58	43	1.97082887496121	n.s.
4690	5953879	58	43	1.97082887496121	n.s.
4690	5953888	59	42	2.56921572309496	n.s.
4690	5953898	60	41	3.24681666936984	n.s.
4690	5953906	61	40	4.00363171378585	*
4690	5953909	61	40	4.00363171378585	*
4690	5953917	62	39	4.83966085634298	*
4690	5953918	62	39	4.83966085634298	*
4690	5953929	62	39	4.83966085634298	*
4690	5953962	63	38	5.75490409704125	*
4690	5953963	63	38	5.75490409704125	*
4690	5954005	64	37	6.74936143588064	**
4690	5954008	64	37	6.74936143588064	**
4690	5954022	65	36	7.82303287286116	**
4690	5954023	65	36	7.82303287286116	**
4690	5954034	65	36	7.82303287286116	**
4690	5954088	66	35	8.97591840798281	**
4690	5954101	67	34	10.2080180412456	**
4690	5954107	68	33	11.5193317726495	***
4690	5954122	69	32	12.9098596021945	***
4690	5954125	69	32	12.9098596021945	***
4690	5954134	70	31	14.3796015298807	***
4690	5954146	71	30	15.928557555708	***
4690	5954152	72	29	17.5567276796764	***
4690	5954159	73	28	19.264111901786	***

4690	5954174	73	28	19.264111901786	***
4690	5954177	73	28	19.264111901786	***
4690	5954180	72	29	17.5567276796764	***
4690	5954182	72	29	17.5567276796764	***
4690	5954192	72	29	17.5567276796764	***
4690	5954215	71	30	15.928557555708	***
4690	5954237	71	30	15.928557555708	***
4690	5954245	71	30	15.928557555708	***
4690	5954250	70	31	14.3796015298807	***
4690	5954266	70	31	14.3796015298807	***
4690	5954285	71	30	15.928557555708	***
4690	5954289	71	30	15.928557555708	***
4690	5954292	70	31	14.3796015298807	***
4690	5954311	69	32	12.9098596021945	***
4690	5954313	69	32	12.9098596021945	***
4690	5954344	70	31	14.3796015298807	***
4690	5954353	69	32	12.9098596021945	***
4690	5954363	68	33	11.5193317726495	***
4690	5954364	69	32	12.9098596021945	***
4690	5954373	68	33	11.5193317726495	***
4690	5954376	68	33	11.5193317726495	***
4690	5954377	68	33	11.5193317726495	***
4690	5954389	67	34	10.2080180412456	**
4690	5954395	66	35	8.97591840798281	**
4690	5954398	66	35	8.97591840798281	**
4690	5954401	65	36	7.82303287286116	**
4690	5954411	66	35	8.97591840798281	**
4690	5954417	66	35	8.97591840798281	**
4690	5954418	66	35	8.97591840798281	**
4690	5954419	65	36	7.82303287286116	**
4690	5954421	65	36	7.82303287286116	**
4690	5954422	64	37	6.74936143588064	**
4690	5954424	63	38	5.75490409704125	*
4690	5954430	64	37	6.74936143588064	**
4690	5954443	64	37	6.74936143588064	**
4690	5954455	63	38	5.75490409704125	*
4690	5954461	63	38	5.75490409704125	*
4690	5954464	63	38	5.75490409704125	*
4690	5954466	63	38	5.75490409704125	*
4690	5954467	64	37	6.74936143588064	**
4690	5954469	63	38	5.75490409704125	*
4690	5954476	64	37	6.74936143588064	**
4690	5954487	64	37	6.74936143588064	**
4690	5954500	63	38	5.75490409704125	*
4690	5954505	63	38	5.75490409704125	*
4690	5954506	62	39	4.83966085634298	*
4690	5954511	62	39	4.83966085634298	*
4690	5954525	62	39	4.83966085634298	*
4690	5954560	63	38	5.75490409704125	*
4690	5954562	62	39	4.83966085634298	*

4690	5954578	63	38	5.75490409704125	*
4690	5954581	63	38	5.75490409704125	*
4690	5954605	63	38	5.75490409704125	*
4690	5954611	62	39	4.83966085634298	*
4690	5954620	63	38	5.75490409704125	*
4690	5954625	62	39	4.83966085634298	*
4690	5954632	62	39	4.83966085634298	*
4690	5954635	61	40	4.00363171378585	*
4690	5954641	60	41	3.24681666936984	n.s.
4690	5954661	59	42	2.56921572309496	n.s.
4690	5954666	59	42	2.56921572309496	n.s.
4690	5954691	59	42	2.56921572309496	n.s.
4690	5954693	58	43	1.97082887496121	n.s.
4690	5954706	57	44	1.45165612496859	n.s.
4690	5954720	58	43	1.97082887496121	n.s.
4690	5954731	57	44	1.45165612496859	n.s.
4690	5954732	56	45	1.0116974731171	n.s.
4690	5954736	57	44	1.45165612496859	n.s.
4690	5954751	57	44	1.45165612496859	n.s.
4690	5954764	56	45	1.0116974731171	n.s.
4690	5954771	55	46	0.650952919406731	n.s.
4690	5954772	54	47	0.369422463837495	n.s.
4690	5954807	53	48	0.167106106409387	n.s.
4690	5954808	52	49	0.044003847122408	n.s.
4690	5954846	52	49	0.044003847122408	n.s.
4690	5954863	51	50	0.000115685976557532	n.s.
4690	5954933	50	51	0.0354416229718356	n.s.
4690	5954936	49	52	0.149981658108242	n.s.
4690	5954945	48	53	0.343735791385777	n.s.
4690	5954952	47	54	0.616704022804441	n.s.
4690	5954955	46	55	0.968886352364233	n.s.
4690	5954961	45	56	1.40028278006515	n.s.
4690	5954978	44	57	1.9108933059072	n.s.
4690	5954979	43	58	2.50071792989038	n.s.
4690	5954988	42	59	3.16975665201469	n.s.
4690	5954995	42	59	3.16975665201469	n.s.
4690	5955012	42	59	3.16975665201469	n.s.
4690	5955031	41	60	3.91800947228012	*
4690	5955043	40	61	4.74547639068669	*
4690	5955048	39	62	5.65215740723438	*
4690	5955050	38	63	6.6380525219232	*
4690	5955067	37	64	7.70316173475315	**
4690	5955075	36	65	8.84748504572423	**
4690	5955081	35	66	10.0710224548364	**
4690	5955108	34	67	11.3737739620898	***
4690	5955115	33	68	12.7557395674842	***
4690	5955119	32	69	14.2169192710198	***
4690	5955174	31	70	15.7573130726965	***
4690	5955176	30	71	17.3769209725144	***
4690	5955181	30	71	17.3769209725144	***

4690	5955194	29	72	19.0757429704734	***
4690	5955205	28	73	20.8537790665735	***
4690	5955211	27	74	22.7110292608147	***
4690	5955214	27	74	22.7110292608147	***
4690	5955250	27	74	22.7110292608147	***
4690	5955284	27	74	22.7110292608147	***
4690	5955290	27	74	22.7110292608147	***
4690	5955293	27	74	22.7110292608147	***
4690	5955316	28	73	20.8537790665735	***
4690	5955317	28	73	20.8537790665735	***
4690	5955339	27	74	22.7110292608147	***
4690	5955359	27	74	22.7110292608147	***
4690	5955379	27	74	22.7110292608147	***
4690	5955380	27	74	22.7110292608147	***
4690	5955405	28	73	20.8537790665735	***
4690	5955426	27	74	22.7110292608147	***
4690	5955427	27	74	22.7110292608147	***
4690	5955434	27	74	22.7110292608147	***
4690	5955484	28	73	20.8537790665735	***
4690	5955496	28	73	20.8537790665735	***
4690	5955497	28	73	20.8537790665735	***
4690	5955499	27	74	22.7110292608147	***
4690	5955504	27	74	22.7110292608147	***
4690	5955526	27	74	22.7110292608147	***
4690	5955540	28	73	20.8537790665735	***
4690	5955569	29	72	19.0757429704734	***
4690	5955583	29	72	19.0757429704734	***
4690	5955584	28	73	20.8537790665735	***
4690	5955589	28	73	20.8537790665735	***
4690	5955605	29	72	19.0757429704734	***
4690	5955620	29	72	19.0757429704734	***
4690	5955621	30	71	17.3769209725144	***
4690	5955639	30	71	17.3769209725144	***
4690	5955656	31	70	15.7573130726965	***
4690	5955660	30	71	17.3769209725144	***
4690	5955668	30	71	17.3769209725144	***
4690	5955669	30	71	17.3769209725144	***
4690	5955690	29	72	19.0757429704734	***
4690	5955696	28	73	20.8537790665735	***
4690	5955699	29	72	19.0757429704734	***
4690	5955708	28	73	20.8537790665735	***
4690	5955739	29	72	19.0757429704734	***
4690	5955776	28	73	20.8537790665735	***
4690	5955784	27	74	22.7110292608147	***
4690	5955785	28	73	20.8537790665735	***
4690	5955799	29	72	19.0757429704734	***
4690	5955801	30	71	17.3769209725144	***
4690	5955805	30	71	17.3769209725144	***
4690	5955807	31	70	15.7573130726965	***
4690	5955817	31	70	15.7573130726965	***

4690	5955833	32	69	14.2169192710198	***
4690	5955838	32	69	14.2169192710198	***
4690	5955887	33	68	12.7557395674842	***
4690	5955890	33	68	12.7557395674842	***
4690	5955901	34	67	11.3737739620898	***
4690	5955928	33	68	12.7557395674842	***
4690	5955956	33	68	12.7557395674842	***
4690	5955958	34	67	11.3737739620898	***
4690	5955980	35	66	10.0710224548364	**
4690	5956042	35	66	10.0710224548364	**
4690	5956047	36	65	8.84748504572423	**
4690	5956054	35	66	10.0710224548364	**
4690	5956071	35	66	10.0710224548364	**
4690	5956078	35	66	10.0710224548364	**
4690	5956096	35	66	10.0710224548364	**
4690	5956097	35	66	10.0710224548364	**
4690	5956107	36	65	8.84748504572423	**
4690	5956115	36	65	8.84748504572423	**
4690	5956120	35	66	10.0710224548364	**
4690	5956126	35	66	10.0710224548364	**
4690	5956129	36	65	8.84748504572423	**
4690	5956152	37	64	7.70316173475315	**
4690	5956175	37	64	7.70316173475315	**
4690	5956187	38	63	6.6380525219232	*
4690	5956207	38	63	6.6380525219232	*
4690	5956230	37	64	7.70316173475315	**
4690	5956286	37	64	7.70316173475315	**
4690	5956291	37	64	7.70316173475315	**
4690	5956323	37	64	7.70316173475315	**
4690	5956331	38	63	6.6380525219232	*
4690	5956340	38	63	6.6380525219232	*
4690	5956370	39	62	5.65215740723438	*
4690	5956392	40	61	4.74547639068669	*
4690	5956436	40	61	4.74547639068669	*
4690	5956438	40	61	4.74547639068669	*
4690	5956493	41	60	3.91800947228012	*
4690	5956522	41	60	3.91800947228012	*
4690	5956544	41	60	3.91800947228012	*
4690	5956545	42	59	3.16975665201469	n.s.
4690	5956569	43	58	2.50071792989038	n.s.
4690	5956572	43	58	2.50071792989038	n.s.
4690	5956573	44	57	1.9108933059072	n.s.
4690	5956577	45	56	1.40028278006515	n.s.
4690	5956578	46	55	0.968886352364233	n.s.
4690	5956579	47	54	0.616704022804441	n.s.
4690	5956580	47	54	0.616704022804441	n.s.
4690	5956581	48	53	0.343735791385777	n.s.
4690	5956588	49	52	0.149981658108242	n.s.
4690	5956597	50	51	0.0354416229718356	n.s.
4690	5956641	50	51	0.0354416229718356	n.s.

4690	5956644	49	52	0.149981658108242	n.s.	
4690	5956663	50	51	0.0354416229718356	n.s.	
4690	5956682	51	50	0.000115685976557532		n.s.
4690	5956686	52	49	0.044003847122408	n.s.	
4690	5956701	53	48	0.167106106409387	n.s.	
4690	5956707	53	48	0.167106106409387	n.s.	
4690	5956712	53	48	0.167106106409387	n.s.	
4690	5956725	54	47	0.369422463837495	n.s.	
4690	5956728	54	47	0.369422463837495	n.s.	
4690	5956730	53	48	0.167106106409387	n.s.	
4690	5956735	54	47	0.369422463837495	n.s.	
4690	5956748	54	47	0.369422463837495	n.s.	
4690	5956757	53	48	0.167106106409387	n.s.	
4690	5956764	54	47	0.369422463837495	n.s.	
4690	5956767	55	46	0.650952919406731	n.s.	
4690	5956768	55	46	0.650952919406731	n.s.	
4690	5956771	55	46	0.650952919406731	n.s.	
4690	5956777	55	46	0.650952919406731	n.s.	
4690	5956778	56	45	1.0116974731171	n.s.	
4690	5956782	55	46	0.650952919406731	n.s.	
4690	5956787	54	47	0.369422463837495	n.s.	
4690	5956788	55	46	0.650952919406731	n.s.	
4690	5956823	56	45	1.0116974731171	n.s.	
4690	5956835	57	44	1.45165612496859	n.s.	
4690	5956851	58	43	1.97082887496121	n.s.	
4690	5956894	58	43	1.97082887496121	n.s.	
4690	5956895	57	44	1.45165612496859	n.s.	
4690	5956917	58	43	1.97082887496121	n.s.	
4690	5956919	58	43	1.97082887496121	n.s.	
4690	5956935	59	42	2.56921572309496	n.s.	
4690	5956939	58	43	1.97082887496121	n.s.	
4690	5956959	59	42	2.56921572309496	n.s.	
4690	5956997	59	42	2.56921572309496	n.s.	
4690	5957011	59	42	2.56921572309496	n.s.	
4690	5957013	59	42	2.56921572309496	n.s.	
4690	5957030	59	42	2.56921572309496	n.s.	
4690	5957040	58	43	1.97082887496121	n.s.	
4690	5957050	59	42	2.56921572309496	n.s.	
4690	5957064	59	42	2.56921572309496	n.s.	
4690	5957066	60	41	3.24681666936984	n.s.	
4690	5957068	60	41	3.24681666936984	n.s.	
4690	5957075	60	41	3.24681666936984	n.s.	
4690	5957081	60	41	3.24681666936984	n.s.	
4690	5957082	60	41	3.24681666936984	n.s.	
4690	5957090	61	40	4.00363171378585	*	
4690	5957094	60	41	3.24681666936984	n.s.	
4690	5957102	59	42	2.56921572309496	n.s.	
4690	5957112	58	43	1.97082887496121	n.s.	
4690	5957115	58	43	1.97082887496121	n.s.	
4690	5957127	58	43	1.97082887496121	n.s.	

4690	5957129	58	43	1.97082887496121	n.s.
4690	5957143	58	43	1.97082887496121	n.s.
4690	5957155	58	43	1.97082887496121	n.s.
4690	5957165	58	43	1.97082887496121	n.s.
4690	5957183	59	42	2.56921572309496	n.s.
4690	5957184	59	42	2.56921572309496	n.s.
4690	5957192	60	41	3.24681666936984	n.s.
4690	5957207	60	41	3.24681666936984	n.s.
4690	5957216	60	41	3.24681666936984	n.s.
4690	5957217	59	42	2.56921572309496	n.s.
4690	5957218	60	41	3.24681666936984	n.s.
4690	5957229	60	41	3.24681666936984	n.s.
4690	5957230	61	40	4.00363171378585	*
4690	5957233	61	40	4.00363171378585	*
4690	5957235	61	40	4.00363171378585	*
4690	5957238	62	39	4.83966085634298	*
4690	5957247	62	39	4.83966085634298	*
4690	5957268	61	40	4.00363171378585	*
4690	5957274	62	39	4.83966085634298	*
4690	5957306	63	38	5.75490409704125	*
4690	5957322	64	37	6.74936143588064	**
4690	5957324	64	37	6.74936143588064	**
4690	5957384	63	38	5.75490409704125	*
4690	5957386	63	38	5.75490409704125	*
4690	5957485	62	39	4.83966085634298	*
4690	5957501	63	38	5.75490409704125	*
4690	5957502	64	37	6.74936143588064	**
4690	5957503	64	37	6.74936143588064	**
4690	5957510	65	36	7.82303287286116	**
4690	5957514	65	36	7.82303287286116	**
4690	5957531	65	36	7.82303287286116	**
4690	5957580	65	36	7.82303287286116	**
4690	5957582	64	37	6.74936143588064	**
4690	5957596	63	38	5.75490409704125	*
4690	5957599	63	38	5.75490409704125	*
4690	5957602	64	37	6.74936143588064	**
4690	5957608	64	37	6.74936143588064	**
4690	5957616	64	37	6.74936143588064	**
4690	5957628	64	37	6.74936143588064	**
4690	5957637	63	38	5.75490409704125	*
4690	5957652	62	39	4.83966085634298	*
4690	5957653	63	38	5.75490409704125	*
4690	5957655	62	39	4.83966085634298	*
4690	5957669	61	40	4.00363171378585	*
4690	5957677	60	41	3.24681666936984	n.s.
4690	5957679	59	42	2.56921572309496	n.s.
4690	5957682	60	41	3.24681666936984	n.s.
4690	5957683	60	41	3.24681666936984	n.s.
4690	5957753	59	42	2.56921572309496	n.s.
4690	5957767	59	42	2.56921572309496	n.s.

4690	5957783	60	41	3.24681666936984	n.s.
4690	5957809	60	41	3.24681666936984	n.s.
4690	5957845	60	41	3.24681666936984	n.s.
4690	5957861	59	42	2.56921572309496	n.s.
4690	5957867	58	43	1.97082887496121	n.s.
4690	5957871	58	43	1.97082887496121	n.s.
4690	5957872	59	42	2.56921572309496	n.s.
4690	5957889	59	42	2.56921572309496	n.s.
4690	5957911	59	42	2.56921572309496	n.s.
4690	5957917	59	42	2.56921572309496	n.s.
4690	5957972	60	41	3.24681666936984	n.s.
4690	5957973	59	42	2.56921572309496	n.s.
4690	5957977	59	42	2.56921572309496	n.s.
4690	5957979	59	42	2.56921572309496	n.s.
4690	5957991	59	42	2.56921572309496	n.s.
4690	5958003	59	42	2.56921572309496	n.s.
4690	5958008	58	43	1.97082887496121	n.s.
4690	5958012	58	43	1.97082887496121	n.s.
4690	5958016	57	44	1.45165612496859	n.s.
4690	5958019	56	45	1.0116974731171	n.s.
4690	5958021	56	45	1.0116974731171	n.s.
4690	5958053	56	45	1.0116974731171	n.s.
4690	5958054	56	45	1.0116974731171	n.s.
4690	5958057	55	46	0.650952919406731	n.s.
4690	5958072	55	46	0.650952919406731	n.s.
4690	5958093	54	47	0.369422463837495	n.s.
4690	5958099	53	48	0.167106106409387	n.s.
4690	5958113	54	47	0.369422463837495	n.s.
4690	5958129	54	47	0.369422463837495	n.s.
4690	5958134	55	46	0.650952919406731	n.s.
4690	5958137	55	46	0.650952919406731	n.s.
4690	5958139	55	46	0.650952919406731	n.s.
4690	5958149	54	47	0.369422463837495	n.s.
4690	5958190	53	48	0.167106106409387	n.s.
4690	5958193	54	47	0.369422463837495	n.s.
4690	5958236	53	48	0.167106106409387	n.s.
4690	5958240	53	48	0.167106106409387	n.s.
4690	5958241	54	47	0.369422463837495	n.s.
4690	5958244	53	48	0.167106106409387	n.s.
4690	5958250	54	47	0.369422463837495	n.s.
4690	5958287	53	48	0.167106106409387	n.s.
4690	5958301	53	48	0.167106106409387	n.s.
4690	5958309	54	47	0.369422463837495	n.s.
4690	5958320	54	47	0.369422463837495	n.s.
4690	5958325	54	47	0.369422463837495	n.s.
4690	5958355	54	47	0.369422463837495	n.s.
4690	5958357	54	47	0.369422463837495	n.s.
4690	5958370	55	46	0.650952919406731	n.s.
4690	5958375	55	46	0.650952919406731	n.s.
4690	5958388	54	47	0.369422463837495	n.s.

4690	5958402	54	47	0.369422463837495	n.s.
4690	5958418	54	47	0.369422463837495	n.s.
4690	5958427	53	48	0.167106106409387	n.s.
4690	5958442	52	49	0.044003847122408	n.s.
4690	5958449	51	50	0.000115685976557532	n.s.
4690	5958462	51	50	0.000115685976557532	n.s.
4690	5958463	50	51	0.0354416229718356	n.s.
4690	5958487	50	51	0.0354416229718356	n.s.
4690	5958489	50	51	0.0354416229718356	n.s.
4690	5958490	49	52	0.149981658108242	n.s.
4690	5958496	49	52	0.149981658108242	n.s.
4690	5958499	48	53	0.343735791385777	n.s.
4690	5958500	47	54	0.616704022804441	n.s.
4690	5958511	46	55	0.968886352364233	n.s.
4690	5958542	46	55	0.968886352364233	n.s.
4690	5958543	46	55	0.968886352364233	n.s.
4690	5958557	46	55	0.968886352364233	n.s.
4690	5958559	45	56	1.40028278006515	n.s.
4690	5958562	45	56	1.40028278006515	n.s.
4690	5958589	44	57	1.9108933059072	n.s.
4690	5958601	43	58	2.50071792989038	n.s.
4690	5958613	42	59	3.16975665201469	n.s.
4690	5958630	41	60	3.91800947228012	*
4690	5958634	41	60	3.91800947228012	*
4690	5958648	41	60	3.91800947228012	*
4690	5958659	41	60	3.91800947228012	*
4690	5958670	40	61	4.74547639068669	*
4690	5958695	39	62	5.65215740723438	*
4690	5958699	39	62	5.65215740723438	*
4690	5958721	38	63	6.6380525219232	*
4690	5958736	39	62	5.65215740723438	*
4690	5958742	38	63	6.6380525219232	*
4690	5958748	38	63	6.6380525219232	*
4690	5958769	38	63	6.6380525219232	*
4690	5958773	38	63	6.6380525219232	*
4690	5958785	38	63	6.6380525219232	*
4690	5958803	38	63	6.6380525219232	*
4690	5958811	37	64	7.70316173475315	**
4690	5958820	37	64	7.70316173475315	**
4690	5958821	37	64	7.70316173475315	**
4690	5958855	38	63	6.6380525219232	*
4690	5958870	39	62	5.65215740723438	*
4690	5958872	39	62	5.65215740723438	*
4690	5958878	40	61	4.74547639068669	*
4690	5958889	40	61	4.74547639068669	*
4690	5958894	41	60	3.91800947228012	*
4690	5958897	42	59	3.16975665201469	n.s.
4690	5958904	42	59	3.16975665201469	n.s.
4690	5958927	41	60	3.91800947228012	*
4690	5958945	42	59	3.16975665201469	n.s.

4690	5958957	42	59	3.16975665201469	n.s.
4690	5958960	42	59	3.16975665201469	n.s.
4690	5958991	43	58	2.50071792989038	n.s.
4690	5958997	43	58	2.50071792989038	n.s.
4690	5959003	44	57	1.9108933059072	n.s.
4690	5959006	45	56	1.40028278006515	n.s.
4690	5959007	45	56	1.40028278006515	n.s.
4690	5959010	45	56	1.40028278006515	n.s.
4690	5959025	46	55	0.968886352364233	n.s.
4690	5959046	46	55	0.968886352364233	n.s.
4690	5959049	46	55	0.968886352364233	n.s.
4690	5959050	45	56	1.40028278006515	n.s.
4690	5959064	45	56	1.40028278006515	n.s.
4690	5959083	45	56	1.40028278006515	n.s.
4690	5959091	45	56	1.40028278006515	n.s.
4690	5959112	44	57	1.9108933059072	n.s.
4690	5959113	43	58	2.50071792989038	n.s.
4690	5959118	44	57	1.9108933059072	n.s.
4690	5959148	44	57	1.9108933059072	n.s.
4690	5959163	44	57	1.9108933059072	n.s.
4690	5959181	45	56	1.40028278006515	n.s.
4690	5959185	45	56	1.40028278006515	n.s.
4690	5959189	45	56	1.40028278006515	n.s.
4690	5959195	45	56	1.40028278006515	n.s.
4690	5959199	46	55	0.968886352364233	n.s.
4690	5959209	45	56	1.40028278006515	n.s.
4690	5959224	46	55	0.968886352364233	n.s.
4690	5959229	46	55	0.968886352364233	n.s.
4690	5959234	45	56	1.40028278006515	n.s.
4690	5959241	44	57	1.9108933059072	n.s.
4690	5959248	43	58	2.50071792989038	n.s.
4690	5959253	42	59	3.16975665201469	n.s.
4690	5959273	43	58	2.50071792989038	n.s.
4690	5959297	44	57	1.9108933059072	n.s.
4690	5959311	45	56	1.40028278006515	n.s.
4690	5959324	44	57	1.9108933059072	n.s.
4690	5959325	44	57	1.9108933059072	n.s.
4690	5959342	44	57	1.9108933059072	n.s.
4690	5959345	44	57	1.9108933059072	n.s.
4690	5959346	44	57	1.9108933059072	n.s.
4690	5959377	44	57	1.9108933059072	n.s.
4690	5959381	45	56	1.40028278006515	n.s.
4690	5959386	45	56	1.40028278006515	n.s.
4690	5959391	45	56	1.40028278006515	n.s.
4690	5959402	45	56	1.40028278006515	n.s.
4690	5959405	45	56	1.40028278006515	n.s.
4690	5959406	44	57	1.9108933059072	n.s.
4690	5959407	44	57	1.9108933059072	n.s.
4690	5959410	43	58	2.50071792989038	n.s.
4690	5959416	43	58	2.50071792989038	n.s.

4690	5959424	44	57	1.9108933059072	n.s.
4690	5959431	43	58	2.50071792989038	n.s.
4690	5959432	43	58	2.50071792989038	n.s.
4690	5959433	43	58	2.50071792989038	n.s.
4690	5959440	43	58	2.50071792989038	n.s.
4690	5959457	43	58	2.50071792989038	n.s.
4690	5959459	43	58	2.50071792989038	n.s.
4690	5959465	44	57	1.9108933059072	n.s.
4690	5959470	44	57	1.9108933059072	n.s.
4690	5959476	45	56	1.40028278006515	n.s.
4690	5959479	45	56	1.40028278006515	n.s.
4690	5959495	46	55	0.968886352364233	n.s.
4690	5959496	47	54	0.616704022804441	n.s.
4690	5959501	48	53	0.343735791385777	n.s.
4690	5959506	49	52	0.149981658108242	n.s.
4690	5959579	49	52	0.149981658108242	n.s.
4690	5959587	49	52	0.149981658108242	n.s.
4690	5959612	49	52	0.149981658108242	n.s.
4690	5959628	49	52	0.149981658108242	n.s.
4690	5959630	49	52	0.149981658108242	n.s.
4690	5959634	49	52	0.149981658108242	n.s.
4690	5959645	50	51	0.0354416229718356	n.s.
4690	5959666	51	50	0.000115685976557532	n.s.
4690	5959681	52	49	0.044003847122408	n.s.
4690	5959690	52	49	0.044003847122408	n.s.
4690	5959708	52	49	0.044003847122408	n.s.
4690	5959716	52	49	0.044003847122408	n.s.
4690	5959730	53	48	0.167106106409387	n.s.
4690	5959735	54	47	0.369422463837495	n.s.
4690	5959746	55	46	0.650952919406731	n.s.
4690	5959747	56	45	1.0116974731171	n.s.
4690	5959778	55	46	0.650952919406731	n.s.
4690	5959806	55	46	0.650952919406731	n.s.
4690	5959821	55	46	0.650952919406731	n.s.
4690	5959832	55	46	0.650952919406731	n.s.
4690	5959840	55	46	0.650952919406731	n.s.
4690	5959841	55	46	0.650952919406731	n.s.
4690	5959859	54	47	0.369422463837495	n.s.
4690	5959861	55	46	0.650952919406731	n.s.
4690	5959876	54	47	0.369422463837495	n.s.
4690	5959888	53	48	0.167106106409387	n.s.
4690	5959897	53	48	0.167106106409387	n.s.
4690	5959922	53	48	0.167106106409387	n.s.
4690	5959928	52	49	0.044003847122408	n.s.
4690	5959936	52	49	0.044003847122408	n.s.
4690	5959975	53	48	0.167106106409387	n.s.
4690	5959980	53	48	0.167106106409387	n.s.
4690	5960003	53	48	0.167106106409387	n.s.
4690	5960005	53	48	0.167106106409387	n.s.
4690	5960012	53	48	0.167106106409387	n.s.

4690	5960014	53	48	0.167106106409387	n.s.
4690	5960017	52	49	0.044003847122408	n.s.
4690	5960033	52	49	0.044003847122408	n.s.
4690	5960037	52	49	0.044003847122408	n.s.
4690	5960044	52	49	0.044003847122408	n.s.
4690	5960046	52	49	0.044003847122408	n.s.
4690	5960065	51	50	0.000115685976557532	n.s.
4690	5960093	50	51	0.0354416229718356	n.s.
4690	5960095	50	51	0.0354416229718356	n.s.
4690	5960130	49	52	0.149981658108242	n.s.
4690	5960141	48	53	0.343735791385777	n.s.
4690	5960165	48	53	0.343735791385777	n.s.
4690	5960172	48	53	0.343735791385777	n.s.
4690	5960178	49	52	0.149981658108242	n.s.
4690	5960179	49	52	0.149981658108242	n.s.
4690	5960182	49	52	0.149981658108242	n.s.
4690	5960184	50	51	0.0354416229718356	n.s.
4690	5960188	50	51	0.0354416229718356	n.s.
4690	5960221	50	51	0.0354416229718356	n.s.
4690	5960228	50	51	0.0354416229718356	n.s.
4690	5960233	50	51	0.0354416229718356	n.s.
4690	5960237	49	52	0.149981658108242	n.s.
4690	5960242	50	51	0.0354416229718356	n.s.
4690	5960246	51	50	0.000115685976557532	n.s.
4690	5960254	50	51	0.0354416229718356	n.s.
4690	5960257	49	52	0.149981658108242	n.s.
4690	5960277	49	52	0.149981658108242	n.s.
4690	5960288	48	53	0.343735791385777	n.s.
4690	5960289	48	53	0.343735791385777	n.s.
4690	5960329	48	53	0.343735791385777	n.s.
4690	5960331	49	52	0.149981658108242	n.s.
4690	5960332	49	52	0.149981658108242	n.s.
4690	5960356	50	51	0.0354416229718356	n.s.
4690	5960357	49	52	0.149981658108242	n.s.
4690	5960362	49	52	0.149981658108242	n.s.
4690	5960372	48	53	0.343735791385777	n.s.
4690	5960377	48	53	0.343735791385777	n.s.
4690	5960390	48	53	0.343735791385777	n.s.
4690	5960417	48	53	0.343735791385777	n.s.
4690	5960450	47	54	0.616704022804441	n.s.
4690	5960469	48	53	0.343735791385777	n.s.
4690	5960471	47	54	0.616704022804441	n.s.
4690	5960487	46	55	0.968886352364233	n.s.
4690	5960498	45	56	1.40028278006515	n.s.
4690	5960511	45	56	1.40028278006515	n.s.
4690	5960530	44	57	1.9108933059072	n.s.
4690	5960559	45	56	1.40028278006515	n.s.
4690	5960569	46	55	0.968886352364233	n.s.
4690	5960578	46	55	0.968886352364233	n.s.
4690	5960584	46	55	0.968886352364233	n.s.

4690	5960595	46	55	0.968886352364233	n.s.
4690	5960608	45	56	1.40028278006515	n.s.
4690	5960610	46	55	0.968886352364233	n.s.
4690	5960641	45	56	1.40028278006515	n.s.
4690	5960642	45	56	1.40028278006515	n.s.
4690	5960643	45	56	1.40028278006515	n.s.
4690	5960645	45	56	1.40028278006515	n.s.
4690	5960646	45	56	1.40028278006515	n.s.
4690	5960651	44	57	1.9108933059072	n.s.
4690	5960657	44	57	1.9108933059072	n.s.
4690	5960658	43	58	2.50071792989038	n.s.
4690	5960679	43	58	2.50071792989038	n.s.
4690	5960684	43	58	2.50071792989038	n.s.
4690	5960687	43	58	2.50071792989038	n.s.
4690	5960696	43	58	2.50071792989038	n.s.
4690	5960711	43	58	2.50071792989038	n.s.
4690	5960713	42	59	3.16975665201469	n.s.
4690	5960723	42	59	3.16975665201469	n.s.
4690	5960725	41	60	3.91800947228012	*
4690	5960750	41	60	3.91800947228012	*
4690	5960760	42	59	3.16975665201469	n.s.
4690	5960770	42	59	3.16975665201469	n.s.
4690	5960778	41	60	3.91800947228012	*
4690	5960794	41	60	3.91800947228012	*
4690	5960813	40	61	4.74547639068669	*
4690	5960825	41	60	3.91800947228012	*
4690	5960834	42	59	3.16975665201469	n.s.
4690	5960858	42	59	3.16975665201469	n.s.
4690	5960864	41	60	3.91800947228012	*
4690	5960870	40	61	4.74547639068669	*
4690	5960890	40	61	4.74547639068669	*
4690	5960904	39	62	5.65215740723438	*
4690	5960924	40	61	4.74547639068669	*
4690	5960932	40	61	4.74547639068669	*
4690	5960953	40	61	4.74547639068669	*
4690	5960957	41	60	3.91800947228012	*
4690	5960958	42	59	3.16975665201469	n.s.
4690	5960982	42	59	3.16975665201469	n.s.
4690	5960989	43	58	2.50071792989038	n.s.
4690	5961003	42	59	3.16975665201469	n.s.
4690	5961005	43	58	2.50071792989038	n.s.
4690	5961012	43	58	2.50071792989038	n.s.
4690	5961013	42	59	3.16975665201469	n.s.
4690	5961028	41	60	3.91800947228012	*
4690	5961032	41	60	3.91800947228012	*
4690	5961034	41	60	3.91800947228012	*
4690	5961043	40	61	4.74547639068669	*
4690	5961045	39	62	5.65215740723438	*
4690	5961055	39	62	5.65215740723438	*
4690	5961062	38	63	6.6380525219232	*

4690	5961068	38	63	6.6380525219232	*
4690	5961070	38	63	6.6380525219232	*
4690	5961088	39	62	5.65215740723438	*
4690	5961089	39	62	5.65215740723438	*
4690	5961099	39	62	5.65215740723438	*
4690	5961123	39	62	5.65215740723438	*
4690	5961131	39	62	5.65215740723438	*
4690	5961133	40	61	4.74547639068669	*
4690	5961149	40	61	4.74547639068669	*
4690	5961150	40	61	4.74547639068669	*
4690	5961157	41	60	3.91800947228012	*
4690	5961159	42	59	3.16975665201469	n.s.
4690	5961162	42	59	3.16975665201469	n.s.
4690	5961166	43	58	2.50071792989038	n.s.
4690	5961167	43	58	2.50071792989038	n.s.
4690	5961185	44	57	1.9108933059072	n.s.
4690	5961187	44	57	1.9108933059072	n.s.
4690	5961219	44	57	1.9108933059072	n.s.
4690	5961235	45	56	1.40028278006515	n.s.
4690	5961257	45	56	1.40028278006515	n.s.
4690	5961265	46	55	0.968886352364233	n.s.
4690	5961268	46	55	0.968886352364233	n.s.
4690	5961269	46	55	0.968886352364233	n.s.
4690	5961277	45	56	1.40028278006515	n.s.
4690	5961280	45	56	1.40028278006515	n.s.
4690	5961295	45	56	1.40028278006515	n.s.
4690	5961297	45	56	1.40028278006515	n.s.
4690	5961310	46	55	0.968886352364233	n.s.
4690	5961318	47	54	0.616704022804441	n.s.
4690	5961343	47	54	0.616704022804441	n.s.
4690	5961344	48	53	0.343735791385777	n.s.
4690	5961345	47	54	0.616704022804441	n.s.
4690	5961349	48	53	0.343735791385777	n.s.
4690	5961375	47	54	0.616704022804441	n.s.
4690	5961386	48	53	0.343735791385777	n.s.
4690	5961401	47	54	0.616704022804441	n.s.
4690	5961407	47	54	0.616704022804441	n.s.
4690	5961415	48	53	0.343735791385777	n.s.
4690	5961437	48	53	0.343735791385777	n.s.
4690	5961449	48	53	0.343735791385777	n.s.
4690	5961456	48	53	0.343735791385777	n.s.
4690	5961457	48	53	0.343735791385777	n.s.
4690	5961463	48	53	0.343735791385777	n.s.
4690	5961480	48	53	0.343735791385777	n.s.
4690	5961483	48	53	0.343735791385777	n.s.
4690	5961486	47	54	0.616704022804441	n.s.
4690	5961488	47	54	0.616704022804441	n.s.
4690	5961496	46	55	0.968886352364233	n.s.
4690	5961515	46	55	0.968886352364233	n.s.
4690	5961521	46	55	0.968886352364233	n.s.

4690	5961523	46	55	0.968886352364233	n.s.
4690	5961539	46	55	0.968886352364233	n.s.
4690	5961542	46	55	0.968886352364233	n.s.
4690	5961546	46	55	0.968886352364233	n.s.
4690	5961547	47	54	0.616704022804441	n.s.
4690	5961553	47	54	0.616704022804441	n.s.
4690	5961560	47	54	0.616704022804441	n.s.
4690	5961561	47	54	0.616704022804441	n.s.
4690	5961563	47	54	0.616704022804441	n.s.
4690	5961568	47	54	0.616704022804441	n.s.
4690	5961569	47	54	0.616704022804441	n.s.
4690	5961581	47	54	0.616704022804441	n.s.
4690	5961596	47	54	0.616704022804441	n.s.
4690	5961605	46	55	0.968886352364233	n.s.
4690	5961609	45	56	1.40028278006515	n.s.
4690	5961620	44	57	1.9108933059072	n.s.
4690	5961630	43	58	2.50071792989038	n.s.
4690	5961635	43	58	2.50071792989038	n.s.
4690	5961657	44	57	1.9108933059072	n.s.
4690	5961664	45	56	1.40028278006515	n.s.
4690	5961681	46	55	0.968886352364233	n.s.
4690	5961682	45	56	1.40028278006515	n.s.
4690	5961684	46	55	0.968886352364233	n.s.
4690	5961686	47	54	0.616704022804441	n.s.
4690	5961694	47	54	0.616704022804441	n.s.
4690	5961699	48	53	0.343735791385777	n.s.
4690	5961704	48	53	0.343735791385777	n.s.
4690	5961710	47	54	0.616704022804441	n.s.
4690	5961711	48	53	0.343735791385777	n.s.
4690	5961732	49	52	0.149981658108242	n.s.
4690	5961744	49	52	0.149981658108242	n.s.
4690	5961757	49	52	0.149981658108242	n.s.
4690	5961762	50	51	0.0354416229718356	n.s.
4690	5961765	50	51	0.0354416229718356	n.s.
4690	5961766	50	51	0.0354416229718356	n.s.
4690	5961774	50	51	0.0354416229718356	n.s.
4690	5961776	50	51	0.0354416229718356	n.s.
4690	5961802	49	52	0.149981658108242	n.s.
4690	5961811	49	52	0.149981658108242	n.s.
4690	5961812	48	53	0.343735791385777	n.s.
4690	5961821	49	52	0.149981658108242	n.s.
4690	5961834	48	53	0.343735791385777	n.s.
4690	5961848	49	52	0.149981658108242	n.s.
4690	5961850	49	52	0.149981658108242	n.s.
4690	5961856	49	52	0.149981658108242	n.s.
4690	5961858	49	52	0.149981658108242	n.s.
4690	5961861	48	53	0.343735791385777	n.s.
4690	5961867	48	53	0.343735791385777	n.s.
4690	5961878	48	53	0.343735791385777	n.s.
4690	5961883	48	53	0.343735791385777	n.s.

4690	5961889	48	53	0.343735791385777	n.s.
4690	5961898	49	52	0.149981658108242	n.s.
4690	5961915	48	53	0.343735791385777	n.s.
4690	5961931	48	53	0.343735791385777	n.s.
4690	5961933	48	53	0.343735791385777	n.s.
4690	5961936	47	54	0.616704022804441	n.s.
4690	5961941	47	54	0.616704022804441	n.s.
4690	5961956	46	55	0.968886352364233	n.s.
4690	5961987	46	55	0.968886352364233	n.s.
4690	5962016	47	54	0.616704022804441	n.s.
4690	5962019	46	55	0.968886352364233	n.s.
4690	5962079	46	55	0.968886352364233	n.s.
4690	5962098	46	55	0.968886352364233	n.s.
4690	5962122	46	55	0.968886352364233	n.s.
4690	5962123	46	55	0.968886352364233	n.s.
4690	5962135	46	55	0.968886352364233	n.s.
4690	5962139	46	55	0.968886352364233	n.s.
4690	5962142	47	54	0.616704022804441	n.s.
4690	5962147	46	55	0.968886352364233	n.s.
4690	5962148	46	55	0.968886352364233	n.s.
4690	5962151	46	55	0.968886352364233	n.s.
4690	5962154	45	56	1.40028278006515	n.s.
4690	5962155	45	56	1.40028278006515	n.s.
4690	5962157	46	55	0.968886352364233	n.s.
4690	5962159	47	54	0.616704022804441	n.s.
4690	5962164	46	55	0.968886352364233	n.s.
4690	5962169	47	54	0.616704022804441	n.s.
4690	5962171	47	54	0.616704022804441	n.s.
4690	5962174	46	55	0.968886352364233	n.s.
4690	5962190	45	56	1.40028278006515	n.s.
4690	5962194	46	55	0.968886352364233	n.s.
4690	5962195	45	56	1.40028278006515	n.s.
4690	5962200	45	56	1.40028278006515	n.s.
4690	5962203	45	56	1.40028278006515	n.s.
4690	5962206	45	56	1.40028278006515	n.s.
4690	5962235	45	56	1.40028278006515	n.s.
4690	5962263	45	56	1.40028278006515	n.s.
4690	5962266	45	56	1.40028278006515	n.s.
4690	5962270	44	57	1.9108933059072	n.s.
4690	5962281	44	57	1.9108933059072	n.s.
4690	5962283	44	57	1.9108933059072	n.s.
4690	5962289	45	56	1.40028278006515	n.s.
4690	5962293	45	56	1.40028278006515	n.s.
4690	5962306	45	56	1.40028278006515	n.s.
4690	5962312	45	56	1.40028278006515	n.s.
4690	5962314	45	56	1.40028278006515	n.s.
4690	5962317	46	55	0.968886352364233	n.s.
4690	5962319	46	55	0.968886352364233	n.s.
4690	5962336	46	55	0.968886352364233	n.s.
4690	5962340	46	55	0.968886352364233	n.s.

4690	5962346	46	55	0.968886352364233	n.s.
4690	5962350	46	55	0.968886352364233	n.s.
4690	5962355	46	55	0.968886352364233	n.s.
4690	5962366	46	55	0.968886352364233	n.s.
4690	5962385	45	56	1.40028278006515	n.s.
4690	5962387	44	57	1.9108933059072	n.s.
4690	5962391	44	57	1.9108933059072	n.s.
4690	5962401	44	57	1.9108933059072	n.s.
4690	5962413	44	57	1.9108933059072	n.s.
4690	5962491	43	58	2.50071792989038	n.s.
4690	5962518	43	58	2.50071792989038	n.s.
4690	5962530	42	59	3.16975665201469	n.s.
4690	5962550	42	59	3.16975665201469	n.s.
4690	5962558	42	59	3.16975665201469	n.s.
4690	5962569	42	59	3.16975665201469	n.s.
4690	5962599	43	58	2.50071792989038	n.s.
4690	5962630	43	58	2.50071792989038	n.s.
4690	5962635	43	58	2.50071792989038	n.s.
4690	5962639	43	58	2.50071792989038	n.s.
4690	5962648	42	59	3.16975665201469	n.s.
4690	5962676	41	60	3.91800947228012	*
4690	5962700	40	61	4.74547639068669	*
4690	5962705	41	60	3.91800947228012	*
4690	5962706	41	60	3.91800947228012	*
4690	5962710	40	61	4.74547639068669	*
4690	5962713	40	61	4.74547639068669	*
4690	5962747	40	61	4.74547639068669	*
4690	5962756	40	61	4.74547639068669	*
4690	5962761	41	60	3.91800947228012	*
4690	5962791	41	60	3.91800947228012	*
4690	5962797	41	60	3.91800947228012	*
4690	5962799	42	59	3.16975665201469	n.s.
4690	5962804	42	59	3.16975665201469	n.s.
4690	5962812	41	60	3.91800947228012	*
4690	5962814	41	60	3.91800947228012	*
4690	5962824	41	60	3.91800947228012	*
4690	5962826	41	60	3.91800947228012	*
4690	5962841	41	60	3.91800947228012	*
4690	5962874	41	60	3.91800947228012	*
4690	5962917	42	59	3.16975665201469	n.s.
4690	5962926	43	58	2.50071792989038	n.s.
4690	5962979	42	59	3.16975665201469	n.s.
4690	5962987	42	59	3.16975665201469	n.s.
4690	5963006	42	59	3.16975665201469	n.s.
4690	5963010	43	58	2.50071792989038	n.s.
4690	5963011	44	57	1.9108933059072	n.s.
4690	5963012	45	56	1.40028278006515	n.s.
4690	5963037	46	55	0.968886352364233	n.s.
4690	5963045	47	54	0.616704022804441	n.s.
4690	5963049	48	53	0.343735791385777	n.s.

4690	5963061	48	53	0.343735791385777	n.s.
4690	5963077	49	52	0.149981658108242	n.s.
4690	5963096	49	52	0.149981658108242	n.s.
4690	5963097	50	51	0.0354416229718356	n.s.
4690	5963129	50	51	0.0354416229718356	n.s.
4690	5963143	50	51	0.0354416229718356	n.s.
4690	5963172	51	50	0.000115685976557532	n.s.
4690	5963177	51	50	0.000115685976557532	n.s.
4690	5963237	51	50	0.000115685976557532	n.s.
4690	5963253	51	50	0.000115685976557532	n.s.
4690	5963306	51	50	0.000115685976557532	n.s.
4690	5963314	51	50	0.000115685976557532	n.s.
4690	5963353	50	51	0.0354416229718356	n.s.
4690	5963358	50	51	0.0354416229718356	n.s.
4690	5963406	50	51	0.0354416229718356	n.s.
4690	5963408	49	52	0.149981658108242	n.s.
4690	5963417	48	53	0.343735791385777	n.s.
4690	5963447	47	54	0.616704022804441	n.s.
4690	5963481	46	55	0.968886352364233	n.s.
4690	5963531	46	55	0.968886352364233	n.s.
4690	5963532	46	55	0.968886352364233	n.s.
4690	5963597	45	56	1.40028278006515	n.s.
4690	5963605	46	55	0.968886352364233	n.s.
4690	5963608	47	54	0.616704022804441	n.s.
4690	5963609	47	54	0.616704022804441	n.s.
4690	5963612	46	55	0.968886352364233	n.s.
4690	5963635	47	54	0.616704022804441	n.s.
4690	5963657	47	54	0.616704022804441	n.s.
4690	5963680	48	53	0.343735791385777	n.s.
4690	5963689	49	52	0.149981658108242	n.s.
4690	5963716	50	51	0.0354416229718356	n.s.
4690	5963743	50	51	0.0354416229718356	n.s.
4690	5963759	51	50	0.000115685976557532	n.s.
4690	5963768	52	49	0.044003847122408	n.s.
4690	5963785	52	49	0.044003847122408	n.s.
4690	5963798	53	48	0.167106106409387	n.s.
4690	5963879	53	48	0.167106106409387	n.s.
4690	5963881	54	47	0.369422463837495	n.s.
4690	5963885	54	47	0.369422463837495	n.s.
4690	5963894	55	46	0.650952919406731	n.s.
4690	5963939	55	46	0.650952919406731	n.s.
4690	5963948	55	46	0.650952919406731	n.s.
4690	5963959	55	46	0.650952919406731	n.s.
4690	5963960	55	46	0.650952919406731	n.s.
4690	5963966	55	46	0.650952919406731	n.s.
4690	5963969	56	45	1.0116974731171	n.s.
4690	5963972	57	44	1.45165612496859	n.s.
4690	5963975	57	44	1.45165612496859	n.s.
4690	5963978	58	43	1.97082887496121	n.s.
4690	5963987	59	42	2.56921572309496	n.s.

4690	5964004	59	42	2.56921572309496	n.s.
4690	5964008	60	41	3.24681666936984	n.s.
4690	5964036	60	41	3.24681666936984	n.s.
4690	5964039	61	40	4.00363171378585	*
4690	5964041	62	39	4.83966085634298	*
4690	5964047	62	39	4.83966085634298	*
4690	5964050	63	38	5.75490409704125	*
4690	5964059	64	37	6.74936143588064	**
4690	5964077	64	37	6.74936143588064	**
4690	5964085	64	37	6.74936143588064	**
4690	5964130	65	36	7.82303287286116	**
4690	5964134	65	36	7.82303287286116	**
4690	5964137	66	35	8.97591840798281	**
4690	5964140	67	34	10.2080180412456	**
4690	5964143	68	33	11.5193317726495	***
4690	5964146	69	32	12.9098596021945	***
4690	5964154	69	32	12.9098596021945	***
4690	5964167	69	32	12.9098596021945	***
4690	5964170	70	31	14.3796015298807	***
4690	5964173	71	30	15.928557555708	***
4690	5964182	71	30	15.928557555708	***
4690	5964194	71	30	15.928557555708	***
4690	5964197	72	29	17.5567276796764	***
4690	5964199	72	29	17.5567276796764	***
4690	5964212	72	29	17.5567276796764	***
4690	5964221	73	28	19.264111901786	***
4690	5964227	73	28	19.264111901786	***
4690	5964230	73	28	19.264111901786	***
4690	5964232	73	28	19.264111901786	***
4690	5964235	73	28	19.264111901786	***
4690	5964236	73	28	19.264111901786	***
4690	5964251	73	28	19.264111901786	***
4690	5964254	73	28	19.264111901786	***
4690	5964285	73	28	19.264111901786	***
4690	5964291	74	27	21.0507102220366	***
4690	5964293	74	27	21.0507102220366	***
4690	5964323	75	26	22.9165226404284	***
4690	5964324	76	25	24.8615491569614	***
4690	5964335	76	25	24.8615491569614	***
4690	5964359	77	24	26.8857897716355	***
4690	5964362	77	24	26.8857897716355	***
4690	5964366	77	24	26.8857897716355	***
4690	5964374	78	23	28.9892444844506	***
4690	5964377	79	22	31.171913295407	***
4690	5964386	79	22	31.171913295407	***
4690	5964389	79	22	31.171913295407	***
4690	5964394	79	22	31.171913295407	***
4690	5964408	79	22	31.171913295407	***
4690	5964409	79	22	31.171913295407	***
4690	5964431	79	22	31.171913295407	***

4690	5964433	78	23	28.9892444844506	***
4690	5964439	78	23	28.9892444844506	***
4690	5964449	78	23	28.9892444844506	***
4690	5964461	78	23	28.9892444844506	***
4690	5964494	78	23	28.9892444844506	***
4690	5964542	78	23	28.9892444844506	***
4690	5964548	77	24	26.8857897716355	***
4690	5964581	76	25	24.8615491569614	***
4690	5964597	76	25	24.8615491569614	***
4690	5964617	76	25	24.8615491569614	***
4690	5964630	76	25	24.8615491569614	***
4690	5964635	75	26	22.9165226404284	***
4690	5964636	76	25	24.8615491569614	***
4690	5964643	76	25	24.8615491569614	***
4690	5964692	76	25	24.8615491569614	***
4690	5964706	75	26	22.9165226404284	***
4690	5964772	76	25	24.8615491569614	***
4690	5964802	76	25	24.8615491569614	***
4690	5964833	76	25	24.8615491569614	***
4690	5964834	77	24	26.8857897716355	***
4690	5964842	78	23	28.9892444844506	***
4690	5964854	78	23	28.9892444844506	***
4690	5964885	79	22	31.171913295407	***
4690	5964902	79	22	31.171913295407	***
4690	5964913	79	22	31.171913295407	***
4690	5964923	79	22	31.171913295407	***
4690	5964940	80	21	33.4337962045044	***
4690	5964941	80	21	33.4337962045044	***
4690	5964943	80	21	33.4337962045044	***
4690	5964944	80	21	33.4337962045044	***
4690	5964949	80	21	33.4337962045044	***
4690	5964955	80	21	33.4337962045044	***
4690	5964961	80	21	33.4337962045044	***
4690	5964962	80	21	33.4337962045044	***
4690	5964968	79	22	31.171913295407	***
4690	5964971	79	22	31.171913295407	***
4690	5964981	78	23	28.9892444844506	***
4690	5965007	77	24	26.8857897716355	***
4690	5965008	77	24	26.8857897716355	***
4690	5965031	77	24	26.8857897716355	***
4690	5965046	76	25	24.8615491569614	***
4690	5965051	76	25	24.8615491569614	***
4690	5965055	76	25	24.8615491569614	***
4690	5965070	76	25	24.8615491569614	***
4690	5965073	75	26	22.9165226404284	***
4690	5965079	76	25	24.8615491569614	***
4690	5965082	76	25	24.8615491569614	***
4690	5965085	75	26	22.9165226404284	***
4690	5965088	74	27	21.0507102220366	***
4690	5965094	74	27	21.0507102220366	***

4690	5965100	73	28	19.264111901786	***
4690	5965106	73	28	19.264111901786	***
4690	5965118	73	28	19.264111901786	***
4690	5965124	73	28	19.264111901786	***
4690	5965127	72	29	17.5567276796764	***
4690	5965137	71	30	15.928557555708	***
4690	5965147	72	29	17.5567276796764	***
4690	5965163	71	30	15.928557555708	***
4690	5965164	71	30	15.928557555708	***
4690	5965169	72	29	17.5567276796764	***
4690	5965172	73	28	19.264111901786	***
4690	5965192	72	29	17.5567276796764	***
4690	5965193	73	28	19.264111901786	***
4690	5965211	72	29	17.5567276796764	***
4690	5965212	71	30	15.928557555708	***
4690	5965213	70	31	14.3796015298807	***
4690	5965218	69	32	12.9098596021945	***
4690	5965221	69	32	12.9098596021945	***
4690	5965234	69	32	12.9098596021945	***
4690	5965237	68	33	11.5193317726495	***
4690	5965244	67	34	10.2080180412456	**
4690	5965249	67	34	10.2080180412456	**
4690	5965258	67	34	10.2080180412456	**
4690	5965261	67	34	10.2080180412456	**
4690	5965264	67	34	10.2080180412456	**
4690	5965273	66	35	8.97591840798281	**
4690	5965276	65	36	7.82303287286116	**
4690	5965280	65	36	7.82303287286116	**
4690	5965288	64	37	6.74936143588064	**
4690	5965289	64	37	6.74936143588064	**
4690	5965303	64	37	6.74936143588064	**
4690	5965308	63	38	5.75490409704125	*
4690	5965311	63	38	5.75490409704125	*
4690	5965357	62	39	4.83966085634298	*
4690	5965399	61	40	4.00363171378585	*
4690	5965414	61	40	4.00363171378585	*
4690	5965427	61	40	4.00363171378585	*
4690	5965434	60	41	3.24681666936984	n.s.
4690	5965437	60	41	3.24681666936984	n.s.
4690	5965441	59	42	2.56921572309496	n.s.
4690	5965455	58	43	1.97082887496121	n.s.
4690	5965470	58	43	1.97082887496121	n.s.
4690	5965500	57	44	1.45165612496859	n.s.
4690	5965509	57	44	1.45165612496859	n.s.
4690	5965528	57	44	1.45165612496859	n.s.
4690	5966011	57	44	1.45165612496859	n.s.
4690	5966074	57	44	1.45165612496859	n.s.
4690	5966114	57	44	1.45165612496859	n.s.
4690	5966154	57	44	1.45165612496859	n.s.
4690	5966180	57	44	1.45165612496859	n.s.

4690	5966194	57	44	1.45165612496859	n.s.
4690	5966227	58	43	1.97082887496121	n.s.
4690	5966228	58	43	1.97082887496121	n.s.
4690	5966239	58	43	1.97082887496121	n.s.
4690	5966250	58	43	1.97082887496121	n.s.
4690	5966256	58	43	1.97082887496121	n.s.
4690	5966304	58	43	1.97082887496121	n.s.
4690	5966364	58	43	1.97082887496121	n.s.
4690	5966367	59	42	2.56921572309496	n.s.
4690	5966371	58	43	1.97082887496121	n.s.
4690	5966373	58	43	1.97082887496121	n.s.
4690	5966394	58	43	1.97082887496121	n.s.
4690	5966423	58	43	1.97082887496121	n.s.
4690	5966425	57	44	1.45165612496859	n.s.
4690	5966438	57	44	1.45165612496859	n.s.
4690	5966477	56	45	1.0116974731171	n.s.
4690	5966480	56	45	1.0116974731171	n.s.
4690	5966495	55	46	0.650952919406731	n.s.
4690	5966497	56	45	1.0116974731171	n.s.
4690	5966499	56	45	1.0116974731171	n.s.
4690	5966502	56	45	1.0116974731171	n.s.
4690	5966530	55	46	0.650952919406731	n.s.
4690	5966550	55	46	0.650952919406731	n.s.
4690	5966576	54	47	0.369422463837495	n.s.
4690	5966586	54	47	0.369422463837495	n.s.
4690	5966592	53	48	0.167106106409387	n.s.
4690	5966595	53	48	0.167106106409387	n.s.
4690	5966603	52	49	0.044003847122408	n.s.
4690	5966609	51	50	0.000115685976557532	n.s.
4690	5966612	50	51	0.0354416229718356	n.s.
4690	5966613	50	51	0.0354416229718356	n.s.
4690	5966624	49	52	0.149981658108242	n.s.
4690	5966627	49	52	0.149981658108242	n.s.
4690	5966630	49	52	0.149981658108242	n.s.
4690	5966634	49	52	0.149981658108242	n.s.
4690	5966635	50	51	0.0354416229718356	n.s.
4690	5966640	49	52	0.149981658108242	n.s.
4690	5966645	50	51	0.0354416229718356	n.s.
4690	5966659	51	50	0.000115685976557532	n.s.
4690	5966664	51	50	0.000115685976557532	n.s.
4690	5966666	52	49	0.044003847122408	n.s.
4690	5966669	53	48	0.167106106409387	n.s.
4690	5966681	54	47	0.369422463837495	n.s.
4690	5966693	55	46	0.650952919406731	n.s.
4690	5966704	55	46	0.650952919406731	n.s.
4690	5966721	55	46	0.650952919406731	n.s.
4690	5966722	55	46	0.650952919406731	n.s.
4690	5966729	55	46	0.650952919406731	n.s.
4690	5966730	56	45	1.0116974731171	n.s.
4690	5966738	56	45	1.0116974731171	n.s.

4690	5966740	56	45	1.0116974731171	n.s.
4690	5966789	56	45	1.0116974731171	n.s.
4690	5966798	56	45	1.0116974731171	n.s.
4690	5966807	56	45	1.0116974731171	n.s.
4690	5966826	57	44	1.45165612496859	n.s.
4690	5966870	58	43	1.97082887496121	n.s.
4690	5966886	58	43	1.97082887496121	n.s.
4690	5966899	57	44	1.45165612496859	n.s.
4690	5966920	58	43	1.97082887496121	n.s.
4690	5966921	58	43	1.97082887496121	n.s.
4690	5966940	57	44	1.45165612496859	n.s.
4690	5966944	56	45	1.0116974731171	n.s.
4690	5966955	56	45	1.0116974731171	n.s.
4690	5966961	56	45	1.0116974731171	n.s.
4690	5966970	56	45	1.0116974731171	n.s.
4690	5966975	56	45	1.0116974731171	n.s.
4690	5966985	56	45	1.0116974731171	n.s.
4690	5966997	57	44	1.45165612496859	n.s.
4690	5966999	57	44	1.45165612496859	n.s.
4690	5967013	58	43	1.97082887496121	n.s.
4690	5967088	59	42	2.56921572309496	n.s.
4690	5967096	60	41	3.24681666936984	n.s.
4690	5967109	60	41	3.24681666936984	n.s.
4690	5967113	61	40	4.00363171378585	*
4690	5967129	61	40	4.00363171378585	*
4690	5967134	61	40	4.00363171378585	*
4690	5967150	62	39	4.83966085634298	*
4690	5967153	63	38	5.75490409704125	*
4690	5967178	63	38	5.75490409704125	*
4690	5967212	64	37	6.74936143588064	**
4690	5967273	64	37	6.74936143588064	**
4690	5967278	63	38	5.75490409704125	*
4690	5967296	63	38	5.75490409704125	*
4690	5967311	62	39	4.83966085634298	*
4690	5967332	62	39	4.83966085634298	*
4690	5967336	62	39	4.83966085634298	*
4690	5967347	62	39	4.83966085634298	*
4690	5967385	62	39	4.83966085634298	*
4690	5967397	63	38	5.75490409704125	*
4690	5967401	63	38	5.75490409704125	*
4690	5967405	63	38	5.75490409704125	*
4690	5967418	64	37	6.74936143588064	**
4690	5967440	64	37	6.74936143588064	**
4690	5967443	65	36	7.82303287286116	**
4690	5967457	64	37	6.74936143588064	**
4690	5967463	64	37	6.74936143588064	**
4690	5967476	63	38	5.75490409704125	*
4690	5967478	63	38	5.75490409704125	*
4690	5967479	63	38	5.75490409704125	*
4690	5967484	63	38	5.75490409704125	*

4690	5967490	63	38	5.75490409704125	*
4690	5967491	63	38	5.75490409704125	*
4690	5967508	63	38	5.75490409704125	*
4690	5967518	62	39	4.83966085634298	*
4690	5967536	62	39	4.83966085634298	*
4690	5967551	62	39	4.83966085634298	*
4690	5967571	61	40	4.00363171378585	*
4690	5967636	60	41	3.24681666936984	n.s.
4690	5967638	61	40	4.00363171378585	*
4690	5967639	61	40	4.00363171378585	*
4690	5967640	62	39	4.83966085634298	*
4690	5967642	63	38	5.75490409704125	*
4690	5967653	63	38	5.75490409704125	*
4690	5967659	63	38	5.75490409704125	*
4690	5967665	63	38	5.75490409704125	*
4690	5967666	64	37	6.74936143588064	**
4690	5967680	65	36	7.82303287286116	**
4690	5967689	66	35	8.97591840798281	**
4690	5967714	66	35	8.97591840798281	**
4690	5967719	66	35	8.97591840798281	**
4690	5967732	67	34	10.2080180412456	**
4690	5967748	66	35	8.97591840798281	**
4690	5967755	66	35	8.97591840798281	**
4690	5967782	65	36	7.82303287286116	**
4690	5967800	66	35	8.97591840798281	**
4690	5967849	66	35	8.97591840798281	**
4690	5967854	67	34	10.2080180412456	**
4690	5967872	67	34	10.2080180412456	**
4690	5967875	68	33	11.5193317726495	***
4690	5967878	69	32	12.9098596021945	***
4690	5967901	70	31	14.3796015298807	***
4690	5967924	70	31	14.3796015298807	***
4690	5967933	70	31	14.3796015298807	***
4690	5967938	70	31	14.3796015298807	***
4690	5967940	70	31	14.3796015298807	***
4690	5967941	69	32	12.9098596021945	***
4690	5967942	69	32	12.9098596021945	***
4690	5967956	70	31	14.3796015298807	***
4690	5967980	70	31	14.3796015298807	***
4690	5968000	69	32	12.9098596021945	***
4690	5968006	69	32	12.9098596021945	***
4690	5968012	69	32	12.9098596021945	***
4690	5968031	69	32	12.9098596021945	***
4690	5968048	69	32	12.9098596021945	***
4690	5968075	68	33	11.5193317726495	***
4690	5968088	68	33	11.5193317726495	***
4690	5968096	69	32	12.9098596021945	***
4690	5968107	68	33	11.5193317726495	***
4690	5968134	68	33	11.5193317726495	***
4690	5968151	68	33	11.5193317726495	***

4690	5968162	69	32	12.9098596021945	***
4690	5968172	68	33	11.5193317726495	***
4690	5968177	68	33	11.5193317726495	***
4690	5968183	67	34	10.2080180412456	**
4690	5968202	67	34	10.2080180412456	**
4690	5968218	66	35	8.97591840798281	**
4690	5968225	66	35	8.97591840798281	**
4690	5968240	67	34	10.2080180412456	**
4690	5968241	67	34	10.2080180412456	**
4690	5968242	66	35	8.97591840798281	**
4690	5968262	66	35	8.97591840798281	**
4690	5968264	67	34	10.2080180412456	**
4690	5968265	67	34	10.2080180412456	**
4690	5968306	67	34	10.2080180412456	**
4690	5968312	67	34	10.2080180412456	**
4690	5968320	67	34	10.2080180412456	**
4690	5968381	67	34	10.2080180412456	**
4690	5968398	68	33	11.5193317726495	***
4690	5968399	68	33	11.5193317726495	***
4690	5968412	67	34	10.2080180412456	**
4690	5968415	66	35	8.97591840798281	**
4690	5968432	65	36	7.82303287286116	**
4690	5968445	65	36	7.82303287286116	**
4690	5968449	65	36	7.82303287286116	**
4690	5968459	65	36	7.82303287286116	**
4690	5968463	65	36	7.82303287286116	**
4690	5968474	64	37	6.74936143588064	**
4690	5968498	63	38	5.75490409704125	*
4690	5968562	62	39	4.83966085634298	*
4690	5968565	61	40	4.00363171378585	*
4690	5968572	60	41	3.24681666936984	n.s.
4690	5968575	60	41	3.24681666936984	n.s.
4690	5968645	60	41	3.24681666936984	n.s.
4690	5968650	61	40	4.00363171378585	*
4690	5968656	61	40	4.00363171378585	*
4690	5968662	61	40	4.00363171378585	*
4690	5968666	61	40	4.00363171378585	*
4690	5968667	61	40	4.00363171378585	*
4690	5968697	61	40	4.00363171378585	*
4690	5968726	60	41	3.24681666936984	n.s.
4690	5968733	60	41	3.24681666936984	n.s.
4690	5968816	61	40	4.00363171378585	*
4690	5968835	60	41	3.24681666936984	n.s.
4690	5968837	59	42	2.56921572309496	n.s.
4690	5968864	58	43	1.97082887496121	n.s.
4690	5968869	58	43	1.97082887496121	n.s.
4690	5968906	57	44	1.45165612496859	n.s.
4690	5968969	58	43	1.97082887496121	n.s.
4690	5968992	57	44	1.45165612496859	n.s.
4690	5969000	57	44	1.45165612496859	n.s.

4690	5969006	57	44	1.45165612496859	n.s.
4690	5969007	56	45	1.0116974731171	n.s.
4690	5969044	55	46	0.650952919406731	n.s.
4690	5969046	54	47	0.369422463837495	n.s.
4690	5969076	54	47	0.369422463837495	n.s.
4690	5969086	53	48	0.167106106409387	n.s.
4690	5969109	53	48	0.167106106409387	n.s.
4690	5969129	53	48	0.167106106409387	n.s.
4690	5969134	53	48	0.167106106409387	n.s.
4690	5969173	52	49	0.044003847122408	n.s.
4690	5969223	51	50	0.000115685976557532	n.s.
4690	5969227	50	51	0.0354416229718356	n.s.
4690	5969280	49	52	0.149981658108242	n.s.
4690	5969283	49	52	0.149981658108242	n.s.
4690	5969284	50	51	0.0354416229718356	n.s.
4690	5969290	51	50	0.000115685976557532	n.s.
4690	5969301	50	51	0.0354416229718356	n.s.
4690	5969309	50	51	0.0354416229718356	n.s.
4690	5969313	49	52	0.149981658108242	n.s.
4690	5969316	49	52	0.149981658108242	n.s.
4690	5969325	48	53	0.343735791385777	n.s.
4690	5969328	48	53	0.343735791385777	n.s.
4690	5969337	49	52	0.149981658108242	n.s.
4690	5969341	50	51	0.0354416229718356	n.s.
4690	5969369	51	50	0.000115685976557532	n.s.
4690	5969390	51	50	0.000115685976557532	n.s.
4690	5969399	50	51	0.0354416229718356	n.s.
4690	5969401	50	51	0.0354416229718356	n.s.
4690	5969418	49	52	0.149981658108242	n.s.
4690	5969437	49	52	0.149981658108242	n.s.
4690	5969439	49	52	0.149981658108242	n.s.
4690	5969451	49	52	0.149981658108242	n.s.
4690	5969458	49	52	0.149981658108242	n.s.
4690	5969459	49	52	0.149981658108242	n.s.
4690	5969467	48	53	0.343735791385777	n.s.
4690	5969478	48	53	0.343735791385777	n.s.
4690	5969509	48	53	0.343735791385777	n.s.
4690	5969510	47	54	0.616704022804441	n.s.
4690	5969511	47	54	0.616704022804441	n.s.
4690	5969513	47	54	0.616704022804441	n.s.
4690	5969515	47	54	0.616704022804441	n.s.
4690	5969524	47	54	0.616704022804441	n.s.
4690	5969526	46	55	0.968886352364233	n.s.
4690	5969527	45	56	1.40028278006515	n.s.
4690	5969538	44	57	1.9108933059072	n.s.
4690	5969543	44	57	1.9108933059072	n.s.
4690	5969554	44	57	1.9108933059072	n.s.
4690	5969556	43	58	2.50071792989038	n.s.
4690	5969613	43	58	2.50071792989038	n.s.
4690	5969614	42	59	3.16975665201469	n.s.

4690	5969615	42	59	3.16975665201469	n.s.	
4690	5969630	42	59	3.16975665201469	n.s.	
4690	5969639	43	58	2.50071792989038	n.s.	
4690	5969652	44	57	1.9108933059072	n.s.	
4690	5969654	45	56	1.40028278006515	n.s.	
4690	5969678	45	56	1.40028278006515	n.s.	
4690	5969679	46	55	0.968886352364233	n.s.	
4690	5969701	46	55	0.968886352364233	n.s.	
4690	5969729	46	55	0.968886352364233	n.s.	
4690	5969775	47	54	0.616704022804441	n.s.	
4690	5969785	48	53	0.343735791385777	n.s.	
4690	5969789	47	54	0.616704022804441	n.s.	
4690	5969790	47	54	0.616704022804441	n.s.	
4690	5969850	48	53	0.343735791385777	n.s.	
4690	5969852	49	52	0.149981658108242	n.s.	
4690	5969853	48	53	0.343735791385777	n.s.	
4690	5969860	48	53	0.343735791385777	n.s.	
4690	5969874	49	52	0.149981658108242	n.s.	
4690	5969875	48	53	0.343735791385777	n.s.	
4690	5969877	47	54	0.616704022804441	n.s.	
4690	5969882	48	53	0.343735791385777	n.s.	
4690	5969890	48	53	0.343735791385777	n.s.	
4690	5969893	48	53	0.343735791385777	n.s.	
4690	5969918	48	53	0.343735791385777	n.s.	
4690	5969933	49	52	0.149981658108242	n.s.	
4690	5969949	48	53	0.343735791385777	n.s.	
4690	5969967	48	53	0.343735791385777	n.s.	
4690	5969971	49	52	0.149981658108242	n.s.	
4690	5969989	50	51	0.0354416229718356	n.s.	
4690	5969990	50	51	0.0354416229718356	n.s.	
4690	5969997	51	50	0.000115685976557532		n.s.
4690	5970001	51	50	0.000115685976557532		n.s.
4690	5970019	50	51	0.0354416229718356	n.s.	
4690	5970031	50	51	0.0354416229718356	n.s.	
4690	5970039	50	51	0.0354416229718356	n.s.	
4690	5970048	50	51	0.0354416229718356	n.s.	
4690	5970054	50	51	0.0354416229718356	n.s.	
4690	5970055	50	51	0.0354416229718356	n.s.	
4690	5970078	50	51	0.0354416229718356	n.s.	
4690	5970101	49	52	0.149981658108242	n.s.	
4690	5970126	50	51	0.0354416229718356	n.s.	
4690	5970127	50	51	0.0354416229718356	n.s.	
4690	5970132	50	51	0.0354416229718356	n.s.	
4690	5970153	51	50	0.000115685976557532		n.s.
4690	5970162	52	49	0.044003847122408	n.s.	
4690	5970182	52	49	0.044003847122408	n.s.	
4690	5970185	53	48	0.167106106409387	n.s.	
4690	5970195	53	48	0.167106106409387	n.s.	
4690	5970210	52	49	0.044003847122408	n.s.	
4690	5970218	53	48	0.167106106409387	n.s.	

4690	5970220	52	49	0.044003847122408	n.s.	
4690	5970234	51	50	0.000115685976557532		n.s.
4690	5970239	52	49	0.044003847122408	n.s.	
4690	5970256	52	49	0.044003847122408	n.s.	
4690	5970258	52	49	0.044003847122408	n.s.	
4690	5970259	52	49	0.044003847122408	n.s.	
4690	5970261	52	49	0.044003847122408	n.s.	
4690	5970262	51	50	0.000115685976557532		n.s.
4690	5970263	52	49	0.044003847122408	n.s.	
4690	5970264	52	49	0.044003847122408	n.s.	
4690	5970269	52	49	0.044003847122408	n.s.	
4690	5970273	52	49	0.044003847122408	n.s.	
4690	5970277	53	48	0.167106106409387	n.s.	
4690	5970278	54	47	0.369422463837495	n.s.	
4690	5970301	54	47	0.369422463837495	n.s.	
4690	5970310	53	48	0.167106106409387	n.s.	
4690	5970325	53	48	0.167106106409387	n.s.	
4690	5970329	54	47	0.369422463837495	n.s.	
4690	5970334	53	48	0.167106106409387	n.s.	
4690	5970348	53	48	0.167106106409387	n.s.	
4690	5970357	53	48	0.167106106409387	n.s.	
4690	5970364	54	47	0.369422463837495	n.s.	
4690	5970368	53	48	0.167106106409387	n.s.	
4690	5970369	52	49	0.044003847122408	n.s.	
4690	5970402	51	50	0.000115685976557532		n.s.
4690	5970442	51	50	0.000115685976557532		n.s.
4690	5970460	50	51	0.0354416229718356	n.s.	
4690	5970484	51	50	0.000115685976557532		n.s.
4690	5970499	51	50	0.000115685976557532		n.s.
4690	5970627	51	50	0.000115685976557532		n.s.
4690	5970640	51	50	0.000115685976557532		n.s.
4690	5970641	50	51	0.0354416229718356	n.s.	
4690	5970685	50	51	0.0354416229718356	n.s.	
4690	5970697	49	52	0.149981658108242	n.s.	
4690	5970729	50	51	0.0354416229718356	n.s.	
4690	5970730	51	50	0.000115685976557532		n.s.
4690	5970732	50	51	0.0354416229718356	n.s.	
4690	5970740	51	50	0.000115685976557532		n.s.
4690	5970750	51	50	0.000115685976557532		n.s.
4690	5970771	51	50	0.000115685976557532		n.s.
4690	5970772	51	50	0.000115685976557532		n.s.
4690	5970789	52	49	0.044003847122408	n.s.	
4690	5970790	51	50	0.000115685976557532		n.s.
4690	5970819	52	49	0.044003847122408	n.s.	
4690	5970825	52	49	0.044003847122408	n.s.	
4690	5970909	53	48	0.167106106409387	n.s.	
4690	5970923	54	47	0.369422463837495	n.s.	
4690	5970940	54	47	0.369422463837495	n.s.	
4690	5970947	55	46	0.650952919406731	n.s.	
4690	5970949	56	45	1.0116974731171	n.s.	

4690	5970951	57	44	1.45165612496859	n.s.	
4690	5970966	56	45	1.0116974731171	n.s.	
4690	5970968	56	45	1.0116974731171	n.s.	
4690	5970988	56	45	1.0116974731171	n.s.	
4690	5970995	55	46	0.650952919406731	n.s.	
4690	5971006	55	46	0.650952919406731	n.s.	
4690	5971015	54	47	0.369422463837495	n.s.	
4690	5971016	54	47	0.369422463837495	n.s.	
4690	5971040	53	48	0.167106106409387	n.s.	
4690	5971057	52	49	0.044003847122408	n.s.	
4690	5971067	51	50	0.000115685976557532		n.s.
4690	5971075	51	50	0.000115685976557532		n.s.
4690	5971099	52	49	0.044003847122408	n.s.	
4690	5971104	52	49	0.044003847122408	n.s.	
4690	5971107	52	49	0.044003847122408	n.s.	
4690	5971121	52	49	0.044003847122408	n.s.	
4690	5971132	53	48	0.167106106409387	n.s.	
4690	5971138	54	47	0.369422463837495	n.s.	
4690	5971165	54	47	0.369422463837495	n.s.	
4690	5971170	55	46	0.650952919406731	n.s.	
4690	5971172	56	45	1.0116974731171	n.s.	
4690	5971174	56	45	1.0116974731171	n.s.	
4690	5971181	57	44	1.45165612496859	n.s.	
4690	5971184	57	44	1.45165612496859	n.s.	
4690	5971187	57	44	1.45165612496859	n.s.	
4690	5971201	56	45	1.0116974731171	n.s.	
4690	5971205	57	44	1.45165612496859	n.s.	
4690	5971206	57	44	1.45165612496859	n.s.	
4690	5971223	56	45	1.0116974731171	n.s.	
4690	5971241	55	46	0.650952919406731	n.s.	
4690	5971249	56	45	1.0116974731171	n.s.	
4690	5971255	56	45	1.0116974731171	n.s.	
4690	5971262	56	45	1.0116974731171	n.s.	
4690	5971263	57	44	1.45165612496859	n.s.	
4690	5971268	58	43	1.97082887496121	n.s.	
4690	5971276	57	44	1.45165612496859	n.s.	
4690	5971279	57	44	1.45165612496859	n.s.	
4690	5971288	58	43	1.97082887496121	n.s.	
4690	5971294	58	43	1.97082887496121	n.s.	
4690	5971301	58	43	1.97082887496121	n.s.	
4690	5971304	59	42	2.56921572309496	n.s.	
4690	5971311	58	43	1.97082887496121	n.s.	
4690	5971345	58	43	1.97082887496121	n.s.	
4690	5971481	57	44	1.45165612496859	n.s.	
4690	5971488	57	44	1.45165612496859	n.s.	
4690	5971495	56	45	1.0116974731171	n.s.	
4690	5971541	57	44	1.45165612496859	n.s.	
4690	5971591	57	44	1.45165612496859	n.s.	
4690	5971601	58	43	1.97082887496121	n.s.	
4690	5971605	59	42	2.56921572309496	n.s.	

4690	5971623	59	42	2.56921572309496	n.s.
4690	5971627	60	41	3.24681666936984	n.s.
4690	5971640	61	40	4.00363171378585	*
4690	5971647	61	40	4.00363171378585	*
4690	5971660	62	39	4.83966085634298	*
4690	5971690	63	38	5.75490409704125	*
4690	5971713	64	37	6.74936143588064	**
4690	5971723	64	37	6.74936143588064	**
4690	5971734	65	36	7.82303287286116	**
4690	5971767	65	36	7.82303287286116	**
4690	5971776	66	35	8.97591840798281	**
4690	5971828	67	34	10.2080180412456	**
4690	5971838	68	33	11.5193317726495	***
4690	5971869	68	33	11.5193317726495	***
4690	5971878	68	33	11.5193317726495	***
4690	5971887	68	33	11.5193317726495	***
4690	5971898	69	32	12.9098596021945	***
4690	5971905	69	32	12.9098596021945	***
4690	5971918	69	32	12.9098596021945	***
4690	5971956	70	31	14.3796015298807	***
4690	5971960	71	30	15.928557555708	***
4690	5971974	72	29	17.5567276796764	***
4690	5971980	71	30	15.928557555708	***
4690	5971998	71	30	15.928557555708	***
4690	5972007	72	29	17.5567276796764	***
4690	5972028	73	28	19.264111901786	***
4690	5972051	72	29	17.5567276796764	***
4690	5972055	72	29	17.5567276796764	***
4690	5972074	71	30	15.928557555708	***
4690	5972083	71	30	15.928557555708	***
4690	5972088	71	30	15.928557555708	***
4690	5972094	71	30	15.928557555708	***
4690	5972110	72	29	17.5567276796764	***
4690	5972111	71	30	15.928557555708	***
4690	5972119	71	30	15.928557555708	***
4690	5972122	71	30	15.928557555708	***
4690	5972125	70	31	14.3796015298807	***
4690	5972134	71	30	15.928557555708	***
4690	5972135	70	31	14.3796015298807	***
4690	5972148	71	30	15.928557555708	***
4690	5972157	70	31	14.3796015298807	***
4690	5972179	69	32	12.9098596021945	***
4690	5972190	69	32	12.9098596021945	***
4690	5972246	69	32	12.9098596021945	***
4690	5972282	68	33	11.5193317726495	***
4690	5972291	68	33	11.5193317726495	***
4690	5972312	67	34	10.2080180412456	**
4690	5972330	67	34	10.2080180412456	**
4690	5972338	67	34	10.2080180412456	**
4690	5972342	66	35	8.97591840798281	**

4690	5972354	66	35	8.97591840798281	**
4690	5972356	66	35	8.97591840798281	**
4690	5972363	66	35	8.97591840798281	**
4690	5972372	65	36	7.82303287286116	**
4690	5972375	64	37	6.74936143588064	**
4690	5972386	64	37	6.74936143588064	**
4690	5972414	64	37	6.74936143588064	**
4690	5972417	65	36	7.82303287286116	**
4690	5972422	64	37	6.74936143588064	**
4690	5972435	65	36	7.82303287286116	**
4690	5972441	65	36	7.82303287286116	**
4690	5972447	65	36	7.82303287286116	**
4690	5972468	64	37	6.74936143588064	**
4690	5972501	63	38	5.75490409704125	*
4690	5972516	62	39	4.83966085634298	*
4690	5972519	61	40	4.00363171378585	*
4690	5972535	60	41	3.24681666936984	n.s.
4690	5972539	59	42	2.56921572309496	n.s.
4690	5972561	59	42	2.56921572309496	n.s.
4690	5972564	58	43	1.97082887496121	n.s.
4690	5972690	58	43	1.97082887496121	n.s.
4690	5972699	57	44	1.45165612496859	n.s.
4690	5972705	56	45	1.0116974731171	n.s.
4690	5972711	56	45	1.0116974731171	n.s.
4690	5972729	56	45	1.0116974731171	n.s.
4690	5972741	56	45	1.0116974731171	n.s.
4690	5972747	57	44	1.45165612496859	n.s.
4690	5972750	57	44	1.45165612496859	n.s.
4690	5972774	57	44	1.45165612496859	n.s.
4690	5972778	58	43	1.97082887496121	n.s.
4690	5972813	58	43	1.97082887496121	n.s.
4690	5972826	57	44	1.45165612496859	n.s.
4690	5972887	57	44	1.45165612496859	n.s.
4690	5972898	57	44	1.45165612496859	n.s.
4690	5972919	56	45	1.0116974731171	n.s.
4690	5972927	55	46	0.650952919406731	n.s.
4690	5972932	55	46	0.650952919406731	n.s.
4690	5972937	56	45	1.0116974731171	n.s.
4690	5972952	55	46	0.650952919406731	n.s.
4690	5972982	55	46	0.650952919406731	n.s.
4690	5973035	55	46	0.650952919406731	n.s.
4690	5973041	55	46	0.650952919406731	n.s.
4690	5973042	56	45	1.0116974731171	n.s.
4690	5973045	56	45	1.0116974731171	n.s.
4690	5973057	56	45	1.0116974731171	n.s.
4690	5973058	56	45	1.0116974731171	n.s.
4690	5973066	56	45	1.0116974731171	n.s.
4690	5973068	55	46	0.650952919406731	n.s.
4690	5973076	54	47	0.369422463837495	n.s.
4690	5973079	53	48	0.167106106409387	n.s.

4690	5973095	53	48	0.167106106409387	n.s.	
4690	5973102	53	48	0.167106106409387	n.s.	
4690	5973114	53	48	0.167106106409387	n.s.	
4690	5973116	52	49	0.044003847122408	n.s.	
4690	5973119	51	50	0.000115685976557532		n.s.
4690	5973128	50	51	0.0354416229718356	n.s.	
4690	5973155	50	51	0.0354416229718356	n.s.	
4690	5973162	49	52	0.149981658108242	n.s.	
4690	5973165	50	51	0.0354416229718356	n.s.	
4690	5973186	49	52	0.149981658108242	n.s.	
4690	5973187	48	53	0.343735791385777	n.s.	
4690	5973202	47	54	0.616704022804441	n.s.	
4690	5973205	47	54	0.616704022804441	n.s.	
4690	5973228	46	55	0.968886352364233	n.s.	
4690	5973232	45	56	1.40028278006515	n.s.	
4690	5973233	44	57	1.9108933059072	n.s.	
4690	5973246	43	58	2.50071792989038	n.s.	
4690	5973271	43	58	2.50071792989038	n.s.	
4690	5973280	42	59	3.16975665201469	n.s.	
4690	5973281	41	60	3.91800947228012	*	
4690	5973294	40	61	4.74547639068669	*	
4690	5973308	39	62	5.65215740723438	*	
4690	5973324	39	62	5.65215740723438	*	
4690	5973341	39	62	5.65215740723438	*	
4690	5973364	39	62	5.65215740723438	*	
4690	5973369	39	62	5.65215740723438	*	
4690	5973371	38	63	6.6380525219232	*	
4690	5973372	38	63	6.6380525219232	*	
4690	5973376	39	62	5.65215740723438	*	
4690	5973380	39	62	5.65215740723438	*	
4690	5973391	39	62	5.65215740723438	*	
4690	5973395	40	61	4.74547639068669	*	
4690	5973398	40	61	4.74547639068669	*	
4690	5973408	39	62	5.65215740723438	*	
4690	5973426	39	62	5.65215740723438	*	
4690	5973531	40	61	4.74547639068669	*	
4690	5973593	39	62	5.65215740723438	*	
4690	5973607	40	61	4.74547639068669	*	
4690	5973640	40	61	4.74547639068669	*	
4690	5973648	41	60	3.91800947228012	*	
4690	5973662	40	61	4.74547639068669	*	
4690	5973704	41	60	3.91800947228012	*	
4690	5973705	42	59	3.16975665201469	n.s.	
4690	5973709	42	59	3.16975665201469	n.s.	
4690	5973722	43	58	2.50071792989038	n.s.	
4690	5973726	44	57	1.9108933059072	n.s.	
4690	5973730	45	56	1.40028278006515	n.s.	
4690	5973733	46	55	0.968886352364233	n.s.	
4690	5973812	46	55	0.968886352364233	n.s.	
4690	5973821	46	55	0.968886352364233	n.s.	

4690	5973827	47	54	0.616704022804441	n.s.
4690	5973847	47	54	0.616704022804441	n.s.
4690	5973852	46	55	0.968886352364233	n.s.
4690	5973889	47	54	0.616704022804441	n.s.
4690	5973900	48	53	0.343735791385777	n.s.
4690	5973921	49	52	0.149981658108242	n.s.
4690	5973965	49	52	0.149981658108242	n.s.
4690	5973985	49	52	0.149981658108242	n.s.
4690	5973987	49	52	0.149981658108242	n.s.
4690	5974029	49	52	0.149981658108242	n.s.
4690	5974046	49	52	0.149981658108242	n.s.
4690	5974076	50	51	0.0354416229718356	n.s.
4690	5974092	51	50	0.000115685976557532	n.s.
4690	5974095	52	49	0.044003847122408	n.s.
4690	5974098	52	49	0.044003847122408	n.s.
4690	5974109	53	48	0.167106106409387	n.s.
4690	5974121	53	48	0.167106106409387	n.s.
4690	5974126	53	48	0.167106106409387	n.s.
4690	5974131	54	47	0.369422463837495	n.s.
4690	5974144	54	47	0.369422463837495	n.s.
4690	5974145	54	47	0.369422463837495	n.s.
4690	5974150	54	47	0.369422463837495	n.s.
4690	5974158	55	46	0.650952919406731	n.s.
4690	5974159	56	45	1.0116974731171	n.s.
4690	5974164	56	45	1.0116974731171	n.s.
4690	5974183	57	44	1.45165612496859	n.s.
4690	5974203	57	44	1.45165612496859	n.s.
4690	5974214	57	44	1.45165612496859	n.s.
4690	5974243	56	45	1.0116974731171	n.s.
4690	5974248	56	45	1.0116974731171	n.s.
4690	5974249	55	46	0.650952919406731	n.s.
4690	5974251	56	45	1.0116974731171	n.s.
4690	5974253	56	45	1.0116974731171	n.s.
4690	5974257	55	46	0.650952919406731	n.s.
4690	5974290	56	45	1.0116974731171	n.s.
4690	5974301	56	45	1.0116974731171	n.s.
4690	5974311	56	45	1.0116974731171	n.s.
4690	5974329	57	44	1.45165612496859	n.s.
4690	5974332	57	44	1.45165612496859	n.s.
4690	5974346	57	44	1.45165612496859	n.s.
4690	5974359	58	43	1.97082887496121	n.s.
4690	5974371	57	44	1.45165612496859	n.s.
4690	5974428	57	44	1.45165612496859	n.s.
4690	5974433	56	45	1.0116974731171	n.s.
4690	5974441	55	46	0.650952919406731	n.s.
4690	5974456	55	46	0.650952919406731	n.s.
4690	5974461	54	47	0.369422463837495	n.s.
4690	5974465	55	46	0.650952919406731	n.s.
4690	5974466	55	46	0.650952919406731	n.s.
4690	5974469	56	45	1.0116974731171	n.s.

4690	5974485	56	45	1.0116974731171	n.s.
4690	5974486	55	46	0.650952919406731	n.s.
4690	5974491	54	47	0.369422463837495	n.s.
4690	5974504	53	48	0.167106106409387	n.s.
4690	5974509	53	48	0.167106106409387	n.s.
4690	5974521	53	48	0.167106106409387	n.s.
4690	5974527	53	48	0.167106106409387	n.s.
4690	5974541	52	49	0.044003847122408	n.s.
4690	5974567	52	49	0.044003847122408	n.s.
4690	5974590	52	49	0.044003847122408	n.s.
4690	5974593	52	49	0.044003847122408	n.s.
4690	5974605	52	49	0.044003847122408	n.s.
4690	5974608	52	49	0.044003847122408	n.s.
4690	5974638	51	50	0.000115685976557532	n.s.
4690	5974662	51	50	0.000115685976557532	n.s.
4690	5974693	52	49	0.044003847122408	n.s.
4690	5974699	52	49	0.044003847122408	n.s.
4690	5974701	52	49	0.044003847122408	n.s.
4690	5974738	51	50	0.000115685976557532	n.s.
4690	5974746	51	50	0.000115685976557532	n.s.
4690	5974755	51	50	0.000115685976557532	n.s.
4690	5974759	51	50	0.000115685976557532	n.s.
4690	5974805	52	49	0.044003847122408	n.s.
4690	5974811	51	50	0.000115685976557532	n.s.
4690	5974825	52	49	0.044003847122408	n.s.
4690	5974839	53	48	0.167106106409387	n.s.
4690	5974900	52	49	0.044003847122408	n.s.
4690	5975022	53	48	0.167106106409387	n.s.
4690	5975034	53	48	0.167106106409387	n.s.
4690	5975042	53	48	0.167106106409387	n.s.
4690	5975076	54	47	0.369422463837495	n.s.
4690	5975086	53	48	0.167106106409387	n.s.
4690	5975112	53	48	0.167106106409387	n.s.
4690	5975127	52	49	0.044003847122408	n.s.
4690	5975130	52	49	0.044003847122408	n.s.
4690	5975148	53	48	0.167106106409387	n.s.
4690	5975172	53	48	0.167106106409387	n.s.
4690	5975175	54	47	0.369422463837495	n.s.
4690	5975191	54	47	0.369422463837495	n.s.
4690	5975200	54	47	0.369422463837495	n.s.
4690	5975208	53	48	0.167106106409387	n.s.
4690	5975219	54	47	0.369422463837495	n.s.
4690	5975228	54	47	0.369422463837495	n.s.
4690	5975231	54	47	0.369422463837495	n.s.
4690	5975236	54	47	0.369422463837495	n.s.
4690	5975251	54	47	0.369422463837495	n.s.
4690	5975252	54	47	0.369422463837495	n.s.
4690	5975254	54	47	0.369422463837495	n.s.
4690	5975261	54	47	0.369422463837495	n.s.
4690	5975289	54	47	0.369422463837495	n.s.

4690	5975314	54	47	0.369422463837495	n.s.
4690	5975331	54	47	0.369422463837495	n.s.
4690	5975339	54	47	0.369422463837495	n.s.
4690	5975359	55	46	0.650952919406731	n.s.
4690	5975418	54	47	0.369422463837495	n.s.
4690	5975474	54	47	0.369422463837495	n.s.
4690	5975594	54	47	0.369422463837495	n.s.
4690	5975605	55	46	0.650952919406731	n.s.
4690	5975614	55	46	0.650952919406731	n.s.
4690	5975634	55	46	0.650952919406731	n.s.
4690	5975648	56	45	1.0116974731171	n.s.
4690	5975664	56	45	1.0116974731171	n.s.
4690	5975692	56	45	1.0116974731171	n.s.
4690	5975707	55	46	0.650952919406731	n.s.
4690	5975711	54	47	0.369422463837495	n.s.
4690	5975725	55	46	0.650952919406731	n.s.
4690	5975774	54	47	0.369422463837495	n.s.
4690	5975785	54	47	0.369422463837495	n.s.
4690	5975800	54	47	0.369422463837495	n.s.
4690	5975801	54	47	0.369422463837495	n.s.
4690	5975803	54	47	0.369422463837495	n.s.
4690	5975820	55	46	0.650952919406731	n.s.
4690	5975848	55	46	0.650952919406731	n.s.
4690	5975904	54	47	0.369422463837495	n.s.
4690	5975905	53	48	0.167106106409387	n.s.
4690	5975920	53	48	0.167106106409387	n.s.
4690	5975951	52	49	0.044003847122408	n.s.
4690	5975969	51	50	0.000115685976557532	n.s.
4690	5975977	50	51	0.0354416229718356	n.s.
4690	5975995	51	50	0.000115685976557532	n.s.
4690	5976036	50	51	0.0354416229718356	n.s.
4690	5976053	51	50	0.000115685976557532	n.s.
4690	5976075	50	51	0.0354416229718356	n.s.
4690	5976091	50	51	0.0354416229718356	n.s.
4690	5976098	50	51	0.0354416229718356	n.s.
4690	5976108	50	51	0.0354416229718356	n.s.
4690	5976112	51	50	0.000115685976557532	n.s.
4690	5976120	52	49	0.044003847122408	n.s.
4690	5976124	52	49	0.044003847122408	n.s.
4690	5976133	51	50	0.000115685976557532	n.s.
4690	5976134	52	49	0.044003847122408	n.s.
4690	5976143	51	50	0.000115685976557532	n.s.
4690	5976161	51	50	0.000115685976557532	n.s.
4690	5976178	50	51	0.0354416229718356	n.s.
4690	5976181	50	51	0.0354416229718356	n.s.
4690	5976187	51	50	0.000115685976557532	n.s.
4690	5976190	52	49	0.044003847122408	n.s.
4690	5976211	53	48	0.167106106409387	n.s.
4690	5976213	52	49	0.044003847122408	n.s.
4690	5976214	52	49	0.044003847122408	n.s.

4690	5976218	52	49	0.044003847122408	n.s.
4690	5976220	53	48	0.167106106409387	n.s.
4690	5976227	54	47	0.369422463837495	n.s.
4690	5976236	54	47	0.369422463837495	n.s.
4690	5976237	55	46	0.650952919406731	n.s.
4690	5976238	55	46	0.650952919406731	n.s.
4690	5976245	56	45	1.0116974731171	n.s.
4690	5976250	56	45	1.0116974731171	n.s.
4690	5976265	56	45	1.0116974731171	n.s.
4690	5976268	56	45	1.0116974731171	n.s.
4690	5976283	56	45	1.0116974731171	n.s.
4690	5976292	57	44	1.45165612496859	n.s.
4690	5976314	58	43	1.97082887496121	n.s.
4690	5976316	59	42	2.56921572309496	n.s.
4690	5976325	60	41	3.24681666936984	n.s.
4690	5976337	60	41	3.24681666936984	n.s.
4690	5976346	59	42	2.56921572309496	n.s.
4690	5976361	59	42	2.56921572309496	n.s.
4690	5976380	59	42	2.56921572309496	n.s.
4690	5976415	59	42	2.56921572309496	n.s.
4690	5976416	59	42	2.56921572309496	n.s.
4690	5976420	59	42	2.56921572309496	n.s.
4690	5976430	60	41	3.24681666936984	n.s.
4690	5976431	59	42	2.56921572309496	n.s.
4690	5976444	60	41	3.24681666936984	n.s.
4690	5976452	59	42	2.56921572309496	n.s.
4690	5976469	59	42	2.56921572309496	n.s.
4690	5976475	59	42	2.56921572309496	n.s.
4690	5976502	58	43	1.97082887496121	n.s.
4690	5976507	58	43	1.97082887496121	n.s.
4690	5976511	58	43	1.97082887496121	n.s.
4690	5976520	57	44	1.45165612496859	n.s.
4690	5976523	57	44	1.45165612496859	n.s.
4690	5976548	56	45	1.0116974731171	n.s.
4690	5976555	57	44	1.45165612496859	n.s.
4690	5976557	58	43	1.97082887496121	n.s.
4690	5976562	57	44	1.45165612496859	n.s.
4690	5976590	56	45	1.0116974731171	n.s.
4690	5976592	55	46	0.650952919406731	n.s.
4690	5976607	55	46	0.650952919406731	n.s.
4690	5976612	54	47	0.369422463837495	n.s.
4690	5976620	55	46	0.650952919406731	n.s.
4690	5976624	55	46	0.650952919406731	n.s.
4690	5976634	54	47	0.369422463837495	n.s.
4690	5976649	53	48	0.167106106409387	n.s.
4690	5976656	52	49	0.044003847122408	n.s.
4690	5976657	51	50	0.000115685976557532	n.s.
4690	5976763	50	51	0.0354416229718356	n.s.
4690	5976777	49	52	0.149981658108242	n.s.
4690	5976790	48	53	0.343735791385777	n.s.

4690	5976797	47	54	0.616704022804441	n.s.
4690	5976802	46	55	0.968886352364233	n.s.
4690	5976809	45	56	1.40028278006515	n.s.
4690	5976825	45	56	1.40028278006515	n.s.
4690	5976826	44	57	1.9108933059072	n.s.
4690	5976853	45	56	1.40028278006515	n.s.
4690	5976856	45	56	1.40028278006515	n.s.
4690	5976874	45	56	1.40028278006515	n.s.
4690	5976877	45	56	1.40028278006515	n.s.
4690	5976881	45	56	1.40028278006515	n.s.
4690	5976883	45	56	1.40028278006515	n.s.
4690	5976901	44	57	1.9108933059072	n.s.
4690	5976916	44	57	1.9108933059072	n.s.
4690	5976926	43	58	2.50071792989038	n.s.
4690	5976974	43	58	2.50071792989038	n.s.
4690	5976985	43	58	2.50071792989038	n.s.
4690	5976996	42	59	3.16975665201469	n.s.
4690	5977009	42	59	3.16975665201469	n.s.
4690	5977024	43	58	2.50071792989038	n.s.
4690	5977030	43	58	2.50071792989038	n.s.
4690	5977095	42	59	3.16975665201469	n.s.
4690	5977102	42	59	3.16975665201469	n.s.
4690	5977108	42	59	3.16975665201469	n.s.
4690	5977113	41	60	3.91800947228012	*
4690	5977118	41	60	3.91800947228012	*
4690	5977120	41	60	3.91800947228012	*
4690	5977123	41	60	3.91800947228012	*
4690	5977131	41	60	3.91800947228012	*
4690	5977138	42	59	3.16975665201469	n.s.
4690	5977155	42	59	3.16975665201469	n.s.
4690	5977159	42	59	3.16975665201469	n.s.
4690	5977177	42	59	3.16975665201469	n.s.
4690	5977187	41	60	3.91800947228012	*
4690	5977189	42	59	3.16975665201469	n.s.
4690	5977208	42	59	3.16975665201469	n.s.
4690	5977222	42	59	3.16975665201469	n.s.
4690	5977232	41	60	3.91800947228012	*
4690	5977235	40	61	4.74547639068669	*
4690	5977251	39	62	5.65215740723438	*
4690	5977271	39	62	5.65215740723438	*
4690	5977290	39	62	5.65215740723438	*
4690	5977292	39	62	5.65215740723438	*
4690	5977297	39	62	5.65215740723438	*
4690	5977348	39	62	5.65215740723438	*
4690	5977349	40	61	4.74547639068669	*
4690	5977364	40	61	4.74547639068669	*
4690	5977371	39	62	5.65215740723438	*
4690	5977374	39	62	5.65215740723438	*
4690	5977403	39	62	5.65215740723438	*
4690	5977413	40	61	4.74547639068669	*

4690	5977418	40	61	4.74547639068669	*
4690	5977424	40	61	4.74547639068669	*
4690	5977432	40	61	4.74547639068669	*
4690	5977455	40	61	4.74547639068669	*
4690	5977464	40	61	4.74547639068669	*
4690	5977469	40	61	4.74547639068669	*
4690	5977470	41	60	3.91800947228012	*
4690	5977482	41	60	3.91800947228012	*
4690	5977485	41	60	3.91800947228012	*
4690	5977491	41	60	3.91800947228012	*
4690	5977515	41	60	3.91800947228012	*
4690	5977528	41	60	3.91800947228012	*
4690	5977538	41	60	3.91800947228012	*
4690	5977546	40	61	4.74547639068669	*
4690	5977548	39	62	5.65215740723438	*
4690	5977550	39	62	5.65215740723438	*
4690	5977558	40	61	4.74547639068669	*
4690	5977562	40	61	4.74547639068669	*
4690	5977568	40	61	4.74547639068669	*
4690	5977577	40	61	4.74547639068669	*
4690	5977584	40	61	4.74547639068669	*
4690	5977588	41	60	3.91800947228012	*
4690	5977596	42	59	3.16975665201469	n.s.
4690	5977599	41	60	3.91800947228012	*
4690	5977605	42	59	3.16975665201469	n.s.
4690	5977614	41	60	3.91800947228012	*
4690	5977624	41	60	3.91800947228012	*
4690	5977629	40	61	4.74547639068669	*
4690	5977636	41	60	3.91800947228012	*
4690	5977649	42	59	3.16975665201469	n.s.
4690	5977658	43	58	2.50071792989038	n.s.
4690	5977668	43	58	2.50071792989038	n.s.
4690	5977671	43	58	2.50071792989038	n.s.
4690	5977674	43	58	2.50071792989038	n.s.
4690	5977691	44	57	1.9108933059072	n.s.
4690	5977708	43	58	2.50071792989038	n.s.
4690	5977712	43	58	2.50071792989038	n.s.
4690	5977715	43	58	2.50071792989038	n.s.
4690	5977723	43	58	2.50071792989038	n.s.
4690	5977729	43	58	2.50071792989038	n.s.
4690	5977732	43	58	2.50071792989038	n.s.
4690	5977743	43	58	2.50071792989038	n.s.
4690	5977747	42	59	3.16975665201469	n.s.
4690	5977751	41	60	3.91800947228012	*
4690	5977756	41	60	3.91800947228012	*
4690	5977759	41	60	3.91800947228012	*
4690	5977761	41	60	3.91800947228012	*
4690	5977763	41	60	3.91800947228012	*
4690	5977765	41	60	3.91800947228012	*
4690	5977770	42	59	3.16975665201469	n.s.

4690	5977780	43	58	2.50071792989038	n.s.
4690	5977783	44	57	1.9108933059072	n.s.
4690	5977788	44	57	1.9108933059072	n.s.
4690	5977795	45	56	1.40028278006515	n.s.
4690	5977798	44	57	1.9108933059072	n.s.
4690	5977812	44	57	1.9108933059072	n.s.
4690	5977815	44	57	1.9108933059072	n.s.
4690	5977817	44	57	1.9108933059072	n.s.
4690	5977827	44	57	1.9108933059072	n.s.
4690	5977829	43	58	2.50071792989038	n.s.
4690	5977844	43	58	2.50071792989038	n.s.
4690	5977897	43	58	2.50071792989038	n.s.
4690	5977942	43	58	2.50071792989038	n.s.
4690	5978004	42	59	3.16975665201469	n.s.
4690	5978017	43	58	2.50071792989038	n.s.
4690	5978028	44	57	1.9108933059072	n.s.
4690	5978044	44	57	1.9108933059072	n.s.
4690	5978049	45	56	1.40028278006515	n.s.
4690	5978068	45	56	1.40028278006515	n.s.
4690	5978095	44	57	1.9108933059072	n.s.
4690	5978097	44	57	1.9108933059072	n.s.
4690	5978103	45	56	1.40028278006515	n.s.
4690	5978127	45	56	1.40028278006515	n.s.
4690	5978136	44	57	1.9108933059072	n.s.
4690	5978203	44	57	1.9108933059072	n.s.
4690	5978204	44	57	1.9108933059072	n.s.
4690	5978212	44	57	1.9108933059072	n.s.
4690	5978220	43	58	2.50071792989038	n.s.
4690	5978246	43	58	2.50071792989038	n.s.
4690	5978269	42	59	3.16975665201469	n.s.
4690	5978279	43	58	2.50071792989038	n.s.
4690	5978283	42	59	3.16975665201469	n.s.
4690	5978286	43	58	2.50071792989038	n.s.
4690	5978297	43	58	2.50071792989038	n.s.
4690	5978309	42	59	3.16975665201469	n.s.
4690	5978316	42	59	3.16975665201469	n.s.
4690	5978325	43	58	2.50071792989038	n.s.
4690	5978329	44	57	1.9108933059072	n.s.
4690	5978349	44	57	1.9108933059072	n.s.
4690	5978351	44	57	1.9108933059072	n.s.
4690	5978368	43	58	2.50071792989038	n.s.
4690	5978371	44	57	1.9108933059072	n.s.
4690	5978380	44	57	1.9108933059072	n.s.
4690	5978384	45	56	1.40028278006515	n.s.
4690	5978407	46	55	0.968886352364233	n.s.
4690	5978413	46	55	0.968886352364233	n.s.
4690	5978440	47	54	0.616704022804441	n.s.
4690	5978443	47	54	0.616704022804441	n.s.
4690	5978447	47	54	0.616704022804441	n.s.
4690	5978456	46	55	0.968886352364233	n.s.

4690	5978477	45	56	1.40028278006515	n.s.	
4690	5978500	46	55	0.968886352364233	n.s.	
4690	5978509	45	56	1.40028278006515	n.s.	
4690	5978534	44	57	1.9108933059072	n.s.	
4690	5978540	44	57	1.9108933059072	n.s.	
4690	5978596	44	57	1.9108933059072	n.s.	
4690	5978609	44	57	1.9108933059072	n.s.	
4690	5978610	43	58	2.50071792989038	n.s.	
4690	5978625	43	58	2.50071792989038	n.s.	
4690	5978627	44	57	1.9108933059072	n.s.	
4690	5978628	45	56	1.40028278006515	n.s.	
4690	5978631	45	56	1.40028278006515	n.s.	
4690	5978639	45	56	1.40028278006515	n.s.	
4690	5978652	45	56	1.40028278006515	n.s.	
4690	5978655	45	56	1.40028278006515	n.s.	
4690	5978661	45	56	1.40028278006515	n.s.	
4690	5978670	44	57	1.9108933059072	n.s.	
4690	5978676	44	57	1.9108933059072	n.s.	
4690	5978679	45	56	1.40028278006515	n.s.	
4690	5978682	46	55	0.968886352364233	n.s.	
4690	5978696	47	54	0.616704022804441	n.s.	
4690	5978708	47	54	0.616704022804441	n.s.	
4690	5978718	46	55	0.968886352364233	n.s.	
4690	5978724	46	55	0.968886352364233	n.s.	
4690	5978741	47	54	0.616704022804441	n.s.	
4690	5978757	47	54	0.616704022804441	n.s.	
4690	5978778	48	53	0.343735791385777	n.s.	
4690	5978786	49	52	0.149981658108242	n.s.	
4690	5978791	50	51	0.0354416229718356	n.s.	
4690	5978793	50	51	0.0354416229718356	n.s.	
4690	5978819	50	51	0.0354416229718356	n.s.	
4690	5978820	50	51	0.0354416229718356	n.s.	
4690	5978861	50	51	0.0354416229718356	n.s.	
4690	5978865	51	50	0.000115685976557532		n.s.
4690	5978871	52	49	0.044003847122408	n.s.	
4690	5978881	52	49	0.044003847122408	n.s.	
4690	5978891	52	49	0.044003847122408	n.s.	
4690	5978905	52	49	0.044003847122408	n.s.	
4690	5978918	53	48	0.167106106409387	n.s.	
4690	5978927	54	47	0.369422463837495	n.s.	
4690	5978938	55	46	0.650952919406731	n.s.	
4690	5978955	55	46	0.650952919406731	n.s.	
4690	5978972	56	45	1.0116974731171	n.s.	
4690	5978975	57	44	1.45165612496859	n.s.	
4690	5978981	58	43	1.97082887496121	n.s.	
4690	5978990	59	42	2.56921572309496	n.s.	
4690	5978993	59	42	2.56921572309496	n.s.	
4690	5979002	60	41	3.24681666936984	n.s.	
4690	5979011	61	40	4.00363171378585	*	
4690	5979018	62	39	4.83966085634298	*	

4690	5979025	61	40	4.00363171378585	*
4690	5979036	60	41	3.24681666936984	n.s.
4690	5979046	60	41	3.24681666936984	n.s.
4690	5979048	61	40	4.00363171378585	*
4690	5979054	61	40	4.00363171378585	*
4690	5979096	62	39	4.83966085634298	*
4690	5979120	63	38	5.75490409704125	*
4690	5979122	63	38	5.75490409704125	*
4690	5979123	63	38	5.75490409704125	*
4690	5979128	63	38	5.75490409704125	*
4690	5979132	63	38	5.75490409704125	*
4690	5979149	63	38	5.75490409704125	*
4690	5979152	63	38	5.75490409704125	*
4690	5979154	63	38	5.75490409704125	*
4690	5979173	63	38	5.75490409704125	*
4690	5979200	63	38	5.75490409704125	*
4690	5979206	62	39	4.83966085634298	*
4690	5979211	63	38	5.75490409704125	*
4690	5979216	62	39	4.83966085634298	*
4690	5979217	62	39	4.83966085634298	*
4690	5979240	62	39	4.83966085634298	*
4690	5979288	63	38	5.75490409704125	*
4690	5979314	62	39	4.83966085634298	*
4690	5979327	62	39	4.83966085634298	*
4690	5979350	63	38	5.75490409704125	*
4690	5979351	63	38	5.75490409704125	*
4690	5979365	63	38	5.75490409704125	*
4690	5979376	63	38	5.75490409704125	*
4690	5979377	63	38	5.75490409704125	*
4690	5979380	64	37	6.74936143588064	**
4690	5979381	65	36	7.82303287286116	**
4690	5979382	64	37	6.74936143588064	**
4690	5979384	65	36	7.82303287286116	**
4690	5979390	65	36	7.82303287286116	**
4690	5979395	66	35	8.97591840798281	**
4690	5979399	66	35	8.97591840798281	**
4690	5979413	67	34	10.2080180412456	**
4690	5979418	66	35	8.97591840798281	**
4690	5979420	65	36	7.82303287286116	**
4690	5979425	66	35	8.97591840798281	**
4690	5979429	67	34	10.2080180412456	**
4690	5979430	68	33	11.5193317726495	***
4690	5979431	68	33	11.5193317726495	***
4690	5979444	68	33	11.5193317726495	***
4690	5979449	67	34	10.2080180412456	**
4690	5979451	66	35	8.97591840798281	**
4690	5979459	66	35	8.97591840798281	**
4690	5979464	66	35	8.97591840798281	**
4690	5979465	67	34	10.2080180412456	**
4690	5979474	67	34	10.2080180412456	**

4690	5979477	67	34	10.2080180412456	**
4690	5979479	68	33	11.5193317726495	***
4690	5979494	68	33	11.5193317726495	***
4690	5979512	68	33	11.5193317726495	***
4690	5979515	68	33	11.5193317726495	***
4690	5979527	68	33	11.5193317726495	***
4690	5979537	69	32	12.9098596021945	***
4690	5979551	68	33	11.5193317726495	***
4690	5979560	68	33	11.5193317726495	***
4690	5979572	67	34	10.2080180412456	**
4690	5979587	67	34	10.2080180412456	**
4690	5979594	67	34	10.2080180412456	**
4690	5979604	68	33	11.5193317726495	***
4690	5979620	68	33	11.5193317726495	***
4690	5979623	68	33	11.5193317726495	***
4690	5979655	68	33	11.5193317726495	***
4690	5979666	69	32	12.9098596021945	***
4690	5979669	70	31	14.3796015298807	***
4690	5979680	70	31	14.3796015298807	***
4690	5979734	70	31	14.3796015298807	***
4690	5979746	69	32	12.9098596021945	***
4690	5979751	68	33	11.5193317726495	***
4690	5979752	68	33	11.5193317726495	***
4690	5979760	68	33	11.5193317726495	***
4690	5979765	67	34	10.2080180412456	**
4690	5979782	66	35	8.97591840798281	**
4690	5979794	66	35	8.97591840798281	**
4690	5979812	66	35	8.97591840798281	**
4690	5979827	65	36	7.82303287286116	**
4690	5979845	65	36	7.82303287286116	**
4690	5979846	65	36	7.82303287286116	**
4690	5979857	64	37	6.74936143588064	**
4690	5979859	63	38	5.75490409704125	*
4690	5979866	63	38	5.75490409704125	*
4690	5979872	63	38	5.75490409704125	*
4690	5979878	62	39	4.83966085634298	*
4690	5979896	62	39	4.83966085634298	*
4690	5979926	62	39	4.83966085634298	*
4690	5979944	61	40	4.00363171378585	*
4690	5979945	60	41	3.24681666936984	n.s.
4690	5979950	59	42	2.56921572309496	n.s.
4690	5979952	59	42	2.56921572309496	n.s.
4690	5979955	58	43	1.97082887496121	n.s.
4690	5979958	57	44	1.45165612496859	n.s.
4690	5979969	56	45	1.0116974731171	n.s.
4690	5979979	56	45	1.0116974731171	n.s.
4690	5980002	55	46	0.650952919406731	n.s.
4690	5980014	55	46	0.650952919406731	n.s.
4690	5980028	54	47	0.369422463837495	n.s.
4690	5980030	53	48	0.167106106409387	n.s.

4690	5980034	52	49	0.044003847122408	n.s.
4690	5980035	53	48	0.167106106409387	n.s.
4690	5980046	53	48	0.167106106409387	n.s.
4690	5980066	52	49	0.044003847122408	n.s.
4690	5980071	52	49	0.044003847122408	n.s.
4690	5980081	52	49	0.044003847122408	n.s.
4690	5980083	52	49	0.044003847122408	n.s.
4690	5980084	52	49	0.044003847122408	n.s.
4690	5980086	52	49	0.044003847122408	n.s.
4690	5980096	52	49	0.044003847122408	n.s.
4690	5980097	52	49	0.044003847122408	n.s.
4690	5980100	53	48	0.167106106409387	n.s.
4690	5980122	53	48	0.167106106409387	n.s.
4690	5980131	53	48	0.167106106409387	n.s.
4690	5980145	54	47	0.369422463837495	n.s.
4690	5980152	55	46	0.650952919406731	n.s.
4690	5980162	54	47	0.369422463837495	n.s.
4690	5980197	54	47	0.369422463837495	n.s.
4690	5980199	54	47	0.369422463837495	n.s.
4690	5980211	55	46	0.650952919406731	n.s.
4690	5980249	55	46	0.650952919406731	n.s.
4690	5980255	56	45	1.0116974731171	n.s.
4690	5980256	56	45	1.0116974731171	n.s.
4690	5980259	57	44	1.45165612496859	n.s.
4690	5980278	57	44	1.45165612496859	n.s.
4690	5980293	57	44	1.45165612496859	n.s.
4690	5980401	58	43	1.97082887496121	n.s.
4690	5980402	59	42	2.56921572309496	n.s.
4690	5980409	60	41	3.24681666936984	n.s.
4690	5980426	61	40	4.00363171378585	*
4690	5980439	61	40	4.00363171378585	*
4690	5980452	60	41	3.24681666936984	n.s.
4690	5980499	61	40	4.00363171378585	*
4690	5980514	61	40	4.00363171378585	*
4690	5980536	60	41	3.24681666936984	n.s.
4690	5980561	59	42	2.56921572309496	n.s.
4690	5980566	59	42	2.56921572309496	n.s.
4690	5980567	59	42	2.56921572309496	n.s.
4690	5980578	60	41	3.24681666936984	n.s.
4690	5980581	61	40	4.00363171378585	*
4690	5980603	61	40	4.00363171378585	*
4690	5980604	61	40	4.00363171378585	*
4690	5980610	61	40	4.00363171378585	*
4690	5980625	60	41	3.24681666936984	n.s.
4690	5980635	60	41	3.24681666936984	n.s.
4690	5980641	61	40	4.00363171378585	*
4690	5980667	62	39	4.83966085634298	*
4690	5980689	62	39	4.83966085634298	*
4690	5980702	62	39	4.83966085634298	*
4690	5980724	62	39	4.83966085634298	*

4690	5980728	62	39	4.83966085634298	*
4690	5980732	62	39	4.83966085634298	*
4690	5980741	61	40	4.00363171378585	*
4690	5980752	60	41	3.24681666936984	n.s.
4690	5980755	60	41	3.24681666936984	n.s.
4690	5980779	60	41	3.24681666936984	n.s.
4690	5980784	60	41	3.24681666936984	n.s.
4690	5980793	59	42	2.56921572309496	n.s.
4690	5980809	60	41	3.24681666936984	n.s.
4690	5980811	60	41	3.24681666936984	n.s.
4690	5980814	60	41	3.24681666936984	n.s.
4690	5980817	59	42	2.56921572309496	n.s.
4690	5980818	59	42	2.56921572309496	n.s.
4690	5980853	59	42	2.56921572309496	n.s.
4690	5980867	59	42	2.56921572309496	n.s.
4690	5980877	59	42	2.56921572309496	n.s.
4690	5980898	60	41	3.24681666936984	n.s.
4690	5980902	59	42	2.56921572309496	n.s.
4690	5980909	59	42	2.56921572309496	n.s.
4690	5980914	59	42	2.56921572309496	n.s.
4690	5980925	59	42	2.56921572309496	n.s.
4690	5980931	60	41	3.24681666936984	n.s.
4690	5980938	60	41	3.24681666936984	n.s.
4690	5980942	61	40	4.00363171378585	*
4690	5980946	62	39	4.83966085634298	*
4690	5980947	63	38	5.75490409704125	*
4690	5980948	64	37	6.74936143588064	**
4690	5980951	64	37	6.74936143588064	**
4690	5980952	64	37	6.74936143588064	**
4690	5980976	65	36	7.82303287286116	**
4690	5980985	65	36	7.82303287286116	**
4690	5980988	64	37	6.74936143588064	**
4690	5981006	65	36	7.82303287286116	**
4690	5981008	66	35	8.97591840798281	**
4690	5981015	65	36	7.82303287286116	**
4690	5981046	65	36	7.82303287286116	**
4690	5981056	66	35	8.97591840798281	**
4690	5981063	65	36	7.82303287286116	**
4690	5981069	66	35	8.97591840798281	**
4690	5981099	67	34	10.2080180412456	**
4690	5981105	68	33	11.5193317726495	***
4690	5981106	69	32	12.9098596021945	***
4690	5981115	69	32	12.9098596021945	***
4690	5981118	70	31	14.3796015298807	***
4690	5981121	71	30	15.928557555708	***
4690	5981122	72	29	17.5567276796764	***
4690	5981127	72	29	17.5567276796764	***
4690	5981130	72	29	17.5567276796764	***
4690	5981140	73	28	19.264111901786	***
4690	5981141	73	28	19.264111901786	***

4690	5981142	74	27	21.0507102220366	***
4690	5981149	74	27	21.0507102220366	***
4690	5981156	74	27	21.0507102220366	***
4690	5981160	74	27	21.0507102220366	***
4690	5981162	75	26	22.9165226404284	***
4690	5981168	74	27	21.0507102220366	***
4690	5981177	73	28	19.264111901786	***
4690	5981179	72	29	17.5567276796764	***
4690	5981185	72	29	17.5567276796764	***
4690	5981200	71	30	15.928557555708	***
4690	5981202	70	31	14.3796015298807	***
4690	5981209	70	31	14.3796015298807	***
4690	5981213	70	31	14.3796015298807	***
4690	5981288	70	31	14.3796015298807	***
4690	5981293	69	32	12.9098596021945	***
4690	5981297	69	32	12.9098596021945	***
4690	5981321	69	32	12.9098596021945	***
4690	5981326	70	31	14.3796015298807	***
4690	5981329	70	31	14.3796015298807	***
4690	5981332	69	32	12.9098596021945	***
4690	5981335	68	33	11.5193317726495	***
4690	5981340	69	32	12.9098596021945	***
4690	5981345	68	33	11.5193317726495	***
4690	5981354	67	34	10.2080180412456	**
4690	5981359	67	34	10.2080180412456	**
4690	5981360	66	35	8.97591840798281	**
4690	5981367	66	35	8.97591840798281	**
4690	5981402	65	36	7.82303287286116	**
4690	5981405	64	37	6.74936143588064	**
4690	5981418	63	38	5.75490409704125	*
4690	5981422	62	39	4.83966085634298	*
4690	5981423	61	40	4.00363171378585	*
4690	5981433	62	39	4.83966085634298	*
4690	5981439	61	40	4.00363171378585	*
4690	5981457	60	41	3.24681666936984	n.s.
4690	5981468	61	40	4.00363171378585	*
4690	5981483	62	39	4.83966085634298	*
4690	5981484	62	39	4.83966085634298	*
4690	5981492	62	39	4.83966085634298	*
4690	5981497	62	39	4.83966085634298	*
4690	5981501	62	39	4.83966085634298	*
4690	5981510	61	40	4.00363171378585	*
4690	5981519	60	41	3.24681666936984	n.s.
4690	5981525	60	41	3.24681666936984	n.s.
4690	5981540	61	40	4.00363171378585	*
4690	5981544	61	40	4.00363171378585	*
4690	5981653	61	40	4.00363171378585	*
4690	5981656	61	40	4.00363171378585	*
4690	5981669	61	40	4.00363171378585	*
4690	5981673	60	41	3.24681666936984	n.s.

4690	5981690	59	42	2.56921572309496	n.s.
4690	5981720	58	43	1.97082887496121	n.s.
4690	5981728	58	43	1.97082887496121	n.s.
4690	5981764	58	43	1.97082887496121	n.s.
4690	5981779	58	43	1.97082887496121	n.s.
4690	5981782	58	43	1.97082887496121	n.s.
4690	5981786	59	42	2.56921572309496	n.s.
4690	5981794	58	43	1.97082887496121	n.s.
4690	5981796	59	42	2.56921572309496	n.s.
4690	5981802	58	43	1.97082887496121	n.s.
4690	5981813	59	42	2.56921572309496	n.s.
4690	5981823	59	42	2.56921572309496	n.s.
4690	5981826	60	41	3.24681666936984	n.s.
4690	5981847	59	42	2.56921572309496	n.s.
4690	5981848	58	43	1.97082887496121	n.s.
4690	5981876	57	44	1.45165612496859	n.s.
4690	5981892	56	45	1.0116974731171	n.s.
4690	5981936	56	45	1.0116974731171	n.s.
4690	5981956	57	44	1.45165612496859	n.s.
4690	5981957	57	44	1.45165612496859	n.s.
4690	5981971	56	45	1.0116974731171	n.s.
4690	5981975	55	46	0.650952919406731	n.s.
4690	5981978	54	47	0.369422463837495	n.s.
4690	5981987	54	47	0.369422463837495	n.s.
4690	5982019	53	48	0.167106106409387	n.s.
4690	5982023	52	49	0.044003847122408	n.s.
4690	5982077	52	49	0.044003847122408	n.s.
4690	5982083	52	49	0.044003847122408	n.s.
4690	5982147	52	49	0.044003847122408	n.s.
4690	5982148	51	50	0.000115685976557532	n.s.
4690	5982158	51	50	0.000115685976557532	n.s.
4690	5982163	50	51	0.0354416229718356	n.s.
4690	5982164	50	51	0.0354416229718356	n.s.
4690	5982178	50	51	0.0354416229718356	n.s.
4690	5982195	50	51	0.0354416229718356	n.s.
4690	5982216	50	51	0.0354416229718356	n.s.
4690	5982228	50	51	0.0354416229718356	n.s.
4690	5982237	49	52	0.149981658108242	n.s.
4690	5982260	50	51	0.0354416229718356	n.s.
4690	5982277	49	52	0.149981658108242	n.s.
4690	5982288	48	53	0.343735791385777	n.s.
4690	5982296	48	53	0.343735791385777	n.s.
4690	5982305	47	54	0.616704022804441	n.s.
4690	5982322	46	55	0.968886352364233	n.s.
4690	5982355	46	55	0.968886352364233	n.s.
4690	5982402	45	56	1.40028278006515	n.s.
4690	5982405	44	57	1.9108933059072	n.s.
4690	5982409	43	58	2.50071792989038	n.s.
4690	5982415	43	58	2.50071792989038	n.s.
4690	5982451	42	59	3.16975665201469	n.s.

4690	5982454	42	59	3.16975665201469	n.s.
4690	5982456	41	60	3.91800947228012	*
4690	5982465	42	59	3.16975665201469	n.s.
4690	5982471	41	60	3.91800947228012	*
4690	5982483	41	60	3.91800947228012	*
4690	5982491	40	61	4.74547639068669	*
4690	5982497	40	61	4.74547639068669	*
4690	5982509	41	60	3.91800947228012	*
4690	5982515	42	59	3.16975665201469	n.s.
4690	5982527	42	59	3.16975665201469	n.s.
4690	5982542	43	58	2.50071792989038	n.s.
4690	5982545	43	58	2.50071792989038	n.s.
4690	5982552	42	59	3.16975665201469	n.s.
4690	5982563	42	59	3.16975665201469	n.s.
4690	5982570	42	59	3.16975665201469	n.s.
4690	5982585	42	59	3.16975665201469	n.s.
4690	5982591	42	59	3.16975665201469	n.s.
4690	5982598	42	59	3.16975665201469	n.s.
4690	5982632	41	60	3.91800947228012	*
4690	5982645	42	59	3.16975665201469	n.s.
4690	5982701	42	59	3.16975665201469	n.s.
4690	5982703	42	59	3.16975665201469	n.s.
4690	5982711	42	59	3.16975665201469	n.s.
4690	5982725	43	58	2.50071792989038	n.s.
4690	5982751	44	57	1.9108933059072	n.s.
4690	5982752	44	57	1.9108933059072	n.s.
4690	5982764	44	57	1.9108933059072	n.s.
4690	5982779	43	58	2.50071792989038	n.s.
4690	5982785	43	58	2.50071792989038	n.s.
4690	5982796	43	58	2.50071792989038	n.s.
4690	5982808	44	57	1.9108933059072	n.s.
4690	5982821	45	56	1.40028278006515	n.s.
4690	5982850	45	56	1.40028278006515	n.s.
4690	5982852	44	57	1.9108933059072	n.s.
4690	5982887	45	56	1.40028278006515	n.s.
4690	5982894	46	55	0.968886352364233	n.s.
4690	5982903	45	56	1.40028278006515	n.s.
4690	5982938	45	56	1.40028278006515	n.s.
4690	5982944	46	55	0.968886352364233	n.s.
4690	5982956	46	55	0.968886352364233	n.s.
4690	5982976	46	55	0.968886352364233	n.s.
4690	5982978	46	55	0.968886352364233	n.s.
4690	5982995	47	54	0.616704022804441	n.s.
4690	5983007	47	54	0.616704022804441	n.s.
4690	5983010	47	54	0.616704022804441	n.s.
4690	5983019	47	54	0.616704022804441	n.s.
4690	5983028	47	54	0.616704022804441	n.s.
4690	5983033	47	54	0.616704022804441	n.s.
4690	5983056	47	54	0.616704022804441	n.s.
4690	5983067	47	54	0.616704022804441	n.s.

4690	5983068	47	54	0.616704022804441	n.s.	
4690	5983071	48	53	0.343735791385777	n.s.	
4690	5983076	49	52	0.149981658108242	n.s.	
4690	5983079	50	51	0.0354416229718356	n.s.	
4690	5983095	51	50	0.000115685976557532		n.s.
4690	5983100	52	49	0.044003847122408	n.s.	
4690	5983130	53	48	0.167106106409387	n.s.	
4690	5983134	53	48	0.167106106409387	n.s.	
4690	5983137	53	48	0.167106106409387	n.s.	
4690	5983142	52	49	0.044003847122408	n.s.	
4690	5983167	53	48	0.167106106409387	n.s.	
4690	5983178	53	48	0.167106106409387	n.s.	
4690	5983180	54	47	0.369422463837495	n.s.	
4690	5983182	53	48	0.167106106409387	n.s.	
4690	5983215	54	47	0.369422463837495	n.s.	
4690	5983217	55	46	0.650952919406731	n.s.	
4690	5983221	55	46	0.650952919406731	n.s.	
4690	5983225	56	45	1.0116974731171	n.s.	
4690	5983227	55	46	0.650952919406731	n.s.	
4690	5983233	55	46	0.650952919406731	n.s.	
4690	5983245	55	46	0.650952919406731	n.s.	
4690	5983255	56	45	1.0116974731171	n.s.	
4690	5983277	57	44	1.45165612496859	n.s.	
4690	5983313	58	43	1.97082887496121	n.s.	
4690	5983352	59	42	2.56921572309496	n.s.	
4690	5983367	59	42	2.56921572309496	n.s.	
4690	5983371	60	41	3.24681666936984	n.s.	
4690	5983394	60	41	3.24681666936984	n.s.	
4690	5983400	60	41	3.24681666936984	n.s.	
4690	5983404	60	41	3.24681666936984	n.s.	
4690	5983405	61	40	4.00363171378585	*	
4690	5983408	61	40	4.00363171378585	*	
4690	5983416	61	40	4.00363171378585	*	
4690	5983439	61	40	4.00363171378585	*	
4690	5983468	60	41	3.24681666936984	n.s.	
4690	5983485	59	42	2.56921572309496	n.s.	
4690	5983500	60	41	3.24681666936984	n.s.	
4690	5983504	59	42	2.56921572309496	n.s.	
4690	5983511	59	42	2.56921572309496	n.s.	
4690	5983512	58	43	1.97082887496121	n.s.	
4690	5983516	58	43	1.97082887496121	n.s.	
4690	5983539	58	43	1.97082887496121	n.s.	
4690	5983545	58	43	1.97082887496121	n.s.	
4690	5983562	58	43	1.97082887496121	n.s.	
4690	5983574	58	43	1.97082887496121	n.s.	
4690	5983577	57	44	1.45165612496859	n.s.	
4690	5983589	58	43	1.97082887496121	n.s.	
4690	5983591	59	42	2.56921572309496	n.s.	
4690	5983597	59	42	2.56921572309496	n.s.	
4690	5983602	59	42	2.56921572309496	n.s.	

4690	5983607	59	42	2.56921572309496	n.s.
4690	5983619	60	41	3.24681666936984	n.s.
4690	5983634	60	41	3.24681666936984	n.s.
4690	5983637	60	41	3.24681666936984	n.s.
4690	5983646	61	40	4.00363171378585	*
4690	5983648	62	39	4.83966085634298	*
4690	5983655	62	39	4.83966085634298	*
4690	5983661	63	38	5.75490409704125	*
4690	5983663	63	38	5.75490409704125	*
4690	5983679	62	39	4.83966085634298	*
4690	5983696	62	39	4.83966085634298	*
4690	5983713	61	40	4.00363171378585	*
4690	5983717	62	39	4.83966085634298	*
4690	5983738	63	38	5.75490409704125	*
4690	5983742	63	38	5.75490409704125	*
4690	5983748	62	39	4.83966085634298	*
4690	5983763	62	39	4.83966085634298	*
4690	5983774	62	39	4.83966085634298	*
4690	5983787	61	40	4.00363171378585	*
4690	5983796	61	40	4.00363171378585	*
4690	5983814	60	41	3.24681666936984	n.s.
4690	5983830	61	40	4.00363171378585	*
4690	5983836	61	40	4.00363171378585	*
4690	5983838	60	41	3.24681666936984	n.s.
4690	5983841	59	42	2.56921572309496	n.s.
4690	5983846	59	42	2.56921572309496	n.s.
4690	5983852	58	43	1.97082887496121	n.s.
4690	5983854	59	42	2.56921572309496	n.s.
4690	5983859	59	42	2.56921572309496	n.s.
4690	5983868	60	41	3.24681666936984	n.s.
4690	5983884	61	40	4.00363171378585	*
4690	5983908	60	41	3.24681666936984	n.s.
4690	5983911	60	41	3.24681666936984	n.s.
4690	5983912	61	40	4.00363171378585	*
4690	5983934	62	39	4.83966085634298	*
4690	5983936	62	39	4.83966085634298	*
4690	5983941	62	39	4.83966085634298	*
4690	5983943	63	38	5.75490409704125	*
4690	5983961	62	39	4.83966085634298	*
4690	5983962	62	39	4.83966085634298	*
4690	5983967	62	39	4.83966085634298	*
4690	5983971	62	39	4.83966085634298	*
4690	5983988	62	39	4.83966085634298	*
4690	5983993	62	39	4.83966085634298	*
4690	5984005	63	38	5.75490409704125	*
4690	5984012	62	39	4.83966085634298	*
4690	5984017	62	39	4.83966085634298	*
4690	5984043	62	39	4.83966085634298	*
4690	5984044	61	40	4.00363171378585	*
4690	5984054	60	41	3.24681666936984	n.s.

4690	5984065	60	41	3.24681666936984	n.s.
4690	5984075	60	41	3.24681666936984	n.s.
4690	5984091	59	42	2.56921572309496	n.s.
4690	5984115	58	43	1.97082887496121	n.s.
4690	5984126	57	44	1.45165612496859	n.s.
4690	5984140	57	44	1.45165612496859	n.s.
4690	5984158	56	45	1.0116974731171	n.s.
4690	5984178	56	45	1.0116974731171	n.s.
4690	5984191	55	46	0.650952919406731	n.s.
4690	5984195	56	45	1.0116974731171	n.s.
4690	5984197	57	44	1.45165612496859	n.s.
4690	5984208	56	45	1.0116974731171	n.s.
4690	5984209	56	45	1.0116974731171	n.s.
4690	5984214	55	46	0.650952919406731	n.s.
4690	5984219	56	45	1.0116974731171	n.s.
4690	5984241	56	45	1.0116974731171	n.s.
4690	5984242	56	45	1.0116974731171	n.s.
4690	5984245	57	44	1.45165612496859	n.s.
4690	5984264	57	44	1.45165612496859	n.s.
4690	5984269	58	43	1.97082887496121	n.s.
4690	5984270	57	44	1.45165612496859	n.s.
4690	5984282	56	45	1.0116974731171	n.s.
4690	5984284	55	46	0.650952919406731	n.s.
4690	5984304	54	47	0.369422463837495	n.s.
4690	5984358	54	47	0.369422463837495	n.s.
4690	5984392	53	48	0.167106106409387	n.s.
4690	5984398	53	48	0.167106106409387	n.s.
4690	5984425	52	49	0.044003847122408	n.s.
4690	5984426	52	49	0.044003847122408	n.s.
4690	5984443	51	50	0.000115685976557532	n.s.
4690	5984488	50	51	0.0354416229718356	n.s.
4690	5984503	49	52	0.149981658108242	n.s.
4690	5984505	49	52	0.149981658108242	n.s.
4690	5984540	49	52	0.149981658108242	n.s.
4690	5984580	49	52	0.149981658108242	n.s.
4690	5984584	49	52	0.149981658108242	n.s.
4690	5984588	49	52	0.149981658108242	n.s.
4690	5984592	49	52	0.149981658108242	n.s.
4690	5984593	49	52	0.149981658108242	n.s.
4690	5984594	49	52	0.149981658108242	n.s.
4690	5984614	49	52	0.149981658108242	n.s.
4690	5984618	49	52	0.149981658108242	n.s.
4690	5984620	49	52	0.149981658108242	n.s.
4690	5984621	48	53	0.343735791385777	n.s.
4690	5984667	48	53	0.343735791385777	n.s.
4690	5984701	48	53	0.343735791385777	n.s.
4690	5984740	48	53	0.343735791385777	n.s.
4690	5984749	48	53	0.343735791385777	n.s.
4690	5984780	48	53	0.343735791385777	n.s.
4690	5984822	49	52	0.149981658108242	n.s.

4690	5984840	49	52	0.149981658108242	n.s.	
4690	5984842	50	51	0.0354416229718356	n.s.	
4690	5984844	50	51	0.0354416229718356	n.s.	
4690	5984856	50	51	0.0354416229718356	n.s.	
4690	5984859	49	52	0.149981658108242	n.s.	
4690	5984888	49	52	0.149981658108242	n.s.	
4690	5984950	49	52	0.149981658108242	n.s.	
4690	5984951	50	51	0.0354416229718356	n.s.	
4690	5984954	49	52	0.149981658108242	n.s.	
4690	5984956	49	52	0.149981658108242	n.s.	
4690	5984996	50	51	0.0354416229718356	n.s.	
4690	5984999	49	52	0.149981658108242	n.s.	
4690	5985019	49	52	0.149981658108242	n.s.	
4690	5985048	49	52	0.149981658108242	n.s.	
4690	5985069	49	52	0.149981658108242	n.s.	
4690	5985073	49	52	0.149981658108242	n.s.	
4690	5985101	50	51	0.0354416229718356	n.s.	
4690	5985103	51	50	0.000115685976557532		n.s.
4690	5985137	51	50	0.000115685976557532		n.s.
4690	5985154	52	49	0.044003847122408	n.s.	
4690	5985157	53	48	0.167106106409387	n.s.	
4690	5985166	54	47	0.369422463837495	n.s.	
4690	5985188	54	47	0.369422463837495	n.s.	
4690	5985189	55	46	0.650952919406731	n.s.	
4690	5985193	56	45	1.0116974731171	n.s.	
4690	5985206	57	44	1.45165612496859	n.s.	
4690	5985216	57	44	1.45165612496859	n.s.	
4690	5985232	58	43	1.97082887496121	n.s.	
4690	5985252	58	43	1.97082887496121	n.s.	
4690	5985266	59	42	2.56921572309496	n.s.	
4690	5985267	59	42	2.56921572309496	n.s.	
4690	5985288	59	42	2.56921572309496	n.s.	
4690	5985311	60	41	3.24681666936984	n.s.	
4690	5985324	60	41	3.24681666936984	n.s.	
4690	5985347	60	41	3.24681666936984	n.s.	
4690	5985373	60	41	3.24681666936984	n.s.	
4690	5985390	59	42	2.56921572309496	n.s.	
4690	5985430	59	42	2.56921572309496	n.s.	
4690	5985437	59	42	2.56921572309496	n.s.	
4690	5985456	59	42	2.56921572309496	n.s.	
4690	5985458	58	43	1.97082887496121	n.s.	
4690	5985522	57	44	1.45165612496859	n.s.	
4690	5985530	57	44	1.45165612496859	n.s.	
4690	5985557	56	45	1.0116974731171	n.s.	
4690	5985570	56	45	1.0116974731171	n.s.	
4690	5985586	56	45	1.0116974731171	n.s.	
4690	5985596	56	45	1.0116974731171	n.s.	
4690	5985601	56	45	1.0116974731171	n.s.	
4690	5985607	56	45	1.0116974731171	n.s.	
4690	5985615	56	45	1.0116974731171	n.s.	

4690	5985638	57	44	1.45165612496859	n.s.
4690	5985639	56	45	1.0116974731171	n.s.
4690	5985640	56	45	1.0116974731171	n.s.
4690	5985645	57	44	1.45165612496859	n.s.
4690	5985646	57	44	1.45165612496859	n.s.
4690	5985647	57	44	1.45165612496859	n.s.
4690	5985655	56	45	1.0116974731171	n.s.
4690	5985694	57	44	1.45165612496859	n.s.
4690	5985698	57	44	1.45165612496859	n.s.
4690	5985701	58	43	1.97082887496121	n.s.
4690	5985705	58	43	1.97082887496121	n.s.
4690	5985707	58	43	1.97082887496121	n.s.
4690	5985712	58	43	1.97082887496121	n.s.
4690	5985716	58	43	1.97082887496121	n.s.
4690	5985719	58	43	1.97082887496121	n.s.
4690	5985731	57	44	1.45165612496859	n.s.
4690	5985732	56	45	1.0116974731171	n.s.
4690	5985747	55	46	0.650952919406731	n.s.
4690	5985748	55	46	0.650952919406731	n.s.
4690	5985752	54	47	0.369422463837495	n.s.
4690	5985760	54	47	0.369422463837495	n.s.
4690	5985761	55	46	0.650952919406731	n.s.
4690	5985763	55	46	0.650952919406731	n.s.
4690	5985764	56	45	1.0116974731171	n.s.
4690	5985765	56	45	1.0116974731171	n.s.
4690	5985767	56	45	1.0116974731171	n.s.
4690	5985769	57	44	1.45165612496859	n.s.
4690	5985775	57	44	1.45165612496859	n.s.
4690	5985776	58	43	1.97082887496121	n.s.
4690	5985777	58	43	1.97082887496121	n.s.
4690	5985778	58	43	1.97082887496121	n.s.
4690	5985872	58	43	1.97082887496121	n.s.
4690	5985885	59	42	2.56921572309496	n.s.
4690	5985898	59	42	2.56921572309496	n.s.
4690	5985914	59	42	2.56921572309496	n.s.
4690	5985916	60	41	3.24681666936984	n.s.
4690	5987179	60	41	3.24681666936984	n.s.
4690	5987183	61	40	4.00363171378585	*
4690	5987185	60	41	3.24681666936984	n.s.
4690	5987186	60	41	3.24681666936984	n.s.
4690	5987199	61	40	4.00363171378585	*
4690	5987234	61	40	4.00363171378585	*
4690	5987256	61	40	4.00363171378585	*
4690	5987320	62	39	4.83966085634298	*
4690	5987325	63	38	5.75490409704125	*
4690	5987383	63	38	5.75490409704125	*
4690	5987395	64	37	6.74936143588064	**
4690	5987402	65	36	7.82303287286116	**
4690	5987435	65	36	7.82303287286116	**
4690	5987463	65	36	7.82303287286116	**

4690	5987467	65	36	7.82303287286116	**
4690	5987480	66	35	8.97591840798281	**
4690	5987481	65	36	7.82303287286116	**
4690	5987487	65	36	7.82303287286116	**
4690	5987498	65	36	7.82303287286116	**
4690	5987514	66	35	8.97591840798281	**
4690	5987519	65	36	7.82303287286116	**
4690	5987520	64	37	6.74936143588064	**
4690	5987524	64	37	6.74936143588064	**
4690	5987562	65	36	7.82303287286116	**
4690	5987570	64	37	6.74936143588064	**
4690	5987579	63	38	5.75490409704125	*
4690	5987585	64	37	6.74936143588064	**
4690	5987621	65	36	7.82303287286116	**
4690	5987630	64	37	6.74936143588064	**
4690	5987657	64	37	6.74936143588064	**
4690	5987669	64	37	6.74936143588064	**
4690	5987679	64	37	6.74936143588064	**
4690	5987685	63	38	5.75490409704125	*
4690	5987690	63	38	5.75490409704125	*
4690	5987702	62	39	4.83966085634298	*
4690	5987705	61	40	4.00363171378585	*
4690	5987718	61	40	4.00363171378585	*
4690	5987723	61	40	4.00363171378585	*
4690	5987731	61	40	4.00363171378585	*
4690	5987732	61	40	4.00363171378585	*
4690	5987766	60	41	3.24681666936984	n.s.
4690	5987767	59	42	2.56921572309496	n.s.
4690	5987781	59	42	2.56921572309496	n.s.
4690	5987795	59	42	2.56921572309496	n.s.
4690	5987797	58	43	1.97082887496121	n.s.
4690	5987802	58	43	1.97082887496121	n.s.
4690	5987805	57	44	1.45165612496859	n.s.
4690	5987812	57	44	1.45165612496859	n.s.
4690	5987822	57	44	1.45165612496859	n.s.
4690	5987823	56	45	1.0116974731171	n.s.
4690	5987879	56	45	1.0116974731171	n.s.
4690	5987881	56	45	1.0116974731171	n.s.
4690	5987936	56	45	1.0116974731171	n.s.
4690	5987959	55	46	0.650952919406731	n.s.
4690	5987964	56	45	1.0116974731171	n.s.
4690	5987967	57	44	1.45165612496859	n.s.
4690	5987975	57	44	1.45165612496859	n.s.
4690	5987976	57	44	1.45165612496859	n.s.
4690	5987983	58	43	1.97082887496121	n.s.
4690	5987986	58	43	1.97082887496121	n.s.
4690	5987989	57	44	1.45165612496859	n.s.
4690	5987997	58	43	1.97082887496121	n.s.
4690	5988000	57	44	1.45165612496859	n.s.
4690	5988017	56	45	1.0116974731171	n.s.

4690	5988018	57	44	1.45165612496859	n.s.
4690	5988037	57	44	1.45165612496859	n.s.
4690	5988054	58	43	1.97082887496121	n.s.
4690	5988067	58	43	1.97082887496121	n.s.
4690	5988072	57	44	1.45165612496859	n.s.
4690	5988080	58	43	1.97082887496121	n.s.
4690	5988083	59	42	2.56921572309496	n.s.
4690	5988109	60	41	3.24681666936984	n.s.
4690	5988132	60	41	3.24681666936984	n.s.
4690	5988145	60	41	3.24681666936984	n.s.
4690	5988170	59	42	2.56921572309496	n.s.
4690	5988175	59	42	2.56921572309496	n.s.
4690	5988181	59	42	2.56921572309496	n.s.
4690	5988184	60	41	3.24681666936984	n.s.
4690	5988202	60	41	3.24681666936984	n.s.
4690	5988205	61	40	4.00363171378585	*
4690	5988216	61	40	4.00363171378585	*
4690	5988220	61	40	4.00363171378585	*
4690	5988229	62	39	4.83966085634298	*
4690	5988239	61	40	4.00363171378585	*
4690	5988240	62	39	4.83966085634298	*
4690	5988247	62	39	4.83966085634298	*
4690	5988268	62	39	4.83966085634298	*
4690	5988283	63	38	5.75490409704125	*
4690	5988286	63	38	5.75490409704125	*
4690	5988296	64	37	6.74936143588064	**
4690	5988304	64	37	6.74936143588064	**
4690	5988308	64	37	6.74936143588064	**
4690	5988312	64	37	6.74936143588064	**
4690	5988336	64	37	6.74936143588064	**
4690	5988343	63	38	5.75490409704125	*
4690	5988354	64	37	6.74936143588064	**
4690	5988382	64	37	6.74936143588064	**
4690	5988390	64	37	6.74936143588064	**
4690	5988411	64	37	6.74936143588064	**
4690	5988412	64	37	6.74936143588064	**
4690	5988413	64	37	6.74936143588064	**
4690	5988454	64	37	6.74936143588064	**
4690	5988474	64	37	6.74936143588064	**
4690	5988484	65	36	7.82303287286116	**
4690	5988497	65	36	7.82303287286116	**
4690	5988502	65	36	7.82303287286116	**
4690	5988508	66	35	8.97591840798281	**
4690	5988636	67	34	10.2080180412456	**
4690	5988648	66	35	8.97591840798281	**
4690	5988655	66	35	8.97591840798281	**
4690	5988661	67	34	10.2080180412456	**
4690	5988673	67	34	10.2080180412456	**
4690	5988688	67	34	10.2080180412456	**
4690	5988697	67	34	10.2080180412456	**

4690	5988718	66	35	8.97591840798281	**
4690	5988724	66	35	8.97591840798281	**
4690	5988831	65	36	7.82303287286116	**
4690	5988845	66	35	8.97591840798281	**
4690	5988865	65	36	7.82303287286116	**
4690	5988898	66	35	8.97591840798281	**
4690	5988906	66	35	8.97591840798281	**
4690	5988912	67	34	10.2080180412456	**
4690	5988914	68	33	11.5193317726495	***
4690	5988915	68	33	11.5193317726495	***
4690	5988943	68	33	11.5193317726495	***
4690	5988961	69	32	12.9098596021945	***
4690	5988983	69	32	12.9098596021945	***
4690	5989000	68	33	11.5193317726495	***
4690	5989033	68	33	11.5193317726495	***
4690	5989077	69	32	12.9098596021945	***
4690	5989097	69	32	12.9098596021945	***
4690	5989098	68	33	11.5193317726495	***
4690	5989132	68	33	11.5193317726495	***
4690	5989144	69	32	12.9098596021945	***
4690	5989161	68	33	11.5193317726495	***
4690	5989181	68	33	11.5193317726495	***
4690	5989186	69	32	12.9098596021945	***
4690	5989190	68	33	11.5193317726495	***
4690	5989196	67	34	10.2080180412456	**
4690	5989212	66	35	8.97591840798281	**
4690	5989231	65	36	7.82303287286116	**
4690	5989234	66	35	8.97591840798281	**
4690	5989252	66	35	8.97591840798281	**
4690	5989258	66	35	8.97591840798281	**
4690	5989261	66	35	8.97591840798281	**
4690	5989285	67	34	10.2080180412456	**
4690	5989294	66	35	8.97591840798281	**
4690	5989306	66	35	8.97591840798281	**
4690	5989318	65	36	7.82303287286116	**
4690	5989328	65	36	7.82303287286116	**
4690	5989330	65	36	7.82303287286116	**
4690	5989348	64	37	6.74936143588064	**
4690	5989357	64	37	6.74936143588064	**
4690	5989360	63	38	5.75490409704125	*
4690	5989369	63	38	5.75490409704125	*
4690	5989378	63	38	5.75490409704125	*
4690	5989400	63	38	5.75490409704125	*
4690	5989402	63	38	5.75490409704125	*
4690	5989408	62	39	4.83966085634298	*
4690	5989414	62	39	4.83966085634298	*
4690	5989415	61	40	4.00363171378585	*
4690	5989417	62	39	4.83966085634298	*
4690	5989420	61	40	4.00363171378585	*
4690	5989429	61	40	4.00363171378585	*

4690	5989444	62	39	4.83966085634298	*
4690	5989456	61	40	4.00363171378585	*
4690	5989459	60	41	3.24681666936984	n.s.
4690	5989463	60	41	3.24681666936984	n.s.
4690	5989471	61	40	4.00363171378585	*
4690	5989484	61	40	4.00363171378585	*
4690	5989486	61	40	4.00363171378585	*
4690	5989489	60	41	3.24681666936984	n.s.
4690	5989491	59	42	2.56921572309496	n.s.
4690	5989492	58	43	1.97082887496121	n.s.
4690	5989515	58	43	1.97082887496121	n.s.
4690	5989522	59	42	2.56921572309496	n.s.
4690	5989523	58	43	1.97082887496121	n.s.
4690	5989525	57	44	1.45165612496859	n.s.
4690	5989531	56	45	1.0116974731171	n.s.
4690	5989585	55	46	0.650952919406731	n.s.
4690	5989610	55	46	0.650952919406731	n.s.
4690	5989640	56	45	1.0116974731171	n.s.
4690	5989642	57	44	1.45165612496859	n.s.
4690	5989658	57	44	1.45165612496859	n.s.
4690	5989660	57	44	1.45165612496859	n.s.
4690	5989682	57	44	1.45165612496859	n.s.
4690	5989692	57	44	1.45165612496859	n.s.
4690	5989708	57	44	1.45165612496859	n.s.
4690	5989711	57	44	1.45165612496859	n.s.
4690	5989747	56	45	1.0116974731171	n.s.
4690	5989770	55	46	0.650952919406731	n.s.
4690	5989773	55	46	0.650952919406731	n.s.
4690	5989779	54	47	0.369422463837495	n.s.
4690	5989815	55	46	0.650952919406731	n.s.
4690	5989823	54	47	0.369422463837495	n.s.
4690	5989824	55	46	0.650952919406731	n.s.
4690	5989836	55	46	0.650952919406731	n.s.
4690	5989872	55	46	0.650952919406731	n.s.
4690	5989873	55	46	0.650952919406731	n.s.
4690	5989878	55	46	0.650952919406731	n.s.
4690	5989888	56	45	1.0116974731171	n.s.
4690	5989891	55	46	0.650952919406731	n.s.
4690	5989908	56	45	1.0116974731171	n.s.
4690	5989918	56	45	1.0116974731171	n.s.
4690	5989923	56	45	1.0116974731171	n.s.
4690	5989927	57	44	1.45165612496859	n.s.
4690	5989944	57	44	1.45165612496859	n.s.
4690	5989952	57	44	1.45165612496859	n.s.
4690	5989964	57	44	1.45165612496859	n.s.
4690	5989998	57	44	1.45165612496859	n.s.
4690	5990004	57	44	1.45165612496859	n.s.
4690	5990008	57	44	1.45165612496859	n.s.
4690	5990032	57	44	1.45165612496859	n.s.
4690	5990034	57	44	1.45165612496859	n.s.

4690	5990035	57	44	1.45165612496859	n.s.
4690	5990040	58	43	1.97082887496121	n.s.
4690	5990053	58	43	1.97082887496121	n.s.
4690	5990056	59	42	2.56921572309496	n.s.
4690	5990081	58	43	1.97082887496121	n.s.
4690	5990094	59	42	2.56921572309496	n.s.
4690	5990108	59	42	2.56921572309496	n.s.
4690	5990110	59	42	2.56921572309496	n.s.
4690	5990129	59	42	2.56921572309496	n.s.
4690	5990137	59	42	2.56921572309496	n.s.
4690	5990165	59	42	2.56921572309496	n.s.
4690	5990167	59	42	2.56921572309496	n.s.
4690	5990175	59	42	2.56921572309496	n.s.
4690	5990176	60	41	3.24681666936984	n.s.
4690	5990186	60	41	3.24681666936984	n.s.
4690	5990195	59	42	2.56921572309496	n.s.
4690	5990196	58	43	1.97082887496121	n.s.
4690	5990197	58	43	1.97082887496121	n.s.
4690	5990202	58	43	1.97082887496121	n.s.
4690	5990203	58	43	1.97082887496121	n.s.
4690	5990242	57	44	1.45165612496859	n.s.
4690	5990268	57	44	1.45165612496859	n.s.
4690	5990303	57	44	1.45165612496859	n.s.
4690	5990317	57	44	1.45165612496859	n.s.
4690	5990318	57	44	1.45165612496859	n.s.
4690	5990340	58	43	1.97082887496121	n.s.
4690	5990342	59	42	2.56921572309496	n.s.
4690	5990345	60	41	3.24681666936984	n.s.
4690	5990363	60	41	3.24681666936984	n.s.
4690	5990369	61	40	4.00363171378585	*
4690	5990383	61	40	4.00363171378585	*
4690	5990387	61	40	4.00363171378585	*
4690	5990388	61	40	4.00363171378585	*
4690	5990417	62	39	4.83966085634298	*
4690	5990446	62	39	4.83966085634298	*
4690	5990462	63	38	5.75490409704125	*
4690	5990486	62	39	4.83966085634298	*
4690	5990492	62	39	4.83966085634298	*
4690	5990521	63	38	5.75490409704125	*
4690	5990523	63	38	5.75490409704125	*
4690	5990530	64	37	6.74936143588064	**
4690	5990537	64	37	6.74936143588064	**
4690	5990542	64	37	6.74936143588064	**
4690	5990545	64	37	6.74936143588064	**
4690	5990547	65	36	7.82303287286116	**
4690	5990560	66	35	8.97591840798281	**
4690	5990561	65	36	7.82303287286116	**
4690	5990562	65	36	7.82303287286116	**
4690	5990563	65	36	7.82303287286116	**
4690	5990566	65	36	7.82303287286116	**

4690	5990575	66	35	8.97591840798281	**
4690	5990579	65	36	7.82303287286116	**
4690	5990584	65	36	7.82303287286116	**
4690	5990589	65	36	7.82303287286116	**
4690	5990595	64	37	6.74936143588064	**
4690	5990598	64	37	6.74936143588064	**
4690	5990634	64	37	6.74936143588064	**
4690	5990643	63	38	5.75490409704125	*
4690	5990646	63	38	5.75490409704125	*
4690	5990648	63	38	5.75490409704125	*
4690	5990655	63	38	5.75490409704125	*
4690	5990664	62	39	4.83966085634298	*
4690	5990665	61	40	4.00363171378585	*
4690	5990681	62	39	4.83966085634298	*
4690	5990683	63	38	5.75490409704125	*
4690	5990703	63	38	5.75490409704125	*
4690	5990724	64	37	6.74936143588064	**
4690	5990726	64	37	6.74936143588064	**
4690	5990727	64	37	6.74936143588064	**
4690	5990733	63	38	5.75490409704125	*
4690	5990738	62	39	4.83966085634298	*
4690	5990742	63	38	5.75490409704125	*
4690	5990773	63	38	5.75490409704125	*
4690	5990783	62	39	4.83966085634298	*
4690	5990800	61	40	4.00363171378585	*
4690	5990802	61	40	4.00363171378585	*
4690	5990803	62	39	4.83966085634298	*
4690	5990811	62	39	4.83966085634298	*
4690	5990833	62	39	4.83966085634298	*
4690	5990834	63	38	5.75490409704125	*
4690	5990854	63	38	5.75490409704125	*
4690	5990860	64	37	6.74936143588064	**
4690	5990888	63	38	5.75490409704125	*
4690	5990934	62	39	4.83966085634298	*
4690	5990935	62	39	4.83966085634298	*
4690	5990941	61	40	4.00363171378585	*
4690	5990954	60	41	3.24681666936984	n.s.
4690	5990960	60	41	3.24681666936984	n.s.
4690	5990966	60	41	3.24681666936984	n.s.
4690	5991010	59	42	2.56921572309496	n.s.
4690	5991030	58	43	1.97082887496121	n.s.
4690	5991032	58	43	1.97082887496121	n.s.
4690	5991054	58	43	1.97082887496121	n.s.
4690	5991077	57	44	1.45165612496859	n.s.
4690	5991086	56	45	1.0116974731171	n.s.
4690	5991104	55	46	0.650952919406731	n.s.
4690	5991107	55	46	0.650952919406731	n.s.
4690	5991122	54	47	0.369422463837495	n.s.
4690	5991125	54	47	0.369422463837495	n.s.
4690	5991140	53	48	0.167106106409387	n.s.

4690	5991166	53	48	0.167106106409387	n.s.
4690	5991170	52	49	0.044003847122408	n.s.
4690	5991171	52	49	0.044003847122408	n.s.
4690	5991212	51	50	0.000115685976557532	n.s.
4690	5991237	50	51	0.0354416229718356	n.s.
4690	5991241	50	51	0.0354416229718356	n.s.
4690	5991259	49	52	0.149981658108242	n.s.
4690	5991265	49	52	0.149981658108242	n.s.
4690	5991277	48	53	0.343735791385777	n.s.
4690	5991299	47	54	0.616704022804441	n.s.
4690	5991320	47	54	0.616704022804441	n.s.
4690	5991322	46	55	0.968886352364233	n.s.
4690	5991371	46	55	0.968886352364233	n.s.
4690	5991377	46	55	0.968886352364233	n.s.
4690	5991382	46	55	0.968886352364233	n.s.
4690	5991383	47	54	0.616704022804441	n.s.
4690	5991387	48	53	0.343735791385777	n.s.
4690	5991392	49	52	0.149981658108242	n.s.
4690	5991397	49	52	0.149981658108242	n.s.
4690	5991398	50	51	0.0354416229718356	n.s.
4690	5991413	49	52	0.149981658108242	n.s.
4690	5991421	50	51	0.0354416229718356	n.s.
4690	5991471	51	50	0.000115685976557532	n.s.
4690	5991498	51	50	0.000115685976557532	n.s.
4690	5991538	51	50	0.000115685976557532	n.s.
4690	5991557	51	50	0.000115685976557532	n.s.
4690	5991567	50	51	0.0354416229718356	n.s.
4690	5991572	50	51	0.0354416229718356	n.s.
4690	5991579	50	51	0.0354416229718356	n.s.
4690	5991581	49	52	0.149981658108242	n.s.
4690	5991584	48	53	0.343735791385777	n.s.
4690	5991589	48	53	0.343735791385777	n.s.
4690	5991600	47	54	0.616704022804441	n.s.
4690	5991607	47	54	0.616704022804441	n.s.
4690	5991631	46	55	0.968886352364233	n.s.
4690	5991635	46	55	0.968886352364233	n.s.
4690	5991650	45	56	1.40028278006515	n.s.
4690	5991668	45	56	1.40028278006515	n.s.
4690	5991687	46	55	0.968886352364233	n.s.
4690	5991729	46	55	0.968886352364233	n.s.
4690	5991738	47	54	0.616704022804441	n.s.
4690	5991796	46	55	0.968886352364233	n.s.
4690	5991818	46	55	0.968886352364233	n.s.
4690	5991851	45	56	1.40028278006515	n.s.
4690	5991861	45	56	1.40028278006515	n.s.
4690	5991880	45	56	1.40028278006515	n.s.
4690	5991881	44	57	1.9108933059072	n.s.
4690	5991887	45	56	1.40028278006515	n.s.
4690	5991891	45	56	1.40028278006515	n.s.
4690	5991895	44	57	1.9108933059072	n.s.

4690	5991936	45	56	1.40028278006515	n.s.
4690	5991945	44	57	1.9108933059072	n.s.
4690	5991965	45	56	1.40028278006515	n.s.
4690	5992054	45	56	1.40028278006515	n.s.
4690	5992055	46	55	0.968886352364233	n.s.
4690	5992071	47	54	0.616704022804441	n.s.
4690	5992094	47	54	0.616704022804441	n.s.
4690	5992096	48	53	0.343735791385777	n.s.
4690	5992117	49	52	0.149981658108242	n.s.
4690	5992129	50	51	0.0354416229718356	n.s.
4690	5992133	50	51	0.0354416229718356	n.s.
4690	5992147	51	50	0.000115685976557532	n.s.
4690	5992148	52	49	0.044003847122408	n.s.
4690	5992198	53	48	0.167106106409387	n.s.
4690	5992239	53	48	0.167106106409387	n.s.
4690	5992251	53	48	0.167106106409387	n.s.
4690	5992267	54	47	0.369422463837495	n.s.
4690	5992289	54	47	0.369422463837495	n.s.
4690	5992303	54	47	0.369422463837495	n.s.
4690	5992305	54	47	0.369422463837495	n.s.
4690	5992309	55	46	0.650952919406731	n.s.
4690	5992349	55	46	0.650952919406731	n.s.
4690	5992351	54	47	0.369422463837495	n.s.
4690	5992363	54	47	0.369422463837495	n.s.
4690	5992377	55	46	0.650952919406731	n.s.
4690	5992378	56	45	1.0116974731171	n.s.
4690	5992379	56	45	1.0116974731171	n.s.
4690	5992381	56	45	1.0116974731171	n.s.
4690	5992397	56	45	1.0116974731171	n.s.
4690	5992425	55	46	0.650952919406731	n.s.
4690	5992449	55	46	0.650952919406731	n.s.
4690	5992453	54	47	0.369422463837495	n.s.
4690	5992459	54	47	0.369422463837495	n.s.
4690	5992507	54	47	0.369422463837495	n.s.
4690	5992515	55	46	0.650952919406731	n.s.
4690	5992518	56	45	1.0116974731171	n.s.
4690	5992523	56	45	1.0116974731171	n.s.
4690	5992530	57	44	1.45165612496859	n.s.
4690	5992550	56	45	1.0116974731171	n.s.
4690	5992552	57	44	1.45165612496859	n.s.
4690	5992558	58	43	1.97082887496121	n.s.
4690	5992559	59	42	2.56921572309496	n.s.
4690	5992567	59	42	2.56921572309496	n.s.
4690	5992569	58	43	1.97082887496121	n.s.
4690	5992571	59	42	2.56921572309496	n.s.
4690	5992574	60	41	3.24681666936984	n.s.
4690	5992582	60	41	3.24681666936984	n.s.
4690	5992592	61	40	4.00363171378585	*
4690	5992625	62	39	4.83966085634298	*
4690	5992636	62	39	4.83966085634298	*

4690	5992644	63	38	5.75490409704125	*
4690	5992662	63	38	5.75490409704125	*
4690	5992670	63	38	5.75490409704125	*
4690	5992692	62	39	4.83966085634298	*
4690	5992750	62	39	4.83966085634298	*
4690	5992765	63	38	5.75490409704125	*
4690	5992805	63	38	5.75490409704125	*
4690	5992815	64	37	6.74936143588064	**
4690	5992836	63	38	5.75490409704125	*
4690	5992862	64	37	6.74936143588064	**
4690	5992872	64	37	6.74936143588064	**
4690	5992877	63	38	5.75490409704125	*
4690	5992887	64	37	6.74936143588064	**
4690	5992899	64	37	6.74936143588064	**
4690	5992910	63	38	5.75490409704125	*
4690	5992919	62	39	4.83966085634298	*
4690	5992920	62	39	4.83966085634298	*
4690	5992937	61	40	4.00363171378585	*
4690	5992948	61	40	4.00363171378585	*
4690	5992959	61	40	4.00363171378585	*
4690	5992999	60	41	3.24681666936984	n.s.
4690	5993004	61	40	4.00363171378585	*
4690	5993016	61	40	4.00363171378585	*
4690	5993039	61	40	4.00363171378585	*
4690	5993049	62	39	4.83966085634298	*
4690	5993061	61	40	4.00363171378585	*
4690	5993083	62	39	4.83966085634298	*
4690	5993086	63	38	5.75490409704125	*
4690	5993115	62	39	4.83966085634298	*
4690	5993130	62	39	4.83966085634298	*
4690	5993145	63	38	5.75490409704125	*
4690	5993178	63	38	5.75490409704125	*
4690	5993185	62	39	4.83966085634298	*
4690	5993187	63	38	5.75490409704125	*
4690	5993201	62	39	4.83966085634298	*
4690	5993219	62	39	4.83966085634298	*
4690	5993268	63	38	5.75490409704125	*
4690	5993270	64	37	6.74936143588064	**
4690	5993272	65	36	7.82303287286116	**
4690	5993273	65	36	7.82303287286116	**
4690	5993278	65	36	7.82303287286116	**
4690	5993281	64	37	6.74936143588064	**
4690	5993283	65	36	7.82303287286116	**
4690	5993284	65	36	7.82303287286116	**
4690	5993441	66	35	8.97591840798281	**
4690	5993443	66	35	8.97591840798281	**
4690	5993455	65	36	7.82303287286116	**
4690	5993525	66	35	8.97591840798281	**
4690	5993532	65	36	7.82303287286116	**
4690	5993546	65	36	7.82303287286116	**

4690	5993555	66	35	8.97591840798281	**
4690	5993631	65	36	7.82303287286116	**
4690	5993653	66	35	8.97591840798281	**
4690	5993656	65	36	7.82303287286116	**
4690	5993738	66	35	8.97591840798281	**
4690	5993746	66	35	8.97591840798281	**
4690	5993747	66	35	8.97591840798281	**
4690	5993751	66	35	8.97591840798281	**
4690	5993780	65	36	7.82303287286116	**
4690	5993829	65	36	7.82303287286116	**
4690	5993834	65	36	7.82303287286116	**
4690	5993838	65	36	7.82303287286116	**
4690	5993844	64	37	6.74936143588064	**
4690	5993862	64	37	6.74936143588064	**
4690	5993888	63	38	5.75490409704125	*
4690	5993901	63	38	5.75490409704125	*
4690	5993953	62	39	4.83966085634298	*
4690	5993962	61	40	4.00363171378585	*
4690	5993983	61	40	4.00363171378585	*
4690	5993995	60	41	3.24681666936984	n.s.
4690	5994007	60	41	3.24681666936984	n.s.
4690	5994008	59	42	2.56921572309496	n.s.
4690	5994012	59	42	2.56921572309496	n.s.
4690	5994015	59	42	2.56921572309496	n.s.
4690	5994016	58	43	1.97082887496121	n.s.
4690	5994033	57	44	1.45165612496859	n.s.
4690	5994038	56	45	1.0116974731171	n.s.
4690	5994039	55	46	0.650952919406731	n.s.
4690	5994040	54	47	0.369422463837495	n.s.
4690	5994054	55	46	0.650952919406731	n.s.
4690	5994062	55	46	0.650952919406731	n.s.
4690	5994064	55	46	0.650952919406731	n.s.
4690	5994065	55	46	0.650952919406731	n.s.
4690	5994074	55	46	0.650952919406731	n.s.
4690	5994090	56	45	1.0116974731171	n.s.
4690	5994134	55	46	0.650952919406731	n.s.
4690	5994144	54	47	0.369422463837495	n.s.
4690	5994159	54	47	0.369422463837495	n.s.
4690	5994165	53	48	0.167106106409387	n.s.
4690	5994166	54	47	0.369422463837495	n.s.
4690	5994167	54	47	0.369422463837495	n.s.
4690	5994172	54	47	0.369422463837495	n.s.
4690	5994185	54	47	0.369422463837495	n.s.
4690	5994224	54	47	0.369422463837495	n.s.
4690	5994248	54	47	0.369422463837495	n.s.
4690	5994275	54	47	0.369422463837495	n.s.
4690	5994319	54	47	0.369422463837495	n.s.
4690	5994331	55	46	0.650952919406731	n.s.
4690	5994339	55	46	0.650952919406731	n.s.
4690	5994340	55	46	0.650952919406731	n.s.

4690	5994354	54	47	0.369422463837495	n.s.
4690	5994356	54	47	0.369422463837495	n.s.
4690	5994358	54	47	0.369422463837495	n.s.
4690	5994370	55	46	0.650952919406731	n.s.
4690	5994372	55	46	0.650952919406731	n.s.
4690	5994382	55	46	0.650952919406731	n.s.
4690	5994432	55	46	0.650952919406731	n.s.
4690	5994473	55	46	0.650952919406731	n.s.
4690	5994502	54	47	0.369422463837495	n.s.
4690	5994504	55	46	0.650952919406731	n.s.
4690	5994519	54	47	0.369422463837495	n.s.
4690	5994522	55	46	0.650952919406731	n.s.
4690	5994528	55	46	0.650952919406731	n.s.
4690	5994535	55	46	0.650952919406731	n.s.
4690	5994561	55	46	0.650952919406731	n.s.
4690	5994563	56	45	1.0116974731171	n.s.
4690	5994570	57	44	1.45165612496859	n.s.
4690	5994579	56	45	1.0116974731171	n.s.
4690	5994580	57	44	1.45165612496859	n.s.
4690	5994598	56	45	1.0116974731171	n.s.
4690	5994602	56	45	1.0116974731171	n.s.
4690	5994675	57	44	1.45165612496859	n.s.
4690	5994677	57	44	1.45165612496859	n.s.
4690	5994687	56	45	1.0116974731171	n.s.
4690	5994714	56	45	1.0116974731171	n.s.
4690	5994729	56	45	1.0116974731171	n.s.
4690	5994756	56	45	1.0116974731171	n.s.
4690	5994765	56	45	1.0116974731171	n.s.
4690	5994771	56	45	1.0116974731171	n.s.
4690	5994772	57	44	1.45165612496859	n.s.
4690	5994788	57	44	1.45165612496859	n.s.
4690	5994801	57	44	1.45165612496859	n.s.
4690	5994809	58	43	1.97082887496121	n.s.
4690	5994816	59	42	2.56921572309496	n.s.
4690	5994831	58	43	1.97082887496121	n.s.
4690	5994834	59	42	2.56921572309496	n.s.
4690	5994843	59	42	2.56921572309496	n.s.
4690	5994873	59	42	2.56921572309496	n.s.
4690	5994879	59	42	2.56921572309496	n.s.
4690	5994922	59	42	2.56921572309496	n.s.
4690	5994927	59	42	2.56921572309496	n.s.
4690	5994930	59	42	2.56921572309496	n.s.
4690	5994951	60	41	3.24681666936984	n.s.
4690	5994956	60	41	3.24681666936984	n.s.
4690	5994977	61	40	4.00363171378585	*
4690	5995038	61	40	4.00363171378585	*
4690	5995039	61	40	4.00363171378585	*
4690	5995071	62	39	4.83966085634298	*
4690	5995086	62	39	4.83966085634298	*
4690	5995123	63	38	5.75490409704125	*

4690	5995132	64	37	6.74936143588064	**
4690	5995133	64	37	6.74936143588064	**
4690	5995140	65	36	7.82303287286116	**
4690	5995160	65	36	7.82303287286116	**
4690	5995170	66	35	8.97591840798281	**
4690	5995185	66	35	8.97591840798281	**
4690	5995198	66	35	8.97591840798281	**
4690	5995245	66	35	8.97591840798281	**
4690	5995257	66	35	8.97591840798281	**
4690	5995259	67	34	10.2080180412456	**
4690	5995273	67	34	10.2080180412456	**
4690	5995315	67	34	10.2080180412456	**
4690	5995328	68	33	11.5193317726495	***
4690	5995329	68	33	11.5193317726495	***
4690	5995333	68	33	11.5193317726495	***
4690	5995335	69	32	12.9098596021945	***
4690	5995345	68	33	11.5193317726495	***
4690	5995354	69	32	12.9098596021945	***
4690	5995374	70	31	14.3796015298807	***
4690	5995401	69	32	12.9098596021945	***
4690	5995402	69	32	12.9098596021945	***
4690	5995411	69	32	12.9098596021945	***
4690	5995413	70	31	14.3796015298807	***
4690	5995416	71	30	15.928557555708	***
4690	5995418	72	29	17.5567276796764	***
4690	5995426	73	28	19.264111901786	***
4690	5995427	74	27	21.0507102220366	***
4690	5995432	75	26	22.9165226404284	***
4690	5995437	76	25	24.8615491569614	***
4690	5995442	77	24	26.8857897716355	***
4690	5995446	77	24	26.8857897716355	***
4690	5995451	78	23	28.9892444844506	***
4690	5995452	78	23	28.9892444844506	***
4690	5995454	79	22	31.171913295407	***
4690	5995473	79	22	31.171913295407	***
4690	5995508	79	22	31.171913295407	***
4690	5995509	80	21	33.4337962045044	***
4690	5995544	81	20	35.774893211743	***
4690	5995551	82	19	38.1952043171227	***
4690	5995553	82	19	38.1952043171227	***
4690	5995554	82	19	38.1952043171227	***
4690	5995560	82	19	38.1952043171227	***
4690	5995568	82	19	38.1952043171227	***
4690	5995581	82	19	38.1952043171227	***
4690	5995590	82	19	38.1952043171227	***
4690	5995595	83	18	40.6947295206435	***
4690	5995601	83	18	40.6947295206435	***
4690	5995605	83	18	40.6947295206435	***
4690	5995618	83	18	40.6947295206435	***
4690	5995626	83	18	40.6947295206435	***

4690	5995646	83	18	40.6947295206435	***
4690	5995650	84	17	43.2734688223055	***
4690	5995657	84	17	43.2734688223055	***
4690	5995660	84	17	43.2734688223055	***
4690	5995686	84	17	43.2734688223055	***
4690	5995690	85	16	45.9314222221086	***
4690	5995694	86	15	48.6685897200528	***
4690	5995695	86	15	48.6685897200528	***
4690	5995698	87	14	51.4849713161382	***
4690	5995705	88	13	54.3805670103646	***
4690	5995720	88	13	54.3805670103646	***
4690	5995725	89	12	57.3553768027323	***
4690	5995726	89	12	57.3553768027323	***
4690	5995731	90	11	60.409400693241	***
4690	5995735	90	11	60.409400693241	***
4690	5995745	90	11	60.409400693241	***
4690	5995755	90	11	60.409400693241	***
4690	5995765	90	11	60.409400693241	***
4690	5995767	91	10	63.5426386818908	***
4690	5995790	91	10	63.5426386818908	***
4690	5995793	92	9	66.7550907686818	***
4690	5995806	92	9	66.7550907686818	***
4690	5995812	92	9	66.7550907686818	***
4690	5995813	92	9	66.7550907686818	***
4690	5995814	93	8	70.046756953614	***
4690	5995815	93	8	70.046756953614	***
4690	5995816	93	8	70.046756953614	***
4690	5995818	94	7	73.4176372366872	***
4690	5995820	94	7	73.4176372366872	***
4690	5995821	94	7	73.4176372366872	***
4690	5995822	94	7	73.4176372366872	***
4690	5995823	94	7	73.4176372366872	***
4690	5995824	94	7	73.4176372366872	***
4690	5995827	94	7	73.4176372366872	***
4690	5995828	94	7	73.4176372366872	***
4690	5995829	95	6	76.8677316179016	***
4690	5995832	95	6	76.8677316179016	***
4690	5995833	96	5	80.3970400972571	***
4690	5995835	96	5	80.3970400972571	***
4690	5995836	95	6	76.8677316179016	***
4690	5995837	94	7	73.4176372366872	***
4690	5995838	94	7	73.4176372366872	***
4690	5995840	93	8	70.046756953614	***
4690	5995842	92	9	66.7550907686818	***
4690	5995843	92	9	66.7550907686818	***
4690	5995844	92	9	66.7550907686818	***
4690	5995845	92	9	66.7550907686818	***
4690	5995849	92	9	66.7550907686818	***
4690	5995851	91	10	63.5426386818908	***
4690	5995852	91	10	63.5426386818908	***

4690	5995855	90	11	60.409400693241	***
4690	5995856	90	11	60.409400693241	***
4690	5995857	89	12	57.3553768027323	***
4690	5995859	89	12	57.3553768027323	***
4690	5995860	89	12	57.3553768027323	***
4690	5995861	89	12	57.3553768027323	***
4690	5995862	89	12	57.3553768027323	***
4690	5995864	89	12	57.3553768027323	***
4690	5995865	88	13	54.3805670103646	***
4690	5995866	88	13	54.3805670103646	***
4690	5995867	88	13	54.3805670103646	***
4690	5995868	88	13	54.3805670103646	***
4690	5995869	87	14	51.4849713161382	***
4690	5995870	86	15	48.6685897200528	***
4690	5995871	86	15	48.6685897200528	***
4690	5995872	86	15	48.6685897200528	***
4690	5995873	86	15	48.6685897200528	***
4690	5995875	87	14	51.4849713161382	***
4690	5995876	87	14	51.4849713161382	***
4690	5995877	86	15	48.6685897200528	***
4690	5995878	86	15	48.6685897200528	***
4690	5995879	87	14	51.4849713161382	***
4690	5995880	87	14	51.4849713161382	***
4690	5995882	87	14	51.4849713161382	***
4690	5995884	87	14	51.4849713161382	***
4690	5995885	87	14	51.4849713161382	***
4690	5995886	87	14	51.4849713161382	***
4690	5995888	86	15	48.6685897200528	***
4690	5995889	86	15	48.6685897200528	***
4690	5995890	86	15	48.6685897200528	***
4690	5995892	86	15	48.6685897200528	***
4690	5995893	86	15	48.6685897200528	***
4690	5995894	86	15	48.6685897200528	***
4690	5995896	86	15	48.6685897200528	***
4690	5995897	86	15	48.6685897200528	***
4690	5995898	85	16	45.9314222221086	***
4690	5995900	85	16	45.9314222221086	***
4690	5995901	85	16	45.9314222221086	***
4690	5995970	84	17	43.2734688223055	***
4690	5995971	84	17	43.2734688223055	***
4690	5995978	84	17	43.2734688223055	***
4690	5995987	84	17	43.2734688223055	***
4690	5995999	84	17	43.2734688223055	***
4690	5996005	83	18	40.6947295206435	***
4690	5996059	83	18	40.6947295206435	***
4690	5996063	82	19	38.1952043171227	***
4690	5996077	81	20	35.774893211743	***
4690	5996085	81	20	35.774893211743	***
4690	5996100	81	20	35.774893211743	***
4690	5996104	81	20	35.774893211743	***

4690	5996115	81	20	35.774893211743	***
4690	5996118	81	20	35.774893211743	***
4690	5996119	81	20	35.774893211743	***
4690	5996128	81	20	35.774893211743	***
4690	5996160	80	21	33.4337962045044	***
4690	5996172	80	21	33.4337962045044	***
4690	5996213	79	22	31.171913295407	***
4690	5996228	78	23	28.9892444844506	***
4690	5996240	77	24	26.8857897716355	***
4690	5996251	77	24	26.8857897716355	***
4690	5996275	77	24	26.8857897716355	***
4690	5996283	77	24	26.8857897716355	***
4690	5996284	77	24	26.8857897716355	***
4690	5996320	77	24	26.8857897716355	***
4690	5996335	77	24	26.8857897716355	***
4690	5996337	77	24	26.8857897716355	***
4690	5996341	77	24	26.8857897716355	***
4690	5996342	77	24	26.8857897716355	***
4690	5996362	77	24	26.8857897716355	***
4690	5996363	77	24	26.8857897716355	***
4690	5996370	77	24	26.8857897716355	***
4690	5996380	77	24	26.8857897716355	***
4690	5996416	77	24	26.8857897716355	***
4690	5996449	76	25	24.8615491569614	***
4690	5996461	75	26	22.9165226404284	***
4690	5996539	74	27	21.0507102220366	***
4690	5996560	73	28	19.264111901786	***
4690	5996566	73	28	19.264111901786	***
4690	5996578	73	28	19.264111901786	***
4690	5996599	72	29	17.5567276796764	***
4690	5996608	72	29	17.5567276796764	***
4690	5996615	71	30	15.928557555708	***
4690	5996644	70	31	14.3796015298807	***
4690	5996668	69	32	12.9098596021945	***
4690	5996681	68	33	11.5193317726495	***
4690	5996689	68	33	11.5193317726495	***
4690	5996735	68	33	11.5193317726495	***
4690	5996755	68	33	11.5193317726495	***
4690	5996764	68	33	11.5193317726495	***
4690	5996767	68	33	11.5193317726495	***
4690	5996769	68	33	11.5193317726495	***
4690	5996772	69	32	12.9098596021945	***
4690	5996786	68	33	11.5193317726495	***
4690	5996821	68	33	11.5193317726495	***
4690	5996824	68	33	11.5193317726495	***
4690	5996837	68	33	11.5193317726495	***
4690	5996838	68	33	11.5193317726495	***
4690	5996854	68	33	11.5193317726495	***
4690	5996859	67	34	10.2080180412456	**
4690	5996876	68	33	11.5193317726495	***

4690	5996890	68	33	11.5193317726495	***
4690	5996893	69	32	12.9098596021945	***
4690	5996909	69	32	12.9098596021945	***
4690	5996925	70	31	14.3796015298807	***
4690	5996971	69	32	12.9098596021945	***
4690	5996973	68	33	11.5193317726495	***
4690	5996978	68	33	11.5193317726495	***
4690	5996985	67	34	10.2080180412456	**
4690	5996986	66	35	8.97591840798281	**
4690	5997030	66	35	8.97591840798281	**
4690	5997054	66	35	8.97591840798281	**
4690	5997056	66	35	8.97591840798281	**
4690	5997064	66	35	8.97591840798281	**
4690	5997074	67	34	10.2080180412456	**
4690	5997098	68	33	11.5193317726495	***
4690	5997101	69	32	12.9098596021945	***
4690	5997125	69	32	12.9098596021945	***
4690	5997160	69	32	12.9098596021945	***
4690	5997161	68	33	11.5193317726495	***
4690	5997171	68	33	11.5193317726495	***
4690	5997181	69	32	12.9098596021945	***
4690	5997183	69	32	12.9098596021945	***
4690	5997203	68	33	11.5193317726495	***
4690	5997204	68	33	11.5193317726495	***
4690	5997207	67	34	10.2080180412456	**
4690	5997210	66	35	8.97591840798281	**
4690	5997215	65	36	7.82303287286116	**
4690	5997216	64	37	6.74936143588064	**
4690	5997225	65	36	7.82303287286116	**
4690	5997237	64	37	6.74936143588064	**
4690	5997242	64	37	6.74936143588064	**
4690	5997244	64	37	6.74936143588064	**
4690	5997262	63	38	5.75490409704125	*
4690	5997271	63	38	5.75490409704125	*
4690	5997272	63	38	5.75490409704125	*
4690	5997288	62	39	4.83966085634298	*
4690	5997302	63	38	5.75490409704125	*
4690	5997307	63	38	5.75490409704125	*
4690	5997310	62	39	4.83966085634298	*
4690	5997330	63	38	5.75490409704125	*
4690	5997338	62	39	4.83966085634298	*
4690	5997346	63	38	5.75490409704125	*
4690	5997347	63	38	5.75490409704125	*
4690	5997367	62	39	4.83966085634298	*
4690	5997370	63	38	5.75490409704125	*
4690	5997389	63	38	5.75490409704125	*
4690	5997400	64	37	6.74936143588064	**
4690	5997403	64	37	6.74936143588064	**
4690	5997404	64	37	6.74936143588064	**
4690	5997406	64	37	6.74936143588064	**

4690	5997407	63	38	5.75490409704125	*
4690	5997408	63	38	5.75490409704125	*
4690	5997411	63	38	5.75490409704125	*
4690	5997412	62	39	4.83966085634298	*
4690	5997427	61	40	4.00363171378585	*
4690	5997459	61	40	4.00363171378585	*
4690	5997473	61	40	4.00363171378585	*
4690	5997486	61	40	4.00363171378585	*
4690	5997510	61	40	4.00363171378585	*
4690	5997523	61	40	4.00363171378585	*
4690	5997524	61	40	4.00363171378585	*
4690	5997550	60	41	3.24681666936984	n.s.
4690	5997551	59	42	2.56921572309496	n.s.
4690	5997557	58	43	1.97082887496121	n.s.
4690	5997558	58	43	1.97082887496121	n.s.
4690	5997559	58	43	1.97082887496121	n.s.
4690	5997560	58	43	1.97082887496121	n.s.
4690	5997561	57	44	1.45165612496859	n.s.
4690	5997598	56	45	1.0116974731171	n.s.
4690	5997614	55	46	0.650952919406731	n.s.
4690	5997615	54	47	0.369422463837495	n.s.
4690	5997625	54	47	0.369422463837495	n.s.
4690	5997627	54	47	0.369422463837495	n.s.
4690	5997637	53	48	0.167106106409387	n.s.
4690	5997640	53	48	0.167106106409387	n.s.
4690	5997659	53	48	0.167106106409387	n.s.
4690	5997670	54	47	0.369422463837495	n.s.
4690	5997696	55	46	0.650952919406731	n.s.
4690	5997699	55	46	0.650952919406731	n.s.
4690	5997708	55	46	0.650952919406731	n.s.
4690	5997772	55	46	0.650952919406731	n.s.
4690	5997801	55	46	0.650952919406731	n.s.
4690	5997813	56	45	1.0116974731171	n.s.
4690	5997822	57	44	1.45165612496859	n.s.
4690	5997839	58	43	1.97082887496121	n.s.
4690	5997887	59	42	2.56921572309496	n.s.
4690	5997956	59	42	2.56921572309496	n.s.
4690	5997989	59	42	2.56921572309496	n.s.
4690	5997991	59	42	2.56921572309496	n.s.
4690	5997996	58	43	1.97082887496121	n.s.
4690	5998025	58	43	1.97082887496121	n.s.
4690	5998026	58	43	1.97082887496121	n.s.
4690	5998046	58	43	1.97082887496121	n.s.
4690	5998061	58	43	1.97082887496121	n.s.
4690	5998066	58	43	1.97082887496121	n.s.
4690	5998093	58	43	1.97082887496121	n.s.
4690	5998099	57	44	1.45165612496859	n.s.
4690	5998102	56	45	1.0116974731171	n.s.
4690	5998117	55	46	0.650952919406731	n.s.
4690	5998156	55	46	0.650952919406731	n.s.

4690	5998171	55	46	0.650952919406731	n.s.	
4690	5998186	55	46	0.650952919406731	n.s.	
4690	5998236	54	47	0.369422463837495	n.s.	
4690	5998256	53	48	0.167106106409387	n.s.	
4690	5998260	52	49	0.044003847122408	n.s.	
4690	5998268	52	49	0.044003847122408	n.s.	
4690	5998271	53	48	0.167106106409387	n.s.	
4690	5998299	52	49	0.044003847122408	n.s.	
4690	5998300	52	49	0.044003847122408	n.s.	
4690	5998306	52	49	0.044003847122408	n.s.	
4690	5998350	52	49	0.044003847122408	n.s.	
4690	5998354	51	50	0.000115685976557532		n.s.
4690	5998369	50	51	0.0354416229718356	n.s.	
4690	5998373	49	52	0.149981658108242	n.s.	
4690	5998390	48	53	0.343735791385777	n.s.	
4690	5998392	47	54	0.616704022804441	n.s.	
4690	5998402	46	55	0.968886352364233	n.s.	
4690	5998406	45	56	1.40028278006515	n.s.	
4690	5998425	44	57	1.9108933059072	n.s.	
4690	5998447	45	56	1.40028278006515	n.s.	
4690	5998467	44	57	1.9108933059072	n.s.	
4690	5998554	44	57	1.9108933059072	n.s.	
4690	5998562	45	56	1.40028278006515	n.s.	
4690	5998581	45	56	1.40028278006515	n.s.	
4690	5998588	44	57	1.9108933059072	n.s.	
4690	5998595	44	57	1.9108933059072	n.s.	
4690	5998602	44	57	1.9108933059072	n.s.	
4690	5998611	45	56	1.40028278006515	n.s.	
4690	5998618	46	55	0.968886352364233	n.s.	
4690	5998620	45	56	1.40028278006515	n.s.	
4690	5998621	46	55	0.968886352364233	n.s.	
4690	5998636	46	55	0.968886352364233	n.s.	
4690	5998638	45	56	1.40028278006515	n.s.	
4690	5998640	45	56	1.40028278006515	n.s.	
4690	5998646	44	57	1.9108933059072	n.s.	
4690	5998647	44	57	1.9108933059072	n.s.	
4690	5998648	45	56	1.40028278006515	n.s.	
4690	5998684	45	56	1.40028278006515	n.s.	
4690	5998687	44	57	1.9108933059072	n.s.	
4690	5998697	45	56	1.40028278006515	n.s.	
4690	5998698	45	56	1.40028278006515	n.s.	
4690	5998727	45	56	1.40028278006515	n.s.	
4690	5998740	44	57	1.9108933059072	n.s.	
4690	5998758	44	57	1.9108933059072	n.s.	
4690	5998782	44	57	1.9108933059072	n.s.	
4690	5998786	43	58	2.50071792989038	n.s.	
4690	5998795	42	59	3.16975665201469	n.s.	
4690	5998802	42	59	3.16975665201469	n.s.	
4690	5998811	42	59	3.16975665201469	n.s.	
4690	5998833	41	60	3.91800947228012	*	

4690	5998835	40	61	4.74547639068669	*
4690	5998840	41	60	3.91800947228012	*
4690	5998868	40	61	4.74547639068669	*
4690	5998871	39	62	5.65215740723438	*
4690	5998878	40	61	4.74547639068669	*
4690	5998882	40	61	4.74547639068669	*
4690	5998889	40	61	4.74547639068669	*
4690	5998892	39	62	5.65215740723438	*
4690	5998901	40	61	4.74547639068669	*
4690	5998904	40	61	4.74547639068669	*
4690	5998908	41	60	3.91800947228012	*
4690	5998914	40	61	4.74547639068669	*
4690	5998918	41	60	3.91800947228012	*
4690	5998924	41	60	3.91800947228012	*
4690	5998929	42	59	3.16975665201469	n.s.
4690	5998943	42	59	3.16975665201469	n.s.
4690	5998957	42	59	3.16975665201469	n.s.
4690	5998961	42	59	3.16975665201469	n.s.
4690	5999006	43	58	2.50071792989038	n.s.
4690	5999009	44	57	1.9108933059072	n.s.
4690	5999031	44	57	1.9108933059072	n.s.
4690	5999060	45	56	1.40028278006515	n.s.
4690	5999081	45	56	1.40028278006515	n.s.
4690	5999099	45	56	1.40028278006515	n.s.
4690	5999107	46	55	0.968886352364233	n.s.
4690	5999125	47	54	0.616704022804441	n.s.
4690	5999152	48	53	0.343735791385777	n.s.
4690	5999166	48	53	0.343735791385777	n.s.
4690	5999167	48	53	0.343735791385777	n.s.
4690	5999182	47	54	0.616704022804441	n.s.
4690	5999199	47	54	0.616704022804441	n.s.
4690	5999200	48	53	0.343735791385777	n.s.
4690	5999201	47	54	0.616704022804441	n.s.
4690	5999213	47	54	0.616704022804441	n.s.
4690	5999232	47	54	0.616704022804441	n.s.
4690	5999237	47	54	0.616704022804441	n.s.
4690	5999268	46	55	0.968886352364233	n.s.
4690	5999293	45	56	1.40028278006515	n.s.
4690	5999310	44	57	1.9108933059072	n.s.
4690	5999311	44	57	1.9108933059072	n.s.
4690	5999324	45	56	1.40028278006515	n.s.
4690	5999333	45	56	1.40028278006515	n.s.
4690	5999340	45	56	1.40028278006515	n.s.
4690	5999361	45	56	1.40028278006515	n.s.
4690	5999364	46	55	0.968886352364233	n.s.
4690	5999369	47	54	0.616704022804441	n.s.
4690	5999371	48	53	0.343735791385777	n.s.
4690	5999381	48	53	0.343735791385777	n.s.
4690	5999385	48	53	0.343735791385777	n.s.
4690	5999399	48	53	0.343735791385777	n.s.

4690	5999402	49	52	0.149981658108242	n.s.
4690	5999403	48	53	0.343735791385777	n.s.
4690	5999405	47	54	0.616704022804441	n.s.
4690	5999407	47	54	0.616704022804441	n.s.
4690	5999414	47	54	0.616704022804441	n.s.
4690	5999416	47	54	0.616704022804441	n.s.
4690	5999421	48	53	0.343735791385777	n.s.
4690	5999423	48	53	0.343735791385777	n.s.
4690	5999426	49	52	0.149981658108242	n.s.
4690	5999429	49	52	0.149981658108242	n.s.
4690	5999440	50	51	0.0354416229718356	n.s.
4690	5999443	50	51	0.0354416229718356	n.s.
4690	5999446	50	51	0.0354416229718356	n.s.
4690	5999456	50	51	0.0354416229718356	n.s.
4690	5999460	51	50	0.000115685976557532	n.s.
4690	5999461	52	49	0.044003847122408	n.s.
4690	5999464	52	49	0.044003847122408	n.s.
4690	5999466	52	49	0.044003847122408	n.s.
4690	5999469	53	48	0.167106106409387	n.s.
4690	5999474	53	48	0.167106106409387	n.s.
4690	5999477	52	49	0.044003847122408	n.s.
4690	5999518	53	48	0.167106106409387	n.s.
4690	5999521	52	49	0.044003847122408	n.s.
4690	5999524	52	49	0.044003847122408	n.s.
4690	5999533	53	48	0.167106106409387	n.s.
4690	5999538	54	47	0.369422463837495	n.s.
4690	5999563	54	47	0.369422463837495	n.s.
4690	5999573	54	47	0.369422463837495	n.s.
4690	5999579	53	48	0.167106106409387	n.s.
4690	5999586	52	49	0.044003847122408	n.s.
4690	5999593	53	48	0.167106106409387	n.s.
4690	5999594	53	48	0.167106106409387	n.s.
4690	5999599	52	49	0.044003847122408	n.s.
4690	5999609	53	48	0.167106106409387	n.s.
4690	5999612	53	48	0.167106106409387	n.s.
4690	5999614	53	48	0.167106106409387	n.s.
4690	5999621	52	49	0.044003847122408	n.s.
4690	5999623	52	49	0.044003847122408	n.s.
4690	5999624	51	50	0.000115685976557532	n.s.
4690	5999632	51	50	0.000115685976557532	n.s.
4690	5999635	50	51	0.0354416229718356	n.s.
4690	5999637	49	52	0.149981658108242	n.s.
4690	5999639	49	52	0.149981658108242	n.s.
4690	5999645	49	52	0.149981658108242	n.s.
4690	5999654	49	52	0.149981658108242	n.s.
4690	5999655	50	51	0.0354416229718356	n.s.
4690	5999661	51	50	0.000115685976557532	n.s.
4690	5999662	52	49	0.044003847122408	n.s.
4690	5999663	52	49	0.044003847122408	n.s.
4690	5999671	53	48	0.167106106409387	n.s.

4690	5999684	53	48	0.167106106409387	n.s.
4690	5999836	53	48	0.167106106409387	n.s.
4690	5999839	53	48	0.167106106409387	n.s.
4690	5999842	54	47	0.369422463837495	n.s.
4690	5999843	55	46	0.650952919406731	n.s.
4690	5999852	54	47	0.369422463837495	n.s.
4690	5999857	55	46	0.650952919406731	n.s.
4690	5999858	56	45	1.0116974731171	n.s.
4690	5999859	56	45	1.0116974731171	n.s.
4690	5999860	56	45	1.0116974731171	n.s.
4690	5999862	57	44	1.45165612496859	n.s.
4690	5999869	57	44	1.45165612496859	n.s.
4690	5999875	57	44	1.45165612496859	n.s.
4690	5999891	57	44	1.45165612496859	n.s.
4690	5999895	58	43	1.97082887496121	n.s.
4690	5999901	58	43	1.97082887496121	n.s.
4690	5999907	58	43	1.97082887496121	n.s.
4690	5999937	58	43	1.97082887496121	n.s.
4690	5999944	58	43	1.97082887496121	n.s.
4690	5999955	57	44	1.45165612496859	n.s.
4690	5999965	57	44	1.45165612496859	n.s.
4690	5999967	58	43	1.97082887496121	n.s.
4690	5999971	58	43	1.97082887496121	n.s.
4690	5999985	57	44	1.45165612496859	n.s.
4690	6000003	56	45	1.0116974731171	n.s.
4690	6000004	56	45	1.0116974731171	n.s.
4690	6000022	55	46	0.650952919406731	n.s.
4690	6000026	54	47	0.369422463837495	n.s.
4690	6000031	54	47	0.369422463837495	n.s.
4690	6000047	54	47	0.369422463837495	n.s.
4690	6000138	54	47	0.369422463837495	n.s.
4690	6000143	53	48	0.167106106409387	n.s.
4690	6000233	53	48	0.167106106409387	n.s.
4690	6000238	54	47	0.369422463837495	n.s.
4690	6000256	54	47	0.369422463837495	n.s.
4690	6000267	54	47	0.369422463837495	n.s.
4690	6000278	54	47	0.369422463837495	n.s.
4690	6000288	55	46	0.650952919406731	n.s.
4690	6000292	56	45	1.0116974731171	n.s.
4690	6000294	56	45	1.0116974731171	n.s.
4690	6000300	56	45	1.0116974731171	n.s.
4690	6000307	55	46	0.650952919406731	n.s.
4690	6000309	55	46	0.650952919406731	n.s.
4690	6000382	56	45	1.0116974731171	n.s.
4690	6000459	56	45	1.0116974731171	n.s.
4690	6000504	56	45	1.0116974731171	n.s.
4690	6000506	55	46	0.650952919406731	n.s.
4690	6000510	54	47	0.369422463837495	n.s.
4690	6000511	54	47	0.369422463837495	n.s.
4690	6000513	55	46	0.650952919406731	n.s.

4690	6000523	55	46	0.650952919406731	n.s.
4690	6000535	55	46	0.650952919406731	n.s.
4690	6000545	56	45	1.0116974731171	n.s.
4690	6000565	56	45	1.0116974731171	n.s.
4690	6000679	56	45	1.0116974731171	n.s.
4690	6000685	57	44	1.45165612496859	n.s.
4690	6000691	58	43	1.97082887496121	n.s.
4690	6000700	58	43	1.97082887496121	n.s.
4690	6000727	58	43	1.97082887496121	n.s.
4690	6000735	58	43	1.97082887496121	n.s.
4690	6000750	59	42	2.56921572309496	n.s.
4690	6000751	58	43	1.97082887496121	n.s.
4690	6000759	58	43	1.97082887496121	n.s.
4690	6000793	59	42	2.56921572309496	n.s.
4690	6000795	59	42	2.56921572309496	n.s.
4690	6000797	59	42	2.56921572309496	n.s.
4690	6000798	59	42	2.56921572309496	n.s.
4690	6000799	60	41	3.24681666936984	n.s.
4690	6000811	60	41	3.24681666936984	n.s.
4690	6000812	59	42	2.56921572309496	n.s.
4690	6000826	59	42	2.56921572309496	n.s.
4690	6000838	59	42	2.56921572309496	n.s.
4690	6000876	58	43	1.97082887496121	n.s.
4690	6000879	58	43	1.97082887496121	n.s.
4690	6000941	57	44	1.45165612496859	n.s.
4690	6000973	56	45	1.0116974731171	n.s.
4690	6000995	55	46	0.650952919406731	n.s.
4690	6000997	55	46	0.650952919406731	n.s.
4690	6000998	55	46	0.650952919406731	n.s.
4690	6001004	55	46	0.650952919406731	n.s.
4690	6001042	55	46	0.650952919406731	n.s.
4690	6001165	55	46	0.650952919406731	n.s.
4690	6001195	55	46	0.650952919406731	n.s.
4690	6001200	56	45	1.0116974731171	n.s.
4690	6001201	56	45	1.0116974731171	n.s.
4690	6001202	57	44	1.45165612496859	n.s.
4690	6001205	58	43	1.97082887496121	n.s.
4690	6001206	58	43	1.97082887496121	n.s.
4690	6001208	57	44	1.45165612496859	n.s.
4690	6001220	58	43	1.97082887496121	n.s.
4690	6001235	58	43	1.97082887496121	n.s.
4690	6001262	59	42	2.56921572309496	n.s.
4690	6001266	60	41	3.24681666936984	n.s.
4690	6001270	60	41	3.24681666936984	n.s.
4690	6001281	61	40	4.00363171378585	*
4690	6001284	61	40	4.00363171378585	*
4690	6001306	60	41	3.24681666936984	n.s.
4690	6001321	60	41	3.24681666936984	n.s.
4690	6001334	59	42	2.56921572309496	n.s.
4690	6001354	59	42	2.56921572309496	n.s.

4690	6001358	58	43	1.97082887496121	n.s.
4690	6001390	59	42	2.56921572309496	n.s.
4690	6001391	60	41	3.24681666936984	n.s.
4690	6001400	59	42	2.56921572309496	n.s.
4690	6001404	59	42	2.56921572309496	n.s.
4690	6001447	59	42	2.56921572309496	n.s.
4690	6001504	59	42	2.56921572309496	n.s.
4690	6001519	59	42	2.56921572309496	n.s.
4690	6001535	59	42	2.56921572309496	n.s.
4690	6001540	59	42	2.56921572309496	n.s.
4690	6001545	58	43	1.97082887496121	n.s.
4690	6001554	58	43	1.97082887496121	n.s.
4690	6001576	57	44	1.45165612496859	n.s.
4690	6001582	57	44	1.45165612496859	n.s.
4690	6001585	57	44	1.45165612496859	n.s.
4690	6001606	57	44	1.45165612496859	n.s.
4690	6001625	56	45	1.0116974731171	n.s.
4690	6001631	55	46	0.650952919406731	n.s.
4690	6001676	55	46	0.650952919406731	n.s.
4690	6001677	55	46	0.650952919406731	n.s.
4690	6001685	56	45	1.0116974731171	n.s.
4690	6001723	55	46	0.650952919406731	n.s.
4690	6001733	54	47	0.369422463837495	n.s.
4690	6001760	54	47	0.369422463837495	n.s.
4690	6001804	54	47	0.369422463837495	n.s.
4690	6001822	54	47	0.369422463837495	n.s.
4690	6001856	54	47	0.369422463837495	n.s.
4690	6001876	55	46	0.650952919406731	n.s.
4690	6001906	55	46	0.650952919406731	n.s.
4690	6001910	54	47	0.369422463837495	n.s.
4690	6001916	54	47	0.369422463837495	n.s.
4690	6001922	54	47	0.369422463837495	n.s.
4690	6001924	54	47	0.369422463837495	n.s.
4690	6001930	53	48	0.167106106409387	n.s.
4690	6001933	52	49	0.044003847122408	n.s.
4690	6001934	52	49	0.044003847122408	n.s.
4690	6001935	52	49	0.044003847122408	n.s.
4690	6001937	52	49	0.044003847122408	n.s.
4690	6001938	52	49	0.044003847122408	n.s.
4690	6001950	52	49	0.044003847122408	n.s.
4690	6001957	53	48	0.167106106409387	n.s.
4690	6001960	53	48	0.167106106409387	n.s.
4690	6001979	54	47	0.369422463837495	n.s.
4690	6001980	54	47	0.369422463837495	n.s.
4690	6001994	54	47	0.369422463837495	n.s.
4690	6001998	54	47	0.369422463837495	n.s.
4690	6002013	54	47	0.369422463837495	n.s.
4690	6002021	54	47	0.369422463837495	n.s.
4690	6002025	54	47	0.369422463837495	n.s.
4690	6002030	55	46	0.650952919406731	n.s.

4690	6002041	54	47	0.369422463837495	n.s.
4690	6002053	55	46	0.650952919406731	n.s.
4690	6002056	55	46	0.650952919406731	n.s.
4690	6002065	54	47	0.369422463837495	n.s.
4690	6002068	55	46	0.650952919406731	n.s.
4690	6002069	56	45	1.0116974731171	n.s.
4690	6002084	55	46	0.650952919406731	n.s.
4690	6002087	54	47	0.369422463837495	n.s.
4690	6002109	54	47	0.369422463837495	n.s.
4690	6002114	53	48	0.167106106409387	n.s.
4690	6002123	52	49	0.044003847122408	n.s.
4690	6002124	52	49	0.044003847122408	n.s.
4690	6002126	53	48	0.167106106409387	n.s.
4690	6002131	54	47	0.369422463837495	n.s.
4690	6002151	54	47	0.369422463837495	n.s.
4690	6002156	55	46	0.650952919406731	n.s.
4690	6002165	55	46	0.650952919406731	n.s.
4690	6002169	55	46	0.650952919406731	n.s.
4690	6002186	55	46	0.650952919406731	n.s.
4690	6002206	55	46	0.650952919406731	n.s.
4690	6002221	56	45	1.0116974731171	n.s.
4690	6002225	57	44	1.45165612496859	n.s.
4690	6002226	58	43	1.97082887496121	n.s.
4690	6002231	59	42	2.56921572309496	n.s.
4690	6002245	60	41	3.24681666936984	n.s.
4690	6002248	61	40	4.00363171378585	*
4690	6002254	62	39	4.83966085634298	*
4690	6002263	62	39	4.83966085634298	*
4690	6002279	62	39	4.83966085634298	*
4690	6002292	62	39	4.83966085634298	*
4690	6002293	62	39	4.83966085634298	*
4690	6002303	63	38	5.75490409704125	*
4690	6002307	63	38	5.75490409704125	*
4690	6002312	62	39	4.83966085634298	*
4690	6002332	62	39	4.83966085634298	*
4690	6002340	62	39	4.83966085634298	*
4690	6002346	61	40	4.00363171378585	*
4690	6002347	60	41	3.24681666936984	n.s.
4690	6002348	61	40	4.00363171378585	*
4690	6002351	61	40	4.00363171378585	*
4690	6002353	60	41	3.24681666936984	n.s.
4690	6002356	60	41	3.24681666936984	n.s.
4690	6002357	60	41	3.24681666936984	n.s.
4690	6002371	59	42	2.56921572309496	n.s.
4690	6002379	59	42	2.56921572309496	n.s.
4690	6002381	58	43	1.97082887496121	n.s.
4690	6002389	57	44	1.45165612496859	n.s.
4690	6002395	58	43	1.97082887496121	n.s.
4690	6002398	58	43	1.97082887496121	n.s.
4690	6002404	58	43	1.97082887496121	n.s.

4690	6002412	58	43	1.97082887496121	n.s.
4690	6002414	58	43	1.97082887496121	n.s.
4690	6002420	57	44	1.45165612496859	n.s.
4690	6002427	57	44	1.45165612496859	n.s.
4690	6002432	57	44	1.45165612496859	n.s.
4690	6002435	57	44	1.45165612496859	n.s.
4690	6002442	57	44	1.45165612496859	n.s.
4690	6002457	58	43	1.97082887496121	n.s.
4690	6002479	57	44	1.45165612496859	n.s.
4690	6002493	57	44	1.45165612496859	n.s.
4690	6002515	57	44	1.45165612496859	n.s.
4690	6002527	58	43	1.97082887496121	n.s.
4690	6002551	58	43	1.97082887496121	n.s.
4690	6002555	59	42	2.56921572309496	n.s.
4690	6002576	60	41	3.24681666936984	n.s.
4690	6002577	60	41	3.24681666936984	n.s.
4690	6002578	60	41	3.24681666936984	n.s.
4690	6002590	61	40	4.00363171378585	*
4690	6002600	61	40	4.00363171378585	*
4690	6002618	60	41	3.24681666936984	n.s.
4690	6002623	60	41	3.24681666936984	n.s.
4690	6002635	60	41	3.24681666936984	n.s.
4690	6002672	61	40	4.00363171378585	*
4690	6002673	62	39	4.83966085634298	*
4690	6002678	62	39	4.83966085634298	*
4690	6002687	63	38	5.75490409704125	*
4690	6002693	64	37	6.74936143588064	**
4690	6002717	64	37	6.74936143588064	**
4690	6002741	64	37	6.74936143588064	**
4690	6002743	64	37	6.74936143588064	**
4690	6002760	65	36	7.82303287286116	**
4690	6002771	65	36	7.82303287286116	**
4690	6002772	65	36	7.82303287286116	**
4690	6002811	66	35	8.97591840798281	**
4690	6002818	67	34	10.2080180412456	**
4690	6002822	68	33	11.5193317726495	***
4690	6002832	68	33	11.5193317726495	***
4690	6002924	67	34	10.2080180412456	**
4690	6002930	66	35	8.97591840798281	**
4690	6002932	66	35	8.97591840798281	**
4690	6002934	66	35	8.97591840798281	**
4690	6002938	65	36	7.82303287286116	**
4690	6002940	65	36	7.82303287286116	**
4690	6002942	65	36	7.82303287286116	**
4690	6002955	64	37	6.74936143588064	**
4690	6002976	64	37	6.74936143588064	**
4690	6002992	64	37	6.74936143588064	**
4690	6003029	63	38	5.75490409704125	*
4690	6003049	63	38	5.75490409704125	*
4690	6003050	63	38	5.75490409704125	*

4690	6003053	62	39	4.83966085634298	*
4690	6003054	63	38	5.75490409704125	*
4690	6003057	62	39	4.83966085634298	*
4690	6003058	62	39	4.83966085634298	*
4690	6003067	62	39	4.83966085634298	*
4690	6003073	62	39	4.83966085634298	*
4690	6003076	62	39	4.83966085634298	*
4690	6003077	63	38	5.75490409704125	*
4690	6003078	64	37	6.74936143588064	**
4690	6003079	63	38	5.75490409704125	*
4690	6003082	64	37	6.74936143588064	**
4690	6003094	64	37	6.74936143588064	**
4690	6003100	63	38	5.75490409704125	*
4690	6003106	62	39	4.83966085634298	*
4690	6003110	61	40	4.00363171378585	*
4690	6003115	60	41	3.24681666936984	n.s.
4690	6003118	59	42	2.56921572309496	n.s.
4690	6003136	58	43	1.97082887496121	n.s.
4690	6003139	57	44	1.45165612496859	n.s.
4690	6003144	57	44	1.45165612496859	n.s.
4690	6003145	57	44	1.45165612496859	n.s.
4690	6003157	57	44	1.45165612496859	n.s.
4690	6003180	57	44	1.45165612496859	n.s.
4690	6003181	57	44	1.45165612496859	n.s.
4690	6003187	56	45	1.0116974731171	n.s.
4690	6003189	55	46	0.650952919406731	n.s.
4690	6003206	54	47	0.369422463837495	n.s.
4690	6003218	53	48	0.167106106409387	n.s.
4690	6003229	54	47	0.369422463837495	n.s.
4690	6003230	55	46	0.650952919406731	n.s.
4690	6003233	56	45	1.0116974731171	n.s.
4690	6003235	57	44	1.45165612496859	n.s.
4690	6003242	56	45	1.0116974731171	n.s.
4690	6003244	55	46	0.650952919406731	n.s.
4690	6003250	56	45	1.0116974731171	n.s.
4690	6003256	56	45	1.0116974731171	n.s.
4690	6003293	56	45	1.0116974731171	n.s.
4690	6003297	57	44	1.45165612496859	n.s.
4690	6003307	58	43	1.97082887496121	n.s.
4690	6003314	58	43	1.97082887496121	n.s.
4690	6003321	58	43	1.97082887496121	n.s.
4690	6003325	59	42	2.56921572309496	n.s.
4690	6003357	60	41	3.24681666936984	n.s.
4690	6003370	60	41	3.24681666936984	n.s.
4690	6003393	60	41	3.24681666936984	n.s.
4690	6003445	60	41	3.24681666936984	n.s.
4690	6003455	60	41	3.24681666936984	n.s.
4690	6003468	60	41	3.24681666936984	n.s.
4690	6003482	60	41	3.24681666936984	n.s.
4690	6003516	60	41	3.24681666936984	n.s.

4690	6003517	61	40	4.00363171378585	*
4690	6003518	61	40	4.00363171378585	*
4690	6003526	62	39	4.83966085634298	*
4690	6003531	63	38	5.75490409704125	*
4690	6003560	63	38	5.75490409704125	*
4690	6003568	64	37	6.74936143588064	**
4690	6003595	64	37	6.74936143588064	**
4690	6003601	64	37	6.74936143588064	**
4690	6003607	64	37	6.74936143588064	**
4690	6003615	65	36	7.82303287286116	**
4690	6003631	65	36	7.82303287286116	**
4690	6003650	66	35	8.97591840798281	**
4690	6003679	66	35	8.97591840798281	**
4690	6003693	65	36	7.82303287286116	**
4690	6003699	64	37	6.74936143588064	**
4690	6003710	63	38	5.75490409704125	*
4690	6003715	62	39	4.83966085634298	*
4690	6003724	61	40	4.00363171378585	*
4690	6003728	60	41	3.24681666936984	n.s.
4690	6003734	61	40	4.00363171378585	*
4690	6003797	61	40	4.00363171378585	*
4690	6003815	60	41	3.24681666936984	n.s.
4690	6003843	59	42	2.56921572309496	n.s.
4690	6003851	59	42	2.56921572309496	n.s.
4690	6003854	58	43	1.97082887496121	n.s.
4690	6003872	57	44	1.45165612496859	n.s.
4690	6003910	57	44	1.45165612496859	n.s.
4690	6003913	56	45	1.0116974731171	n.s.
4690	6003933	56	45	1.0116974731171	n.s.
4690	6003947	55	46	0.650952919406731	n.s.
4690	6003964	55	46	0.650952919406731	n.s.
4690	6003965	54	47	0.369422463837495	n.s.
4690	6003980	53	48	0.167106106409387	n.s.
4690	6003994	54	47	0.369422463837495	n.s.
4690	6004086	54	47	0.369422463837495	n.s.
4690	6004088	53	48	0.167106106409387	n.s.
4690	6004103	52	49	0.044003847122408	n.s.
4690	6004114	51	50	0.000115685976557532	n.s.
4690	6004151	51	50	0.000115685976557532	n.s.
4690	6004153	51	50	0.000115685976557532	n.s.
4690	6004186	51	50	0.000115685976557532	n.s.
4690	6004212	50	51	0.0354416229718356	n.s.
4690	6004218	50	51	0.0354416229718356	n.s.
4690	6004225	49	52	0.149981658108242	n.s.
4690	6004245	48	53	0.343735791385777	n.s.
4690	6004260	49	52	0.149981658108242	n.s.
4690	6004263	49	52	0.149981658108242	n.s.
4690	6004270	48	53	0.343735791385777	n.s.
4690	6004276	48	53	0.343735791385777	n.s.
4690	6004289	48	53	0.343735791385777	n.s.

4690	6004291	48	53	0.343735791385777	n.s.
4690	6004295	48	53	0.343735791385777	n.s.
4690	6004332	47	54	0.616704022804441	n.s.
4690	6004337	47	54	0.616704022804441	n.s.
4690	6004339	46	55	0.968886352364233	n.s.
4690	6004341	47	54	0.616704022804441	n.s.
4690	6004345	46	55	0.968886352364233	n.s.
4690	6004346	45	56	1.40028278006515	n.s.
4690	6004348	44	57	1.9108933059072	n.s.
4690	6004353	44	57	1.9108933059072	n.s.
4690	6004355	44	57	1.9108933059072	n.s.
4690	6004362	43	58	2.50071792989038	n.s.
4690	6004365	43	58	2.50071792989038	n.s.
4690	6004366	43	58	2.50071792989038	n.s.
4690	6004367	43	58	2.50071792989038	n.s.
4690	6004377	43	58	2.50071792989038	n.s.
4690	6004386	43	58	2.50071792989038	n.s.
4690	6004390	44	57	1.9108933059072	n.s.
4690	6004403	45	56	1.40028278006515	n.s.
4690	6004411	46	55	0.968886352364233	n.s.
4690	6004424	45	56	1.40028278006515	n.s.
4690	6004427	46	55	0.968886352364233	n.s.
4690	6004455	45	56	1.40028278006515	n.s.
4690	6004465	44	57	1.9108933059072	n.s.
4690	6004488	43	58	2.50071792989038	n.s.
4690	6004491	44	57	1.9108933059072	n.s.
4690	6004494	45	56	1.40028278006515	n.s.
4690	6004510	45	56	1.40028278006515	n.s.
4690	6004578	46	55	0.968886352364233	n.s.
4690	6004596	45	56	1.40028278006515	n.s.
4690	6004624	45	56	1.40028278006515	n.s.
4690	6004625	45	56	1.40028278006515	n.s.
4690	6004638	44	57	1.9108933059072	n.s.
4690	6004650	44	57	1.9108933059072	n.s.
4690	6004657	45	56	1.40028278006515	n.s.
4690	6004658	44	57	1.9108933059072	n.s.
4690	6004769	43	58	2.50071792989038	n.s.
4690	6004773	42	59	3.16975665201469	n.s.
4690	6004787	41	60	3.91800947228012	*
4690	6004789	40	61	4.74547639068669	*
4690	6004841	39	62	5.65215740723438	*
4690	6004846	40	61	4.74547639068669	*
4690	6004847	39	62	5.65215740723438	*
4690	6004854	38	63	6.6380525219232	*
4690	6004861	37	64	7.70316173475315	**
4690	6004889	37	64	7.70316173475315	**
4690	6004890	38	63	6.6380525219232	*
4690	6004892	39	62	5.65215740723438	*
4690	6004897	40	61	4.74547639068669	*
4690	6004902	40	61	4.74547639068669	*

4690	6004911	40	61	4.74547639068669	*
4690	6004915	40	61	4.74547639068669	*
4690	6004924	39	62	5.65215740723438	*
4690	6004951	39	62	5.65215740723438	*
4690	6004958	39	62	5.65215740723438	*
4690	6004985	38	63	6.6380525219232	*
4690	6004988	37	64	7.70316173475315	**
4690	6005000	36	65	8.84748504572423	**
4690	6005006	35	66	10.0710224548364	**
4690	6005011	34	67	11.3737739620898	***
4690	6005021	33	68	12.7557395674842	***
4690	6005037	32	69	14.2169192710198	***
4690	6005047	32	69	14.2169192710198	***
4690	6005057	32	69	14.2169192710198	***
4690	6005061	32	69	14.2169192710198	***
4690	6005070	32	69	14.2169192710198	***
4690	6005082	32	69	14.2169192710198	***
4690	6005103	32	69	14.2169192710198	***
4690	6005136	33	68	12.7557395674842	***
4690	6005137	33	68	12.7557395674842	***
4690	6005139	32	69	14.2169192710198	***
4690	6005148	33	68	12.7557395674842	***
4690	6005180	34	67	11.3737739620898	***
4690	6005184	34	67	11.3737739620898	***
4690	6005185	33	68	12.7557395674842	***
4690	6005200	33	68	12.7557395674842	***
4690	6005204	33	68	12.7557395674842	***
4690	6005205	32	69	14.2169192710198	***
4690	6005217	32	69	14.2169192710198	***
4690	6005226	32	69	14.2169192710198	***
4690	6005227	32	69	14.2169192710198	***
4690	6005247	32	69	14.2169192710198	***
4690	6005312	33	68	12.7557395674842	***
4690	6005315	33	68	12.7557395674842	***
4690	6005327	33	68	12.7557395674842	***
4690	6005334	32	69	14.2169192710198	***
4690	6005335	32	69	14.2169192710198	***
4690	6005355	33	68	12.7557395674842	***
4690	6005356	34	67	11.3737739620898	***
4690	6005367	34	67	11.3737739620898	***
4690	6005378	35	66	10.0710224548364	**
4690	6005389	34	67	11.3737739620898	***
4690	6005393	35	66	10.0710224548364	**
4690	6005395	35	66	10.0710224548364	**
4690	6005400	35	66	10.0710224548364	**
4690	6005404	36	65	8.84748504572423	**
4690	6005411	36	65	8.84748504572423	**
4690	6005418	35	66	10.0710224548364	**
4690	6005423	35	66	10.0710224548364	**
4690	6005437	35	66	10.0710224548364	**

4690	6005444	36	65	8.84748504572423	**
4690	6005446	37	64	7.70316173475315	**
4690	6005505	38	63	6.6380525219232	*
4690	6005536	38	63	6.6380525219232	*
4690	6005538	38	63	6.6380525219232	*
4690	6005547	39	62	5.65215740723438	*
4690	6005549	39	62	5.65215740723438	*
4690	6005560	39	62	5.65215740723438	*
4690	6005568	39	62	5.65215740723438	*
4690	6005771	39	62	5.65215740723438	*
4690	6005803	38	63	6.6380525219232	*
4690	6005810	39	62	5.65215740723438	*
4690	6005815	39	62	5.65215740723438	*
4690	6005862	39	62	5.65215740723438	*
4690	6005885	39	62	5.65215740723438	*
4690	6005886	39	62	5.65215740723438	*
4690	6005902	39	62	5.65215740723438	*
4690	6005906	40	61	4.74547639068669	*
4690	6005930	40	61	4.74547639068669	*
4690	6005959	40	61	4.74547639068669	*
4690	6005974	40	61	4.74547639068669	*
4690	6005981	41	60	3.91800947228012	*
4690	6006014	40	61	4.74547639068669	*
4690	6006020	41	60	3.91800947228012	*
4690	6006038	41	60	3.91800947228012	*
4690	6006044	42	59	3.16975665201469	n.s.
4690	6006079	42	59	3.16975665201469	n.s.
4690	6006091	42	59	3.16975665201469	n.s.
4690	6006094	42	59	3.16975665201469	n.s.
4690	6006109	42	59	3.16975665201469	n.s.
4690	6006113	43	58	2.50071792989038	n.s.
4690	6006114	43	58	2.50071792989038	n.s.
4690	6006120	42	59	3.16975665201469	n.s.
4690	6006121	42	59	3.16975665201469	n.s.
4690	6006122	42	59	3.16975665201469	n.s.
4690	6006151	41	60	3.91800947228012	*
4690	6006163	42	59	3.16975665201469	n.s.
4690	6006166	42	59	3.16975665201469	n.s.
4690	6006167	42	59	3.16975665201469	n.s.
4690	6006179	42	59	3.16975665201469	n.s.
4690	6006183	42	59	3.16975665201469	n.s.
4690	6006199	42	59	3.16975665201469	n.s.
4690	6006203	41	60	3.91800947228012	*
4690	6006211	41	60	3.91800947228012	*
4690	6006229	41	60	3.91800947228012	*
4690	6006236	41	60	3.91800947228012	*
4690	6006243	41	60	3.91800947228012	*
4690	6006253	40	61	4.74547639068669	*
4690	6006259	40	61	4.74547639068669	*
4690	6006269	40	61	4.74547639068669	*

4690	6006271	39	62	5.65215740723438	*	
4690	6006287	38	63	6.6380525219232	*	
4690	6006291	38	63	6.6380525219232	*	
4690	6006320	39	62	5.65215740723438	*	
4690	6006321	39	62	5.65215740723438	*	
4690	6006346	39	62	5.65215740723438	*	
4690	6006347	39	62	5.65215740723438	*	
4690	6006369	39	62	5.65215740723438	*	
4690	6006371	39	62	5.65215740723438	*	
4690	6006376	40	61	4.74547639068669	*	
4690	6006390	41	60	3.91800947228012	*	
4690	6006391	42	59	3.16975665201469	n.s.	
4690	6006394	43	58	2.50071792989038	n.s.	
4690	6006424	43	58	2.50071792989038	n.s.	
4690	6006433	43	58	2.50071792989038	n.s.	
4690	6006436	43	58	2.50071792989038	n.s.	
4690	6006441	44	57	1.9108933059072	n.s.	
4690	6006450	44	57	1.9108933059072	n.s.	
4690	6006459	44	57	1.9108933059072	n.s.	
4690	6006462	44	57	1.9108933059072	n.s.	
4690	6006466	45	56	1.40028278006515	n.s.	
4690	6006467	46	55	0.968886352364233	n.s.	
4690	6006468	46	55	0.968886352364233	n.s.	
4690	6006469	46	55	0.968886352364233	n.s.	
4690	6006471	47	54	0.616704022804441	n.s.	
4690	6006475	48	53	0.343735791385777	n.s.	
4690	6006478	49	52	0.149981658108242	n.s.	
4690	6006494	50	51	0.0354416229718356	n.s.	
4690	6006503	51	50	0.000115685976557532		n.s.
4690	6006505	52	49	0.044003847122408	n.s.	
4690	6006508	52	49	0.044003847122408	n.s.	
4690	6006517	53	48	0.167106106409387	n.s.	
4690	6006520	54	47	0.369422463837495	n.s.	
4690	6006529	54	47	0.369422463837495	n.s.	
4690	6006531	55	46	0.650952919406731	n.s.	
4690	6006541	55	46	0.650952919406731	n.s.	
4690	6006567	56	45	1.0116974731171	n.s.	
4690	6006580	57	44	1.45165612496859	n.s.	
4690	6006582	57	44	1.45165612496859	n.s.	
4690	6006594	57	44	1.45165612496859	n.s.	
4690	6006607	58	43	1.97082887496121	n.s.	
4690	6006610	58	43	1.97082887496121	n.s.	
4690	6006644	59	42	2.56921572309496	n.s.	
4690	6006679	59	42	2.56921572309496	n.s.	
4690	6006694	60	41	3.24681666936984	n.s.	
4690	6006695	61	40	4.00363171378585	*	
4690	6006700	61	40	4.00363171378585	*	
4690	6006708	61	40	4.00363171378585	*	
4690	6006710	62	39	4.83966085634298	*	
4690	6006717	63	38	5.75490409704125	*	

4690	6006726	64	37	6.74936143588064	**
4690	6006734	64	37	6.74936143588064	**
4690	6006738	64	37	6.74936143588064	**
4690	6006742	64	37	6.74936143588064	**
4690	6006750	65	36	7.82303287286116	**
4690	6006752	66	35	8.97591840798281	**
4690	6006758	66	35	8.97591840798281	**
4690	6006760	66	35	8.97591840798281	**
4690	6006776	67	34	10.2080180412456	**
4690	6006783	68	33	11.5193317726495	***
4690	6006791	69	32	12.9098596021945	***
4690	6006793	70	31	14.3796015298807	***
4690	6006794	70	31	14.3796015298807	***
4690	6006804	70	31	14.3796015298807	***
4690	6006811	70	31	14.3796015298807	***
4690	6006833	70	31	14.3796015298807	***
4690	6006837	70	31	14.3796015298807	***
4690	6006855	71	30	15.928557555708	***
4690	6006866	71	30	15.928557555708	***
4690	6006868	70	31	14.3796015298807	***
4690	6006871	70	31	14.3796015298807	***
4690	6006878	69	32	12.9098596021945	***
4690	6006880	68	33	11.5193317726495	***
4690	6006881	69	32	12.9098596021945	***
4690	6006882	69	32	12.9098596021945	***
4690	6006884	69	32	12.9098596021945	***
4690	6006886	68	33	11.5193317726495	***
4690	6006888	68	33	11.5193317726495	***
4690	6006897	67	34	10.2080180412456	**
4690	6006899	67	34	10.2080180412456	**
4690	6006901	66	35	8.97591840798281	**
4690	6006902	66	35	8.97591840798281	**
4690	6006903	65	36	7.82303287286116	**
4690	6006906	65	36	7.82303287286116	**
4690	6006907	66	35	8.97591840798281	**
4690	6006909	66	35	8.97591840798281	**
4690	6006910	66	35	8.97591840798281	**
4690	6006911	65	36	7.82303287286116	**
4690	6006913	65	36	7.82303287286116	**
4690	6006915	66	35	8.97591840798281	**
4690	6006916	66	35	8.97591840798281	**
4690	6006917	67	34	10.2080180412456	**
4690	6006918	67	34	10.2080180412456	**
4690	6006919	67	34	10.2080180412456	**
4690	6006920	68	33	11.5193317726495	***
4690	6006921	69	32	12.9098596021945	***
4690	6006922	69	32	12.9098596021945	***
4690	6006924	70	31	14.3796015298807	***
4690	6006926	70	31	14.3796015298807	***
4690	6006927	69	32	12.9098596021945	***

4690	6006928	68	33	11.5193317726495	***
4690	6006930	68	33	11.5193317726495	***
4690	6006932	68	33	11.5193317726495	***
4690	6006933	68	33	11.5193317726495	***
4690	6006934	68	33	11.5193317726495	***
4690	6006936	68	33	11.5193317726495	***
4690	6006937	68	33	11.5193317726495	***
4690	6006939	68	33	11.5193317726495	***
4690	6006942	68	33	11.5193317726495	***
4690	6006950	69	32	12.9098596021945	***
4690	6006953	69	32	12.9098596021945	***
4690	6006963	69	32	12.9098596021945	***
4690	6006969	68	33	11.5193317726495	***
4690	6006975	68	33	11.5193317726495	***
4690	6006981	68	33	11.5193317726495	***
4690	6006986	68	33	11.5193317726495	***
4690	6006998	68	33	11.5193317726495	***
4690	6006999	67	34	10.2080180412456	**
4690	6007003	67	34	10.2080180412456	**
4690	6007020	67	34	10.2080180412456	**
4690	6007023	67	34	10.2080180412456	**
4690	6007035	67	34	10.2080180412456	**
4690	6007050	66	35	8.97591840798281	**
4690	6007066	66	35	8.97591840798281	**
4690	6007071	65	36	7.82303287286116	**
4690	6007089	64	37	6.74936143588064	**
4690	6007094	64	37	6.74936143588064	**
4690	6007095	64	37	6.74936143588064	**
4690	6007099	64	37	6.74936143588064	**
4690	6007118	63	38	5.75490409704125	*
4690	6007120	62	39	4.83966085634298	*
4690	6007133	61	40	4.00363171378585	*
4690	6007134	60	41	3.24681666936984	n.s.
4690	6007155	60	41	3.24681666936984	n.s.
4690	6007156	60	41	3.24681666936984	n.s.
4690	6007160	60	41	3.24681666936984	n.s.
4690	6007161	59	42	2.56921572309496	n.s.
4690	6007176	58	43	1.97082887496121	n.s.
4690	6007179	58	43	1.97082887496121	n.s.
4690	6007206	58	43	1.97082887496121	n.s.
4690	6007209	57	44	1.45165612496859	n.s.
4690	6007223	56	45	1.0116974731171	n.s.
4690	6007224	56	45	1.0116974731171	n.s.
4690	6007244	56	45	1.0116974731171	n.s.
4690	6007250	55	46	0.650952919406731	n.s.
4690	6007270	54	47	0.369422463837495	n.s.
4690	6007285	53	48	0.167106106409387	n.s.
4690	6007291	53	48	0.167106106409387	n.s.
4690	6007298	52	49	0.044003847122408	n.s.
4690	6007305	51	50	0.000115685976557532	n.s.

4690	6007312	50	51	0.0354416229718356	n.s.
4690	6007313	50	51	0.0354416229718356	n.s.
4690	6007319	50	51	0.0354416229718356	n.s.
4690	6007321	49	52	0.149981658108242	n.s.
4690	6007345	49	52	0.149981658108242	n.s.
4690	6007346	49	52	0.149981658108242	n.s.
4690	6007354	49	52	0.149981658108242	n.s.
4690	6007355	49	52	0.149981658108242	n.s.
4690	6007371	48	53	0.343735791385777	n.s.
4690	6007389	47	54	0.616704022804441	n.s.
4690	6007424	46	55	0.968886352364233	n.s.
4690	6007432	45	56	1.40028278006515	n.s.
4690	6007435	44	57	1.9108933059072	n.s.
4690	6007438	44	57	1.9108933059072	n.s.
4690	6007439	45	56	1.40028278006515	n.s.
4690	6007448	45	56	1.40028278006515	n.s.
4690	6007457	45	56	1.40028278006515	n.s.
4690	6007458	45	56	1.40028278006515	n.s.
4690	6007464	45	56	1.40028278006515	n.s.
4690	6007468	45	56	1.40028278006515	n.s.
4690	6007478	46	55	0.968886352364233	n.s.
4690	6007490	46	55	0.968886352364233	n.s.
4690	6007493	47	54	0.616704022804441	n.s.
4690	6007496	48	53	0.343735791385777	n.s.
4690	6007519	48	53	0.343735791385777	n.s.
4690	6007523	48	53	0.343735791385777	n.s.
4690	6007537	48	53	0.343735791385777	n.s.
4690	6007548	48	53	0.343735791385777	n.s.
4690	6007550	47	54	0.616704022804441	n.s.
4690	6007566	47	54	0.616704022804441	n.s.
4690	6007567	47	54	0.616704022804441	n.s.
4690	6007579	48	53	0.343735791385777	n.s.
4690	6007582	48	53	0.343735791385777	n.s.
4690	6007584	49	52	0.149981658108242	n.s.
4690	6007588	49	52	0.149981658108242	n.s.
4690	6007593	49	52	0.149981658108242	n.s.
4690	6007599	50	51	0.0354416229718356	n.s.
4690	6007600	51	50	0.000115685976557532	n.s.
4690	6007615	52	49	0.044003847122408	n.s.
4690	6007627	53	48	0.167106106409387	n.s.
4690	6007644	52	49	0.044003847122408	n.s.
4690	6007658	53	48	0.167106106409387	n.s.
4690	6007659	53	48	0.167106106409387	n.s.
4690	6007662	54	47	0.369422463837495	n.s.
4690	6007672	55	46	0.650952919406731	n.s.
4690	6007682	55	46	0.650952919406731	n.s.
4690	6007694	55	46	0.650952919406731	n.s.
4690	6007696	56	45	1.0116974731171	n.s.
4690	6007698	56	45	1.0116974731171	n.s.
4690	6007718	57	44	1.45165612496859	n.s.

4690	6007720	57	44	1.45165612496859	n.s.
4690	6007732	58	43	1.97082887496121	n.s.
4690	6007739	59	42	2.56921572309496	n.s.
4690	6007746	59	42	2.56921572309496	n.s.
4690	6007747	59	42	2.56921572309496	n.s.
4690	6007751	59	42	2.56921572309496	n.s.
4690	6007778	59	42	2.56921572309496	n.s.
4690	6007804	59	42	2.56921572309496	n.s.
4690	6007824	60	41	3.24681666936984	n.s.
4690	6007828	61	40	4.00363171378585	*
4690	6007843	61	40	4.00363171378585	*
4690	6007845	61	40	4.00363171378585	*
4690	6007847	60	41	3.24681666936984	n.s.
4690	6007849	61	40	4.00363171378585	*
4690	6007855	61	40	4.00363171378585	*
4690	6007863	61	40	4.00363171378585	*
4690	6007895	61	40	4.00363171378585	*
4690	6007976	61	40	4.00363171378585	*
4690	6007998	61	40	4.00363171378585	*
4690	6008001	61	40	4.00363171378585	*
4690	6008026	62	39	4.83966085634298	*
4690	6008027	61	40	4.00363171378585	*
4690	6008030	60	41	3.24681666936984	n.s.
4690	6008036	61	40	4.00363171378585	*
4690	6008058	61	40	4.00363171378585	*
4690	6008059	61	40	4.00363171378585	*
4690	6008063	62	39	4.83966085634298	*
4690	6008066	62	39	4.83966085634298	*
4690	6008107	61	40	4.00363171378585	*
4690	6008112	60	41	3.24681666936984	n.s.
4690	6008113	60	41	3.24681666936984	n.s.
4690	6008127	60	41	3.24681666936984	n.s.
4690	6008147	60	41	3.24681666936984	n.s.
4690	6008151	61	40	4.00363171378585	*
4690	6008155	61	40	4.00363171378585	*
4690	6008158	60	41	3.24681666936984	n.s.
4690	6008160	60	41	3.24681666936984	n.s.
4690	6008200	61	40	4.00363171378585	*
4690	6008214	61	40	4.00363171378585	*
4690	6008218	60	41	3.24681666936984	n.s.
4690	6008244	60	41	3.24681666936984	n.s.
4690	6008246	60	41	3.24681666936984	n.s.
4690	6008250	60	41	3.24681666936984	n.s.
4690	6008254	59	42	2.56921572309496	n.s.
4690	6008256	59	42	2.56921572309496	n.s.
4690	6008260	60	41	3.24681666936984	n.s.
4690	6008262	60	41	3.24681666936984	n.s.
4690	6008311	60	41	3.24681666936984	n.s.
4690	6008319	59	42	2.56921572309496	n.s.
4690	6008322	59	42	2.56921572309496	n.s.

4690	6008336	59	42	2.56921572309496	n.s.
4690	6008338	59	42	2.56921572309496	n.s.
4690	6008380	58	43	1.97082887496121	n.s.
4690	6008387	58	43	1.97082887496121	n.s.
4690	6008388	59	42	2.56921572309496	n.s.
4690	6008389	59	42	2.56921572309496	n.s.
4690	6008391	59	42	2.56921572309496	n.s.
4690	6008394	59	42	2.56921572309496	n.s.
4690	6008398	59	42	2.56921572309496	n.s.
4690	6008399	59	42	2.56921572309496	n.s.
4690	6008433	59	42	2.56921572309496	n.s.
4690	6008468	59	42	2.56921572309496	n.s.
4690	6008490	59	42	2.56921572309496	n.s.
4690	6008493	60	41	3.24681666936984	n.s.
4690	6008510	59	42	2.56921572309496	n.s.
4690	6008511	58	43	1.97082887496121	n.s.
4690	6008520	58	43	1.97082887496121	n.s.
4690	6008525	58	43	1.97082887496121	n.s.
4690	6008545	58	43	1.97082887496121	n.s.
4690	6008557	57	44	1.45165612496859	n.s.
4690	6008560	56	45	1.0116974731171	n.s.
4690	6008565	56	45	1.0116974731171	n.s.
4690	6008594	56	45	1.0116974731171	n.s.
4690	6008602	55	46	0.650952919406731	n.s.
4690	6008622	54	47	0.369422463837495	n.s.
4690	6008633	54	47	0.369422463837495	n.s.
4690	6008640	53	48	0.167106106409387	n.s.
4690	6008654	54	47	0.369422463837495	n.s.
4690	6008664	54	47	0.369422463837495	n.s.
4690	6008670	55	46	0.650952919406731	n.s.
4690	6008673	55	46	0.650952919406731	n.s.
4690	6008688	55	46	0.650952919406731	n.s.
4690	6008692	54	47	0.369422463837495	n.s.
4690	6008701	53	48	0.167106106409387	n.s.
4690	6008706	52	49	0.044003847122408	n.s.
4690	6008740	53	48	0.167106106409387	n.s.
4690	6008741	53	48	0.167106106409387	n.s.
4690	6008747	52	49	0.044003847122408	n.s.
4690	6008757	52	49	0.044003847122408	n.s.
4690	6008762	51	50	0.000115685976557532	n.s.
4690	6008766	50	51	0.0354416229718356	n.s.
4690	6008770	50	51	0.0354416229718356	n.s.
4690	6008772	50	51	0.0354416229718356	n.s.
4690	6008776	49	52	0.149981658108242	n.s.
4690	6008777	48	53	0.343735791385777	n.s.
4690	6008787	47	54	0.616704022804441	n.s.
4690	6008796	46	55	0.968886352364233	n.s.
4690	6008798	45	56	1.40028278006515	n.s.
4690	6008800	44	57	1.9108933059072	n.s.
4690	6008805	43	58	2.50071792989038	n.s.

4690	6008822	43	58	2.50071792989038	n.s.
4690	6008826	43	58	2.50071792989038	n.s.
4690	6008842	42	59	3.16975665201469	n.s.
4690	6008858	42	59	3.16975665201469	n.s.
4690	6008923	43	58	2.50071792989038	n.s.
4690	6008945	42	59	3.16975665201469	n.s.
4690	6008947	41	60	3.91800947228012	*
4690	6008952	41	60	3.91800947228012	*
4690	6008953	40	61	4.74547639068669	*
4690	6008959	39	62	5.65215740723438	*
4690	6008980	39	62	5.65215740723438	*
4690	6009002	38	63	6.6380525219232	*
4690	6009026	38	63	6.6380525219232	*
4690	6009032	38	63	6.6380525219232	*
4690	6009035	38	63	6.6380525219232	*
4690	6009054	38	63	6.6380525219232	*
4690	6009055	38	63	6.6380525219232	*
4690	6009076	38	63	6.6380525219232	*
4690	6009100	39	62	5.65215740723438	*
4690	6009113	40	61	4.74547639068669	*
4690	6009118	41	60	3.91800947228012	*
4690	6009124	40	61	4.74547639068669	*
4690	6009144	41	60	3.91800947228012	*
4690	6009174	42	59	3.16975665201469	n.s.
4690	6009176	41	60	3.91800947228012	*
4690	6009182	40	61	4.74547639068669	*
4690	6009211	40	61	4.74547639068669	*
4690	6009314	40	61	4.74547639068669	*
4690	6009323	39	62	5.65215740723438	*
4690	6009334	40	61	4.74547639068669	*
4690	6009341	40	61	4.74547639068669	*
4690	6009372	40	61	4.74547639068669	*
4690	6009374	40	61	4.74547639068669	*
4690	6009382	40	61	4.74547639068669	*
4690	6009385	39	62	5.65215740723438	*
4690	6009387	39	62	5.65215740723438	*
4690	6009389	39	62	5.65215740723438	*
4690	6009404	39	62	5.65215740723438	*
4690	6009405	39	62	5.65215740723438	*
4690	6009406	39	62	5.65215740723438	*
4690	6009507	40	61	4.74547639068669	*
4690	6009550	40	61	4.74547639068669	*
4690	6009556	41	60	3.91800947228012	*
4690	6009563	41	60	3.91800947228012	*
4690	6009564	42	59	3.16975665201469	n.s.
4690	6009569	41	60	3.91800947228012	*
4690	6009577	40	61	4.74547639068669	*
4690	6009591	40	61	4.74547639068669	*
4690	6009593	40	61	4.74547639068669	*
4690	6009601	40	61	4.74547639068669	*

4690	6009606	41	60	3.91800947228012	*
4690	6009607	41	60	3.91800947228012	*
4690	6009614	41	60	3.91800947228012	*
4690	6009621	42	59	3.16975665201469	n.s.
4690	6009626	42	59	3.16975665201469	n.s.
4690	6009634	42	59	3.16975665201469	n.s.
4690	6009653	41	60	3.91800947228012	*
4690	6009676	40	61	4.74547639068669	*
4690	6009687	39	62	5.65215740723438	*
4690	6009715	39	62	5.65215740723438	*
4690	6009718	40	61	4.74547639068669	*
4690	6009729	40	61	4.74547639068669	*
4690	6009736	41	60	3.91800947228012	*
4690	6009739	42	59	3.16975665201469	n.s.
4690	6009742	42	59	3.16975665201469	n.s.
4690	6009749	43	58	2.50071792989038	n.s.
4690	6009756	44	57	1.9108933059072	n.s.
4690	6009759	45	56	1.40028278006515	n.s.
4690	6009760	46	55	0.968886352364233	n.s.
4690	6009767	47	54	0.616704022804441	n.s.
4690	6009768	48	53	0.343735791385777	n.s.
4690	6009778	49	52	0.149981658108242	n.s.
4690	6009782	49	52	0.149981658108242	n.s.
4690	6009785	49	52	0.149981658108242	n.s.
4690	6009787	50	51	0.0354416229718356	n.s.
4690	6009789	51	50	0.000115685976557532	n.s.
4690	6009814	50	51	0.0354416229718356	n.s.
4690	6009833	50	51	0.0354416229718356	n.s.
4690	6009835	50	51	0.0354416229718356	n.s.
4690	6009840	51	50	0.000115685976557532	n.s.
4690	6009864	51	50	0.000115685976557532	n.s.
4690	6009866	51	50	0.000115685976557532	n.s.
4690	6009871	51	50	0.000115685976557532	n.s.
4690	6009881	51	50	0.000115685976557532	n.s.
4690	6009885	51	50	0.000115685976557532	n.s.
4690	6009887	52	49	0.044003847122408	n.s.
4690	6009890	51	50	0.000115685976557532	n.s.
4690	6009909	50	51	0.0354416229718356	n.s.
4690	6009913	50	51	0.0354416229718356	n.s.
4690	6009998	49	52	0.149981658108242	n.s.
4690	6010018	49	52	0.149981658108242	n.s.
4690	6010040	49	52	0.149981658108242	n.s.
4690	6010052	50	51	0.0354416229718356	n.s.
4690	6010058	49	52	0.149981658108242	n.s.
4690	6010076	49	52	0.149981658108242	n.s.
4690	6010087	49	52	0.149981658108242	n.s.
4690	6010108	50	51	0.0354416229718356	n.s.
4690	6010141	51	50	0.000115685976557532	n.s.
4690	6010152	52	49	0.044003847122408	n.s.
4690	6010170	52	49	0.044003847122408	n.s.

4690	6010172	53	48	0.167106106409387	n.s.
4690	6010181	52	49	0.044003847122408	n.s.
4690	6010252	52	49	0.044003847122408	n.s.
4690	6010255	53	48	0.167106106409387	n.s.
4690	6010257	53	48	0.167106106409387	n.s.
4690	6010258	53	48	0.167106106409387	n.s.
4690	6010286	53	48	0.167106106409387	n.s.
4690	6010295	53	48	0.167106106409387	n.s.
4690	6010327	52	49	0.044003847122408	n.s.
4690	6010336	53	48	0.167106106409387	n.s.
4690	6010337	53	48	0.167106106409387	n.s.
4690	6010357	53	48	0.167106106409387	n.s.
4690	6010358	53	48	0.167106106409387	n.s.
4690	6010372	52	49	0.044003847122408	n.s.
4690	6010373	52	49	0.044003847122408	n.s.
4690	6010377	51	50	0.000115685976557532	n.s.
4690	6010378	51	50	0.000115685976557532	n.s.
4690	6010380	51	50	0.000115685976557532	n.s.
4690	6010385	51	50	0.000115685976557532	n.s.
4690	6010387	51	50	0.000115685976557532	n.s.
4690	6010395	51	50	0.000115685976557532	n.s.
4690	6010404	51	50	0.000115685976557532	n.s.
4690	6010414	51	50	0.000115685976557532	n.s.
4690	6010423	50	51	0.0354416229718356	n.s.
4690	6010427	49	52	0.149981658108242	n.s.
4690	6010428	49	52	0.149981658108242	n.s.
4690	6010434	49	52	0.149981658108242	n.s.
4690	6010448	50	51	0.0354416229718356	n.s.
4690	6010487	49	52	0.149981658108242	n.s.
4690	6010499	50	51	0.0354416229718356	n.s.
4690	6010502	49	52	0.149981658108242	n.s.
4690	6010532	49	52	0.149981658108242	n.s.
4690	6010551	50	51	0.0354416229718356	n.s.
4690	6010553	51	50	0.000115685976557532	n.s.
4690	6010563	52	49	0.044003847122408	n.s.
4690	6010594	53	48	0.167106106409387	n.s.
4690	6010603	52	49	0.044003847122408	n.s.
4690	6010626	53	48	0.167106106409387	n.s.
4690	6010629	53	48	0.167106106409387	n.s.
4690	6010641	53	48	0.167106106409387	n.s.
4690	6010656	54	47	0.369422463837495	n.s.
4690	6010662	54	47	0.369422463837495	n.s.
4690	6010673	54	47	0.369422463837495	n.s.
4690	6010674	54	47	0.369422463837495	n.s.
4690	6010678	55	46	0.650952919406731	n.s.
4690	6010686	55	46	0.650952919406731	n.s.
4690	6010690	55	46	0.650952919406731	n.s.
4690	6010695	56	45	1.0116974731171	n.s.
4690	6010713	57	44	1.45165612496859	n.s.
4690	6010723	58	43	1.97082887496121	n.s.

4690	6010726	58	43	1.97082887496121	n.s.
4690	6010730	58	43	1.97082887496121	n.s.
4690	6010733	59	42	2.56921572309496	n.s.
4690	6010735	60	41	3.24681666936984	n.s.
4690	6010738	59	42	2.56921572309496	n.s.
4690	6010750	58	43	1.97082887496121	n.s.
4690	6010753	58	43	1.97082887496121	n.s.
4690	6010759	58	43	1.97082887496121	n.s.
4690	6010771	59	42	2.56921572309496	n.s.
4690	6010773	60	41	3.24681666936984	n.s.
4690	6010784	59	42	2.56921572309496	n.s.
4690	6010789	58	43	1.97082887496121	n.s.
4690	6010794	59	42	2.56921572309496	n.s.
4690	6010799	59	42	2.56921572309496	n.s.
4690	6010812	59	42	2.56921572309496	n.s.
4690	6010813	58	43	1.97082887496121	n.s.
4690	6010816	58	43	1.97082887496121	n.s.
4690	6010817	58	43	1.97082887496121	n.s.
4690	6010818	58	43	1.97082887496121	n.s.
4690	6010824	58	43	1.97082887496121	n.s.
4690	6010825	58	43	1.97082887496121	n.s.
4690	6010827	58	43	1.97082887496121	n.s.
4690	6010856	58	43	1.97082887496121	n.s.
4690	6010864	57	44	1.45165612496859	n.s.
4690	6010872	57	44	1.45165612496859	n.s.
4690	6010885	56	45	1.0116974731171	n.s.
4690	6010907	55	46	0.650952919406731	n.s.
4690	6010915	55	46	0.650952919406731	n.s.
4690	6010922	55	46	0.650952919406731	n.s.
4690	6010923	54	47	0.369422463837495	n.s.
4690	6010925	53	48	0.167106106409387	n.s.
4690	6010940	52	49	0.044003847122408	n.s.
4690	6010953	53	48	0.167106106409387	n.s.
4690	6010980	54	47	0.369422463837495	n.s.
4690	6010985	55	46	0.650952919406731	n.s.
4690	6010992	55	46	0.650952919406731	n.s.
4690	6011002	54	47	0.369422463837495	n.s.
4690	6011015	54	47	0.369422463837495	n.s.
4690	6011022	54	47	0.369422463837495	n.s.
4690	6011028	55	46	0.650952919406731	n.s.
4690	6011033	55	46	0.650952919406731	n.s.
4690	6011047	56	45	1.0116974731171	n.s.
4690	6011084	57	44	1.45165612496859	n.s.
4690	6011090	57	44	1.45165612496859	n.s.
4690	6011093	57	44	1.45165612496859	n.s.
4690	6011101	57	44	1.45165612496859	n.s.
4690	6011105	57	44	1.45165612496859	n.s.
4690	6011111	57	44	1.45165612496859	n.s.
4690	6011114	57	44	1.45165612496859	n.s.
4690	6011130	56	45	1.0116974731171	n.s.

4690	6011131	57	44	1.45165612496859	n.s.
4690	6011163	57	44	1.45165612496859	n.s.
4690	6011174	57	44	1.45165612496859	n.s.
4690	6011175	58	43	1.97082887496121	n.s.
4690	6011198	58	43	1.97082887496121	n.s.
4690	6011203	57	44	1.45165612496859	n.s.
4690	6011205	56	45	1.0116974731171	n.s.
4690	6011227	57	44	1.45165612496859	n.s.
4690	6011230	57	44	1.45165612496859	n.s.
4690	6011233	58	43	1.97082887496121	n.s.
4690	6011238	58	43	1.97082887496121	n.s.
4690	6011251	59	42	2.56921572309496	n.s.
4690	6011259	59	42	2.56921572309496	n.s.
4690	6011260	60	41	3.24681666936984	n.s.
4690	6011265	60	41	3.24681666936984	n.s.
4690	6011275	61	40	4.00363171378585	*
4690	6011277	62	39	4.83966085634298	*
4690	6011281	61	40	4.00363171378585	*
4690	6011285	61	40	4.00363171378585	*
4690	6011286	61	40	4.00363171378585	*
4690	6011291	61	40	4.00363171378585	*
4690	6011464	61	40	4.00363171378585	*
4690	6011489	60	41	3.24681666936984	n.s.
4690	6011505	61	40	4.00363171378585	*
4690	6011514	62	39	4.83966085634298	*
4690	6011523	63	38	5.75490409704125	*
4690	6011527	64	37	6.74936143588064	**
4690	6011532	65	36	7.82303287286116	**
4690	6011549	64	37	6.74936143588064	**
4690	6011558	64	37	6.74936143588064	**
4690	6011586	63	38	5.75490409704125	*
4690	6011596	64	37	6.74936143588064	**
4690	6011645	65	36	7.82303287286116	**
4690	6011655	64	37	6.74936143588064	**
4690	6011668	63	38	5.75490409704125	*
4690	6011730	63	38	5.75490409704125	*
4690	6011735	63	38	5.75490409704125	*
4690	6011743	63	38	5.75490409704125	*
4690	6011751	63	38	5.75490409704125	*
4690	6011763	62	39	4.83966085634298	*
4690	6011806	62	39	4.83966085634298	*
4690	6011807	62	39	4.83966085634298	*
4690	6011819	61	40	4.00363171378585	*
4690	6011822	60	41	3.24681666936984	n.s.
4690	6011837	60	41	3.24681666936984	n.s.
4690	6011865	59	42	2.56921572309496	n.s.
4690	6011866	58	43	1.97082887496121	n.s.
4690	6011957	59	42	2.56921572309496	n.s.
4690	6011958	59	42	2.56921572309496	n.s.
4690	6011964	59	42	2.56921572309496	n.s.

4690	6011983	59	42	2.56921572309496	n.s.
4690	6011987	60	41	3.24681666936984	n.s.
4690	6012005	59	42	2.56921572309496	n.s.
4690	6012010	59	42	2.56921572309496	n.s.
4690	6012046	58	43	1.97082887496121	n.s.
4690	6012056	59	42	2.56921572309496	n.s.
4690	6012096	60	41	3.24681666936984	n.s.
4690	6012109	59	42	2.56921572309496	n.s.
4690	6012112	60	41	3.24681666936984	n.s.
4690	6012113	60	41	3.24681666936984	n.s.
4690	6012141	60	41	3.24681666936984	n.s.
4690	6012143	60	41	3.24681666936984	n.s.
4690	6012144	61	40	4.00363171378585	*
4690	6012149	60	41	3.24681666936984	n.s.
4690	6012207	60	41	3.24681666936984	n.s.
4690	6012277	60	41	3.24681666936984	n.s.
4690	6012282	61	40	4.00363171378585	*
4690	6012288	60	41	3.24681666936984	n.s.
4690	6012293	59	42	2.56921572309496	n.s.
4690	6012317	58	43	1.97082887496121	n.s.
4690	6012321	57	44	1.45165612496859	n.s.
4690	6012413	57	44	1.45165612496859	n.s.
4690	6012414	56	45	1.0116974731171	n.s.
4690	6012421	55	46	0.650952919406731	n.s.
4690	6012442	55	46	0.650952919406731	n.s.
4690	6012444	54	47	0.369422463837495	n.s.
4690	6012470	54	47	0.369422463837495	n.s.
4690	6012471	54	47	0.369422463837495	n.s.
4690	6012492	55	46	0.650952919406731	n.s.
4690	6012495	56	45	1.0116974731171	n.s.
4690	6012516	56	45	1.0116974731171	n.s.
4690	6012546	56	45	1.0116974731171	n.s.
4690	6012556	57	44	1.45165612496859	n.s.
4690	6012558	57	44	1.45165612496859	n.s.
4690	6012559	56	45	1.0116974731171	n.s.
4690	6012577	55	46	0.650952919406731	n.s.
4690	6012589	56	45	1.0116974731171	n.s.
4690	6012600	57	44	1.45165612496859	n.s.
4690	6012671	57	44	1.45165612496859	n.s.
4690	6012676	57	44	1.45165612496859	n.s.
4690	6012688	56	45	1.0116974731171	n.s.
4690	6012701	57	44	1.45165612496859	n.s.
4690	6012708	57	44	1.45165612496859	n.s.
4690	6012756	56	45	1.0116974731171	n.s.
4690	6012762	55	46	0.650952919406731	n.s.
4690	6012786	55	46	0.650952919406731	n.s.
4690	6012804	55	46	0.650952919406731	n.s.
4690	6012810	55	46	0.650952919406731	n.s.
4690	6012812	54	47	0.369422463837495	n.s.
4690	6012814	53	48	0.167106106409387	n.s.

4690	6012819	54	47	0.369422463837495	n.s.	
4690	6012831	53	48	0.167106106409387	n.s.	
4690	6012834	52	49	0.044003847122408	n.s.	
4690	6012851	52	49	0.044003847122408	n.s.	
4690	6012936	51	50	0.000115685976557532		n.s.
4690	6012956	50	51	0.0354416229718356	n.s.	
4690	6012992	51	50	0.000115685976557532		n.s.
4690	6012994	51	50	0.000115685976557532		n.s.
4690	6012998	51	50	0.000115685976557532		n.s.
4690	6013007	51	50	0.000115685976557532		n.s.
4690	6013010	50	51	0.0354416229718356	n.s.	
4690	6013026	49	52	0.149981658108242	n.s.	
4690	6013033	48	53	0.343735791385777	n.s.	
4690	6013036	47	54	0.616704022804441	n.s.	
4690	6013044	47	54	0.616704022804441	n.s.	
4690	6013045	48	53	0.343735791385777	n.s.	
4690	6013057	47	54	0.616704022804441	n.s.	
4690	6013081	47	54	0.616704022804441	n.s.	
4690	6013100	47	54	0.616704022804441	n.s.	
4690	6013111	46	55	0.968886352364233	n.s.	
4690	6013112	45	56	1.40028278006515	n.s.	
4690	6013120	44	57	1.9108933059072	n.s.	
4690	6013128	43	58	2.50071792989038	n.s.	
4690	6013132	44	57	1.9108933059072	n.s.	
4690	6013136	43	58	2.50071792989038	n.s.	
4690	6013142	42	59	3.16975665201469	n.s.	
4690	6013143	42	59	3.16975665201469	n.s.	
4690	6013153	42	59	3.16975665201469	n.s.	
4690	6013158	41	60	3.91800947228012	*	
4690	6013163	42	59	3.16975665201469	n.s.	
4690	6013181	42	59	3.16975665201469	n.s.	
4690	6013232	42	59	3.16975665201469	n.s.	
4690	6013238	41	60	3.91800947228012	*	
4690	6013263	41	60	3.91800947228012	*	
4690	6013269	42	59	3.16975665201469	n.s.	
4690	6013277	42	59	3.16975665201469	n.s.	
4690	6013281	41	60	3.91800947228012	*	
4690	6013296	41	60	3.91800947228012	*	
4690	6013301	41	60	3.91800947228012	*	
4690	6013308	40	61	4.74547639068669	*	
4690	6013322	41	60	3.91800947228012	*	
4690	6013338	41	60	3.91800947228012	*	
4690	6013381	41	60	3.91800947228012	*	
4690	6013386	42	59	3.16975665201469	n.s.	
4690	6013408	42	59	3.16975665201469	n.s.	
4690	6013424	41	60	3.91800947228012	*	
4690	6013426	42	59	3.16975665201469	n.s.	
4690	6013433	42	59	3.16975665201469	n.s.	
4690	6013470	42	59	3.16975665201469	n.s.	
4690	6013479	42	59	3.16975665201469	n.s.	

4690	6013510	41	60	3.91800947228012	*
4690	6013515	40	61	4.74547639068669	*
4690	6013523	40	61	4.74547639068669	*
4690	6013534	40	61	4.74547639068669	*
4690	6013546	40	61	4.74547639068669	*
4690	6013555	41	60	3.91800947228012	*
4690	6013591	41	60	3.91800947228012	*
4690	6013592	40	61	4.74547639068669	*
4690	6013594	40	61	4.74547639068669	*
4690	6013619	40	61	4.74547639068669	*
4690	6013623	39	62	5.65215740723438	*
4690	6013635	38	63	6.6380525219232	*
4690	6013638	39	62	5.65215740723438	*
4690	6013667	39	62	5.65215740723438	*
4690	6013674	39	62	5.65215740723438	*
4690	6013678	38	63	6.6380525219232	*
4690	6013710	38	63	6.6380525219232	*
4690	6013813	37	64	7.70316173475315	**
4690	6013824	37	64	7.70316173475315	**
4690	6013845	38	63	6.6380525219232	*
4690	6013869	38	63	6.6380525219232	*
4690	6013874	38	63	6.6380525219232	*
4690	6013891	37	64	7.70316173475315	**
4690	6013917	38	63	6.6380525219232	*
4690	6013926	39	62	5.65215740723438	*
4690	6013930	40	61	4.74547639068669	*
4690	6013931	40	61	4.74547639068669	*
4690	6013991	41	60	3.91800947228012	*
4690	6014016	41	60	3.91800947228012	*
4690	6014025	41	60	3.91800947228012	*
4690	6014026	41	60	3.91800947228012	*
4690	6014047	42	59	3.16975665201469	n.s.
4690	6014055	42	59	3.16975665201469	n.s.
4690	6014077	41	60	3.91800947228012	*
4690	6014101	41	60	3.91800947228012	*
4690	6014116	42	59	3.16975665201469	n.s.
4690	6014135	43	58	2.50071792989038	n.s.
4690	6014142	43	58	2.50071792989038	n.s.
4690	6014155	43	58	2.50071792989038	n.s.
4690	6014162	44	57	1.9108933059072	n.s.
4690	6014164	45	56	1.40028278006515	n.s.
4690	6014165	46	55	0.968886352364233	n.s.
4690	6014166	46	55	0.968886352364233	n.s.
4690	6014167	46	55	0.968886352364233	n.s.
4690	6014197	47	54	0.616704022804441	n.s.
4690	6014199	48	53	0.343735791385777	n.s.
4690	6014215	49	52	0.149981658108242	n.s.
4690	6014216	50	51	0.0354416229718356	n.s.
4690	6014301	51	50	0.000115685976557532	n.s.
4690	6014302	52	49	0.044003847122408	n.s.

4690	6014304	53	48	0.167106106409387	n.s.
4690	6014307	53	48	0.167106106409387	n.s.
4690	6014323	54	47	0.369422463837495	n.s.
4690	6014338	55	46	0.650952919406731	n.s.
4690	6014349	55	46	0.650952919406731	n.s.
4690	6014385	55	46	0.650952919406731	n.s.
4690	6014397	56	45	1.0116974731171	n.s.
4690	6014416	55	46	0.650952919406731	n.s.
4690	6014441	56	45	1.0116974731171	n.s.
4690	6014455	56	45	1.0116974731171	n.s.
4690	6014465	57	44	1.45165612496859	n.s.
4690	6014471	58	43	1.97082887496121	n.s.
4690	6014485	59	42	2.56921572309496	n.s.
4690	6014486	59	42	2.56921572309496	n.s.
4690	6014505	59	42	2.56921572309496	n.s.
4690	6014508	59	42	2.56921572309496	n.s.
4690	6014546	58	43	1.97082887496121	n.s.
4690	6014562	58	43	1.97082887496121	n.s.
4690	6014572	57	44	1.45165612496859	n.s.
4690	6014573	57	44	1.45165612496859	n.s.
4690	6014711	58	43	1.97082887496121	n.s.
4690	6014712	59	42	2.56921572309496	n.s.
4690	6014763	60	41	3.24681666936984	n.s.
4690	6014764	60	41	3.24681666936984	n.s.
4690	6014767	59	42	2.56921572309496	n.s.
4690	6014778	59	42	2.56921572309496	n.s.
4690	6014802	60	41	3.24681666936984	n.s.
4690	6014806	60	41	3.24681666936984	n.s.
4690	6014815	60	41	3.24681666936984	n.s.
4690	6014824	60	41	3.24681666936984	n.s.
4690	6014827	60	41	3.24681666936984	n.s.
4690	6014847	61	40	4.00363171378585	*
4690	6014853	61	40	4.00363171378585	*
4690	6014856	62	39	4.83966085634298	*
4690	6014874	62	39	4.83966085634298	*
4690	6014875	62	39	4.83966085634298	*
4690	6014876	62	39	4.83966085634298	*
4690	6014882	63	38	5.75490409704125	*
4690	6014884	62	39	4.83966085634298	*
4690	6014888	62	39	4.83966085634298	*
4690	6014895	63	38	5.75490409704125	*
4690	6014904	63	38	5.75490409704125	*
4690	6014905	62	39	4.83966085634298	*
4690	6014909	62	39	4.83966085634298	*
4690	6014924	62	39	4.83966085634298	*
4690	6014938	63	38	5.75490409704125	*
4690	6014941	64	37	6.74936143588064	**
4690	6014948	63	38	5.75490409704125	*
4690	6014953	63	38	5.75490409704125	*
4690	6014959	62	39	4.83966085634298	*

4690	6014960	61	40	4.00363171378585	*
4690	6014971	62	39	4.83966085634298	*
4690	6014973	63	38	5.75490409704125	*
4690	6014997	62	39	4.83966085634298	*
4690	6015036	62	39	4.83966085634298	*
4690	6015040	61	40	4.00363171378585	*
4690	6015048	60	41	3.24681666936984	n.s.
4690	6015052	59	42	2.56921572309496	n.s.
4690	6015070	59	42	2.56921572309496	n.s.
4690	6015075	59	42	2.56921572309496	n.s.
4690	6015082	58	43	1.97082887496121	n.s.
4690	6015083	59	42	2.56921572309496	n.s.
4690	6015084	60	41	3.24681666936984	n.s.
4690	6015092	59	42	2.56921572309496	n.s.
4690	6015099	59	42	2.56921572309496	n.s.
4690	6015102	60	41	3.24681666936984	n.s.
4690	6015107	60	41	3.24681666936984	n.s.
4690	6015114	60	41	3.24681666936984	n.s.
4690	6015116	61	40	4.00363171378585	*
4690	6015127	61	40	4.00363171378585	*
4690	6015140	60	41	3.24681666936984	n.s.
4690	6015170	61	40	4.00363171378585	*
4690	6015190	61	40	4.00363171378585	*
4690	6015202	61	40	4.00363171378585	*
4690	6015229	60	41	3.24681666936984	n.s.
4690	6015239	59	42	2.56921572309496	n.s.
4690	6015263	59	42	2.56921572309496	n.s.
4690	6015280	60	41	3.24681666936984	n.s.
4690	6015393	59	42	2.56921572309496	n.s.
4690	6015456	60	41	3.24681666936984	n.s.
4690	6015524	59	42	2.56921572309496	n.s.
4690	6015595	58	43	1.97082887496121	n.s.
4690	6015596	57	44	1.45165612496859	n.s.
4690	6015604	57	44	1.45165612496859	n.s.
4690	6015656	58	43	1.97082887496121	n.s.
4690	6015691	58	43	1.97082887496121	n.s.
4690	6015692	57	44	1.45165612496859	n.s.
4690	6015698	57	44	1.45165612496859	n.s.
4690	6015728	56	45	1.0116974731171	n.s.
4690	6015752	55	46	0.650952919406731	n.s.
4690	6015773	55	46	0.650952919406731	n.s.
4690	6015800	54	47	0.369422463837495	n.s.
4690	6015818	53	48	0.167106106409387	n.s.
4690	6015819	53	48	0.167106106409387	n.s.
4690	6015828	52	49	0.044003847122408	n.s.
4690	6015834	52	49	0.044003847122408	n.s.
4690	6015835	51	50	0.000115685976557532	n.s.
4690	6015840	50	51	0.0354416229718356	n.s.
4690	6015843	49	52	0.149981658108242	n.s.
4690	6015848	48	53	0.343735791385777	n.s.

4690	6015869	47	54	0.616704022804441	n.s.
4690	6015881	47	54	0.616704022804441	n.s.
4690	6015884	47	54	0.616704022804441	n.s.
4690	6015887	47	54	0.616704022804441	n.s.
4690	6015909	46	55	0.968886352364233	n.s.
4690	6015914	47	54	0.616704022804441	n.s.
4690	6015934	47	54	0.616704022804441	n.s.
4690	6015981	47	54	0.616704022804441	n.s.
4690	6015989	47	54	0.616704022804441	n.s.
4690	6015990	46	55	0.968886352364233	n.s.
4690	6015999	46	55	0.968886352364233	n.s.
4690	6016008	46	55	0.968886352364233	n.s.
4690	6016010	46	55	0.968886352364233	n.s.
4690	6016011	46	55	0.968886352364233	n.s.
4690	6016022	47	54	0.616704022804441	n.s.
4690	6016024	47	54	0.616704022804441	n.s.
4690	6016030	46	55	0.968886352364233	n.s.
4690	6016032	46	55	0.968886352364233	n.s.
4690	6016035	47	54	0.616704022804441	n.s.
4690	6016061	48	53	0.343735791385777	n.s.
4690	6016126	48	53	0.343735791385777	n.s.
4690	6016130	48	53	0.343735791385777	n.s.
4690	6016137	48	53	0.343735791385777	n.s.
4690	6016173	47	54	0.616704022804441	n.s.
4690	6016181	48	53	0.343735791385777	n.s.
4690	6016196	49	52	0.149981658108242	n.s.
4690	6016199	50	51	0.0354416229718356	n.s.
4690	6016200	50	51	0.0354416229718356	n.s.
4690	6016206	50	51	0.0354416229718356	n.s.
4690	6016216	49	52	0.149981658108242	n.s.
4690	6016228	49	52	0.149981658108242	n.s.
4690	6016242	49	52	0.149981658108242	n.s.
4690	6016259	49	52	0.149981658108242	n.s.
4690	6016274	49	52	0.149981658108242	n.s.
4690	6016282	49	52	0.149981658108242	n.s.
4690	6016285	49	52	0.149981658108242	n.s.
4690	6016297	49	52	0.149981658108242	n.s.
4690	6016314	50	51	0.0354416229718356	n.s.
4690	6016326	50	51	0.0354416229718356	n.s.
4690	6016364	51	50	0.000115685976557532	n.s.
4690	6016368	51	50	0.000115685976557532	n.s.
4690	6016370	51	50	0.000115685976557532	n.s.
4690	6016376	50	51	0.0354416229718356	n.s.
4690	6016378	50	51	0.0354416229718356	n.s.
4690	6016382	49	52	0.149981658108242	n.s.
4690	6016431	48	53	0.343735791385777	n.s.
4690	6016437	48	53	0.343735791385777	n.s.
4690	6016445	47	54	0.616704022804441	n.s.
4690	6016452	48	53	0.343735791385777	n.s.
4690	6016476	49	52	0.149981658108242	n.s.

4690	6016502	50	51	0.0354416229718356	n.s.
4690	6016503	51	50	0.000115685976557532	n.s.
4690	6016510	51	50	0.000115685976557532	n.s.
4690	6016535	51	50	0.000115685976557532	n.s.
4690	6016536	52	49	0.044003847122408	n.s.
4690	6016537	52	49	0.044003847122408	n.s.
4690	6016539	52	49	0.044003847122408	n.s.
4690	6016574	52	49	0.044003847122408	n.s.
4690	6016623	53	48	0.167106106409387	n.s.
4690	6016692	54	47	0.369422463837495	n.s.
4690	6016706	55	46	0.650952919406731	n.s.
4690	6016709	56	45	1.0116974731171	n.s.
4690	6016734	56	45	1.0116974731171	n.s.
4690	6016779	56	45	1.0116974731171	n.s.
4690	6016785	57	44	1.45165612496859	n.s.
4690	6016801	57	44	1.45165612496859	n.s.
4690	6016804	56	45	1.0116974731171	n.s.
4690	6016806	56	45	1.0116974731171	n.s.
4690	6016817	56	45	1.0116974731171	n.s.
4690	6016828	56	45	1.0116974731171	n.s.
4690	6016834	57	44	1.45165612496859	n.s.
4690	6016849	58	43	1.97082887496121	n.s.
4690	6016851	57	44	1.45165612496859	n.s.
4690	6016866	57	44	1.45165612496859	n.s.
4690	6016872	58	43	1.97082887496121	n.s.
4690	6016882	59	42	2.56921572309496	n.s.
4690	6016924	60	41	3.24681666936984	n.s.
4690	6016944	59	42	2.56921572309496	n.s.
4690	6016951	58	43	1.97082887496121	n.s.
4690	6016958	58	43	1.97082887496121	n.s.
4690	6016963	57	44	1.45165612496859	n.s.
4690	6016969	57	44	1.45165612496859	n.s.
4690	6016974	58	43	1.97082887496121	n.s.
4690	6016983	59	42	2.56921572309496	n.s.
4690	6016984	60	41	3.24681666936984	n.s.
4690	6017043	60	41	3.24681666936984	n.s.
4690	6017047	60	41	3.24681666936984	n.s.
4690	6017054	61	40	4.00363171378585	*
4690	6017055	61	40	4.00363171378585	*
4690	6017057	61	40	4.00363171378585	*
4690	6017151	61	40	4.00363171378585	*
4690	6017152	61	40	4.00363171378585	*
4690	6017163	62	39	4.83966085634298	*
4690	6017177	63	38	5.75490409704125	*
4690	6017179	63	38	5.75490409704125	*
4690	6017257	63	38	5.75490409704125	*
4690	6017302	63	38	5.75490409704125	*
4690	6017321	64	37	6.74936143588064	**
4690	6017353	65	36	7.82303287286116	**
4690	6017357	66	35	8.97591840798281	**

4690	6017362	67	34	10.2080180412456	**	
4690	6017366	67	34	10.2080180412456	**	
4690	6017375	66	35	8.97591840798281	**	
4690	6017379	65	36	7.82303287286116	**	
4690	6017388	64	37	6.74936143588064	**	
4690	6017427	64	37	6.74936143588064	**	
4690	6017432	63	38	5.75490409704125	*	
4690	6017448	63	38	5.75490409704125	*	
4690	6017519	63	38	5.75490409704125	*	
4690	6017520	63	38	5.75490409704125	*	
4690	6017521	63	38	5.75490409704125	*	
4690	6017522	62	39	4.83966085634298	*	
4690	6017523	62	39	4.83966085634298	*	
4690	6017529	61	40	4.00363171378585	*	
4690	6017541	60	41	3.24681666936984	n.s.	
4690	6017551	60	41	3.24681666936984	n.s.	
4690	6017560	61	40	4.00363171378585	*	
4690	6017568	61	40	4.00363171378585	*	
4690	6017596	61	40	4.00363171378585	*	
4690	6017599	60	41	3.24681666936984	n.s.	
4690	6017600	59	42	2.56921572309496	n.s.	
4690	6017601	59	42	2.56921572309496	n.s.	
4690	6017604	58	43	1.97082887496121	n.s.	
4690	6017610	57	44	1.45165612496859	n.s.	
4690	6017625	57	44	1.45165612496859	n.s.	
4690	6017641	56	45	1.0116974731171	n.s.	
4690	6017646	55	46	0.650952919406731	n.s.	
4690	6017659	55	46	0.650952919406731	n.s.	
4690	6017662	54	47	0.369422463837495	n.s.	
4690	6017663	53	48	0.167106106409387	n.s.	
4690	6017691	53	48	0.167106106409387	n.s.	
4690	6017704	53	48	0.167106106409387	n.s.	
4690	6017708	53	48	0.167106106409387	n.s.	
4690	6017725	53	48	0.167106106409387	n.s.	
4690	6017751	53	48	0.167106106409387	n.s.	
4690	6017752	52	49	0.044003847122408	n.s.	
4690	6017757	52	49	0.044003847122408	n.s.	
4690	6017811	51	50	0.000115685976557532		n.s.
4690	6017812	50	51	0.0354416229718356	n.s.	
4690	6019157	49	52	0.149981658108242	n.s.	
4690	6019250	48	53	0.343735791385777	n.s.	
4690	6020411	49	52	0.149981658108242	n.s.	
4690	6020414	48	53	0.343735791385777	n.s.	
4690	6020439	49	52	0.149981658108242	n.s.	
4690	6020452	50	51	0.0354416229718356	n.s.	
4690	6020474	51	50	0.000115685976557532		n.s.
4690	6020477	52	49	0.044003847122408	n.s.	
4690	6020500	51	50	0.000115685976557532		n.s.
4690	6020505	52	49	0.044003847122408	n.s.	
4690	6020515	51	50	0.000115685976557532		n.s.

4690	6021347	50	51	0.0354416229718356	n.s.
4690	6021350	50	51	0.0354416229718356	n.s.
4690	6021358	50	51	0.0354416229718356	n.s.
4690	6021364	50	51	0.0354416229718356	n.s.
4690	6021365	49	52	0.149981658108242	n.s.
4690	6021370	48	53	0.343735791385777	n.s.
4690	6021392	48	53	0.343735791385777	n.s.
4690	6021403	48	53	0.343735791385777	n.s.
4690	6021406	48	53	0.343735791385777	n.s.
4690	6021413	47	54	0.616704022804441	n.s.
4690	6021446	47	54	0.616704022804441	n.s.
4690	6021475	46	55	0.968886352364233	n.s.
4690	6021485	45	56	1.40028278006515	n.s.
4690	6021486	44	57	1.9108933059072	n.s.
4690	6021520	43	58	2.50071792989038	n.s.
4690	6021570	42	59	3.16975665201469	n.s.
4690	6021574	41	60	3.91800947228012	*
4690	6021581	41	60	3.91800947228012	*
4690	6021592	42	59	3.16975665201469	n.s.
4690	6021633	42	59	3.16975665201469	n.s.
4690	6021638	42	59	3.16975665201469	n.s.
4690	6021654	41	60	3.91800947228012	*
4690	6021665	40	61	4.74547639068669	*
4690	6021667	40	61	4.74547639068669	*
4690	6021680	41	60	3.91800947228012	*
4690	6021694	40	61	4.74547639068669	*
4690	6021719	39	62	5.65215740723438	*
4690	6021729	38	63	6.6380525219232	*
4690	6021731	38	63	6.6380525219232	*
4690	6021746	38	63	6.6380525219232	*
4690	6021771	38	63	6.6380525219232	*
4690	6021793	38	63	6.6380525219232	*
4690	6021852	39	62	5.65215740723438	*
4690	6021928	38	63	6.6380525219232	*
4690	6022101	38	63	6.6380525219232	*
4690	6022106	38	63	6.6380525219232	*
4690	6022114	38	63	6.6380525219232	*
4690	6022159	37	64	7.70316173475315	**
4690	6022173	36	65	8.84748504572423	**
4690	6022199	35	66	10.0710224548364	**
4690	6022204	35	66	10.0710224548364	**
4690	6022212	35	66	10.0710224548364	**
4690	6022218	35	66	10.0710224548364	**
4690	6022219	34	67	11.3737739620898	***
4690	6022225	33	68	12.7557395674842	***
4690	6022238	32	69	14.2169192710198	***
4690	6022240	32	69	14.2169192710198	***
4690	6022244	32	69	14.2169192710198	***
4690	6022262	32	69	14.2169192710198	***
4690	6022265	32	69	14.2169192710198	***

4690	6022273	32	69	14.2169192710198	***
4690	6022283	32	69	14.2169192710198	***
4690	6022284	33	68	12.7557395674842	***
4690	6022302	33	68	12.7557395674842	***
4690	6022315	33	68	12.7557395674842	***
4690	6022320	33	68	12.7557395674842	***
4690	6022329	33	68	12.7557395674842	***
4690	6022337	33	68	12.7557395674842	***
4690	6022386	33	68	12.7557395674842	***
4690	6022396	33	68	12.7557395674842	***
4690	6022422	33	68	12.7557395674842	***
4690	6022427	33	68	12.7557395674842	***
4690	6022430	33	68	12.7557395674842	***
4690	6022434	32	69	14.2169192710198	***
4690	6022438	32	69	14.2169192710198	***
4690	6022444	33	68	12.7557395674842	***
4690	6022449	32	69	14.2169192710198	***
4690	6022450	31	70	15.7573130726965	***
4690	6022553	32	69	14.2169192710198	***
4690	6022665	32	69	14.2169192710198	***
4690	6022666	32	69	14.2169192710198	***
4690	6022675	33	68	12.7557395674842	***
4690	6022678	34	67	11.3737739620898	***
4690	6022681	34	67	11.3737739620898	***
4690	6022683	35	66	10.0710224548364	**
4690	6022690	36	65	8.84748504572423	**
4690	6022695	36	65	8.84748504572423	**
4690	6022696	36	65	8.84748504572423	**
4690	6022705	35	66	10.0710224548364	**
4690	6022758	35	66	10.0710224548364	**
4690	6022767	35	66	10.0710224548364	**
4690	6022769	35	66	10.0710224548364	**
4690	6022784	35	66	10.0710224548364	**
4690	6022817	35	66	10.0710224548364	**
4690	6022822	35	66	10.0710224548364	**
4690	6022843	36	65	8.84748504572423	**
4690	6022846	37	64	7.70316173475315	**
4690	6022849	37	64	7.70316173475315	**
4690	6022864	38	63	6.6380525219232	*
4690	6022879	39	62	5.65215740723438	*
4690	6022883	40	61	4.74547639068669	*
4690	6022933	41	60	3.91800947228012	*
4690	6022951	41	60	3.91800947228012	*
4690	6022968	42	59	3.16975665201469	n.s.
4690	6022985	42	59	3.16975665201469	n.s.
4690	6023005	42	59	3.16975665201469	n.s.
4690	6023054	42	59	3.16975665201469	n.s.
4690	6023060	42	59	3.16975665201469	n.s.
4690	6023061	43	58	2.50071792989038	n.s.
4690	6023063	43	58	2.50071792989038	n.s.

4690	6023066	44	57	1.9108933059072	n.s.	
4690	6023067	45	56	1.40028278006515	n.s.	
4690	6023069	45	56	1.40028278006515	n.s.	
4690	6023077	45	56	1.40028278006515	n.s.	
4690	6023078	45	56	1.40028278006515	n.s.	
4690	6023087	46	55	0.968886352364233	n.s.	
4690	6023088	47	54	0.616704022804441	n.s.	
4690	6023096	48	53	0.343735791385777	n.s.	
4690	6023101	49	52	0.149981658108242	n.s.	
4690	6023106	50	51	0.0354416229718356	n.s.	
4690	6023108	51	50	0.000115685976557532		n.s.
4690	6023131	51	50	0.000115685976557532		n.s.
4690	6023160	52	49	0.044003847122408	n.s.	
4690	6023172	53	48	0.167106106409387	n.s.	
4690	6023201	54	47	0.369422463837495	n.s.	
4690	6023202	55	46	0.650952919406731	n.s.	
4690	6023207	56	45	1.0116974731171	n.s.	
4690	6023214	57	44	1.45165612496859	n.s.	
4690	6023268	58	43	1.97082887496121	n.s.	
4690	6023271	58	43	1.97082887496121	n.s.	
4690	6023273	58	43	1.97082887496121	n.s.	
4690	6023275	58	43	1.97082887496121	n.s.	
4690	6023276	59	42	2.56921572309496	n.s.	
4690	6023309	60	41	3.24681666936984	n.s.	
4690	6023310	61	40	4.00363171378585	*	
4690	6023317	61	40	4.00363171378585	*	
4690	6023331	61	40	4.00363171378585	*	
4690	6023332	62	39	4.83966085634298	*	
4690	6023343	63	38	5.75490409704125	*	
4690	6023344	63	38	5.75490409704125	*	
4690	6023346	64	37	6.74936143588064	**	
4690	6023355	64	37	6.74936143588064	**	
4690	6023413	64	37	6.74936143588064	**	
4690	6023428	64	37	6.74936143588064	**	
4690	6023434	64	37	6.74936143588064	**	
4690	6023439	63	38	5.75490409704125	*	
4690	6023451	63	38	5.75490409704125	*	
4690	6023458	62	39	4.83966085634298	*	
4690	6023460	63	38	5.75490409704125	*	
4690	6023469	63	38	5.75490409704125	*	
4690	6023473	64	37	6.74936143588064	**	
4690	6023475	64	37	6.74936143588064	**	
4690	6023478	64	37	6.74936143588064	**	
4690	6023482	64	37	6.74936143588064	**	
4690	6023487	64	37	6.74936143588064	**	
4690	6023491	65	36	7.82303287286116	**	
4690	6023496	66	35	8.97591840798281	**	
4690	6023509	67	34	10.2080180412456	**	
4690	6023514	66	35	8.97591840798281	**	
4690	6023518	65	36	7.82303287286116	**	

4690	6023520	65	36	7.82303287286116	**
4690	6023523	64	37	6.74936143588064	**
4690	6023525	63	38	5.75490409704125	*
4690	6023527	63	38	5.75490409704125	*
4690	6023532	63	38	5.75490409704125	*
4690	6023536	63	38	5.75490409704125	*
4690	6023538	63	38	5.75490409704125	*
4690	6023541	63	38	5.75490409704125	*
4690	6023543	64	37	6.74936143588064	**
4690	6023545	64	37	6.74936143588064	**
4690	6023547	63	38	5.75490409704125	*
4690	6023548	64	37	6.74936143588064	**
4690	6023550	65	36	7.82303287286116	**
4690	6023554	66	35	8.97591840798281	**
4690	6023555	67	34	10.2080180412456	**
4690	6023559	68	33	11.5193317726495	***
4690	6023563	68	33	11.5193317726495	***
4690	6023565	69	32	12.9098596021945	***
4690	6023568	69	32	12.9098596021945	***
4690	6023572	69	32	12.9098596021945	***
4690	6023574	70	31	14.3796015298807	***
4690	6023575	70	31	14.3796015298807	***
4690	6023577	69	32	12.9098596021945	***
4690	6023587	69	32	12.9098596021945	***
4690	6023588	70	31	14.3796015298807	***
4690	6023594	70	31	14.3796015298807	***
4690	6023599	70	31	14.3796015298807	***
4690	6023609	71	30	15.928557555708	***
4690	6023617	72	29	17.5567276796764	***
4690	6023621	72	29	17.5567276796764	***
4690	6023627	73	28	19.264111901786	***
4690	6023635	74	27	21.0507102220366	***
4690	6023637	75	26	22.9165226404284	***
4690	6023639	75	26	22.9165226404284	***
4690	6023645	74	27	21.0507102220366	***
4690	6023664	73	28	19.264111901786	***
4690	6023669	72	29	17.5567276796764	***
4690	6023695	71	30	15.928557555708	***
4690	6023696	71	30	15.928557555708	***
4690	6023701	70	31	14.3796015298807	***
4690	6023704	69	32	12.9098596021945	***
4690	6023745	69	32	12.9098596021945	***
4690	6023747	68	33	11.5193317726495	***
4690	6023750	68	33	11.5193317726495	***
4690	6023775	68	33	11.5193317726495	***
4690	6023796	68	33	11.5193317726495	***
4690	6023798	68	33	11.5193317726495	***
4690	6023804	68	33	11.5193317726495	***
4690	6023833	68	33	11.5193317726495	***
4690	6023835	67	34	10.2080180412456	**

4690	6023844	66	35	8.97591840798281	**
4690	6023859	66	35	8.97591840798281	**
4690	6023865	65	36	7.82303287286116	**
4690	6023887	65	36	7.82303287286116	**
4690	6023921	64	37	6.74936143588064	**
4690	6023924	63	38	5.75490409704125	*
4690	6023928	63	38	5.75490409704125	*
4690	6023952	62	39	4.83966085634298	*
4690	6023954	61	40	4.00363171378585	*
4690	6023955	60	41	3.24681666936984	n.s.
4690	6023979	60	41	3.24681666936984	n.s.
4690	6023998	59	42	2.56921572309496	n.s.
4690	6024009	59	42	2.56921572309496	n.s.
4690	6024010	59	42	2.56921572309496	n.s.
4690	6024019	59	42	2.56921572309496	n.s.
4690	6024020	59	42	2.56921572309496	n.s.
4690	6024029	59	42	2.56921572309496	n.s.
4690	6024032	59	42	2.56921572309496	n.s.
4690	6024036	59	42	2.56921572309496	n.s.
4690	6024048	59	42	2.56921572309496	n.s.
4690	6024050	59	42	2.56921572309496	n.s.
4690	6024053	59	42	2.56921572309496	n.s.
4690	6024056	59	42	2.56921572309496	n.s.
4690	6024062	59	42	2.56921572309496	n.s.
4690	6024070	59	42	2.56921572309496	n.s.
4690	6024075	59	42	2.56921572309496	n.s.
4690	6024085	59	42	2.56921572309496	n.s.
4690	6024089	59	42	2.56921572309496	n.s.
4690	6024095	59	42	2.56921572309496	n.s.
4690	6024102	58	43	1.97082887496121	n.s.
4690	6024116	59	42	2.56921572309496	n.s.
4690	6024120	58	43	1.97082887496121	n.s.
4690	6024121	59	42	2.56921572309496	n.s.
4690	6024132	60	41	3.24681666936984	n.s.
4690	6024136	60	41	3.24681666936984	n.s.
4690	6024145	61	40	4.00363171378585	*
4690	6024178	61	40	4.00363171378585	*
4690	6024191	61	40	4.00363171378585	*
4690	6024194	62	39	4.83966085634298	*
4690	6024195	62	39	4.83966085634298	*
4690	6024196	63	38	5.75490409704125	*
4690	6024207	62	39	4.83966085634298	*
4690	6024213	63	38	5.75490409704125	*
4690	6024223	63	38	5.75490409704125	*
4690	6024224	62	39	4.83966085634298	*
4690	6024234	63	38	5.75490409704125	*
4690	6024250	63	38	5.75490409704125	*
4690	6024257	62	39	4.83966085634298	*
4690	6024287	62	39	4.83966085634298	*
4690	6024294	62	39	4.83966085634298	*

4690	6024295	62	39	4.83966085634298	*
4690	6024298	62	39	4.83966085634298	*
4690	6024334	63	38	5.75490409704125	*
4690	6024339	64	37	6.74936143588064	**
4690	6024367	63	38	5.75490409704125	*
4690	6024373	63	38	5.75490409704125	*
4690	6024407	63	38	5.75490409704125	*
4690	6024419	64	37	6.74936143588064	**
4690	6024436	65	36	7.82303287286116	**
4690	6024442	65	36	7.82303287286116	**
4690	6024455	66	35	8.97591840798281	**
4690	6024457	67	34	10.2080180412456	**
4690	6024480	66	35	8.97591840798281	**
4690	6024495	66	35	8.97591840798281	**
4690	6024509	66	35	8.97591840798281	**
4690	6024522	66	35	8.97591840798281	**
4690	6024537	66	35	8.97591840798281	**
4690	6024539	66	35	8.97591840798281	**
4690	6024555	65	36	7.82303287286116	**
4690	6024563	65	36	7.82303287286116	**
4690	6024571	65	36	7.82303287286116	**
4690	6024575	64	37	6.74936143588064	**
4690	6024581	65	36	7.82303287286116	**
4690	6024584	66	35	8.97591840798281	**
4690	6024588	65	36	7.82303287286116	**
4690	6024617	64	37	6.74936143588064	**
4690	6024646	63	38	5.75490409704125	*
4690	6024670	63	38	5.75490409704125	*
4690	6024672	62	39	4.83966085634298	*
4690	6024695	61	40	4.00363171378585	*
4690	6024734	61	40	4.00363171378585	*
4690	6024739	61	40	4.00363171378585	*
4690	6024763	61	40	4.00363171378585	*
4690	6024768	60	41	3.24681666936984	n.s.
4690	6024769	60	41	3.24681666936984	n.s.
4690	6024773	60	41	3.24681666936984	n.s.
4690	6024793	61	40	4.00363171378585	*
4690	6024795	61	40	4.00363171378585	*
4690	6024796	61	40	4.00363171378585	*
4690	6024836	61	40	4.00363171378585	*
4690	6024838	62	39	4.83966085634298	*
4690	6024844	63	38	5.75490409704125	*
4690	6024852	63	38	5.75490409704125	*
4690	6024892	63	38	5.75490409704125	*
4690	6024895	63	38	5.75490409704125	*
4690	6024925	63	38	5.75490409704125	*
4690	6024930	63	38	5.75490409704125	*
4690	6024949	63	38	5.75490409704125	*
4690	6024979	63	38	5.75490409704125	*
4690	6024982	63	38	5.75490409704125	*

4690	6024990	64	37	6.74936143588064	**
4690	6024994	65	36	7.82303287286116	**
4690	6025004	65	36	7.82303287286116	**
4690	6025008	66	35	8.97591840798281	**
4690	6025011	65	36	7.82303287286116	**
4690	6025015	66	35	8.97591840798281	**
4690	6025018	66	35	8.97591840798281	**
4690	6025023	66	35	8.97591840798281	**
4690	6025025	66	35	8.97591840798281	**
4690	6025027	66	35	8.97591840798281	**
4690	6025031	66	35	8.97591840798281	**
4690	6025033	65	36	7.82303287286116	**
4690	6025036	65	36	7.82303287286116	**
4690	6025051	65	36	7.82303287286116	**
4690	6025056	65	36	7.82303287286116	**
4690	6025067	65	36	7.82303287286116	**
4690	6025079	65	36	7.82303287286116	**
4690	6025090	64	37	6.74936143588064	**
4690	6025096	64	37	6.74936143588064	**
4690	6025104	63	38	5.75490409704125	*
4690	6025123	63	38	5.75490409704125	*
4690	6025159	62	39	4.83966085634298	*
4690	6025176	62	39	4.83966085634298	*
4690	6025179	62	39	4.83966085634298	*
4690	6025184	62	39	4.83966085634298	*
4690	6025192	62	39	4.83966085634298	*
4690	6025222	62	39	4.83966085634298	*
4690	6025227	61	40	4.00363171378585	*
4690	6025228	61	40	4.00363171378585	*
4690	6025261	61	40	4.00363171378585	*
4690	6025268	62	39	4.83966085634298	*
4690	6025280	62	39	4.83966085634298	*
4690	6025293	63	38	5.75490409704125	*
4690	6025310	62	39	4.83966085634298	*
4690	6025312	61	40	4.00363171378585	*
4690	6025336	61	40	4.00363171378585	*
4690	6025338	61	40	4.00363171378585	*
4690	6025354	62	39	4.83966085634298	*
4690	6025360	62	39	4.83966085634298	*
4690	6025362	62	39	4.83966085634298	*
4690	6025363	62	39	4.83966085634298	*
4690	6025372	62	39	4.83966085634298	*
4690	6025378	63	38	5.75490409704125	*
4690	6025380	63	38	5.75490409704125	*
4690	6025396	64	37	6.74936143588064	**
4690	6025411	65	36	7.82303287286116	**
4690	6025432	65	36	7.82303287286116	**
4690	6025433	65	36	7.82303287286116	**
4690	6025438	66	35	8.97591840798281	**
4690	6025462	66	35	8.97591840798281	**

4690	6025465	67	34	10.2080180412456	**
4690	6025468	67	34	10.2080180412456	**
4690	6025473	67	34	10.2080180412456	**
4690	6025496	66	35	8.97591840798281	**
4690	6025504	66	35	8.97591840798281	**
4690	6025514	67	34	10.2080180412456	**
4690	6025519	67	34	10.2080180412456	**
4690	6025528	67	34	10.2080180412456	**
4690	6025552	67	34	10.2080180412456	**
4690	6025554	67	34	10.2080180412456	**
4690	6025624	66	35	8.97591840798281	**
4690	6025648	66	35	8.97591840798281	**
4690	6025657	66	35	8.97591840798281	**
4690	6025660	67	34	10.2080180412456	**
4690	6025666	67	34	10.2080180412456	**
4690	6025675	67	34	10.2080180412456	**
4690	6025692	67	34	10.2080180412456	**
4690	6025720	66	35	8.97591840798281	**
4690	6025732	65	36	7.82303287286116	**
4690	6025736	66	35	8.97591840798281	**
4690	6025737	67	34	10.2080180412456	**
4690	6025741	67	34	10.2080180412456	**
4690	6025792	67	34	10.2080180412456	**
4690	6025801	67	34	10.2080180412456	**
4690	6025810	66	35	8.97591840798281	**
4690	6025820	66	35	8.97591840798281	**
4690	6025821	66	35	8.97591840798281	**
4690	6025822	66	35	8.97591840798281	**
4690	6025834	66	35	8.97591840798281	**
4690	6025837	67	34	10.2080180412456	**
4690	6025840	67	34	10.2080180412456	**
4690	6025841	68	33	11.5193317726495	***
4690	6025846	67	34	10.2080180412456	**
4690	6025868	67	34	10.2080180412456	**
4690	6025879	68	33	11.5193317726495	***
4690	6025896	69	32	12.9098596021945	***
4690	6025898	70	31	14.3796015298807	***
4690	6025908	69	32	12.9098596021945	***
4690	6025915	69	32	12.9098596021945	***
4690	6025924	69	32	12.9098596021945	***
4690	6025930	69	32	12.9098596021945	***
4690	6025940	68	33	11.5193317726495	***
4690	6025948	68	33	11.5193317726495	***
4690	6025951	68	33	11.5193317726495	***
4690	6025963	69	32	12.9098596021945	***
4690	6025968	69	32	12.9098596021945	***
4690	6025972	69	32	12.9098596021945	***
4690	6025973	69	32	12.9098596021945	***
4690	6025975	68	33	11.5193317726495	***
4690	6025982	67	34	10.2080180412456	**

4690	6025990	67	34	10.2080180412456	**
4690	6026002	66	35	8.97591840798281	**
4690	6026048	66	35	8.97591840798281	**
4690	6026068	66	35	8.97591840798281	**
4690	6026081	66	35	8.97591840798281	**
4690	6026085	67	34	10.2080180412456	**
4690	6026089	66	35	8.97591840798281	**
4690	6026092	67	34	10.2080180412456	**
4690	6026099	67	34	10.2080180412456	**
4690	6026105	68	33	11.5193317726495	***
4690	6026129	69	32	12.9098596021945	***
4690	6026141	69	32	12.9098596021945	***
4690	6026153	69	32	12.9098596021945	***
4690	6026174	69	32	12.9098596021945	***
4690	6026192	68	33	11.5193317726495	***
4690	6026226	68	33	11.5193317726495	***
4690	6026229	68	33	11.5193317726495	***
4690	6026233	68	33	11.5193317726495	***
4690	6026243	69	32	12.9098596021945	***
4690	6026271	69	32	12.9098596021945	***
4690	6026274	70	31	14.3796015298807	***
4690	6026284	70	31	14.3796015298807	***
4690	6026296	71	30	15.928557555708	***
4690	6026301	71	30	15.928557555708	***
4690	6026311	70	31	14.3796015298807	***
4690	6026344	70	31	14.3796015298807	***
4690	6026353	70	31	14.3796015298807	***
4690	6026377	70	31	14.3796015298807	***
4690	6026386	71	30	15.928557555708	***
4690	6026411	72	29	17.5567276796764	***
4690	6026413	72	29	17.5567276796764	***
4690	6026420	72	29	17.5567276796764	***
4690	6026428	72	29	17.5567276796764	***
4690	6026429	72	29	17.5567276796764	***
4690	6026452	73	28	19.264111901786	***
4690	6026479	74	27	21.0507102220366	***
4690	6026491	74	27	21.0507102220366	***
4690	6026509	74	27	21.0507102220366	***
4690	6026551	73	28	19.264111901786	***
4690	6026552	73	28	19.264111901786	***
4690	6026569	73	28	19.264111901786	***
4690	6026578	73	28	19.264111901786	***
4690	6026581	73	28	19.264111901786	***
4690	6026584	73	28	19.264111901786	***
4690	6026586	73	28	19.264111901786	***
4690	6026609	73	28	19.264111901786	***
4690	6026613	73	28	19.264111901786	***
4690	6026620	73	28	19.264111901786	***
4690	6026623	73	28	19.264111901786	***
4690	6026630	72	29	17.5567276796764	***

4690	6026631	71	30	15.928557555708	***
4690	6026635	71	30	15.928557555708	***
4690	6026656	72	29	17.5567276796764	***
4690	6026674	72	29	17.5567276796764	***
4690	6026678	73	28	19.264111901786	***
4690	6026679	73	28	19.264111901786	***
4690	6026696	73	28	19.264111901786	***
4690	6026714	73	28	19.264111901786	***
4690	6026741	74	27	21.0507102220366	***
4690	6026758	74	27	21.0507102220366	***
4690	6026759	74	27	21.0507102220366	***
4690	6026767	74	27	21.0507102220366	***
4690	6026770	74	27	21.0507102220366	***
4690	6026809	74	27	21.0507102220366	***
4690	6026826	73	28	19.264111901786	***
4690	6026833	72	29	17.5567276796764	***
4690	6026836	72	29	17.5567276796764	***
4690	6026845	71	30	15.928557555708	***
4690	6026860	71	30	15.928557555708	***
4690	6026890	71	30	15.928557555708	***
4690	6026893	71	30	15.928557555708	***
4690	6026897	71	30	15.928557555708	***
4690	6026901	71	30	15.928557555708	***
4690	6026903	71	30	15.928557555708	***
4690	6026905	70	31	14.3796015298807	***
4690	6026908	70	31	14.3796015298807	***
4690	6026931	70	31	14.3796015298807	***
4690	6026933	70	31	14.3796015298807	***
4690	6026936	71	30	15.928557555708	***
4690	6026944	71	30	15.928557555708	***
4690	6026951	71	30	15.928557555708	***
4690	6026953	72	29	17.5567276796764	***
4690	6026955	72	29	17.5567276796764	***
4690	6026967	73	28	19.264111901786	***
4690	6026968	73	28	19.264111901786	***
4690	6026977	73	28	19.264111901786	***
4690	6026996	72	29	17.5567276796764	***
4690	6027002	72	29	17.5567276796764	***
4690	6027007	73	28	19.264111901786	***
4690	6027019	74	27	21.0507102220366	***
4690	6027038	74	27	21.0507102220366	***
4690	6027046	74	27	21.0507102220366	***
4690	6027061	74	27	21.0507102220366	***
4690	6027077	73	28	19.264111901786	***
4690	6027078	73	28	19.264111901786	***
4690	6027088	72	29	17.5567276796764	***
4690	6027093	71	30	15.928557555708	***
4690	6027096	70	31	14.3796015298807	***
4690	6027097	70	31	14.3796015298807	***
4690	6027102	71	30	15.928557555708	***

4690	6027106	71	30	15.928557555708	***
4690	6027124	70	31	14.3796015298807	***
4690	6027132	70	31	14.3796015298807	***
4690	6027133	70	31	14.3796015298807	***
4690	6027145	70	31	14.3796015298807	***
4690	6027150	69	32	12.9098596021945	***
4690	6027164	68	33	11.5193317726495	***
4690	6027174	68	33	11.5193317726495	***
4690	6027190	67	34	10.2080180412456	**
4690	6027191	66	35	8.97591840798281	**
4690	6027201	66	35	8.97591840798281	**
4690	6027258	66	35	8.97591840798281	**
4690	6027283	67	34	10.2080180412456	**
4690	6027285	67	34	10.2080180412456	**
4690	6027320	68	33	11.5193317726495	***
4690	6027322	68	33	11.5193317726495	***
4690	6027326	68	33	11.5193317726495	***
4690	6027343	68	33	11.5193317726495	***
4690	6027359	68	33	11.5193317726495	***
4690	6027403	68	33	11.5193317726495	***
4690	6027406	67	34	10.2080180412456	**
4690	6027444	66	35	8.97591840798281	**
4690	6027472	65	36	7.82303287286116	**
4690	6027487	65	36	7.82303287286116	**
4690	6027513	64	37	6.74936143588064	**
4690	6027537	65	36	7.82303287286116	**
4690	6027543	65	36	7.82303287286116	**
4690	6027545	65	36	7.82303287286116	**
4690	6027553	64	37	6.74936143588064	**
4690	6027556	64	37	6.74936143588064	**
4690	6027567	63	38	5.75490409704125	*
4690	6027578	63	38	5.75490409704125	*
4690	6027579	62	39	4.83966085634298	*
4690	6027592	61	40	4.00363171378585	*
4690	6027593	60	41	3.24681666936984	n.s.
4690	6027597	60	41	3.24681666936984	n.s.
4690	6027604	60	41	3.24681666936984	n.s.
4690	6027606	60	41	3.24681666936984	n.s.
4690	6027614	59	42	2.56921572309496	n.s.
4690	6027652	59	42	2.56921572309496	n.s.
4690	6027656	58	43	1.97082887496121	n.s.
4690	6027657	57	44	1.45165612496859	n.s.
4690	6027661	56	45	1.0116974731171	n.s.
4690	6027672	55	46	0.650952919406731	n.s.
4690	6027673	55	46	0.650952919406731	n.s.
4690	6027680	54	47	0.369422463837495	n.s.
4690	6027681	53	48	0.167106106409387	n.s.
4690	6027697	52	49	0.044003847122408	n.s.
4690	6027710	51	50	0.000115685976557532	n.s.
4690	6027726	50	51	0.0354416229718356	n.s.

4690	6027729	50	51	0.0354416229718356	n.s.
4690	6027757	50	51	0.0354416229718356	n.s.
4690	6027767	49	52	0.149981658108242	n.s.
4690	6027768	49	52	0.149981658108242	n.s.
4690	6027772	49	52	0.149981658108242	n.s.
4690	6027776	49	52	0.149981658108242	n.s.
4690	6027781	48	53	0.343735791385777	n.s.
4690	6027785	47	54	0.616704022804441	n.s.
4690	6027786	47	54	0.616704022804441	n.s.
4690	6027800	47	54	0.616704022804441	n.s.
4690	6027802	46	55	0.968886352364233	n.s.
4690	6027815	45	56	1.40028278006515	n.s.
4690	6027817	45	56	1.40028278006515	n.s.
4690	6027831	44	57	1.9108933059072	n.s.
4690	6027834	44	57	1.9108933059072	n.s.
4690	6027840	45	56	1.40028278006515	n.s.
4690	6027845	45	56	1.40028278006515	n.s.
4690	6027846	45	56	1.40028278006515	n.s.
4690	6027850	46	55	0.968886352364233	n.s.
4690	6027852	47	54	0.616704022804441	n.s.
4690	6027866	48	53	0.343735791385777	n.s.
4690	6027872	47	54	0.616704022804441	n.s.
4690	6027874	46	55	0.968886352364233	n.s.
4690	6027888	46	55	0.968886352364233	n.s.
4690	6027891	46	55	0.968886352364233	n.s.
4690	6027893	46	55	0.968886352364233	n.s.
4690	6027900	46	55	0.968886352364233	n.s.
4690	6027919	46	55	0.968886352364233	n.s.
4690	6027923	46	55	0.968886352364233	n.s.
4690	6027936	46	55	0.968886352364233	n.s.
4690	6027945	45	56	1.40028278006515	n.s.
4690	6027962	45	56	1.40028278006515	n.s.
4690	6027963	45	56	1.40028278006515	n.s.
4690	6027981	44	57	1.9108933059072	n.s.
4690	6027991	44	57	1.9108933059072	n.s.
4690	6028009	44	57	1.9108933059072	n.s.
4690	6028012	43	58	2.50071792989038	n.s.
4690	6028032	44	57	1.9108933059072	n.s.
4690	6028050	43	58	2.50071792989038	n.s.
4690	6028054	42	59	3.16975665201469	n.s.
4690	6028064	41	60	3.91800947228012	*
4690	6028080	42	59	3.16975665201469	n.s.
4690	6028084	42	59	3.16975665201469	n.s.
4690	6028101	43	58	2.50071792989038	n.s.
4690	6028103	43	58	2.50071792989038	n.s.
4690	6028109	43	58	2.50071792989038	n.s.
4690	6028111	44	57	1.9108933059072	n.s.
4690	6028118	44	57	1.9108933059072	n.s.
4690	6028131	44	57	1.9108933059072	n.s.
4690	6028184	43	58	2.50071792989038	n.s.

4690	6028211	43	58	2.50071792989038	n.s.
4690	6028216	43	58	2.50071792989038	n.s.
4690	6028222	43	58	2.50071792989038	n.s.
4690	6028241	43	58	2.50071792989038	n.s.
4690	6028251	43	58	2.50071792989038	n.s.
4690	6028257	42	59	3.16975665201469	n.s.
4690	6028280	42	59	3.16975665201469	n.s.
4690	6028321	42	59	3.16975665201469	n.s.
4690	6028323	43	58	2.50071792989038	n.s.
4690	6028325	43	58	2.50071792989038	n.s.
4690	6028344	44	57	1.9108933059072	n.s.
4690	6028348	44	57	1.9108933059072	n.s.
4690	6028350	44	57	1.9108933059072	n.s.
4690	6028374	43	58	2.50071792989038	n.s.
4690	6028380	43	58	2.50071792989038	n.s.
4690	6028389	42	59	3.16975665201469	n.s.
4690	6028394	43	58	2.50071792989038	n.s.
4690	6028401	43	58	2.50071792989038	n.s.
4690	6028402	42	59	3.16975665201469	n.s.
4690	6028404	41	60	3.91800947228012	*
4690	6028409	40	61	4.74547639068669	*
4690	6028419	41	60	3.91800947228012	*
4690	6028425	42	59	3.16975665201469	n.s.
4690	6028430	42	59	3.16975665201469	n.s.
4690	6028446	42	59	3.16975665201469	n.s.
4690	6028458	42	59	3.16975665201469	n.s.
4690	6028464	42	59	3.16975665201469	n.s.
4690	6028480	41	60	3.91800947228012	*
4690	6028492	41	60	3.91800947228012	*
4690	6028493	42	59	3.16975665201469	n.s.
4690	6028501	42	59	3.16975665201469	n.s.
4690	6028510	42	59	3.16975665201469	n.s.
4690	6028626	41	60	3.91800947228012	*
4690	6028650	42	59	3.16975665201469	n.s.
4690	6028659	42	59	3.16975665201469	n.s.
4690	6028668	42	59	3.16975665201469	n.s.
4690	6028675	42	59	3.16975665201469	n.s.
4690	6028699	41	60	3.91800947228012	*
4690	6028709	40	61	4.74547639068669	*
4690	6028744	40	61	4.74547639068669	*
4690	6028746	41	60	3.91800947228012	*
4690	6028765	41	60	3.91800947228012	*
4690	6028783	41	60	3.91800947228012	*
4690	6028793	41	60	3.91800947228012	*
4690	6028802	42	59	3.16975665201469	n.s.
4690	6028833	41	60	3.91800947228012	*
4690	6028842	42	59	3.16975665201469	n.s.
4690	6028853	43	58	2.50071792989038	n.s.
4690	6028855	43	58	2.50071792989038	n.s.
4690	6028856	44	57	1.9108933059072	n.s.

4690	6028875	45	56	1.40028278006515	n.s.
4690	6028881	46	55	0.968886352364233	n.s.
4690	6028906	46	55	0.968886352364233	n.s.
4690	6028911	47	54	0.616704022804441	n.s.
4690	6028912	47	54	0.616704022804441	n.s.
4690	6028917	46	55	0.968886352364233	n.s.
4690	6028920	46	55	0.968886352364233	n.s.
4690	6028945	46	55	0.968886352364233	n.s.
4690	6028948	47	54	0.616704022804441	n.s.
4690	6028950	48	53	0.343735791385777	n.s.
4690	6028953	47	54	0.616704022804441	n.s.
4690	6028954	47	54	0.616704022804441	n.s.
4690	6028959	47	54	0.616704022804441	n.s.
4690	6029003	48	53	0.343735791385777	n.s.
4690	6029016	49	52	0.149981658108242	n.s.
4690	6029025	48	53	0.343735791385777	n.s.
4690	6029028	47	54	0.616704022804441	n.s.
4690	6029029	48	53	0.343735791385777	n.s.
4690	6029030	47	54	0.616704022804441	n.s.
4690	6029039	47	54	0.616704022804441	n.s.
4690	6029051	47	54	0.616704022804441	n.s.
4690	6029053	47	54	0.616704022804441	n.s.
4690	6029065	47	54	0.616704022804441	n.s.
4690	6029089	47	54	0.616704022804441	n.s.
4690	6029090	47	54	0.616704022804441	n.s.
4690	6029103	48	53	0.343735791385777	n.s.
4690	6029114	49	52	0.149981658108242	n.s.
4690	6029117	50	51	0.0354416229718356	n.s.
4690	6029119	50	51	0.0354416229718356	n.s.
4690	6029133	50	51	0.0354416229718356	n.s.
4690	6029141	50	51	0.0354416229718356	n.s.
4690	6029142	51	50	0.000115685976557532	n.s.
4690	6029152	50	51	0.0354416229718356	n.s.
4690	6029154	49	52	0.149981658108242	n.s.
4690	6029159	49	52	0.149981658108242	n.s.
4690	6029164	49	52	0.149981658108242	n.s.
4690	6029170	48	53	0.343735791385777	n.s.
4690	6029172	48	53	0.343735791385777	n.s.
4690	6029173	48	53	0.343735791385777	n.s.
4690	6029174	49	52	0.149981658108242	n.s.
4690	6029180	50	51	0.0354416229718356	n.s.
4690	6029184	51	50	0.000115685976557532	n.s.
4690	6029186	51	50	0.000115685976557532	n.s.
4690	6029198	50	51	0.0354416229718356	n.s.
4690	6029207	50	51	0.0354416229718356	n.s.
4690	6029225	51	50	0.000115685976557532	n.s.
4690	6029227	51	50	0.000115685976557532	n.s.
4690	6029237	51	50	0.000115685976557532	n.s.
4690	6029239	52	49	0.044003847122408	n.s.
4690	6029276	53	48	0.167106106409387	n.s.

4690	6029307	54	47	0.369422463837495	n.s.
4690	6029314	53	48	0.167106106409387	n.s.
4690	6029317	54	47	0.369422463837495	n.s.
4690	6029333	55	46	0.650952919406731	n.s.
4690	6029354	56	45	1.0116974731171	n.s.
4690	6029392	56	45	1.0116974731171	n.s.
4690	6029397	57	44	1.45165612496859	n.s.
4690	6029400	58	43	1.97082887496121	n.s.
4690	6029427	59	42	2.56921572309496	n.s.
4690	6029434	59	42	2.56921572309496	n.s.
4690	6029435	60	41	3.24681666936984	n.s.
4690	6029437	60	41	3.24681666936984	n.s.
4690	6029458	60	41	3.24681666936984	n.s.
4690	6029479	60	41	3.24681666936984	n.s.
4690	6029535	61	40	4.00363171378585	*
4690	6029543	62	39	4.83966085634298	*
4690	6029557	63	38	5.75490409704125	*
4690	6029558	63	38	5.75490409704125	*
4690	6029559	63	38	5.75490409704125	*
4690	6029565	64	37	6.74936143588064	**
4690	6029570	65	36	7.82303287286116	**
4690	6029572	66	35	8.97591840798281	**
4690	6029575	66	35	8.97591840798281	**
4690	6029583	66	35	8.97591840798281	**
4690	6029597	67	34	10.2080180412456	**
4690	6029598	66	35	8.97591840798281	**
4690	6029639	66	35	8.97591840798281	**
4690	6029666	65	36	7.82303287286116	**
4690	6029688	65	36	7.82303287286116	**
4690	6029691	65	36	7.82303287286116	**
4690	6029699	64	37	6.74936143588064	**
4690	6029706	63	38	5.75490409704125	*
4690	6029708	63	38	5.75490409704125	*
4690	6029714	63	38	5.75490409704125	*
4690	6029770	63	38	5.75490409704125	*
4690	6029802	64	37	6.74936143588064	**
4690	6029809	64	37	6.74936143588064	**
4690	6029812	63	38	5.75490409704125	*
4690	6029827	63	38	5.75490409704125	*
4690	6029845	64	37	6.74936143588064	**
4690	6029853	65	36	7.82303287286116	**
4690	6029880	65	36	7.82303287286116	**
4690	6029887	66	35	8.97591840798281	**
4690	6029890	66	35	8.97591840798281	**
4690	6029896	67	34	10.2080180412456	**
4690	6029920	66	35	8.97591840798281	**
4690	6029921	67	34	10.2080180412456	**
4690	6029929	67	34	10.2080180412456	**
4690	6029941	66	35	8.97591840798281	**
4690	6029944	66	35	8.97591840798281	**

4690	6029953	66	35	8.97591840798281	**
4690	6029962	66	35	8.97591840798281	**
4690	6029967	66	35	8.97591840798281	**
4690	6029968	66	35	8.97591840798281	**
4690	6029971	66	35	8.97591840798281	**
4690	6029981	65	36	7.82303287286116	**
4690	6029986	66	35	8.97591840798281	**
4690	6029989	65	36	7.82303287286116	**
4690	6029998	65	36	7.82303287286116	**
4690	6029999	64	37	6.74936143588064	**
4690	6030004	64	37	6.74936143588064	**
4690	6030010	64	37	6.74936143588064	**
4690	6030013	64	37	6.74936143588064	**
4690	6030016	65	36	7.82303287286116	**
4690	6030028	64	37	6.74936143588064	**
4690	6030029	63	38	5.75490409704125	*
4690	6030034	63	38	5.75490409704125	*
4690	6030039	64	37	6.74936143588064	**
4690	6030040	64	37	6.74936143588064	**
4690	6030043	64	37	6.74936143588064	**
4690	6030045	63	38	5.75490409704125	*
4690	6030061	62	39	4.83966085634298	*
4690	6030072	61	40	4.00363171378585	*
4690	6030076	62	39	4.83966085634298	*
4690	6030082	63	38	5.75490409704125	*
4690	6030101	62	39	4.83966085634298	*
4690	6030106	61	40	4.00363171378585	*
4690	6030114	61	40	4.00363171378585	*
4690	6030115	61	40	4.00363171378585	*
4690	6030134	61	40	4.00363171378585	*
4690	6030142	61	40	4.00363171378585	*
4690	6030148	60	41	3.24681666936984	n.s.
4690	6030178	59	42	2.56921572309496	n.s.
4690	6030192	59	42	2.56921572309496	n.s.
4690	6030197	60	41	3.24681666936984	n.s.
4690	6030204	61	40	4.00363171378585	*
4690	6030218	61	40	4.00363171378585	*
4690	6030239	62	39	4.83966085634298	*
4690	6030245	63	38	5.75490409704125	*
4690	6030248	62	39	4.83966085634298	*
4690	6030250	61	40	4.00363171378585	*
4690	6030252	61	40	4.00363171378585	*
4690	6030253	60	41	3.24681666936984	n.s.
4690	6030273	59	42	2.56921572309496	n.s.
4690	6030274	59	42	2.56921572309496	n.s.
4690	6030277	58	43	1.97082887496121	n.s.
4690	6030283	58	43	1.97082887496121	n.s.
4690	6030284	58	43	1.97082887496121	n.s.
4690	6030286	58	43	1.97082887496121	n.s.
4690	6030299	57	44	1.45165612496859	n.s.

4690	6030305	57	44	1.45165612496859	n.s.
4690	6030327	56	45	1.0116974731171	n.s.
4690	6030330	57	44	1.45165612496859	n.s.
4690	6030335	56	45	1.0116974731171	n.s.
4690	6030340	55	46	0.650952919406731	n.s.
4690	6030346	54	47	0.369422463837495	n.s.
4690	6030365	53	48	0.167106106409387	n.s.
4690	6030380	52	49	0.044003847122408	n.s.
4690	6030381	52	49	0.044003847122408	n.s.
4690	6030393	51	50	0.000115685976557532	n.s.
4690	6030399	50	51	0.0354416229718356	n.s.
4690	6030416	50	51	0.0354416229718356	n.s.
4690	6030443	49	52	0.149981658108242	n.s.
4690	6030479	48	53	0.343735791385777	n.s.
4690	6030492	47	54	0.616704022804441	n.s.
4690	6030515	46	55	0.968886352364233	n.s.
4690	6030533	46	55	0.968886352364233	n.s.
4690	6030553	45	56	1.40028278006515	n.s.
4690	6030556	44	57	1.9108933059072	n.s.
4690	6030557	44	57	1.9108933059072	n.s.
4690	6030576	43	58	2.50071792989038	n.s.
4690	6030577	42	59	3.16975665201469	n.s.
4690	6030587	42	59	3.16975665201469	n.s.
4690	6030614	41	60	3.91800947228012	*
4690	6030618	41	60	3.91800947228012	*
4690	6030633	40	61	4.74547639068669	*
4690	6030643	40	61	4.74547639068669	*
4690	6030644	41	60	3.91800947228012	*
4690	6030654	41	60	3.91800947228012	*
4690	6030655	41	60	3.91800947228012	*
4690	6030661	40	61	4.74547639068669	*
4690	6030665	41	60	3.91800947228012	*
4690	6030671	42	59	3.16975665201469	n.s.
4690	6030674	42	59	3.16975665201469	n.s.
4690	6030686	43	58	2.50071792989038	n.s.
4690	6030693	43	58	2.50071792989038	n.s.
4690	6030699	42	59	3.16975665201469	n.s.
4690	6030701	42	59	3.16975665201469	n.s.
4690	6030705	42	59	3.16975665201469	n.s.
4690	6030710	43	58	2.50071792989038	n.s.
4690	6030717	43	58	2.50071792989038	n.s.
4690	6030720	43	58	2.50071792989038	n.s.
4690	6030748	43	58	2.50071792989038	n.s.
4690	6030749	43	58	2.50071792989038	n.s.
4690	6030775	44	57	1.9108933059072	n.s.
4690	6030793	44	57	1.9108933059072	n.s.
4690	6030796	44	57	1.9108933059072	n.s.
4690	6030797	43	58	2.50071792989038	n.s.
4690	6030798	42	59	3.16975665201469	n.s.
4690	6030804	43	58	2.50071792989038	n.s.

4690	6030813	43	58	2.50071792989038	n.s.
4690	6030815	43	58	2.50071792989038	n.s.
4690	6030840	42	59	3.16975665201469	n.s.
4690	6030843	41	60	3.91800947228012	*
4690	6030858	41	60	3.91800947228012	*
4690	6030864	40	61	4.74547639068669	*
4690	6030873	40	61	4.74547639068669	*
4690	6030876	39	62	5.65215740723438	*
4690	6030924	39	62	5.65215740723438	*
4690	6030928	40	61	4.74547639068669	*
4690	6030936	40	61	4.74547639068669	*
4690	6030971	39	62	5.65215740723438	*
4690	6030996	39	62	5.65215740723438	*
4690	6031000	39	62	5.65215740723438	*
4690	6031003	39	62	5.65215740723438	*
4690	6031010	40	61	4.74547639068669	*
4690	6031023	41	60	3.91800947228012	*
4690	6031030	41	60	3.91800947228012	*
4690	6031034	40	61	4.74547639068669	*
4690	6031036	39	62	5.65215740723438	*
4690	6031069	39	62	5.65215740723438	*
4690	6031074	39	62	5.65215740723438	*
4690	6031087	39	62	5.65215740723438	*
4690	6031101	39	62	5.65215740723438	*
4690	6031114	38	63	6.6380525219232	*
4690	6031155	37	64	7.70316173475315	**
4690	6031165	37	64	7.70316173475315	**
4690	6031177	37	64	7.70316173475315	**
4690	6031221	36	65	8.84748504572423	**
4690	6031223	35	66	10.0710224548364	**
4690	6031241	36	65	8.84748504572423	**
4690	6031267	36	65	8.84748504572423	**
4690	6031288	36	65	8.84748504572423	**
4690	6031311	37	64	7.70316173475315	**
4690	6031314	38	63	6.6380525219232	*
4690	6031329	38	63	6.6380525219232	*
4690	6031335	38	63	6.6380525219232	*
4690	6031341	38	63	6.6380525219232	*
4690	6031386	38	63	6.6380525219232	*
4690	6031394	38	63	6.6380525219232	*
4690	6031404	39	62	5.65215740723438	*
4690	6031416	40	61	4.74547639068669	*
4690	6031433	40	61	4.74547639068669	*
4690	6031458	41	60	3.91800947228012	*
4690	6031513	42	59	3.16975665201469	n.s.
4690	6031515	42	59	3.16975665201469	n.s.
4690	6031533	42	59	3.16975665201469	n.s.
4690	6031539	42	59	3.16975665201469	n.s.
4690	6031557	42	59	3.16975665201469	n.s.
4690	6031559	41	60	3.91800947228012	*

4690	6031570	41	60	3.91800947228012	*
4690	6031588	41	60	3.91800947228012	*
4690	6031617	42	59	3.16975665201469	n.s.
4690	6031622	41	60	3.91800947228012	*
4690	6031642	42	59	3.16975665201469	n.s.
4690	6031643	43	58	2.50071792989038	n.s.
4690	6031662	43	58	2.50071792989038	n.s.
4690	6031667	44	57	1.9108933059072	n.s.
4690	6031679	45	56	1.40028278006515	n.s.
4690	6031695	45	56	1.40028278006515	n.s.
4690	6031728	46	55	0.968886352364233	n.s.
4690	6031733	47	54	0.616704022804441	n.s.
4690	6031749	46	55	0.968886352364233	n.s.
4690	6031752	46	55	0.968886352364233	n.s.
4690	6031756	46	55	0.968886352364233	n.s.
4690	6031758	47	54	0.616704022804441	n.s.
4690	6031759	47	54	0.616704022804441	n.s.
4690	6031805	46	55	0.968886352364233	n.s.
4690	6031851	46	55	0.968886352364233	n.s.
4690	6031854	46	55	0.968886352364233	n.s.
4690	6031857	46	55	0.968886352364233	n.s.
4690	6031872	46	55	0.968886352364233	n.s.
4690	6031881	47	54	0.616704022804441	n.s.
4690	6031888	47	54	0.616704022804441	n.s.
4690	6031893	47	54	0.616704022804441	n.s.
4690	6031894	47	54	0.616704022804441	n.s.
4690	6031906	48	53	0.343735791385777	n.s.
4690	6031916	49	52	0.149981658108242	n.s.
4690	6031933	48	53	0.343735791385777	n.s.
4690	6031980	49	52	0.149981658108242	n.s.
4690	6031982	49	52	0.149981658108242	n.s.
4690	6031987	49	52	0.149981658108242	n.s.
4690	6031998	48	53	0.343735791385777	n.s.
4690	6032011	48	53	0.343735791385777	n.s.
4690	6032024	49	52	0.149981658108242	n.s.
4690	6032028	48	53	0.343735791385777	n.s.
4690	6032038	47	54	0.616704022804441	n.s.
4690	6032051	47	54	0.616704022804441	n.s.
4690	6032072	47	54	0.616704022804441	n.s.
4690	6032076	47	54	0.616704022804441	n.s.
4690	6032083	47	54	0.616704022804441	n.s.
4690	6032111	47	54	0.616704022804441	n.s.
4690	6032134	46	55	0.968886352364233	n.s.
4690	6032159	45	56	1.40028278006515	n.s.
4690	6032160	44	57	1.9108933059072	n.s.
4690	6032162	43	58	2.50071792989038	n.s.
4690	6032222	43	58	2.50071792989038	n.s.
4690	6032228	44	57	1.9108933059072	n.s.
4690	6032254	45	56	1.40028278006515	n.s.
4690	6032265	45	56	1.40028278006515	n.s.

4690	6032267	44	57	1.9108933059072	n.s.
4690	6032270	44	57	1.9108933059072	n.s.
4690	6032294	43	58	2.50071792989038	n.s.
4690	6032343	43	58	2.50071792989038	n.s.
4690	6032353	44	57	1.9108933059072	n.s.
4690	6032358	44	57	1.9108933059072	n.s.
4690	6032366	44	57	1.9108933059072	n.s.
4690	6032373	44	57	1.9108933059072	n.s.
4690	6032377	44	57	1.9108933059072	n.s.
4690	6032405	44	57	1.9108933059072	n.s.
4690	6032419	43	58	2.50071792989038	n.s.
4690	6032420	44	57	1.9108933059072	n.s.
4690	6032430	45	56	1.40028278006515	n.s.
4690	6032431	45	56	1.40028278006515	n.s.
4690	6032450	45	56	1.40028278006515	n.s.
4690	6032480	45	56	1.40028278006515	n.s.
4690	6032495	44	57	1.9108933059072	n.s.
4690	6032504	43	58	2.50071792989038	n.s.
4690	6032510	44	57	1.9108933059072	n.s.
4690	6032524	44	57	1.9108933059072	n.s.
4690	6032536	44	57	1.9108933059072	n.s.
4690	6032549	44	57	1.9108933059072	n.s.
4690	6032573	45	56	1.40028278006515	n.s.
4690	6032591	45	56	1.40028278006515	n.s.
4690	6032600	45	56	1.40028278006515	n.s.
4690	6032609	46	55	0.968886352364233	n.s.
4690	6032633	47	54	0.616704022804441	n.s.
4690	6032642	47	54	0.616704022804441	n.s.
4690	6032648	47	54	0.616704022804441	n.s.
4690	6032663	48	53	0.343735791385777	n.s.
4690	6032688	48	53	0.343735791385777	n.s.
4690	6032704	47	54	0.616704022804441	n.s.
4690	6032720	47	54	0.616704022804441	n.s.
4690	6032731	47	54	0.616704022804441	n.s.
4690	6032763	46	55	0.968886352364233	n.s.
4690	6032801	45	56	1.40028278006515	n.s.
4690	6032805	45	56	1.40028278006515	n.s.
4690	6032823	45	56	1.40028278006515	n.s.
4690	6032828	45	56	1.40028278006515	n.s.
4690	6032841	44	57	1.9108933059072	n.s.
4690	6032846	43	58	2.50071792989038	n.s.
4690	6032849	42	59	3.16975665201469	n.s.
4690	6032862	41	60	3.91800947228012	*
4690	6032884	41	60	3.91800947228012	*
4690	6032912	40	61	4.74547639068669	*
4690	6032922	40	61	4.74547639068669	*
4690	6032935	40	61	4.74547639068669	*
4690	6033010	41	60	3.91800947228012	*
4690	6033028	40	61	4.74547639068669	*
4690	6033031	39	62	5.65215740723438	*

4690	6033039	39	62	5.65215740723438	*
4690	6033041	39	62	5.65215740723438	*
4690	6033057	38	63	6.6380525219232	*
4690	6033100	37	64	7.70316173475315	**
4690	6033101	37	64	7.70316173475315	**
4690	6033126	36	65	8.84748504572423	**
4690	6033129	35	66	10.0710224548364	**
4690	6033159	35	66	10.0710224548364	**
4690	6033165	35	66	10.0710224548364	**
4690	6033203	35	66	10.0710224548364	**
4690	6033213	34	67	11.3737739620898	***
4690	6033227	34	67	11.3737739620898	***
4690	6033228	34	67	11.3737739620898	***
4690	6033295	34	67	11.3737739620898	***
4690	6033324	34	67	11.3737739620898	***
4690	6033334	34	67	11.3737739620898	***
4690	6033335	33	68	12.7557395674842	***
4690	6033357	34	67	11.3737739620898	***
4690	6033360	34	67	11.3737739620898	***
4690	6033441	35	66	10.0710224548364	**
4690	6033444	35	66	10.0710224548364	**
4690	6033465	35	66	10.0710224548364	**
4690	6033480	35	66	10.0710224548364	**
4690	6033481	34	67	11.3737739620898	***
4690	6033484	33	68	12.7557395674842	***
4690	6033501	34	67	11.3737739620898	***
4690	6033516	34	67	11.3737739620898	***
4690	6033548	33	68	12.7557395674842	***
4690	6033549	33	68	12.7557395674842	***
4690	6033653	34	67	11.3737739620898	***
4690	6033682	33	68	12.7557395674842	***
4690	6033700	33	68	12.7557395674842	***
4690	6033704	33	68	12.7557395674842	***
4690	6033717	33	68	12.7557395674842	***
4690	6033722	32	69	14.2169192710198	***
4690	6033729	31	70	15.7573130726965	***
4690	6033732	31	70	15.7573130726965	***
4690	6033753	32	69	14.2169192710198	***
4690	6033762	33	68	12.7557395674842	***
4690	6033766	33	68	12.7557395674842	***
4690	6033772	33	68	12.7557395674842	***
4690	6033773	32	69	14.2169192710198	***
4690	6033781	32	69	14.2169192710198	***
4690	6033789	33	68	12.7557395674842	***
4690	6033833	33	68	12.7557395674842	***
4690	6033839	34	67	11.3737739620898	***
4690	6033841	34	67	11.3737739620898	***
4690	6033846	33	68	12.7557395674842	***
4690	6033848	32	69	14.2169192710198	***
4690	6033850	31	70	15.7573130726965	***

4690	6033865	31	70	15.7573130726965	***
4690	6033875	32	69	14.2169192710198	***
4690	6033884	32	69	14.2169192710198	***
4690	6033899	32	69	14.2169192710198	***
4690	6033900	32	69	14.2169192710198	***
4690	6033902	31	70	15.7573130726965	***
4690	6033908	31	70	15.7573130726965	***
4690	6033910	32	69	14.2169192710198	***
4690	6033911	32	69	14.2169192710198	***
4690	6033918	32	69	14.2169192710198	***
4690	6033926	32	69	14.2169192710198	***
4690	6033950	33	68	12.7557395674842	***
4690	6033958	33	68	12.7557395674842	***
4690	6033974	32	69	14.2169192710198	***
4690	6034027	32	69	14.2169192710198	***
4690	6034044	32	69	14.2169192710198	***
4690	6034062	31	70	15.7573130726965	***
4690	6034072	31	70	15.7573130726965	***
4690	6034075	31	70	15.7573130726965	***
4690	6034100	30	71	17.3769209725144	***
4690	6034115	30	71	17.3769209725144	***
4690	6034116	31	70	15.7573130726965	***
4690	6034117	31	70	15.7573130726965	***
4690	6034121	30	71	17.3769209725144	***
4690	6034128	30	71	17.3769209725144	***
4690	6034138	31	70	15.7573130726965	***
4690	6034142	30	71	17.3769209725144	***
4690	6034143	29	72	19.0757429704734	***
4690	6034158	29	72	19.0757429704734	***
4690	6034160	30	71	17.3769209725144	***
4690	6034203	30	71	17.3769209725144	***
4690	6034205	31	70	15.7573130726965	***
4690	6034218	32	69	14.2169192710198	***
4690	6034233	33	68	12.7557395674842	***
4690	6034240	33	68	12.7557395674842	***
4690	6034248	34	67	11.3737739620898	***
4690	6034251	34	67	11.3737739620898	***
4690	6034260	34	67	11.3737739620898	***
4690	6034271	33	68	12.7557395674842	***
4690	6034296	33	68	12.7557395674842	***
4690	6034313	34	67	11.3737739620898	***
4690	6034328	35	66	10.0710224548364	**
4690	6034330	35	66	10.0710224548364	**
4690	6034333	35	66	10.0710224548364	**
4690	6034340	35	66	10.0710224548364	**
4690	6034355	36	65	8.84748504572423	**
4690	6034361	35	66	10.0710224548364	**
4690	6034371	36	65	8.84748504572423	**
4690	6034388	36	65	8.84748504572423	**
4690	6034394	36	65	8.84748504572423	**

4690	6034401	36	65	8.84748504572423	**
4690	6034404	37	64	7.70316173475315	**
4690	6034412	38	63	6.6380525219232	*
4690	6034420	39	62	5.65215740723438	*
4690	6034424	40	61	4.74547639068669	*
4690	6034428	40	61	4.74547639068669	*
4690	6034436	40	61	4.74547639068669	*
4690	6034441	40	61	4.74547639068669	*
4690	6034442	40	61	4.74547639068669	*
4690	6034454	39	62	5.65215740723438	*
4690	6034463	38	63	6.6380525219232	*
4690	6034475	38	63	6.6380525219232	*
4690	6034486	37	64	7.70316173475315	**
4690	6034500	36	65	8.84748504572423	**
4690	6034511	36	65	8.84748504572423	**
4690	6034514	36	65	8.84748504572423	**
4690	6034527	37	64	7.70316173475315	**
4690	6034533	38	63	6.6380525219232	*
4690	6034549	38	63	6.6380525219232	*
4690	6034550	39	62	5.65215740723438	*
4690	6034559	39	62	5.65215740723438	*
4690	6034565	40	61	4.74547639068669	*
4690	6034586	39	62	5.65215740723438	*
4690	6034617	39	62	5.65215740723438	*
4690	6034628	39	62	5.65215740723438	*
4690	6034630	40	61	4.74547639068669	*
4690	6034634	40	61	4.74547639068669	*
4690	6034636	40	61	4.74547639068669	*
4690	6034681	41	60	3.91800947228012	*
4690	6034685	41	60	3.91800947228012	*
4690	6034687	40	61	4.74547639068669	*
4690	6034692	40	61	4.74547639068669	*
4690	6034698	40	61	4.74547639068669	*
4690	6034699	40	61	4.74547639068669	*
4690	6034702	40	61	4.74547639068669	*
4690	6034744	41	60	3.91800947228012	*
4690	6034746	42	59	3.16975665201469	n.s.
4690	6034751	43	58	2.50071792989038	n.s.
4690	6034760	43	58	2.50071792989038	n.s.
4690	6035234	43	58	2.50071792989038	n.s.
4690	6035286	44	57	1.9108933059072	n.s.
4690	6035289	45	56	1.40028278006515	n.s.
4690	6035295	45	56	1.40028278006515	n.s.
4690	6035298	45	56	1.40028278006515	n.s.
4690	6035335	45	56	1.40028278006515	n.s.
4690	6035344	45	56	1.40028278006515	n.s.
4690	6035356	44	57	1.9108933059072	n.s.
4690	6035387	44	57	1.9108933059072	n.s.
4690	6035399	45	56	1.40028278006515	n.s.
4690	6035494	46	55	0.968886352364233	n.s.

4690	6035517	47	54	0.616704022804441	n.s.	
4690	6035610	48	53	0.343735791385777	n.s.	
4690	6035611	48	53	0.343735791385777	n.s.	
4690	6035621	49	52	0.149981658108242	n.s.	
4690	6035678	49	52	0.149981658108242	n.s.	
4690	6036032	50	51	0.0354416229718356	n.s.	
4690	6036043	51	50	0.000115685976557532		n.s.
4690	6036086	52	49	0.044003847122408	n.s.	
4690	6036093	51	50	0.000115685976557532		n.s.
4690	6036156	52	49	0.044003847122408	n.s.	
4690	6036176	52	49	0.044003847122408	n.s.	
4690	6036211	53	48	0.167106106409387	n.s.	
4690	6036263	53	48	0.167106106409387	n.s.	
4690	6036298	53	48	0.167106106409387	n.s.	
4690	6036320	54	47	0.369422463837495	n.s.	
4690	6036327	55	46	0.650952919406731	n.s.	
4690	6036363	55	46	0.650952919406731	n.s.	
4690	6036365	56	45	1.0116974731171	n.s.	
4690	6036370	57	44	1.45165612496859	n.s.	
4690	6036377	57	44	1.45165612496859	n.s.	
4690	6036382	57	44	1.45165612496859	n.s.	
4690	6036384	57	44	1.45165612496859	n.s.	
4690	6036427	56	45	1.0116974731171	n.s.	
4690	6036564	56	45	1.0116974731171	n.s.	
4690	6036691	57	44	1.45165612496859	n.s.	
4690	6036720	57	44	1.45165612496859	n.s.	
4690	6036893	57	44	1.45165612496859	n.s.	
4690	6036896	57	44	1.45165612496859	n.s.	
4690	6036901	57	44	1.45165612496859	n.s.	
4690	6036905	57	44	1.45165612496859	n.s.	
4690	6036909	56	45	1.0116974731171	n.s.	
4690	6036919	55	46	0.650952919406731	n.s.	
4690	6036922	54	47	0.369422463837495	n.s.	
4690	6036949	53	48	0.167106106409387	n.s.	
4690	6036950	52	49	0.044003847122408	n.s.	
4690	6036963	52	49	0.044003847122408	n.s.	
4690	6036975	51	50	0.000115685976557532		n.s.
4690	6036976	52	49	0.044003847122408	n.s.	
4690	6036984	51	50	0.000115685976557532		n.s.
4690	6037231	51	50	0.000115685976557532		n.s.
4690	6037404	51	50	0.000115685976557532		n.s.
4690	6037485	50	51	0.0354416229718356	n.s.	
4690	6037607	49	52	0.149981658108242	n.s.	
4690	6037610	49	52	0.149981658108242	n.s.	
4690	6037638	49	52	0.149981658108242	n.s.	
4690	6037646	48	53	0.343735791385777	n.s.	
4690	6037705	48	53	0.343735791385777	n.s.	
4690	6037719	48	53	0.343735791385777	n.s.	
4690	6037766	47	54	0.616704022804441	n.s.	
4690	6037767	46	55	0.968886352364233	n.s.	

4690	6037770	46	55	0.968886352364233	n.s.
4690	6037771	46	55	0.968886352364233	n.s.
4690	6037772	45	56	1.40028278006515	n.s.
4690	6037780	45	56	1.40028278006515	n.s.
4690	6037784	45	56	1.40028278006515	n.s.
4690	6037799	44	57	1.9108933059072	n.s.
4690	6037820	44	57	1.9108933059072	n.s.
4690	6037826	44	57	1.9108933059072	n.s.
4690	6037832	45	56	1.40028278006515	n.s.
4690	6037886	45	56	1.40028278006515	n.s.
4690	6037903	46	55	0.968886352364233	n.s.
4690	6037933	47	54	0.616704022804441	n.s.
4690	6037985	47	54	0.616704022804441	n.s.
4690	6038096	48	53	0.343735791385777	n.s.
4690	6038111	48	53	0.343735791385777	n.s.
4690	6038112	49	52	0.149981658108242	n.s.
4690	6038121	50	51	0.0354416229718356	n.s.
4690	6038192	49	52	0.149981658108242	n.s.
4690	6038232	49	52	0.149981658108242	n.s.
4690	6038240	49	52	0.149981658108242	n.s.
4690	6038256	49	52	0.149981658108242	n.s.
4690	6038268	48	53	0.343735791385777	n.s.
4690	6038414	48	53	0.343735791385777	n.s.
4690	6038426	48	53	0.343735791385777	n.s.
4690	6038460	48	53	0.343735791385777	n.s.
4690	6038486	48	53	0.343735791385777	n.s.
4690	6038560	48	53	0.343735791385777	n.s.
4690	6038564	48	53	0.343735791385777	n.s.
4690	6038584	48	53	0.343735791385777	n.s.
4690	6038589	48	53	0.343735791385777	n.s.
4690	6038630	48	53	0.343735791385777	n.s.
4690	6038650	48	53	0.343735791385777	n.s.
4690	6038674	49	52	0.149981658108242	n.s.
4690	6038681	50	51	0.0354416229718356	n.s.
4690	6038686	51	50	0.000115685976557532	n.s.
4690	6038692	52	49	0.044003847122408	n.s.
4690	6038698	51	50	0.000115685976557532	n.s.
4690	6038732	50	51	0.0354416229718356	n.s.
4690	6038748	49	52	0.149981658108242	n.s.
4690	6038766	48	53	0.343735791385777	n.s.
4690	6038787	47	54	0.616704022804441	n.s.
4690	6038814	46	55	0.968886352364233	n.s.
4690	6038832	46	55	0.968886352364233	n.s.
4690	6038859	47	54	0.616704022804441	n.s.
4690	6038869	47	54	0.616704022804441	n.s.
4690	6038899	46	55	0.968886352364233	n.s.
4690	6038962	46	55	0.968886352364233	n.s.
4690	6038964	46	55	0.968886352364233	n.s.
4690	6038990	46	55	0.968886352364233	n.s.
4690	6039012	46	55	0.968886352364233	n.s.

4690	6039020	46	55	0.968886352364233	n.s.
4690	6039024	46	55	0.968886352364233	n.s.
4690	6039032	46	55	0.968886352364233	n.s.
4690	6039037	46	55	0.968886352364233	n.s.
4690	6039056	45	56	1.40028278006515	n.s.
4690	6039058	45	56	1.40028278006515	n.s.
4690	6039082	44	57	1.9108933059072	n.s.
4690	6039086	43	58	2.50071792989038	n.s.
4690	6039091	43	58	2.50071792989038	n.s.
4690	6039109	43	58	2.50071792989038	n.s.
4690	6039123	42	59	3.16975665201469	n.s.
4690	6039124	42	59	3.16975665201469	n.s.
4690	6039136	42	59	3.16975665201469	n.s.
4690	6039242	42	59	3.16975665201469	n.s.
4690	6039253	41	60	3.91800947228012	*
4690	6039320	40	61	4.74547639068669	*
4690	6039348	40	61	4.74547639068669	*
4690	6039373	41	60	3.91800947228012	*
4690	6039374	40	61	4.74547639068669	*
4690	6039429	39	62	5.65215740723438	*
4690	6039439	39	62	5.65215740723438	*
4690	6039464	40	61	4.74547639068669	*
4690	6039473	41	60	3.91800947228012	*
4690	6039528	42	59	3.16975665201469	n.s.
4690	6039549	43	58	2.50071792989038	n.s.
4690	6039561	43	58	2.50071792989038	n.s.
4690	6039599	44	57	1.9108933059072	n.s.
4690	6039601	43	58	2.50071792989038	n.s.
4690	6039602	43	58	2.50071792989038	n.s.
4690	6039603	44	57	1.9108933059072	n.s.
4690	6039604	44	57	1.9108933059072	n.s.
4690	6039609	45	56	1.40028278006515	n.s.
4690	6039627	45	56	1.40028278006515	n.s.
4690	6039634	45	56	1.40028278006515	n.s.
4690	6039640	46	55	0.968886352364233	n.s.
4690	6039647	46	55	0.968886352364233	n.s.
4690	6039677	46	55	0.968886352364233	n.s.
4690	6039718	47	54	0.616704022804441	n.s.
4690	6039786	47	54	0.616704022804441	n.s.
4690	6039795	48	53	0.343735791385777	n.s.
4690	6039796	48	53	0.343735791385777	n.s.
4690	6039901	47	54	0.616704022804441	n.s.
4690	6039927	47	54	0.616704022804441	n.s.
4690	6039953	48	53	0.343735791385777	n.s.
4690	6040282	48	53	0.343735791385777	n.s.
4690	6040313	48	53	0.343735791385777	n.s.
4690	6040339	49	52	0.149981658108242	n.s.
4690	6040375	48	53	0.343735791385777	n.s.
4690	6040376	48	53	0.343735791385777	n.s.
4690	6040377	48	53	0.343735791385777	n.s.

4690	6040378	48	53	0.343735791385777	n.s.	
4690	6040411	48	53	0.343735791385777	n.s.	
4690	6040414	48	53	0.343735791385777	n.s.	
4690	6040442	49	52	0.149981658108242	n.s.	
4690	6040481	49	52	0.149981658108242	n.s.	
4690	6040546	49	52	0.149981658108242	n.s.	
4690	6040581	50	51	0.0354416229718356	n.s.	
4690	6040584	51	50	0.000115685976557532		n.s.
4690	6040613	51	50	0.000115685976557532		n.s.
4690	6040617	51	50	0.000115685976557532		n.s.
4690	6040649	52	49	0.044003847122408	n.s.	
4690	6040670	51	50	0.000115685976557532		n.s.
4690	6040681	50	51	0.0354416229718356	n.s.	
4690	6040686	51	50	0.000115685976557532		n.s.
4690	6041024	51	50	0.000115685976557532		n.s.
4690	6041043	52	49	0.044003847122408	n.s.	
4690	6041219	51	50	0.000115685976557532		n.s.
4690	6041224	51	50	0.000115685976557532		n.s.
4690	6041267	51	50	0.000115685976557532		n.s.
4690	6041273	50	51	0.0354416229718356	n.s.	
4690	6041330	50	51	0.0354416229718356	n.s.	
4690	6041339	49	52	0.149981658108242	n.s.	
4690	6041387	50	51	0.0354416229718356	n.s.	
4690	6041389	50	51	0.0354416229718356	n.s.	
4690	6041391	51	50	0.000115685976557532		n.s.
4690	6041394	52	49	0.044003847122408	n.s.	
4690	6041396	53	48	0.167106106409387	n.s.	
4690	6041414	53	48	0.167106106409387	n.s.	
4690	6041415	52	49	0.044003847122408	n.s.	
4690	6041417	52	49	0.044003847122408	n.s.	
4690	6041437	52	49	0.044003847122408	n.s.	
4690	6041442	52	49	0.044003847122408	n.s.	
4690	6041472	53	48	0.167106106409387	n.s.	
4690	6041473	52	49	0.044003847122408	n.s.	
4690	6041496	51	50	0.000115685976557532		n.s.
4690	6041505	50	51	0.0354416229718356	n.s.	
4690	6041512	49	52	0.149981658108242	n.s.	
4690	6041533	49	52	0.149981658108242	n.s.	
4690	6041546	49	52	0.149981658108242	n.s.	
4690	6041547	49	52	0.149981658108242	n.s.	
4690	6041556	50	51	0.0354416229718356	n.s.	
4690	6041580	51	50	0.000115685976557532		n.s.
4690	6041583	51	50	0.000115685976557532		n.s.
4690	6041598	51	50	0.000115685976557532		n.s.
4690	6041610	50	51	0.0354416229718356	n.s.	
4690	6041612	50	51	0.0354416229718356	n.s.	
4690	6041616	50	51	0.0354416229718356	n.s.	
4690	6041621	50	51	0.0354416229718356	n.s.	
4690	6041653	50	51	0.0354416229718356	n.s.	
4690	6041655	50	51	0.0354416229718356	n.s.	

4690	6041656	49	52	0.149981658108242	n.s.
4690	6041693	48	53	0.343735791385777	n.s.
4690	6041705	47	54	0.616704022804441	n.s.
4690	6041715	46	55	0.968886352364233	n.s.
4690	6041716	45	56	1.40028278006515	n.s.
4690	6041717	45	56	1.40028278006515	n.s.
4690	6041721	45	56	1.40028278006515	n.s.
4690	6041723	45	56	1.40028278006515	n.s.
4690	6041764	45	56	1.40028278006515	n.s.
4690	6041765	45	56	1.40028278006515	n.s.
4690	6041773	46	55	0.968886352364233	n.s.
4690	6041861	47	54	0.616704022804441	n.s.
4690	6041863	48	53	0.343735791385777	n.s.
4690	6042186	48	53	0.343735791385777	n.s.
4690	6042274	47	54	0.616704022804441	n.s.
4690	6042327	48	53	0.343735791385777	n.s.
4690	6042339	48	53	0.343735791385777	n.s.
4690	6042417	47	54	0.616704022804441	n.s.
4690	6042719	46	55	0.968886352364233	n.s.
4690	6042753	47	54	0.616704022804441	n.s.
4690	6042852	48	53	0.343735791385777	n.s.
4690	6042892	49	52	0.149981658108242	n.s.
4690	6042896	49	52	0.149981658108242	n.s.
4690	6042898	49	52	0.149981658108242	n.s.
4690	6042910	48	53	0.343735791385777	n.s.
4690	6042913	48	53	0.343735791385777	n.s.
4690	6042923	48	53	0.343735791385777	n.s.
4690	6042974	48	53	0.343735791385777	n.s.
4690	6042982	49	52	0.149981658108242	n.s.
4690	6042995	50	51	0.0354416229718356	n.s.
4690	6043006	49	52	0.149981658108242	n.s.
4690	6043015	49	52	0.149981658108242	n.s.
4690	6043036	49	52	0.149981658108242	n.s.
4690	6043076	49	52	0.149981658108242	n.s.
4690	6043111	50	51	0.0354416229718356	n.s.
4690	6043126	50	51	0.0354416229718356	n.s.
4690	6043148	51	50	0.000115685976557532	n.s.
4690	6043155	52	49	0.044003847122408	n.s.
4690	6043170	52	49	0.044003847122408	n.s.
4690	6043192	52	49	0.044003847122408	n.s.
4690	6043573	52	49	0.044003847122408	n.s.
4690	6043605	53	48	0.167106106409387	n.s.
4690	6043612	53	48	0.167106106409387	n.s.
4690	6043624	54	47	0.369422463837495	n.s.
4690	6043628	54	47	0.369422463837495	n.s.
4690	6043671	54	47	0.369422463837495	n.s.
4690	6043689	55	46	0.650952919406731	n.s.
4690	6043782	55	46	0.650952919406731	n.s.
4690	6043849	55	46	0.650952919406731	n.s.
4690	6043860	55	46	0.650952919406731	n.s.

4690	6043885	55	46	0.650952919406731	n.s.
4690	6043899	55	46	0.650952919406731	n.s.
4690	6043905	56	45	1.0116974731171	n.s.
4690	6043911	56	45	1.0116974731171	n.s.
4690	6043974	55	46	0.650952919406731	n.s.
4690	6044003	54	47	0.369422463837495	n.s.
4690	6044032	53	48	0.167106106409387	n.s.
4690	6044458	52	49	0.044003847122408	n.s.
4690	6044459	51	50	0.000115685976557532	n.s.
4690	6044491	51	50	0.000115685976557532	n.s.
4690	6044492	50	51	0.0354416229718356	n.s.
4690	6044494	49	52	0.149981658108242	n.s.
4690	6044495	49	52	0.149981658108242	n.s.
4690	6044496	50	51	0.0354416229718356	n.s.
4690	6044523	49	52	0.149981658108242	n.s.
4690	6044662	48	53	0.343735791385777	n.s.
4690	6044687	48	53	0.343735791385777	n.s.
4690	6044857	48	53	0.343735791385777	n.s.
4690	6045058	48	53	0.343735791385777	n.s.
4690	6045059	49	52	0.149981658108242	n.s.
4690	6045067	50	51	0.0354416229718356	n.s.
4690	6045072	50	51	0.0354416229718356	n.s.
4690	6045187	51	50	0.000115685976557532	n.s.
4690	6045189	50	51	0.0354416229718356	n.s.
4690	6045342	50	51	0.0354416229718356	n.s.
4690	6045358	50	51	0.0354416229718356	n.s.
4690	6045359	49	52	0.149981658108242	n.s.
4690	6045364	48	53	0.343735791385777	n.s.
4690	6045365	48	53	0.343735791385777	n.s.
4690	6045368	49	52	0.149981658108242	n.s.
4690	6045384	48	53	0.343735791385777	n.s.
4690	6045704	48	53	0.343735791385777	n.s.
4690	6045891	48	53	0.343735791385777	n.s.
4690	6045902	48	53	0.343735791385777	n.s.
4690	6045915	49	52	0.149981658108242	n.s.
4690	6045920	50	51	0.0354416229718356	n.s.
4690	6045921	50	51	0.0354416229718356	n.s.
4690	6045930	50	51	0.0354416229718356	n.s.
4690	6045936	51	50	0.000115685976557532	n.s.
4690	6045938	52	49	0.044003847122408	n.s.
4690	6045955	53	48	0.167106106409387	n.s.
4690	6045958	53	48	0.167106106409387	n.s.
4690	6045972	53	48	0.167106106409387	n.s.
4690	6045987	54	47	0.369422463837495	n.s.
4690	6046007	54	47	0.369422463837495	n.s.
4690	6046019	54	47	0.369422463837495	n.s.
4690	6046649	54	47	0.369422463837495	n.s.
4690	6046652	55	46	0.650952919406731	n.s.
4690	6046700	55	46	0.650952919406731	n.s.
4690	6046710	56	45	1.0116974731171	n.s.

4690	6046856	56	45	1.0116974731171	n.s.	
4690	6046866	57	44	1.45165612496859	n.s.	
4690	6046876	58	43	1.97082887496121	n.s.	
4690	6047011	58	43	1.97082887496121	n.s.	
4690	6047039	59	42	2.56921572309496	n.s.	
4690	6047089	60	41	3.24681666936984	n.s.	
4690	6047098	61	40	4.00363171378585	*	
4690	6047099	62	39	4.83966085634298	*	
4690	6047123	62	39	4.83966085634298	*	
4690	6047136	63	38	5.75490409704125	*	
4690	6047150	62	39	4.83966085634298	*	
4690	6047153	61	40	4.00363171378585	*	
4690	6047275	60	41	3.24681666936984	n.s.	
4690	6047281	59	42	2.56921572309496	n.s.	
4690	6047298	58	43	1.97082887496121	n.s.	
4690	6047301	58	43	1.97082887496121	n.s.	
4690	6047320	57	44	1.45165612496859	n.s.	
4690	6047327	57	44	1.45165612496859	n.s.	
4690	6047328	57	44	1.45165612496859	n.s.	
4690	6047333	58	43	1.97082887496121	n.s.	
4690	6047340	58	43	1.97082887496121	n.s.	
4690	6047341	58	43	1.97082887496121	n.s.	
4690	6047360	58	43	1.97082887496121	n.s.	
4690	6047367	57	44	1.45165612496859	n.s.	
4690	6047371	57	44	1.45165612496859	n.s.	
4690	6047373	57	44	1.45165612496859	n.s.	
4690	6047375	57	44	1.45165612496859	n.s.	
4690	6047376	57	44	1.45165612496859	n.s.	
4690	6047384	56	45	1.0116974731171	n.s.	
4690	6047386	55	46	0.650952919406731	n.s.	
4690	6047401	54	47	0.369422463837495	n.s.	
4690	6047419	55	46	0.650952919406731	n.s.	
4690	6047422	56	45	1.0116974731171	n.s.	
4690	6047424	56	45	1.0116974731171	n.s.	
4690	6047427	56	45	1.0116974731171	n.s.	
4690	6047429	55	46	0.650952919406731	n.s.	
4690	6047439	55	46	0.650952919406731	n.s.	
4690	6047449	54	47	0.369422463837495	n.s.	
4690	6047455	54	47	0.369422463837495	n.s.	
4690	6047460	53	48	0.167106106409387	n.s.	
4690	6047463	52	49	0.044003847122408	n.s.	
4690	6047464	51	50	0.000115685976557532		n.s.
4690	6047469	51	50	0.000115685976557532		n.s.
4690	6047478	51	50	0.000115685976557532		n.s.
4690	6047485	51	50	0.000115685976557532		n.s.
4690	6047487	51	50	0.000115685976557532		n.s.
4690	6047522	51	50	0.000115685976557532		n.s.
4690	6047535	51	50	0.000115685976557532		n.s.
4690	6047577	51	50	0.000115685976557532		n.s.
4690	6047587	52	49	0.044003847122408	n.s.	

4690	6047593	52	49	0.044003847122408	n.s.
4690	6047596	52	49	0.044003847122408	n.s.
4690	6047597	52	49	0.044003847122408	n.s.
4690	6047599	52	49	0.044003847122408	n.s.
4690	6047608	53	48	0.167106106409387	n.s.
4690	6047613	54	47	0.369422463837495	n.s.
4690	6047616	55	46	0.650952919406731	n.s.
4690	6047618	56	45	1.0116974731171	n.s.
4690	6047641	56	45	1.0116974731171	n.s.
4690	6047731	57	44	1.45165612496859	n.s.
4690	6047740	57	44	1.45165612496859	n.s.
4690	6047757	57	44	1.45165612496859	n.s.
4690	6048034	57	44	1.45165612496859	n.s.
4690	6048035	57	44	1.45165612496859	n.s.
4690	6048062	56	45	1.0116974731171	n.s.
4690	6048081	56	45	1.0116974731171	n.s.
4690	6048112	56	45	1.0116974731171	n.s.
4690	6048152	55	46	0.650952919406731	n.s.
4690	6048184	55	46	0.650952919406731	n.s.
4690	6048188	55	46	0.650952919406731	n.s.
4690	6048204	55	46	0.650952919406731	n.s.
4690	6048215	55	46	0.650952919406731	n.s.
4690	6048217	55	46	0.650952919406731	n.s.
4690	6048220	55	46	0.650952919406731	n.s.
4690	6048268	55	46	0.650952919406731	n.s.
4690	6048293	54	47	0.369422463837495	n.s.
4690	6048298	53	48	0.167106106409387	n.s.
4690	6048300	54	47	0.369422463837495	n.s.
4690	6048319	54	47	0.369422463837495	n.s.
4690	6048325	53	48	0.167106106409387	n.s.
4690	6048332	52	49	0.044003847122408	n.s.
4690	6048351	52	49	0.044003847122408	n.s.
4690	6048401	51	50	0.000115685976557532	n.s.
4690	6048406	50	51	0.0354416229718356	n.s.
4690	6048472	49	52	0.149981658108242	n.s.
4690	6048524	48	53	0.343735791385777	n.s.
4690	6048568	47	54	0.616704022804441	n.s.
4690	6048571	47	54	0.616704022804441	n.s.
4690	6048585	47	54	0.616704022804441	n.s.
4690	6048590	46	55	0.968886352364233	n.s.
4690	6048594	46	55	0.968886352364233	n.s.
4690	6048604	45	56	1.40028278006515	n.s.
4690	6048605	44	57	1.9108933059072	n.s.
4690	6048620	43	58	2.50071792989038	n.s.
4690	6048652	42	59	3.16975665201469	n.s.
4690	6048834	41	60	3.91800947228012	*
4690	6048970	41	60	3.91800947228012	*
4690	6048983	41	60	3.91800947228012	*
4690	6048984	40	61	4.74547639068669	*
4690	6048986	40	61	4.74547639068669	*

4690	6049131	39	62	5.65215740723438	*
4690	6049133	40	61	4.74547639068669	*
4690	6049203	40	61	4.74547639068669	*
4690	6049213	40	61	4.74547639068669	*
4690	6049248	40	61	4.74547639068669	*
4690	6049255	39	62	5.65215740723438	*
4690	6049269	38	63	6.6380525219232	*
4690	6049294	38	63	6.6380525219232	*
4690	6049307	38	63	6.6380525219232	*
4690	6049310	39	62	5.65215740723438	*
4690	6049457	39	62	5.65215740723438	*
4690	6049462	39	62	5.65215740723438	*
4690	6049486	40	61	4.74547639068669	*
4690	6049525	41	60	3.91800947228012	*
4690	6049532	42	59	3.16975665201469	n.s.
4690	6049628	43	58	2.50071792989038	n.s.
4690	6049669	44	57	1.9108933059072	n.s.
4690	6049712	45	56	1.40028278006515	n.s.
4690	6049782	45	56	1.40028278006515	n.s.
4690	6049838	46	55	0.968886352364233	n.s.
4690	6049846	46	55	0.968886352364233	n.s.
4690	6049847	46	55	0.968886352364233	n.s.
4690	6049854	46	55	0.968886352364233	n.s.
4690	6049855	46	55	0.968886352364233	n.s.
4690	6049872	47	54	0.616704022804441	n.s.
4690	6049896	46	55	0.968886352364233	n.s.
4690	6049907	47	54	0.616704022804441	n.s.
4690	6049915	46	55	0.968886352364233	n.s.
4690	6049916	46	55	0.968886352364233	n.s.
4690	6049933	47	54	0.616704022804441	n.s.
4690	6049941	48	53	0.343735791385777	n.s.
4690	6049959	48	53	0.343735791385777	n.s.
4690	6049998	47	54	0.616704022804441	n.s.

Supplementary Table 2 | Data for locus-based HKA test. Polymorphism and divergence counts at the *t*_MSE-containing locus and neighbouring loci.

Locus	Polymorphism	Divergence
*t*_MSE	135	88
5 kb upstream	45	68
10 kb downstream	91	150

Supplementary Note 1 | Alignment of the t_MSE region. Dore= D. orena, D_ = dark haplogroup, L_= light haplogroup, ECAM = erecta from Cameroon, EGAB = erecta from Gabon.

>Dore
CCATGGAATCCGAGCACCTGGTATAGCCGCAAGTGGAACTGCAGCACCCGACCCAGAC
GGCCGCTCATTGTTGACTACGCGAAAGCGAAACCCTTCAG-ATGGACACG------
CCTTATCCCAGTGGCCGATC--TC-CCCTT-
AAATGGGCCACCAAACAACTGACGAATCCATTAGCCTAAGTGAATTTGTCAAAAACACT
GAGAACAGTAATCCATGTAGCTT---------CCTTGATTTCCGTACTT-
AAATGAATATAAGCGACCAGAAACTAAAGATTGCCTGTATC------------------------
TACACTGTATGTTTTATAATTTTATAGGTGGAATCAAGGCTTAGGATAATTGCATTTAGTAG
TGCACTTAAATTCCAAT-GCTAAATGGACCGGA----TCCTAAAGTTGCGATCAATAA-
AAAAATAAGTGCATTAAAATGGTCCAAGCACATTAGAATACTAATTTTTGGTGTTTAATTA
AACTAATTTGCGAATTCAAGATCATAGTATATTAGTATACAATATATAGTATAGTGTATAGTA
TATATATATTAGTATATTAATTAACCAGTCTC-TTTTTTT-
ATTACTCCAACTATTGAAATTTGCGTTTTTATTACTAGATAA------
TTTCCAAGTGCCTTTTTTGCATTTCGAACT-GCTAGAATTGCGTTGAAAGA-
AACATGATCATTAAATTGATA----
TTTCAAAGGAGTTTGTCTCTTGCCAATTACCTATAAAATGTAGTTTTCCGGAAAATGTGA
AAACATTTTGTT
>D_ECAM13
CCATGGAAGCCGAGCACCTGGTAGAGCCGCAAGTGGAACTGCAGCACCCGACCCAGAC
GGCCGCTCATTGTTGACTACGCGAAGGCGAAACCCTTTAC-
ATGGACAGGGACAGGCCTTATCCC-------GATC-
ATCTCCCTTCAAATGGGCCACCAAACAACTGACAAATCCATTAGCCTAAGTGAATTTGTG
AAAAACACTGAGAACAGTAAT--------------------CCTTGATTTCCGTATGT-
AAATGAATATAAACGACCAGAATGTAAATATTGCCTGTATC------------------------
TACATTTCATGTTTTATAATTTTATAGGCAGAATCAAGGCTTAGGATAATTGCATTTAGTAG
TGTACTTAAATTCCAAT-GCTAAATGAACCGGT----ATCTACAGTTGCGATCAATAA-
AAAAATAAGTGCATTAAAATAGTCCAAGAACATTAGAATACTAATTTTTGGTGTTTAATTA
AACTATTTTGCGAATTCAAGATCATA--ATT-
GTATATTAATTGGACAGTCTC-TTTTTTT-
ATTACTCCAACTATTCAAATTTTCGTTTTTATTACTAGATAA------
TTTCCTAGTGCTTTTTGTGCATTTCGAACT-ACTAGAATTGTGTTGAAAGA-
AACATGAACATTAAATTGATACACATTTTCAAGGAGTTTGCCTTTTGCCAATTACCAATAA
AATGTAG-TTTCCGGAAGATCTAAAAACATTTCGTT
>D_ECAM14
CCATGGAAGCCGAGCACCTGGTAGAGCCGCAAGTGGAACTGCAGCACCCGACCCAGAC
GGCCGCTCATTGTTGACTACGCGAAGGCGAAACCCTTTAC-
ATGGACAGGGACAGGCCTTATCCCAGCGGCCGATC-
ATCTGCCTTCAAATGGGCCACCAAACAACTGACAAATCCATTAGCCTAAGTGAATTTGTG
AAAAACACTGAGAACAGTAAT--------------------CCTTGATTTCCGTATGT-
AAATGAATATAAACGACCAGAATGTAAATATTGCCTGTATC------------------------
TACATTTTATGTTTTATAATTTTATAGGCAGAATCAAGGCTTAGGATAATTGCATTTAGTAG
TGTACTTAAATTCCAAT-GCTAAATGAACCGGT----ATCTAAAGTTGCGATCAATAA-
AAAAATAAGTGCATTAAAATAGTCCAAGAACATTAGAATACTAATTTTTGGTGTTTAATTA
ACCTATTTTGCGAATTCAAGATCATA--ATT-
GTATATTAATTGGACAGTCTC-TTTTTTT-
ATTACTCCAACTATTCAAATTTTCGTTTTTATTACTAGATAA------

TTTCCTAGTGCTTTTTGTGCATTTCGAACT-GCTAGAATTGTGTTGAAATA-
AACATGAACATTAAATTGATACACATTTCAAAGGAGTTTGCCTTTTGCCAATTACCAATA
AAATGTAG-TTTCCGGAAGATCTAAAAACATTTCGGT
>L_ECAM15
CCATGGAAGCCGAGCACCTGGTAGAGCCGCAAGTGGAACTGCAGCACCCGACCCAGAC
GGCCGCTCATTGTTGACTACGCGAAGGCGAAACCCTTTAC-
ATGGACAGGGACAGGCCTTATCCCAGCGGCCGATC-
ATCTGCCTTCAAATGGGCCACCAAACAACTGACAAATCCATTAGCCTAAGTGAATTTGTG
AAAAACACTGAGAACAGTAATCCATGTAGCTT---------CCTTGATTTCCGTATTT-
AAATGAACACAAACCACCAGAAAGTAAAGATTGTCTGTGTC------------------------
TACATTATATGTTTTATAATTTTATAGGTAGAATCAAGGCTTAGGATAATTGCATTTAGTAGT
ATACTTAAATTCCAAT-GCTAAATGAACCGGT----
ATCTAAAGTTGCAACCAATAACAAAAATATGTGCATTAAAATGTTCTTGGACCATTAGAA
TATTAAATTTATTAGTT-AAATAAACTAATTTGCGAATTCAAGATCATA---------------------------
-----------------ATTTGTATATTAAGTGGACAGTCTC-TTTTTTT-ATTACTCAAACTATTT------
GCGATTTTATCACTAGATAA------TTTCCAAGTGGGTTTTGTGCGTTTCGAACT-
GCTAGAATTGTGTTGAAAGA-------AACATTGATATTATA----
TTTCAAAGGAGTTTGCCTTTTGCCAATTACCTATAAAA----------------
TTGTAAAAACATTTCGCT
>L_ECAM16
CCATGGAAGCCGAGCACCTGGTAGAGCCGCAAGTGGAACTGCAGCACCCGACCCAGAC
GGCCGCTCATTGTTGACTACGCGAAGGCGAAACCCTTTAC-
ATGGACAGGGACAGGNCTTATCCC-------GATC-
ATCTCCCTTCAAATGGGCCACCAAACAACTGACAAATCCATTAGCCTAAGTGAATTTGTG
AAAAACACTGAGAACAGTA-TCCATGTAGCTT---------CCTTGATTTCCGTATTT-
AAATGAACACAAACCACCAGAAAGTAAAGATTGTCTGTGTC------------------------
TACATTATATGTTTTATAATATTATAGGCAGAATCAAGGCTTAGGATAATTGCATTTAGTAG
TATACTTAAATTCCAAT-GCTAAATGAACCGGT----
ATCTAAAGTTGCAACCAATAACAAAAATATGTGCATTAAAATGTTCTTGGACCATTAGAA
TATTAAATTTATTAGTT-AAATAAACTAATTTGCGAATTCAAGATCATA---------------------------
-----------------ATTTGTATATTAAGTGGACAGTCTC-TTTTTTTTATTACTCAAACTATTT------
GCGATTTTATCACTAGATAA------TTTCCAAGTGGGGTTTGTGCGTTTCGAACT-
GCTAGAATTGTGTTGAAAGA-------AACATTGATATTATA----
TTTCAAAGGAGTTTGCCTTTTGCCAATTACCTATAAAA----------------
TTGTAAAAACTTTTCGCT
>D_ECAM19
CCATGGAAGCCGAGCACCTGGTAGAGCCGCAAGTGGAACTGCAGCACCCGACCCAGAC
GGCCGCTCATTGTTGACTACGCGAAGGCGAAACCCTTTAC-
ATGGACAGGGACAGGGCTTATCCC-------GATC-
ATCTCCCTTCAAATGGGCCACCAAACAACTGACAAATCCATTAGCCTAAGTGAATTTGTG
AAAAACACTGAGAACAGTAAT-------------------CCTTGATTTCCGTATGT-
AAATGAATATAAACGACCAGAATGTAAATATTGCCTGTATC------------------------
TACATTTCATGTTTTATAATTTTATAGGCAGAATCAAGGCTTAGGATAATTGCATTTAGTAG
TGTACTTAAATTCCAAT-GCTAAATGAACCGGT----ATCTACAGTTGCGATCAATAA-
AAAAATAAGTGCATTAAAATAGTCCAAGAACATTAGAATACTAATTTTTGGTGTTTAATTA
AACTATTTTGCGAATTCAAGATCATA---ATT-
GTATATTAATTGGACAGTCTC-TTTTTTT-
ATTACTCCAACTATTCAAATTTTCGTTTTTATTACTAGATAA------
TTTCCTAGTGCTTTTTGTGCATTTCGAACT-ACTAGAATTGTGTTGAAAGA-
AACATGAACTTTAAATTGATACACATTTCAAAGGAGTTTGCCTTTTGCCAATTACCAATA

AAATGTAG-TTTCCGGAAGATCTAAAAACATTTCGTT
>L_ECAM20
CCATGGAAGCCGAGCACCTGGTAGAGCCGCAAGTGGAACTGCAGCACCCGACCCAGAC
GGCCGCTCATTGTTGACTACGCGAAGGCGAAACCCTTTAG-
ATGGACAGGGACAGGCCTTATCCCAGCGGCCGATC-
ATCTGCCTTCAAATGGGCCACCAAACAACTGACAAATCCATTAGCCTAAGTGAATTTGTG
AAAAACACTGAGAACAGTACTCCATGTAGCTT---------CCTTGATTTCCGTATTT-
AAATGAACACAAACCACCAGAAAGTAAAGATTGTCTGTGTC-----------------------
TACATTATATGTTTTATAATTTTATAGGTAGAATCAAGGCTTAGGATAATTGCATTTAGTAG
TGTACTTAAATTCCAAT-GCTAAATGAACCGGT----
ATCTAAAGTTGCAACCAATAACTAAAATAAGTGCATTAAAATGTTCTTGGACCATTAGAA
TATTAAATTTATTAGTT-AAATAAACTAATTTGCGAATTCAAGATCATA--------------------------
-----------------ATTTGTATATTAAGTGGACAGTCTC-TTTTTTT-ATTACTCAAACTATTT------
GCGATTTTATCACTAGATAA------TTTCCAAGTGGGTTTTGTGCGTTTCGAACT-
GCTAGAATTGTGTTGAAAGA-------AACATTGATATTATA----
TTTCAAAGGAGTTTTCCTTTTGCCAATTACCTATAAAA----------------
TTGTAAAAACATTTCGCT
>L_ECAM21
CCATGGAAGCCGAGCACCTGGTAGAGCCGCAAGTGGAACTGCAGCACCCGACCCAGAC
GGCCGCTCATTGTTGACTACGCGAAGGCGAAACCCTTTAC-
ATGGACAGGGACAGGCCTTATCCC-------GATC-
ATCTCCCTTCAAATGGGCCACCAAACAACTGACAAATCCATTAGCCTAAGTGAATTTGTG
AAAAACACTGAGAACAGTA-TCCATGTAGCTT---------CCTTGATTTCCGTATTT-
AAATGAACACAAACCACCAGAAAGTAAAGATTGTCTGTGTC-----------------------
TACATTATATGTTTTATAATTTTATAGGCAGAATCAAGGCTTAGGATAATTGCATTTAGTAG
TATACTTAAATTCCAAT-GCTAAATGAACCGGT----
ATCTAAAGTTGCAACCAATAACAAAAATATGTGCATTAAAATGTTCTTGGACCATTAGAA
TATTAAATTTATTAGTT-AAATAAACTAATTTGCGAATTCCAGATCATA--------------------------
-----------------ATTTGTATATTAAGTGGACAGTCTC-TTTTTTT-ATTACTCAAACTATTT------
GCGATTTTATCACTAGATAA------TTTCCAAGTGGGTTTTGTGCGTTTCGAACT-
GCTAGAATTGTGTTGAAAGA-------AACATTGATATTATA----
TTTCAAAGGAGTTTGCCTTTTGCCAATTACCTATAAAA----------------
TTGTAAAAACATTTCGCT
>D_ECAM21b
NNNNNNNNNNNNNNNNNNNNNNNNNNNNNNNNNNNNNNNNNNNNNNNNNNNNNNNNNNNN
NNNNNNNNNNNNNNNNNNNNNNNNNNNNNNNNNNNNNNNNNNNNNNNNNNNNNNNNNNNN
NNNNNNNNNNNNNNNNNNNNNNNNNNNNNNNNNNNNNNNNNNNNNNNNNNNNNNNNNNNN
NNNNNNNNNNNNNNNNNNNNNNNNNNNNNNNNNNNNNNNNNNNNNNNNNNNNNNNNNNNN
NNNNNNNNNNNNNNNNNNNNNNNNNNNNNNNNNNNN-
NNNNNNNNNNNNNNNNNNNNNNNNNNNNNNNNNNNNNNNNNNNNN-----------------------
NNNNNNNNNNNNNNNNNNNNNNNNNNNNNNNNNNNNNNNNNNNNNNNNNNNNNNNNNNNN
NNNNNNNNNNNNNNNNNNNNNNNNNNNNNNNNNNNNNNNNNNNN----
NNNNNNNNNNNNNNNNNNNNNNNNNNNNNNNNAGTGCATTAAAATAGTCCAAGAACATTA
GAATACTAATTTTTGGTGTTTAATTAAACTATTTTGCGAATTCAAGATCATA-------------------
-------------------------ATT-GTATATTAATTGGACAGTCTC-TTTTTTT-
ATTACTCCAACTATTCAAATTTTCGTTTTTATTACTAGATAA------
TTTCCTAGTGCTTTTTGTGCATTTCGAACT-ACTAGAATTGTGTTGAAAGA-
AACATGAACATTAAATTGATACACATTTCAAAGGAGTTTGCCTTTTGCCAATTACCAATA
AAATGTAG-TTTCCGGAAGATCTAAAAACATTTCGTT
>L_ECAM22b

NNNNNNNNNNNNNNNNNNNNNNNNNNNNNNNNNNNNNNNNNNNNNNNNNNNNNNNNNNN
NNNNNNNNNNNNNNNNNNNNNNNNNNNNNNNNNNNNNNNNNNNNNNNNNNNNNNNNNNN
NNNNNNNNNNNNNNNNNNNNNNNNNNNNNNNNNNNNNNNNNNNNNNNNNNNNNNNNNNN
NNNNNNNNNNNNNNNNNNNNNNNNNNNNNNNNNNNNNNNNNNNNNNNNNNNNNNNNNNN
NNNNNNNNNNNNNNNNNNNNNNNNNNNNNNNNNNN-
NNNNNNNNNNNNNNNNNNNNNNNNNNNNNNNNNNNNNNNNNNNN--------------------------
NNNNNNNNNNNNNNNNNNNNNNNNNNNNNNNNNNNNNNNNNNNNNNNNNNNNNNNNNNN
NNNNNNNNNNNNNNNNNNNNNNNNNNNNNNNNNNNNNNNNNNN----
NNNNNNNNNNNNNNNNNNNNNNNNNNNNNNNAGTGCATTAAAATGTTCTTGGACCATTAG
AATATTAAATTTATTAGTT-AAATAAACTAATTTGCCAAGTCAAGATCATA------------------------
------------------------ATTTGTATATTAAGTGGACAGTCTC-TTTTTTT-ATTACTCAAACTATTT----
--GCGATTTTATCACTAGATAA------TTTCCAAGTGGGTTTTGTGCGTTTCGAACT-
GCTAGAATTGTGTTGAAAGA-------AACATT-----GATA----
TTTCAAAGGAGTTTGCCTTTTGCCAATTACCTATAAAA------------------
GTGTAAAAACATTTCGCT
>D_ECAM22
CCATGGAAGCCGAGCACCTGGTAGAGCCGCAAGTGGAACTGCAGCACCCGACCCAGAC
GGCCGCTCATTGTTGACTACNCGAAGGCGAAACCCTTTAC-
ATGGACAGGGACAGGCCTTATCCCAGCGGCCGATC-
ATCTGCCTTCAAATGGGCCACCAAACAACTGACAAATCCATTAGCCTAAGTGAATTTGTG
AAAAACACTGAGAACAGTAAT----------------------CCTTGATTTCCGTATGT-
AAATGAATATAAACGACCAGAATGTAAATATTGCCTGTATC---------------------------
TACATTTTATGTTTTATAATTTTATAGGCAGAATCAAGGCTTAGGATAATTGCATTTAGTAG
TGTACTTAAATTCCAAT-GCTAAATGAACCGGT----ATCTAAAGTTGCGATCAATAA-
AAAAATAAGTGCATTAAAATAGTCCAAGAACATTAGAATACTAATTTTTGGTGTTTAATTA
ACCTATTTTGCGAATTCAAGATCATA---ATT-
GTATATTAATTGGACAGTCTC-TTTTTTT-
ATTACTCCAACTATTCAAATTTTCGTTTTTATTACTAGATAA------
TTTCCTAGTGCTTTTTGTGCATTTCGAACTGCCTAGAATTGTGTTGAAATAAAACATGAA
CATTAAATTGATACACATTTCAAAGGAGTTTGCCTTTTGCCAATTACCAATAAAATGTAG-
TTTCCGGAAGATCTAAAAACATTTCGGT
>D_ECAM24b
NNNNNNNNNNNNNNNNNNNNNNNNNNNNNNNNNNNNNNNNNNNNNNNNNNNNNNNNNNN
NNNNNNNNNNNNNNNNNNNNNNNNNNNNNNNNNNNNNNNNNNNNNNNNNNNNNNNNNNN
NNNNNNNNNNNNNNNNNNNNNNNNNNNNNNNNNNNNNNNNNNNNNNNNNNNNNNNNNNN
NNNNNNNNNNNNNNNNNNNNNNNNNNNNNNNNNNNNNNNNNNNNNNNNNNNNNNNNNNN
NNNNNNNNNNNNNNNNNNNNNNNNNNNNNNNNNNN-
NNNNNNNNNNNNNNNNNNNNNNNNNNNNNNNNNNNNNNNNNNNN--------------------------
NNNNNNNNNNNNNNNNNNNNNNNNNNNNNNNNNNNNNNNNNNNNNNNNNNNNNNNNNNN
NNNNNNNNNNNNNNNNNNNNNNNNNNNNNNNNNNNNNNNNNNN----
NNNNNNNNNNNNNNNNNNNNNNNNNNNNNNNAGTGCATTAAAATAGTCCAAGAACATTA
GAATACTAATTTTTGGTGTTTAATTAACCTATTTTGCGAATTCAAGATCATA---------------------
----------------------------ATT-GTATATTAATTGGACAGTCTC-TTTTTTT-
ATTACTCCAACTATTCAAATTTTCGTTTTTATTACTAGATAA------
TTTCCTAGTGCTTTTTGTGCATTTCGAACT-GCTAGAATTGTGTTGAAATA-
AACATGAACATTAAATTGATACACATTTCAAAGGAGTTTGCCTTTTGCCAATTACCAATA
AAATGTAG-TTTCCGGAAGATCTAAAAACATTTCGTT
>L_ECAM25
NNNNNNNNNNNNNNNNNNNNNNNNNNNNNNNNNNNNNNNNNNNNNNNNNNNNNNNNNNN
NNNNNNNNNNNNNNNNNNNNNNNNNNNNNNNNNNNNNNNNNNNNNNNNNNNNNNNNNNN

NNNNNNNNNNNNNNNNNNNNNNNNNNNNNNNNNNNNNNNNNNNNNNNNNNNNNNNNNNNN
NNNNNNNNNNNNNNNNNNNNNNNNNNNNNNNNNNNNNNNNNNNNNNNNNNNNNNNNNNNN
NNNNNNNNNNNNNNNNNNNNNNNNNNNNNNNNNNNNN-
NNNNNNNNNNNNNNNNNNNNNNNNNNNNNNNNNNNNNNNNNNNNN--------------------------
NNNNNNNNNNNNNNNNNNNNNNNNNNNNNNNNNNNNNNNNNNNNNNNNNNNNNNNNNNNN
NNNNNNNNNNNNNNNNNNNNNNNNNNNNNNNNNNNNNNNNNNNN----
NNNNNNNNNNNNNNNNNNNNNNNNNNNNNNNAGTGCATTAAAATGTTCTTGGACCATTAG
AATATTAAATTTATTAGTT-AAATAAACTAATTTGCGAATTCAAGATCATA-------------------------
----------------------ATTTGTATATTAAGTGGACAGTCTC-TTTTTTT-ATTACTCAAACTATTT-----
-GCGATTTTATCACTAGATAA------TTTCCAAGTGGGTTTTGTGCGTTTCGAACT-
GCTAGAATTGTGTTGAAAGA-------AACATC-----GATA----
TTTCAAAGGAGGTTGCCTTTTGCCAATTACCTATAAAA-----------------
GTGTTAAAACATTTCGCT
>D_ECAM26
NNNNNNNNNNNNNNNNNNNNNNNNNNNNNNNNNNNNNNNNNNNNNNNNNNNNNNNNNNNN
NNNNNNNNNNNNNNNNNNNNNNNNNNNNNNNNNNNNNNNNNNNNNNNNNNNNNNNNNNNN
NNNNNNNNNNNNNNNNNNNNNNNNNNNNNNNNNNNNNNNNNNNNNNNNNNNNNNNNNNNN
NNNNNNNNNNNNNNNNNNNNNNNNNNNNNNNNNNNNNNNNNNNNNNNNNNNNNNNNNNNN
NNNNNNNNNNNNNNNNNNNNNNNNNNNNNNNNNNNNN-
NNNNNNNNNNNNNNNNNNNNNNNNNNNNNNNNNNNNNNNNNNNNN--------------------------
NNNNNNNNNNNNNNNNNNNNNNNNNNNNNNNNNNNNNNNNNNNNNNNNNNNNNNNNNNNN
NNNNNNNNNNNNNNNNNNNNNNNNNNNNNNNNNNNNNNNNNNNN----
NNNNNNNNNNNNNNNNNNNNNNNNNNNNNNNAGTGCATTAAAATAGTCCAAGAACATTA
GAATACTAATTTTTGGTGTTTAATTAAACTATTTTGCGAATTCAAGATCATA--------------------
---------------------------ATT-GTATATTAATTGGACAGTCTC-TTTTTTT-
ATTACTCCAACTATTCAAATTTTCGTTTTTATTACTAGATAA------
TTTCCTAGTGCTTTTTGTGCATTTCGAACT-ACTAGAATTGTGTTGAAAGA-
AACATGAACATTAAATTGATACACATTTCAAAGGAGTTTGCCTTTTGCCAATTACCAATA
AAATGTAG-TTTCCGGAAGATCTAAAAACATTTCGTT
>D_ECAM27
NNNNNNNNNNNNNNNNNNNNNNNNNNNNNNNNNNNNNNNNNNNNNNNNNNNNNNNNNNNN
NNNNNNNNNNNNNNNNNNNNNNNNNNNNNNNNNNNNNNNNNNNNNNNNNNNNNNNNNNNN
NNNNNNNNNNNNNNNNNNNNNNNNNNNNNNNNNNNNNNNNNNNNNNNNNNNNNNNNNNNN
NNNNNNNNNNNNNNNNNNNNNNNNNNNNNNNNNNNNNNNNNNNNNNNNNNNNNNNNNNNN
NNNNNNNNNNNNNNNNNNNNNNNNNNNNNNNNNNNNN-
NNNNNNNNNNNNNNNNNNNNNNNNNNNNNNNNNNNNNNNNNNNNN--------------------------
NNNNNNNNNNNNNNNNNNNNNNNNNNNNNNNNNNNNNNNNNNNNNNNNNNNNNNNNNNNN
NNNNNNNNNNNNNNNNNNNNNNNNNNNNNNNNNNNNNNNNNNNN----
NNNNNNNNNNNNNNNNNNNNNNNNNNNNNNNAGTGCATTAAAATAGTCCAAGAACATTA
GAATACTAATTTTTGGTGTTTAATTAACCTATTTTGCGAATTCAAGATCATA--------------------
---------------------------ATT-GTATATTAATTGGACAGTCTC-TTTTTTT-
ATTACTCCAACTATTCAAATTTTCGTTTTTATTACTAGATAA------
TTTCCTAGTGCTTTTTGTGCATTTCGAACT-GCTAGAATTGTGTTGAAATA-
AACATGAACATTAAATTGATACACATTTCAAAGGAGTTTGCCTTTTGCCAATTACCAATA
AAATGTAG-TTTCCGGAAGATCTAAAAACATTTCGTT
>L_ECAM28
NNNNNNNNNNNNNNNNNNNNNNNNNNNNNNNNNNNNNNNNNNNNNNNNNNNNNNNNNNNN
NNNNNNNNNNNNNNNNNNNNNNNNNNNNNNNNNNNNNNNNNNNNNNNNNNNNNNNNNNNN
NNNNNNNNNNNNNNNNNNNNNNNNNNNNNNNNNNNNNNNNNNNNNNNNNNNNNNNNNNNN
NNNNNNNNNNNNNNNNNNNNNNNNNNNNNNNNNNNNNNNNNNNNNNNNNNNNNNNNNNNN

NNNNNNNNNNNNNNNNNNNNNNNNNNNNNNNNNNNN-
NNNNNNNNNNNNNNNNNNNNNNNNNNNNNNNNNNNNNNNNNNNNN------------------------
NNNNNNNNNNNNNNNNNNNNNNNNNNNNNNNNNNNNNNNNNNNNNNNNNNNNNNNNNNNN
NNNNNNNNNNNNNNNNNNNNNNNNNNNNNNNNNNNNNNNNNNNN----
NNNNNNNNNNNNNNNNNNNNNNNNNNNNNNNAGTGCATTAAAATGTTCTTGGACCATTAG
AATATTAAATTTATTAGTT-AAATAAACTAATTTGCGAATTCAAGATCATA-----------------------
---------------------ATTTGTATATTAAGTGGACAGTCTC-TTTTTTT-ATTACTCAAACTATTT-----
-GCGATTTTATCACTAGATAA------TTTCCAAGTGGGTTTTGTGCGTTTCGAACT-
GCTAGAATTGTGTTGAAAGA-------AACATT-----GATA----TTTCAAAGGAG-
TTGCTTTTTGCCAATTACCTATAAAA----------------GTGTAAAAACATTTCGCT
>D_ECAM31
CCATGGAAGCCGAGCACCTGGTAGAGCCGCAAGTGGAACTGCAGCACCCGACCCAGAC
GGCCGCTCATTGTTGACTACGCGAAGGCGAAACCCTTTAC-
ATGGACAGGGACAGGCCTTATCCC-------GATC-
ATCTCCCTTCAAATGGGCCACCAAACAACTGACAAATCCATTAGCCTAAGTGAATTTGTG
AAAAACACTGAGAACAGTAAT--------------------CCTTGATTTCCGTATGT-
AAATGAATATAAACGACCAGAATGTAAATATTGCCTGTATC-----------------------
TACATTTCATGTTTTATAATTTTATAGGCAGAATCAAGGCTTAGGATAATTGCATTTAGTAG
TGTACTTAAATTCCAAT-GCTAAATGAACCGGT----ATCTACAGTTGCGATCAATAA-
AAAAATAAGTGCATTAAAATAGTCCAAGAACATTAGAATACTAATTTTTGGTGTTTAATTA
AACTATTTTGCGAATTCAAGATCATA--ATT-
GTATATTAATTGGACAGTCTC-TTTTTTT-
ATTACTCCAACTATTCAAATTTTCGTTTTTATTACTAGATAA------
TTTCCTAGTGCTTTTTGTGCATTTCGAACT-ACTAGAATTGTGTTGAAAGA-
AACATGAACATTAAATTGATACACATTTCAAAGGAGTTTGCCTTTTGCCAATTACCAATA
AAATGTAG-TTTCCGGAAGATCTAAAAACATTTCGTT
>D_ECAM33
NNNNNNNNNNNNNNNNNNNNNNNNNNNNNNNNNNNNNNNNNNNNNNNNNNNNNNNNNNNN
NNNNNNNNNNNNNNNNNNNNNNNNNNNNNNNNNNNNNNNNNNNNNNNNNNNNNNNNNNNN
NNNNNNNNNNNNNNNNNNNNNNNNNNNNNNNNNNNNNNNNNNNNNNNNNNNNNNNNNNNN
NNNNNNNNNNNNNNNNNNNNNNNNNNNNNNNNNNNNNNNNNNNNNNNNNNNNNNNNNNNN
NNNNNNNNNNNNNNNNNNNNNNNNNNNNNNNNNNNN-
NNNNNNNNNNNNNNNNNNNNNNNNNNNNNNNNNNNNNNNNNNNNN------------------------
NNNNNNNNNNNNNNNNNNNNNNNNNNNNNNNNNNNNNNNNNNNNNNNNNNNNNNNNNNNN
NNNNNNNNNNNNNNNNNNTTCCAAT-GCTAAATGAACCGGT----
ATCTACAGTTGCGATCAATAA-
AAAAATAAGTGCATTAAAATAGTCCAAGAACATTAGAATACTAATTTTTGGTGTTTAATTA
AACTATTTTGCGAATTCAAGATCATA--ATT-
GTATATTAATTGGACAGTCTC-TTTTTTT-
ATTACTCCAACTATTCAAATTTTCGTTTTTATTACTAGATAA------
TTTCCTAGTGCTTTTTGTGCATTTCGAACT-ACTAGAATTGTGTTGAAAGA-
AACATGAACATTAAATTGATACACATTTCAAAGGAGTTTGCCTTTTGCCAATTACCAATA
AAATGTAG-TTTCCGGAAGATCTAAAAACATTTCGTT
>L_ECAM34
NNNNNNNNNNNNNNNNNNNNNNNNNNNNNNNNNNNNNNNNNNNNNNNNNNNNNNNNNNNN
NNNNNNNNNNNNNNNNNNNNNNNNNNNNNNNNNNNNNNNNNNNNNNNNNNNNNNNNNNNN
NNNNNNNNNNNNNNNNNNNNNNNNNNNNNNNNNNNNNNNNNNNNNNNNNNNNNNNNNNNN
NNNNNNNNNNNNNNNNNNNNNNNNNNNNNNNNNNNNNNNNNNNNNNNNNNNNNNNNNNNN
NNNNNNNNNNNNNNNNNNNNNNNNNNNNNNNNNNNN-
NNNNNNNNNNNNNNNNNNNNNNNNNNNNNNNNNNNNNNNNNNNNN------------------------

NNNNNNNNNNNNNNNNNNNNNNNNNNNNNNNNNNNNNNNNNNNNNNNNNNNNNN
NNNNNNNNNNNNNNNNNNTTCCAATGGCCTAATGAACCGGT----
ATCTAAAGTTGCAACCAATAACTAAAATAAGTGCATTAAAATGTTCTTGGACCATTAGAA
TATTAAATTTATTAGTT-AAATAAACTAATTTGCCAAGTCAAGATCATA--------------------------
------------------ATTTGTATATTAAGTGGACAGTCTC-TTTTTTT-ATTACTCAAACTATTT------
GCGATTTTATCACTAGATAA------TTTCCAAGTGGGTTTTGTGCGTTTCGAACT-
GCTAGAATTGTGTTGAAAGA-------AACATT-----GATA----
TTTCAAAGGAGTTTGCCTTTTGCCAATTACCTATAAAA----------------
GTGTAAAAACATTTCGCT
>L_ECAM38
CCATGGAAGCCGAGCACCTGGTAGAGCCGCAAGTGGAACTGCAGCACCCGACCCAGAC
GGCCGCTCATTGTTGACTACGCGAAGGCGAAACCCTTTAG-
ATGGACAGGGACAGGCCTTATCCCAGCGGCCGATC-
ATCTGCCTTCAAATGGGCCACCAAACAACTGACAAATCCATTAGCCTAAGTGAATTTGTG
AAAAACACTGAGAACAGTAATCCATGTAGCTT---------CCTTGATTTCCGTATTT-
AAATGAACACAAACCACCAGAAAGTAAAGATTGTCTGTGTC-----------------------
TACATTATATGTTTTATAATTTTATAGGCAGAATCAAGGCTTAGGATAATTGCATTTAGTAG
TATACTTAAATTCCAAT-GCTAAATGAACCGGT----
ATCTAAAGTTGCAACCAATAACAAAAATAAGTGCATTAAAATGTTCTTGGACCATTAGAA
TATTAAATTTATTAGTT-AAATAAACTAATTTGCGAATTCAAGATCATA---------------------------
-----------------ATTTGTATATTAAGTGGACAGTCTC-TTTTTTT-ATTACTCAAACTATTT------
GCGATTTTATCACTAGATAA------TTTCCAAGTGGGTTTTGTGCGTTTCGAACT-
GCTAGAATTGTGTTGAAAGA-------AACATT-----GATA----
TTTCAAAGGAGGTTGCCTTTTGCCAATTACCTATAAAA----------------
GTGTTAAAACATTTCGCT
>L_EGAB_C3a
CCATGGAAGCCGAGCACCTGGTAGAGCCGCAAGTGGAACTGCAGCACCCGACCCAGAC
GGCCGCTCATTGTTGACTACGCGAAGGCGAAACCCTTTAG-
ATGGACAGGGACAGGCCTTATCCCAGCGGCCGATC-
ATCTGCCTTCAAATGGGCCACCAAACAACTGACAAATCCATTAGCCTAAGTGAATTTGTG
AAAAACACTGAGAACAGTACTCCATGTAGCTT---------CCTTGATTTCCGTATTT-
AAATGAACACAAACCACCAGAAAGTAAAGATTGTCTGTGTC-----------------------
TACATTATATGTTTTATAATTTTATAGGCAGAATCAAGGCTTAGGATAATTGCATTTAGTAG
TGTACTTAAATTCCAAT-GCTAAATGAACCGGT----
ATCTAAAGTTGCAACTAATAACTAAAATAAGTGCATTAAAATGTTCTTGGACCATTAGAA
TATTAAATTTATTAGTT-AAATAAACTAATTTGCGAATTCAAGATCATC---------------------------
-----------------ATTTGTATATTAAGTGGACAGTCTC-TTTTTTT-ATTACTCAAACTATTT------
GCGATTTTATCACTAGATAA------TTTCCAAGTGGGTTTTGTGCGTTTCGAACT-
GCTAGAATTGTGTTGAAAGA-------CACATT-----GATA----
TTTCAAAGGAGTTTGCCTTTTGCCAATTACCTATAAAA----------------
GTGTAAAAACATTTCGCT
>L_EGAB_C3b
CCATGGAAGCCGAGCACCTGGTAGAGCCGCAAGTGGAACTGCAGCACCCGACCCAGAC
GGCCGCTCATTGTTGACTACGCGAAGGCGAAACCCTTTAC-
ATGGACAGGGACAGGCCTTATCCC-------GATC-
ATCTCCCTTCAAATGGGCCACCAAACAACTGACAAATCCATTAGCCTAAGTGAATTTGTG
AAAAACACTGAGAACAGTA-TCCATGTAGCTT---------CCTTGATTTCCGTATTT-
AAATGAACACAAACCACCAGAAAGTAAAGATTGTCTGTGTC-----------------------
TACATTATATGTTTTATAATTTTATAGGCAGAATCAAGGCTTAGGATAATTGCATTTAGTAG
TATACTTAAATTCCAAT-GCTAAATGAACCGGT----

ATCTAAAGTTGCAACCAATAACAAAAATATGTGCATTAAAATGTTCTTGGACCATTAGAA
TATTAAATTTATTAGTT-AAATAAACTAATTTGCGAATTCAAGATCATA---------------------------
-----------------ATTTGTATATTAAGTGGACAGTCTC-TTTTTTT-ATTACTCAAACTATTT------
GCGATTTTATCACTAGATAA------TTTCCAAGTGGGTTTTGTGCGTTTCGAACT-
GCTAGAATTGTGTTGAAAGA-------AACATTGATATTATA----
TTTCAAAGGAGTTTGCCTTTTGCCAATTACCTATAAAA----------------
TTGTAAAAACATTTCGCT
>L_EGAB_C136
NNNNNNNNNNNNNNNNNNNNNNNNNNNNNNNNNNNNNNNNNNNNNNNNNNNNNNN
NNNNNNNNNNNNNNNNNNNNNNNNNNNNNNNNNNNNNNNNNNNNNNNNNNNNNNN
NNNNNNNNNNNNNNNNNNNNNNNNNNNNNNNNNNNNNNNNNNNNNNNNNNNNNNN
NNNNNNNNNNNNNNNNNNNNNNNNNNNNNNNNNNNNNNNNNNNNNNNNNNNNNNN
NNNNNNNNNNNNNNNNCCTTGATTTCCGTATTT-
AAATGAACACAAACCACCAGAAAGTAAAGATTGTCTGTGTC-------------------------
TACATTATATGTTTTATAATTTTATAGGCAGAATCAAGGCTTAGGATAATTGCATTTAGTAG
TATACTTAAATTCCAAT-GCTAAATGAACCGGT----
ATCTAAAGTTGCAACCAATAACAAAAATAAGTGCATTAAAATGTTCTTGGACCATTAGAA
TATTAAATTTATTAGTT-AAATAAACTAATTTGCGAATTCAAGATCATC---------------------------
-----------------ATTTGTATATTAAGTGGACAGTCTC-TTTTTTT-ATTACTCAAACTATTT------
GCGATTTTATCACTAGATAA------TTTCCAAGTGGGTTTTGTGCGTTTCGAACT-
GCTAGAATTGTGTTGAAAGA-------CACATT-----GATA----
TTTCAAAGGAGTTTGCCTTTTGCCAATTACCTATAAAA----------------
GTGTAAAAACATTTCGCT
>D_EGAB_N2
NNNNNNNNNNNNNNNNNNNNNNNNNNNNNNNNNNNNNNNNNNNNNNNNNNNNNNN
NNNNNNNNNNNNNNNNNNNNNNNNNNNNNNNNNNNNNNNNNNNNNNNNNNNNNNN
NNNNNNNNNNNNNNNNNNNNNNNNNNNNNNNNNNNNNNNNNNNNNNNNNNNNNNN
NNNNNNNNNNNNNNNNNNNNNNNNNNNNNNNNNNNNNNNNNNNNNNNNNNNNNNN
NNNNNNNNNNNNNNNNCCTTGATTTCCGTATGT-
AAATGAATATAAACGACCAGAATGTAAATATTGCCTGTATC-------------------------
TACATTATATGTTTTATAATTTTATAGGCAGAATCAAGGCTTAGGATAATTGCATTTAGTAG
TGTACTTAAATTCCAAT-GCTAAATGAACCGGT----ATCTACAGTTGCGATCAATAA-
AAAAATAAGTGCATTAAAATAGTCCAAGAACATTAGAATACTAATTTTTGGTGTTTAATTA
AACTATTTTGCGAATTCAAGATCATA---ATT-
GTATATTAATTGGACAGTCTC-TTTTTTT-
ATTACTCCAACTATTCAAATTTTCGTTTTTATTACTAGATAA------
TTTCCTAGTGCTTTTTGTGCATTTCGAACT-ACTAGAATTGTGTTGAAAGA-
AACATGAACATTAAATTGATACACATTTCAAAGGAGTTTGCCTTTTGCCAATTACCAATA
AAATGTAG-TTTCCGGAAGATGTAAAAACATTTCGTT
>L_EGAB6A
CCATGGAAGCCGAGCACCTGGTAGAGCCGCAAGTGGAACTGCAGCACCCGACCCAGAC
GGCCGCTCATTGTTGACTACGCGAAGGCGAAACCCTTTAC-
ATGGACAGGGACAGGCCTTATCCC-------GATC-
ATCTCCCTTCAAATGGGCCACCAAACAACTGACAAATCCATTAGCCTAAGTGAATTTGTG
AAAAACACTGAGAACAGTAATCCATGTAGCTT---------CCTTGATTTCCGTATTT-
AAATGAACATAAACCACCAGAAAGTAAAGATTGTCTGTGTC-------------------------
TACATTATAAGTTTTATAATTTTATAGGTAGAATCAAGGCTTAGGATAATTGCATTTAGTAG
TGTACTTAAATTCCAAT-GCTAAATGAACCGGT----
ATCTAAAGTAGCAACCAATAACAAAAATGAGTGCATTAAAATGTTCTTGGACCATTAGAA
TATTAAATTTATTAGTT-AAATAAACTAATTTGCGAATTCAAGATCATA---------------------------

-----------------ATTTGTATATTAAGTGGACAGTCTC-TTTTTTT-ATTACTCAAACTATTT------
GCGATTTTATCACTAGATAA------TTTCCAAGTGCGTTTTGTGCATTTCGAACT-
GCTAGAATTGTGTTGAAAGA-------AACATT----TGATA----TTTCAAAGGAGTT-
GCCTTTTGCCAATTACCTATAAAA----------------GTGTAAAAACATTTCGTT
>L_EGAB6B
CCATGGAAGCCGAGCACCTGGTAGAGCCGCAAGTGGAACTGCAGCACCCGACCCAGAC
GGCCGCTCATTGTTGACTACGCGAAGGCGAAACCCTTTAC-
ATGGACAGGGACAGGCCTTATCCCAGCGGCCGATC-
ATCTGCCTTCAAATGGGCCACCAAACAACTGACAAATCCATTAGCCTAAGTGAATTTGTG
AAAAACACTGAGAACAGTAATCCATGTAGCTT---------CCTTGATTTCCGTATTT-
AAATGAACACAAACCACCAGAAAGTAAAGATTGTCTGTGTC------------------------
TACATTATATGTTTTATAATTTTATAGGCAGAATCAAGGCTTAGGATAATTGCATTTAGTAG
TGTACTTAAATTCCAAT-GCTAAATGAACCGGT----
ATCTAAAGTTGCAACCAATAACTAAAATAAGTGCATTAAAATGTTCTTGGACCATTAGAA
TATAAAATTTATTAGTT-AAATAAACTAATTTGCGAATTCAAGATCATA--------------------------
------------------ATTTGTATATTAAGTGGACAGTCTC-TTTTTTT-ATTACTCAAACTATTT------
GCGATTTTATCACTAGATAA------TTTCCAAGTGGGTTTTGTGCGTTTCGAACT-
GCTAGAATTGTGTTGAAAGAA------AACATT-----GATA----TTTCAAAGGAGTT-
GCCTTTTGCCAATTACCTATAAAA----------------GTGTAAAAACAATTCGCT
>D_EGAB7
CCATGGAAGCCGAGCACCTGGTAGAGCCGCAAGTGGAACTGCAGCACCCGACCCAGAC
GGCCGCTCATTGTTGACTACGCGAAGGCGAAACCCTTTAC-
ATGGACAGGGACAGGCCTTATCCC-------GATC-
ATCTCCCTTCAAATGGGCCACCAAACAACTGACAAATCCATTAGCCTAAGTGAATTTGTG
AAAAACACTGAGAACAGTAAT--------------------CCTTGATTTCCGTATGT-
AAATGAATATAAACGACCAGAATGTAAATATTGCCTGTATC------------------------
TACATTTCATGTTTTATAATTTTATAGGCAGAATCAAGGCTTAGGATAATTGCATTTAGTAG
TGTACTTAAATTCCAAT-GCTAAATGAACCGGT----ATCTACAGTTGCGATCAATAA-
AAAAATAAGTGCATTAAAATAGTCCAAGAACATTAGAATACTAATTTTTGGTGTTTAATTA
AACTATTTTGCGAATTCAAGATCATA---ATT-
GTATATTAATTGGACAGTCTC-TTTTTTT-
ATTACTCCAACTATTCAAATTTTCGTTTTTATTACTAGATAA------
TTTCCTAGTGCTTTTTGTGCATTTCGAACT-ACTAGAATTGTGTTGAAAGA-
AACATGAACATTAAATTGATACACATTTCAAAGGAGTTTGCCTTTTGCCAATTACCAATA
AAATGTAG-TTTCCGGAAGATCTAAAAACATTTCGTT
>L_EGAB10
NNNNNNNNNNNNNNNNNNNNNNNNNNNNNNNNNNNNNNNNNNNNNNNNNNNNNNNNNNN
NNNNNNNNNNNNNNNNNNNNNNNNNNNNNNNNNNNNNNNNNNNNNNNNNNNNNNNNNNN
NNNNNNNNNNNNNNNNNNNNNNNNNNNNNNNNNNNNNNNNNNNNNNNNNNNNNNNNNNN
NNNNNNNNNNNNNNNNNNNNNNNNNNNNNNNNNNNNNNNNNNNNNNNNNNNNNNNNNNN
NNNNNNNNNNNNNNNNNNNNNNNNNNNNNNNNNNNNN-
NNNNNNNNNNNNNNNNNNNNNNNNNNNNNNNNNNNNNNNNNNNNNNN------------------------
NNNNNNNNNNNNNNNNNNNNNNNNNNNNNNNNNNNNNNNNNNNNNNNNNNNNNNNNNNN
NNNNNNNNNNNNNNNNNNNNNNNNNNNNNNNNNNNNNNNNNNNNNNNN----
NNNNNNNNNNNNNNNNNNNNNNNNNNNNNNNNNAGTGCATTAAAATGTTCTTGGACCATTAG
AATATTAAATTTATTAGCTTAAATAAACTAATTTGCCAAGTCAAGATCATA--------------------
----------------------ATTTGTATATTAAGTGGACAGTCTC-TTTTTTT-ATTACTCAAACTATTT---
---GCGATTTTATCACTAGATAA------TTTCCAAGTGGGTTTTGTGCGTTTCGAACT-
GCTAGAATTGTGTTGATAGA-------AACATT-----GATA----
TTTCAAAGGAGTTTGCCTTTTGCCAATTACCTATAAAA----------------

GTGTAAAAACATTTCGCT
>L_EGAB150A
NNNNNNNNNNNNNNNNNNNNNNNNNNNNNNNNNNNNNNNNNNNNNNNNNNNNNNN
NNNNNNNNNNNNNNNNNNNNNNNNNNNNNNNNNNNNNNNNNNNNNNNNNNNNNNN
NNNNNNNNNNNNNNNNNNNNNNNNNNNNNNNNNNNNNNNNNNNNNNNNNNNNNNN
NNNNNNNNNNNNNNNNNNNNNNNNNNNNNNNNNNNNNNNNNNNNNNNNNNNNNNN
NNNNNNNNNNNNNNNNNNNNNNNNNNNNNNNNN-
NNNNNNNNNNNNNNNNNNNNNNNNNNNNNNNNNNNNNNNNN------------------------
NNNNNNNNNNNNNNNNNNNNNNNNNNNNNNNNNNNNNNNNNNNNNNNNNNNNNNN
NNNNNNNNNNNNNNNNNNNNNNNNNNNNNNNNNNNNNNNN----
NNNNNNNNNNNNNNNNNNNNNNNNNNNNNAGTGCATTAAAATGTTCTTGGACCATTAG
AATATTAAATTTATTAGTTTAAATAAACTAATTTGCCAAGTCAAGATCATA---------------------
-----------------------ATTTGTATATTAAGTGGACAGTCTC-TTTTTTT-ATTACTCAAACTATTT---
---GCGATTTTATCACTAGATAA------TTTCCAAGTGGGTTTTGTGCGTTTCGAACT-
GCTAGAATTGTGTTGAAAGA-------AACATT-----GATA----
TTTCAAAGGAGTTTGCCTTTTGCCAATTACCTATAAAA----------------
GTGTTAAAACATTTCGCT
>L_EGAB150B
NNNNNNNNNNNNNNNNNNNNNNNNNNNNNNNNNNNNNNNNNNNNNNNNNNNNNNN
NNNNNNNNNNNNNNNNNNNNNNNNNNNNNNNNNNNNNNNNNNNNNNNNNNNNNNN
NNNNNNNNNNNNNNNNNNNNNNNNNNNNNNNNNNNNNNNNNNNNNNNNNNNNNNN
NNNNNNNNNNNNNNNNNNNNNNNNNNNNNNNNNNNNNNNNNNNNNNNNNNNNNNN
NNNNNNNNNNNNNNNNNNNNNNNNNNNNNNNNN-
NNNNNNNNNNNNNNNNNNNNNNNNNNNNNNNNNNNNNNNNN------------------------
NNNNNNNNNNNNNNNNNNNNNNNNNNNNNNNNNNNNNNNNNNNNNNNNNNNNNNN
NNNNNNNNNNNNNNNNNNNNNNNNNNNNNNNNNNNNNNNN----
NNNNNNNNNNNNNNNNNNNNNNNNNNNNNAGTGCATTAAAATGTTCTTGGACCATTAG
AATATTAAATTTATTAGTT-AAATAAACTAATTTGCCAAGTCAAGATCATA----------------------
----------------------ATTTGTATATTAAGTGGACAGTCTC-TTTTTTT-ATTACTCAAACTATTT----
--GCGATTTTATCACTAGATAA------TTTCCAAGTGGGTTTTGTGCGTTTCGAACT-
GCTAGAATTGTGTTGAAAGA-------AACATT-----GATA----
TTTCAAAGGAGTTTGCCTTTTGCCAATTACCTATAAAA----------------
GTGTTAAAACATTTCGCT
>L_EGAB152A
NNNNNNNNNNNNNNNNNNNNNNNNNNNNNNNNNNNNNNNNNNNNNNNNNNNNNNN
NNNNNNNNNNNNNNNNNNNNNNNNNNNNNNNNNNNNNNNNNNNNNNNNNNNNNNN
NNNNNNNNNNNNNNNNNNNNNNNNNNNNNNNNNNNNNNNNNNNNNNNNNNNNNNN
NNNNNNNNNNNNNNNNNNNNNNNNNNNNNNNNNNNNNNNNNNNNNNNNNNNNNNN
NNNNNNNNNNNNNNNNNNNNNNNNNNNNNNNNN-
NNNNNNNNNNNNNNNNNNNNNNNNNNNNNNNNNNNNNNNNN------------------------
NNNNNNNNNNNNNNNNNNNNNNNNNNNNNNNNNNNNNNNNNNNNNNNNNNNNNNN
NNNNNNNNNNNNNNNNNNNNNNNNNNNNNNNNNNNNNNNN----
NNNNNNNNNNNNNNNNNNNNNNNNNNNNNTGTGCATTAAAATGTTCTTGGACCATTAG
AATATTAAATTTATTAGTT-AAATAAACTAATTTGCGAATTCAAGATCATA-----------------------
---------------------ATTTGTATATTAAGTGGACAGTCTC-TTTTTTT-ATTACTCAAACTATTT-----
-GCGATTTTATCACTAGATAA------TTTCCAAGTGGGTTTTGTGCGTTTCGAACT-
GCTAGAATTGTGTTGAAAGA-------AACATTGATATTATA----
TTTCAAAGGAGTTTGCCTTTTGCCAATTACCTATAAAA----------------
TTGTAAAAACATTTCGCT
>L_EGAB152B

NNNNNNNNNNNNNNNNNNNNNNNNNNNNNNNNNNNNNNNNNNNNNNNNNNNNNNNNNNNN
NNNNNNNNNNNNNNNNNNNNNNNNNNNNNNNNNNNNNNNNNNNNNNNNNNNNNNNNNNNN
NNNNNNNNNNNNNNNNNNNNNNNNNNNNNNNNNNNNNNNNNNNNNNNNNNNNNNNNNNNN
NNNNNNNNNNNNNNNNNNNNNNNNNNNNNNNNNNNNNNNNNNNNNNNNNNNNNNNNNNNN
NNNNNNNNNNNNNNNNNNNNNNNNNNNNNNNNNNNNN-
NNNNNNNNNNNNNNNNNNNNNNNNNNNNNNNNNNNNNNNNNNNNN------------------------
NNNNNNNNNNNNNNNNNNNNNNNNNNNNNNNNNNNNNNNNNNNNNNNNNNNNNNNNNNNN
NNNNNNNNNNNNNNNNNNNNNNNNNNNNNNNNNNNNNNNNNNNN----
NNNNNNNNNNNNNNNNNNNNNNNNNNNNNNNNNAGTGCATTAAAATGTTCTTGGACCATTAG
AATATTAAATTTATTAGTT-AAATAAACTAATTTGCCAAGTCAAGATCATA----------------------
----------------------ATTTGTATATTAAGTGGACAGTCTC-TTTTTTT-ATTACTCAAACTATTT----
--GCGATTTTATCACTAGATAA------TTTCCAAGTGGGTTTTGTGCGTTTCGAACT-
GCTAGAATTGTGTTGAAAGA-------AACATT-----GATA----
TTTCAAAGGAGTTTGCCTTTTGCCAATTACCTATAAAA----------------
GTGTAAAAACATTTCGCT
>D_EGAB153A
NNNNNNNNNNNNNNNNNNNNNNNNNNNNNNNNNNNNNNNNNNNNNNNNNNNNNNNNNNNN
NNNNNNNNNNNNNNNNNNNNNNNNNNNNNNNNNNNNNNNNNNNNNNNNNNNNNNNNNNNN
NNNNNNNNNNNNNNNNNNNNNNNNNNNNNNNNNNNNNNNNNNNNNNNNNNNNNNNNNNNN
NNNNNNNNNNNNNNNNNNNNNNNNNNNNNNNNNNNNNNNNNNNNNNNNNNNNNNNNNNNN
NNNNNNNNNNNNNNNNNNNNNNNNNNNNNNNNNNNNN-
NNNNNNNNNNNNNNNNNNNNNNNNNNNNNNNNNNNNNNNNNNNNN------------------------
NNNNNNNNNNNNNNNNNNNNNNNNNNNNNNNNNNNNNNNNNNNNNNNNNNNNNNNNNNNN
NNNNNNNNNNNNNNNNNNNNNNNNNNNNNNNNNNNNNNNNNNNN----
NNNNNNNNNNNNNNNNNNNNNNNNNNNNNNNNNAGTGCATTAAAATAGTCCAAGAACATTA
GAATACTAATTTTTGGTGTTTAATTAACCTATTTTGCTAATTCAAGATCATA--------------------
------------------------ATT-GTATATTAATTGGACAGTCTC-TTTTTTT-
ATTACTCCAACTATTCAAATTTTCGTTTTTATTACTAGATAA------
TTTCCTAGTGCTTTTTGTGCATTTCGAACT-GCTAGAATTGTGTTGAAATA-
AACATGAACATTAAATTGATACACATTTCAAAGGAGTTTGCCTTTTGCCAATTACCAATA
AAATGTAG-TTTCCGGAAGATCTAAAAACATTTCGTT
>D_EGAB153B
NNNNNNNNNNNNNNNNNNNNNNNNNNNNNNNNNNNNNNNNNNNNNNNNNNNNNNNNNNNN
NNNNNNNNNNNNNNNNNNNNNNNNNNNNNNNNNNNNNNNNNNNNNNNNNNNNNNNNNNNN
NNNNNNNNNNNNNNNNNNNNNNNNNNNNNNNNNNNNNNNNNNNNNNNNNNNNNNNNNNNN
NNNNNNNNNNNNNNNNNNNNNNNNNNNNNNNNNNNNNNNNNNNNNNNNNNNNNNNNNNNN
NNNNNNNNNNNNNNNNNNNNNNNNNNNNNNNNNNNNN-
NNNNNNNNNNNNNNNNNNNNNNNNNNNNNNNNNNNNNNNNNNNNN------------------------
NNNNNNNNNNNNNNNNNNNNNNNNNNNNNNNNNNNNNNNNNNNNNNNNNNNNNNNNNNNN
NNNNNNNNNNNNNNNNNNNNNNNNNNNNNNNNNNNNNNNNNNNN----
NNNNNNNNNNNNNNNNNNNNNNNNNNNNNNNNNAGTGCATTAAAATAGTCCAAGAACATTA
GAATACTAATTTTTGGTGTTTAATTAACCTATTTTGCTAATTCAAGATCATA--------------------
------------------------ATT-GTATATTAATTGGACAGTCTC-TTTTTTT-
ATTACTCCAACTATTCAAATTTTCGTTTTTATTACTAGATAA------
TTTCCTAGTGCTTTTTGTGCATTTCGAACT-GCTAGAATTGTGTTGAAATA-
AACATGAACATTAAATTGATACACATTTCAAAGGAGTTTGCCTTTTGCCAATTACCAATA
AAATGTAG-TTTCCGGAAGATCTAAAAACATTTCGTT
>D_EGAB154B
NNNNNNNNNNNNNNNNNNNNNNNNNNNNNNNNNNNNNNNNNNNNNNNNNNNNNNNNNNNN
NNNNNNNNNNNNNNNNNNNNNNNNNNNNNNNNNNNNNNNNNNNNNNNNNNNNNNNNNNNN

NNNNNNNNNNNNNNNNNNNNNNNNNNNNNNNNNNNNNNNNNNNNNNNNNNNNNNN
NNNNNNNNNNNNNNNNNNNNNNNNNNNNNNNNNNNNNNNNNNNNNNNNNNNNNNN
NNNNNNNNNNNNNNNNNNNNNNNNNNNNNNNNN-
NNNNNNNNNNNNNNNNNNNNNNNNNNNNNNNNNNNNNNNNN------------------------
NNNNNNNNNNNNNNNNNNNNNNNNNNNNNNNNNNNNNNNNNNNNNNNNNNNNNNN
NNNNNNNNNNNNNNNNNNNNNNNNNNNNNNNNNNNNNNNN----
NNNNNNNNNNNNNNNNNNNNNNNNNNNNNAGTGCATTAAA-
TAGTCCAAGAACATTAGAATACTAATTTTTGGTGTTTAATTAACCTATTTTGCGAAGTCAA
GATCATA--ATT-GTATATTAATTGGACAGTCTC-TTTTTTT-
ATTACTCCAACTATTCAAATTTTCGTTTTTATTACTAGATAA------
TTTCCTAGTGCTTTTTGTGCATTTCGAACT-GCTAGAATTGTGTTGAAATA-
AACATGAACATTAAATTGATACACATTTCAAAGGAGTTTGCCTTTTGCCAATTACCAATA
AAATGTAG-TTTCCGGAAGATCTAAAAACATTTCGTT
>D_EGAB154A
NNNNNNNNNNNNNNNNNNNNNNNNNNNNNNNNNNNNNNNNNNNNNNNNNNNNNNN
NNNNNNNNNNNNNNNNNNNNNNNNNNNNNNNNNNNNNNNNNNNNNNNNNNNNNNN
NNNNNNNNNNNNNNNNNNNNNNNNNNNNNNNNNNNNNNNNNNNNNNNNNNNNNNN
NNNNNNNNNNNNNNNNNNNNNNNNNNNNNNNNNNNNNNNNNNNNNNNNNNNNNNN
NNNNNNNNNNNNNNNNNNNNNNNNNNNNNNNNN-
NNNNNNNNNNNNNNNNNNNNNNNNNNNNNNNNNNNNNNNNN------------------------
NNNNNNNNNNNNNNNNNNNNNNNNNNNNNNNNNNNNNNNNNNNNNNNNNNNNNNN
NNNNNNNNNNNNNNNNNNNNNNNNNNNNNNNNNNNNNNNN----
NNNNNNNNNNNNNNNNNNNNNNNNNNNNNAGTGCATTAAAATAGTCCAAGAACATTA
GAATACTAATTTTTGGTGTTTAATTAACCTATTTTGCGAAGTCAAGATCATA-------------------
-------------------------ATT-GTATATTAATTGGACAGTCTC-TTTTTTT-
ATTACTCCAACTATTCAAATTTTCGTTTTTATTACTAGATAA------
TTTCCTAGTGCTTTTTGTGCATTTCGAACT-GCTAGAATTGTGTTGAAATA-
AACATGAACATTAAATTGATACACATTTCAAAGGAGTTTGCCTTTTGCCAATTACCAATA
AAATGTAG-TTTCCGGAAGATCTAAAAACATTTCGTT

Supplementary Note 2 | Alignment of locus 5 kb upstream of the t_MSE region. Dore= D. orena, D_ = dark haplogroup, L_= light haplogroup, ECAM = erecta from Cameroon, EGAB = erecta from Gabon.

>Dore
TATTGATTTAACATCCCC--ATCGGGAGCCC-ACATATTT-CTGTATTT-
TCCCCGAATTTTCCGCTCCTACTCTTCAGCGACGTCTCGTGGCACTTTTGATCCACAATT
CCGGGCGAAACGTTCCGGCGCACAGTTCCGCGCTACGTGCGATCCAAAATGCGGCAATC
GATTTGCCGCGACCGCCGTCACAGTTCTTATTATTGACACCTCACCTTGATAAAAACTCC
AAAGAAATTTATTTTTTTGCTAGGGGAAAAAAGTTGAGCTATCGGAGACAATCGGTCTTC
GATATCAGCATGCAGTATTTGTACGTATCGGGACATATCGAAATGAAAATACCACACCAG
CTT--------CTAAATCTGGCTGTTTTACTC--AAAATAACGGGTAGCGGGAATT--------
CAAACACATATCTAACAGTCTAACATCAAATTACATTAGCCATTTTAGGTCTTGAAAATAT
CTCATGAATTTAATGCTTTATTCGGTTGTGATAATGTTTATTTCAAAATTGTGCAAACGAG
TTTGTCTCATAACGCAATGCAATTTTAAATTTCACGCATAGTTTTCGCTTGTTTTAATGTTC
AAATTTTGAAATTCTACAGA-TAGGTGCAGAAAGAAAAAATATT------
TGTGGTCGACCATGTAGGCCTCCTTACTCCATGTAGCCACTCTGGGATTCGGAGGTACCG
AGGTTCCTCCTTGAGTACAGTCCCTGCCGCGGCTTAAACTTGCCATCGCCCAGGGTGAT
CCTGGCCGGGAGTTTATTGCCCCTAAATGTGGCTGTTTCGAAGGAGGGCGGTGGCGACA
CCTT
>D_ECAM13
TATTGATTTAACATCCCC--
GTCGGGAGCCCCACATATTTTCTGTATTTTCTTCCGAGTTTTCCGCTCCTATTCTTCGGCG
ACGTCTCGTGGCACTTTCGGTCCACAATTCCGGGCGAAACGATCCGGCGCACAGTTCCG
CGCTACGTGCGATCCAAAATGCGGCAATCGATTTGCCGCGACCGCCGTCACAGTTCTTTT
TATTGACACCTCACCTTGATAAAAACTCCAAAGAAATTTATTTTTCTGCTAGGGCAAAAA
AGTTAGGCTATCGGAAACAATCGGTCTTCGATATCAGCATGCATTATTTGTTCGTATCGGG
ACATATCGATATGAAAATACCACACGAGCTTATTCCCCGCTAAATCTGGCTTTTTTACTCA
TAAAACAACGGGTAACGGTAATTTGATTTTGAAAACACATATCTAACAGTCTAAAATCAA
ATTACATTAGCCATTTTAGAGCTTTAAAATATCTGATGAATTTAATACTTTATTCGGTTGTG
ATAATGTTTATTTGAAAAGTGTGCAAACAAGTTTGTCTCATAACAGAAAGAAATTTTAAA
TTTTGCGCATATTTTTCGCTTGTTTTAATGTTCAAATTTGAAAATTCTACAGACTAGGTGC
AGAAAGAAAAAATATTTGCTTGTGTGGTCGACCATGTGGGCCTCCTCACTCCATGTAGC
CACTCTGGGAGTCGGAGGTACCGAGGTTCCTCCTTGAGTACAGTCCTTGGCGCGGCTTA
AACTTGCCATCGCCCAAGGTGATCCTGGCCGGGAGTTTCTTGCCCCTAAATGTGGCAGC
TTCGAAGGAGGGCGGTGGCGACACCTT
>L_ECAM15
TATTGATTTAACATCCCC--GTCGGGAGCCCCACATATTT-
CTGTATTTTCCCCCGAGTTTTCCGCTCCCTTTCTTCGGCGACGTCTCGTGGCACTTTCGGT
CCACAATTCCGGGCGAAACGTTCCGGCGCACAGTTCCGCGCTACGTGCGATCCAAAATG
CGGCAATCGATTTGCCGCGACCGCCGTCACAGTTCTTTTTATTGACACCTCACCTTGATA
AAAACTCCAAAGAAATTTATTTTTCTGCTAGGGCAAAAAAGTTAGGCTATCGGAAACAA
TCGGTCTTCGATATCAGCATGCATTATTTGTTCGTATCGGGACATATCGATATGAAAATACC
ACACGAGCTTATTCCCCGCTAAATCTGGCTTTTTTACTCCTAAAACAACGGGTAACGATA
ATTTGATTTTGAAAACACATATCTAACAGTCTAAAATCAAATTACATTAGCCATTTTAGAG
CTTTAAAATATCTGATGAATTTAATACTTTATTGGGTTGTGATAATGTTTATTTGAAAAGTG
TGCAAACAAGTTTGTCTCATAACAGAAAGAAATTTTACATTTTGCGCATATTTTTCGCTTG
TTTTAATGTTCAAATTTGAAAATTCTACAGACTAGGTGCAAAAAGAAAAAATATTTGCTT

GTGTGGTCGACCATGTGGACCTCCTCACTCCATGTAGCCACTCTGGGAGTCGGAGGTAC
CGAGGTTCCTCCTTGAGTACAGTCCTTGGCGCGGCTTAAACTTGCCATCGCCCAAGGTG
ATCCTGGCCGGGAGTTTCTTGCCCCTAAATGTGGCTGCTTCGAAGGAGGGCGGTGGCGA
CACCTT
>L_ECAM16
TATTGATTTAACATCCCC--
GTCGGGAGCCCCACATATTTTCTGTATTTTCCCCGAGTTTTCCGCTCCTATTCTTCGGCG
ACGTCTCGTGGCACTTTCGGTCCACAATTCCGGGCGAAACGTTCCGGCGCACAGTTCCG
CGCTACGTGCGATCCAAAATGCGGCAATCGATTTGCCGCGACCGCCGTCACAGTTCTTTT
TATTGACACCTCACCTTGATAAAAACTCCAAAGAAATTTATTTTTCTGCTAGGGCAAAAA
AGTTAGGCTATCGGAGACAATCGGTCTTCGATATCAGCATGCATTATTTGTTCGTATCGGG
ACATATCGATATGAAAATACCACACGAGCTTATTCCCCGCTAAATCTGGCTTTTTTACTCA
TAAAACAACGGGTAACGGTAATTTGATTTTGAAAACACATATCTAACAGTCTAAAATCAA
ATTACATTAGCCATTTTAGAGCTTTAAAATATCTGATGAATTTAATACTTTATTGGGTTGTG
ATAATGTTTATTTGAAAAGTGTGCAAACAAGTTTGTCTCATAACAGAAAGAAATTTTACA
TTTGGCGCATATTTTTCGCTTGTTTTAATGTTCAAATTTGAAAATTCTACAGACTAGGTGC
AGAAAGAAAAAATATTTGCTTGTGTGGTCGACCATGTGGGCCTCCTCACTCCATGTAGC
CACTCTGGGAGTCGGAGGTACCGAGGTTCCTCCTTGAGTACAGTCCTTGGCGCGGCTTA
AACTTGCCATCGCCCAAGGTGATCCTGGCCGGGAGTTTCTTGCCCCTAAATGTGGCTGCT
TCGAAGGAGGGCGGTGGCGACACCTT
>L_ECAM20
TATTGATTTAACATCCCC--GTCGGGAGCCCCACATATTTTCTGTATTT-
TCTTCGAGTTTTCCGCTCCTATTCTTCGGCGACGTCTCGTGGCACTTTCGGTCCACAATT
CCGGGCGAAACGATCCGGCGCACAGTTCCGCGCTACGTGCGATCCAAAATGCGGCAATC
GATTTGCCGCGACCGCCGTCACAGTTCTTTTTATTGACACCTCACCTTGATAAAAACTCC
AAAGAAATTTATTTTTCTGCTAGGGCAAAAAAGTTAGGCTATCGGAGACAATCGGTCTTC
GATATCAGCATGCATTATTTGTTCGTATCGGGACATATCGATATGAAAATACCACACGAGC
TTATTCCCCGCTAAATCTGGCTTTTTTACTCATAAAACAACGGGTAACGGTAATTTGATTT
TGAAAACACATATCTAACAGTCTAAAATCAAATTACATTAGCCATTTTAGAGCTTTAAAAT
ATCTGATGAATTTAATACTTTATTGGGTTGTGATAATGTTTATTTGAAAAGTGTGCAAACA
AGTTTGTCTCATAACAGAAAGAAATTTTACATTTTGCGCATATTTTTCGCTTGTTTTAATG
TTCAAATTTGAAAATTCTACAGACTAGGTGCAGAAAGAAAAAATATTTGCTTGTGTGGTC
GACCATGTGGGCCTCCTCACTCCATGTAGCCACTCTGGGAGTCGGAGGTACCGAGGTTC
CTCCTTGAGTACAGTCCTTGGCGCGGCTTAAACTTGCCATCGCCCAAGGTGATCCTGGC
CGGGAGTTTCTTGCCCCTAAATGTGGCTGCTTCGAAGGAGGGCGGTGGCGACACCTT
>D_ECAM21b
TATTGATTTAACATCCCC--GTCGGGAGCCC-ACATATTT-CTGTATTT-
TCCCCGAGTTTTCCGCTCCTATTCTTCGGCGACGTCTCGTGGCACTTTCGGTCCACAATT
CCGGGCGAAACGATCCGGCGCACAGTTCCGCGCTACGTGCGATCCAAAATGCGGCAATC
GATTTGCCGCGACCGCCGTCACAGTTCTTTTTATTGACACCTCACCTTGATAAAAACTCC
AAAGAAATTTATTTTTCTGCTAGGGCAAAAAAGTTAGGCTATCGGAGACAATCGGTCTTC
GATATCAGCATGCATTATTTGTTCGTATCGGGACATATCGATATGAAAATACCACACGAGC
TTATTCCCCGCTAAATCTGGCTTTTTTACTCATAAAACAACGGGTAACGGTAATTTGATTT
TGAAAACACATATCTAACAGTCTAAAATCAAATTACATTAGCCATTTTAGAGCTTTAAAAT
ATCTGATGAATTTAATACTTTATTGGGTTGTGATAATGTTTATTTGAAAAGTGTGCAAACA
AGTTTGTCTCATAACAGAAAGAAATTTTACATTTTGCGCATATTTTTCGCTTGTTTTAATG
TTCAAATTTGAAAATTCTACAGACTAGGTGCAAAAAGAAAAAATATTTGCTTGTGTGGTC
GACCATGTGGGCCTCCTCACTCCATGTAGCCACTCTGGGAGTCGGAGGTACCGAGGTTC
CTCCTTGAGTACAGTCCTTGGCGCGGCTTAAACTTGCCATCGCCCAAGGTGATCCTGGC
CGGGAGTTTCTTGCCCCTAAATGTGGCTGCTTCGAAGGAGGGCGGTGGCGACACCTT

>D_ECAM22
TATTGATTTAACATCCCC--GTCGGGAGCCC-ACATATTT-CTGTATTT-
TCCCCGAGTTTTCCGCTCCTATTCTTCGGCGACGTCTCGTGGCACTTTTGGTCCACAATT
CCGGGCGAAACGTTCCGGCGCACAGTTCCGCGCTACGTGCGATCCAAAATGCGGCAATC
GATTTGCCGCGACCGCCGTCACAGTTCTTTTTATTGACACCTCACCTTGATAAAAACTCC
AATGAAATTTATTTTTCTGCTAGGGCAAAAAAGTTAGGCTATCGGAGACAATCGGTCTTC
GATATCAGCATGCATTATTTGTTCGTATCGGGACATATCGATATGAAAATACCACACGAGC
TTATTCCCCGCTAAATCTGGCTTTTTTACTCATAAAACAACGGGTAACGGTAATTTGATTT
TGAAAACACATATCTAACAGTCTAAAATCAAATTACATTAGCCATTTTAGAGCTTTAAAAT
ATCTGATGAATTTAATACTTTATTCGGTTGTGATAATGTTTATTTGAAAAGTGTGCAAACA
AGTTTGTCTCATAACAGAAAGAAATTTTAAATTTTGCGCATATTTTTCGCTTGTTTTAATG
TTCAAATTTGAAAATTCTACAGACTAGGTGCAGAAAGAAAAAAATATTTGCTTGTGTGGTC
GACCATGTGGGCCTCCTCACTCCATGTAGCCACTCTGGGAGTCGGAGGTACCGAGGTTC
CTCCTTGAGTACAGTCCTTGGCGCGGCTTAAACTTGCCATCGCCCAAGGTGATCCTGGC
CGGGAGTTTCTTGCCCCTAAATGTGGCAGCTTCGAAGGAGGGCGGTGGCGACACCTT
>ECAM24
TATTGATTTAACATCCCC--GTCGGGAGCCCCACATATTT-CTGTATTT-
TCCCCGAGTTTTCCGCTCCTATTCTTCGGCGACGTCTCGTGGCACTTTCGGTCCACAATT
CCGGGCGAAACGATCCGGCGCACAGTTCCGCGCTACGTGCGATCCAAAATGCGGCAATC
GATTTGCCGCGACCGCCGTCACAGTTCTTTTTATTGACACCTCACCTTGATAAAAACTCC
AAAGAAATTTATTTTTCTGCTAGGGCAAAAAAGTTAGGCTATCGGAGACAATCGGTCTTC
GATATCAGCATGCATTATTTGTTCGTATCGGGACATATCGATATGAAAATACCACACGAGC
TTATTCCCCGCTAAATCTGGCTTTTTTACTCATAAAACAACGGGTAACGGTAATTTGATTT
TGAAAACACATATCTAACAGTCTAAAATCAACTTACATTAGCCATTTTAGAGCTTTAAAAT
ATCTGATGAATTTAATACTTTATTCGGTTGTGATAATGTTTATTTGAAAAGTGTGCAAACA
AGTTTGTCTCATAACAGAAAGAAATTTTACATTTTGCGCATATTTTTCGCTTGTTTTAATG
TTCAAATTTGAAAATTCTACAGACTAGGTGCAAAAAGAAAAAAATATTTGCTTGTGTGGTC
GACCATGTGGGCCTCCTCACTCCATGTAGCCACTCTGGGAGTCGGAGGTACCGAGGTTC
CTCCTTGAGTACAGTCCTTGGCGCGGCTTAAACTTGCCATCGCCCAAGGTGATCCTGGC
CGGGAGTTTCTTGCCCCTAAATGTGGCTGCTTCGAAGGAGGGCGGTGGCGACACCTT
>L_ECAM26
TATTGATTTAACATCCCC--GTCGGGAGCCC-ACATATTT-CTGTATTT-
TCTTCGAGTTTTCGGCTCCTATTCTTCGGCGACGTCTCGTGGCACTTTCGGACCACAATT
CCGGGCGAAACGATCCGGCGCACAGTTCCGCGCTACGTGCGATCCAAAATGCGGCAATC
GATTTGCCGCGACCGCCGTCACAGTTCTTTTTATTGACACCTCACCTTGATAAAAACTCC
AAAGAAATTTATTTTTCTGCTAGGGCAAAAAAGTTAGGCTATCGGAGACAATCGGTCTTC
GATATCAGCATGCATTATTTGTTCGTATCGGGACATATCGATATGAAAATACCACACGAGC
TTATTCCCCGCTAAATCTGGCTTTTTTACTCATAAAACAACGGGTAACGGTAATTTGATTT
TGAAAACACATATCTAACAGTCTAAAATCAAATTACATTAGCCATTTTAGAGCTTTAAAAT
ATCTGATGAATTTAATACTTTATTCGGTTGTGATAATGTTTATTTGAAAAGTGTGCAAACA
AGTTTGTCTCATAACAGAAAGAAATTTTAAATTTTGCGCATATTTTTCGCTTGTTTTAATG
TTCAAATTTGAAAATTCTACAGACTAGGTGCAGAAAGAAAAAAATATTTGCTTGTGTGGTC
GACCATGTGGGCCTCCTCACTCCATGTAGCCACTCTGGGAGTCGGAGGTACCGAGGTTC
CTCCTTGAGTACAGTCCTTGGCGCGGCTTAAACTTGCCATCGCCCCAGGTGATCCTGGCC
GGGAGTTTCTTGCCCCTAAATGTGGCTGCTTCGAAGGAGGGCGGTGGCGACACCTT
>D_ECAM27
TATTGATTTAACATCCCC--GTCGGGAGCCC-ACATATTT-CTGTATTT-
TCTTCGAGTTTTCCCATCCTATTCTTCGGCGACGTCTCGTGGCACTTTCGGTCCACAACT
CCGGGCGAAACGATCCGGCGCACAGTTCCGCGCTACGTGCGATCCAAAATGCGGCAATC
GATTTGCCGCGACCGCCGTCACAGTTCTTTTTATTGACACCTCACCTTGATAAAAACTCC

AAAGAAATTTATTTTTCTGCTAGGGCAAAAAAGTTAGGCTATCGGAGACAATCGGTCTTC
GATATCAGCATGCATTATTTGTTCGTATCGGGACATATCGATATGAAAATACCACACGAGC
TTATTCCCCGCTAAATCTGGCTTTTTTACTCATAAAACAACGGGTAACGGTAATTTGATTT
TGAAAACACATATCTAACAGTCTAAAATCAAATTACATTAGCCATTTTAGAGCTTTAAAAT
ATCTGATGAATTTAATACTTTATTGGGTTGTGATAATGTTTATTTGAAAAGTGTGCAAACA
AGTTTGTCTCATAACAGAAAGAAATTTTACATTTTGCGCATATTTTTCGCTTGTTTTAATG
TTCAAATTTGAAAATTCTACAGACTAGGTGCAAAAAGAAAAAATATTTGCTTGTGTGGTC
GACCATGTGGGCCTCCTCACTCCATGTAGCCACTCTGGGAGTCGGAGGTACCGAGGTTC
CTCCTTGAGTACAGTCCTTGGCGCGGCTTAAACTTGCCATCGCCCAAGGTGATCCTGGC
CGGGAGTTTCTTGCCCCTAAATGTGGCAGCTTCGAAGGAGGGCGGTGGCGACACCTT
>D_ECAM31
TATTGATTTAACATCCCC--GTCGGGAGCCCCACATATTT-CTGTATTT-
TCTTCGAGTTTTTCGCTCCCTTTCTTCGGCGACGTCTCGTGGCACTTTCGGTCCACAATT
CCGGGCGAAACGATCCGGCGCACAGTTCCGCGCTACGTGCGATCCAAAATGCGGCAATC
GATTTGCCGCGACCGCCGTCACAGTTCTTTTTATTGACACCTCACCTTGATAAAAACTCC
AAAGAAATTTATTTTTCTGCTAGGGCAAAAAAGTTAGGCTATCGGAAACAATCGGTCTTC
GATATCAGCATGCATTATTTGTTCGTATCGGGACATATCGATATGAAAATACCACACGAGC
TTATTCCCCGCTAAATCTGGCTTTTTTACTCATAAAACAACGGGTAACGATAATTTGATTT
TGAAAACACATATCTAACAGTCTAAAATCAAATTACATTAGCCATTTTAGAGCTTTAAAAT
ATCTGATGAATTTAATACTTTATTGGGTTGTGATAATGTTTATTTGAAAAGTGTGCAAACA
AGTTTGTCTCATAACAGAAAGAAATTTTACATTTTGCGCATATTTTTCGCTTGTTTTAATG
TTCAAATTTGAAAATTCTACAGACTAGGTGCAAAAAGAAAAAATATTTGCTTGTGTGGTC
GACCATGTGGACCTCCTCACTCCATGTAGCCACTCTGGGAGTCGGAGGTACCGAGGTTC
TTCCTTGAGTACAGTCCTTGGCGCGGCTTAAACTTGCCATCGCCCAAGGTGATCCTGGCC
GGGAGTTTCTTGCCCCTAAATGTGGCTGCTTCGAAGGAGGGCGGTGGCGACACCTT
>L_ECAM34
TATTGATTTAACATCCCC--GTCGGGAGCCC-ACATATTT-CTGTATTT-
TCTTCGAGTTTTCCGCTCCTATTCTTCGGCGACGTCTCGTGGCACTTTCGGTCCACAATT
CCGGGCGAAACGATCCGGCGCACAGTTCCGCGCTACGTGCGATCCAAAATGCGGCAATC
GATTTGCCGCGACCGCCGTCACAGTTCTTTTTATTGACACCTCACCTTGATAAAAACTCC
AAAGAAATTTATTTTTCTGCTAGGGCAAAAAAGTTAGGCTATCGGAAACAATCGGTCTTC
GATATCAGCATGCATTATTTGTTCGTATCGGGACATATCGATATGAAAATACCACACGAGC
TTATTCCCCGCTAAATCTGGCTTTTTTACTCATAAAACAACGGGTAACGGTAATTTGATTT
TGAAAACACATATCTAACAGTCTAAAATCAAATTACATTAGCCATTTTAGAGCTTTAAAAT
ATCTGATGAATTTAATACTTTATTCGGTTGTGATAATGTTTATTTGAAAAGTGTGCAAACA
AGTTTGTCTCATAACAGAAAGAAATTTTACATTTTGCGCATATTTTTCGCTTGTTTTAATG
TTCAAATTTGAAAATTCTACAGACTAGGTGCAGAAAGAAAAAATATTTGCTTGTGTGGTC
GACCATGTGGGCCTCCTCACTCCATGTAGCCACTCTGGGAGGCGGAGGTACCGAGGTTC
CTCCTTGAGTACAGTCCTGGGCGCGGCTTAAACTTGCCATCGCCCAAGGTGATCCTGGC
CGGGAGTTTCTTGCCCCTAAATGTGGCTGCTTCGAAGGAGGGCGGTGGCGACACCTT
>D_EGAB_N2
TATTGATTTAACATCCCC--GTCGGGAGCCC-ACATATTT-CTGTATTT-
TCCCCGAGTTTTCCGCTCCTATTCTTCGGCGACGTCTCGTGGCACTTTCGGTCCACAATT
CCGGGCGAAACGATCCGGCGCACAGTTCCGCGCTACGTGCGATCCAAAATGCGGCAATC
GATTTGCCGCGACCGCCGTCACAGTTCTTTTTATTGACACCTCACCTTGATAAAAACTCC
AAAGAAATTTATTTTTCTGCTAGGGCAAAAAAGTTAGGCTATCGGAAACAATCGGTCTTC
GATATCAGCATGCATTATTTGTTCGTATCGGGACATATCGATAAGAAAATACCACACGAGC
TTATTCCCCGCTAAATCTGGCTTTTTTACTCATAAAACAACGGGTAACGGTAATTTGATTT
TGAAAACACATATCTAACAGTCTAAAATCAACTTACATTAGCCATTTTAGAGCTTTAAAAT
ATCTGATGAATTTAATACTTTATTCGGTTGTGATAATGTTTATTTGAAAAGTGTGCAAACA

AGTTTGTCTCATAACAGAAAGAAATTTTACATTTTGCGCATATTTTTCGCTTGTTTTAATG
TTCAAATTTGAAAATTCTACAGA-
TAGGTGCAGAAAGAAAAAATATTTGCTTGTGTGGTCGACCATGTGGGCCTCCTCACTCC
ATGTAGCCACTCTGGGAGTCGGAGGTACCGAGGTTCCTCCTTGAGTACAGTCCTTGGCG
CGGCTTAAACTTGCCATCGCCCAAGGTGATCCTGGCCGGGAGTTTCTTGCCCCTAAATGT
GGCTGCTTCGAAGGAGGGCGGTGGCGACACCTT
>L_EGAB_C136
TATTGATTTAACATCCCC--GTCGGGAGCCC-ACATATTT-CTGTATTT-
TCTTCGAGTTTTCCGCTCCTATTCTTCGGCGACGTCTCGTGGCACTTTCGGTCCACAATT
CCGGGCGAAACGATCCGGCGCACAGTTCCGCGCTACGTGCGATCCAAAATGCGGCAATC
GATTTGCCGCGACCGCCGTCACAGTTCTTTTTATTGACACCTCACCTTGATAAAAACTCC
AAAGAAATTTATTTTTCTGCTAGGGCAAAAAAGTTAGGCTATCGGAGACAATCGGTCTTC
GATATCAGCATGCATTATTTGTTCGTATCGGGACATATCGATATGAAAATACCACACGAGC
TTATTCCCCGCTAAATCTGGCTTTTTTACTCATAAAACAACGGGTAACGGTAATTTGATTT
TGAAAACACATATCTAACAGTTTAAAATCAAATTACATTAGCCATTTTAGAGCTTTAAAAT
ATCTGATGAATTTAATACTTTATTCGGTTGTGATAATGTTTATTTGAAAAGTGTGCAAACA
AGTTTGTCTCATAACAGAAAGAAATTTTACATTTTGCGCATATTTTTCGCTTGTTTTAATG
TTCAAATTTGAAAATTCTACAGA-
TAGGTGCAAAAAGAAAAAATATTTGCTTGTGTGGTCGACCATGTGGGCCTTCTCACTCC
ATGTAGCCACTCTGGGAGTCGGAGGTACCGAGGTTCCTCCTTGAGTACAGTCCTTGGCG
CGGCTTAAACTTGCCATCGCCCAAGGTGATCCTGGCCGGGAGTTTCTTGCCCCTAAATGT
GGCTGCTTCGAAGGAGGGCGGTGGCGACACCTT
>L_EGAB_C3
TATTGATTTAACATCCCC--GTCGGGAGCCC-ACATATTT-CTGTATTT-
TCCCCGAGTTTTCCGCTCCTATTCTTCGGCGACGTCTCGTGGCACTTTCGGTCCACAATT
CCGGGCGAAACGATCCGGCGCACAGTTCCGCGCTACGTGCGATCCAAAATGCGGCAATC
GATTTGCCGCGACCGCCGTCACAGTTCTTTTTATTGACACCTCACCTTGATAAAAACTCC
AAAGAAATTTATTTTTCTGCTAGGGCAAAAAAGTTAGGCTATCGGAGACAATCGGTCTTC
GATATCAGCATGCATTATTTGTTCGTATCGGGACATATCGATATGAAAATACCACACGAGC
TTATTCCCCGCTAAATCTGGCTTTTTTACTCATAAAACAACGGGTAACGGTAATTTGATTT
TGAAAACACATATCTAACAGTTTAAAATCAAATTACATTAGCCATTTTAGAGCTTTAAAAT
ATCTGATGAATTTAATACTTTATTGGGTTGTGATAATGTTTATTTGAAAAGTGTGCAAACA
AGTTTGTCTCATAACAGAAAGAAATTTTACATTTTGCGCATATTTTTCGCTTGTTTTAATG
TTCAAATTTGAAAATTCTACAGA-
TAGGTGCAAAAAGAAAAAATATTTGCTTGTGTGGTCGACCATGTGGGCCTCCTCACTCC
ATGTAGCCACTCTGGGAGTCGGAGGTACCGAGGTTCCTCCTTGAGTACAGTCCTTGGCG
CGGCTTAAACTTGCCATCGCCCAAGGTGATCCTGGCCGGGAGTTTCTTGCCCCTAAATGT
GGCTGCTTCGAAGGAGGGCGGTGGCGACACCTT
>L_EGAB6A
TATTGATTTAACATCCCC--GTCGGGAGCCC-ACATATTT-CTGTATTT-
TCCCCGAGTTTTCCGCTCCTATTCTTCGGCGACGTCTCGTGGCACTTTCGGTCCGCAATT
CCGGGCGAAACGATCCGGCGCACAGTTCCGCGCTACGTGCGATCCAAAATGCGGCAATC
GATTTGCCGCGACCGCCGTCACAGTTCTTTTTATTGACACCTCACCTTGATAAAAACTCC
AAAGAAATTTATTTTTCTGCTAGGGCAAAAAAGTTAGGCTATCGGAAACAATCGGTCTTC
GATATCAGCATGCATTATTTGTTCGTATCGGGACATATCGATATGAAAATACCACACGAGC
TTATTCCCCGCTAAATCTGGCTTTATTACTCATAAAACAACGGGTAACGGTAATTTGATTT
TGAAAACACATATCTAACAGTCGAAAATCAAATTACATTAGCCATTTTAGAGCTTTAAAA
TATCTGATGAATTTAATACTTTATTCGGTTGTGATAATGTTTATTTGAAAAGTGTGCAAAC
AAGTTTGTCTCATAACAGAAAGAAATTTTAAATTTTGCGCAAATTTTTCGCTTGTTTTAAT
GTTCAAATTTTAAAATTCTACAGA-

TAGGTGCAGAAAGAAAAAATATTTGCTTGTGTGGTCGACCATGTGGGCCTCCTCACTCC
ATGTAGCCACTCTGGGAGTCGGAGGTACCGACGTTCCTCCTTGAGTACAGTCCTTGGCG
CGGCTTAAACTTGCCATCGCCCAAGGTGATCCTGGCCGGGAGTTTCTTGCCCCTAAATGT
GGCAGCTTCGAAGGAGGGCGGTGGCGACACCTT
>L_EGAB7A
TATTGATTTAACATCCCC--GTCGGGAGCCC-ACATATTT-CTGTATTT-
TCTTCGAGTTTTCCGCTCCTATTCTTCGGCGACGTCTCGTGGCACTTTCGGTCCACAATT
CCGGGCGAAACGATCCGGCGCACAGTTCCGCGCTACGTGCGATCCAAAATGCGGCAATC
GATTTGCCGCGACCGCCGTCACAGTTCTTTTTATTGACACCTCACCTTGATAAAAACTCC
AAAGATATTTATTTTTCTGCTAGGGCAAAAAAGTTAGGCTATCGGAAACAATCGGTCTTC
GATATCAGCATGCATTATTTGTTCGTATTGGGACATATCGATATGAAAATACCACACGAGC
TTATTCCCCGCTAAATCTGGCTTTTTTACTCATAAAACAACGGGTAACGGTAATTTGATTT
TGAAAACACATATCTAACAGTCTAAAATCAAATTACATTAGCCATTTTAGAGCTTTAAAAT
ATCTGATGAATTTAATACTTTATTCGGTTGTGATAATGTTTATTTGAAAAGTGTGCAAACA
AGTTTGTCTCATAACAGAAAGAAATTTTACATTTTGCGCATATTTTTCGCTTGTTTTAATG
TTCAAATTTGAAAATTCTACAGACTAGGTGCAGAAAGAAAAAATATTTGCTTGTGTGGTC
GACCATGTGGGCCTCCTCACTCCATGTAGCCACTCTGGGAGTCGGAGGTACCGAGGTTC
CTCCTTGAGTACAGTCCTTGGCGCGGCTTAAACTTGCCATCGCCCAAGGTGATCCTGGC
CGGGAGTTTCTTGCCCCTAAATGTGGCTGCTTCGAAGGAGGGCGGTGGCGACACCTT
>L_EGAB150A
TATTGATTTAACATCCCC--GTCGGGAGCCC-ACATATTT-CTGTATTT-CCC-
CGAGTTTTCCGCTCCTATTCTTCGGCGACGTCTCGTGGCACTT-
CGGTCCACAATTCCGGGCGAAACGATCCGGCGCACAGTTCCGCGCTACGTGCGATCCAA
AATGCGGCAATCGATTTGCCGCGACCGCCGTCACAGTTCTTTTTATTGACACCTCACCTT
GATAAAAACTCCAAAGAAATTTATTTTTCTGCTAGGGCAAAAAAGTTAGGCTATCGGAG
ACAATCGGTCTTCGATATCAGCATGCATTATTTGTTCGTATCGGGACATATCGATATGAAA
ATACCACACGAGCTTATTCCCCGCTAAATCTGGCTTTTTTACTCATAAAACAACGGGTAA
CGGTAATTTGATTTTGAAAACACATATCTAACAGTCTAAAATCAAATTACATTAGCCATTT
TAGAGCTTTAAAATATCTGATGAATTTAATACTTTATTGGGTTGTGATAATGTTTATTTGAA
AAGTGTGCAAACAAGTTTGTCTCATAACAGAAAGAAATTTTACATTTTGCGCATATTTTT
CGCTTGTTTTAATGTTCAAATTTGAAAATTCTACAGACTAGGTGCAAAAAGAAAAAATAT
TTGCTTGTGTGGTCGACCATGTGGGCCTCCTCACTCCATGTAGCCACTCTGGTTGTCGGA
GGTACCGAGGTTCCTCCTTGAGTACAGTCCTTGGCGCGGCTTAAACTTGCCATCGCCCA
AGGTGATCCTGGCCGGGAGTTTCTTGCCCCTAAATGTGGCTGCTTCGAAGGAGGGCGGT
GGCGACACCTT
>D_EGAB153A
TATTGATTTAACATCCCC--GTCGGGAGCCC-ACATATTT-
CTGTATTTTCCCCCGAGTTTTCCGCTCCTATTCTTCGGCGACGTCTCGTGGCACTTTCGGT
CCACAATTCCGGGCGAAACGATCCGGCGCACAGTTCCGCGCTACGTGCGATCCAAAATG
CGGCAATCGATTTGCCGCGACCGCCGTCACAGTTCTTTTTATTGACACCTCACCTTGATA
AAAACTCCAAAGAAATTTATTTTTCTGCTAGGGCAAAAAAGTTAGGCTATCGGAAACAA
TCGGTCTTCGATATCAGCATGCATTATTTGTTCGTATCGGGACATATCGATATGAAAATACC
ACACGAGCTTATTCCCCGCTAAATCTGGCTTTTTTACTCATAAAACAACGGGTAACGGTA
ATTTGATTTTGAAAACACATATCTAACAGTCTAAAATCAAATTACATTAGCCATTTTAGAG
CTTTAAAATATCTGATGAATTTAATACTTTATTGGGTTGTGATAATGTTTATTTGAAAAGTG
TGCAAACAAGTTTGTCTCATAACAGAAAGAAATTTTACATTTTGCGCATATTTTTCGCTTG
TTTTAATGTTCAAATTTGAAAATTCTACAGACTAGGTGCAAAAAGAAAAAATATTTGCTT
GTGTGGTCGACCATGTGGGCCTCCTCACTCCATGTAGCCACTCTGGGAGTCGGAGGTAC
CGAGGTTCCTCCTTGAGTACAGTCCTTGGCGCGGCTTAAACTTGCCATCGCCCAAGGTG
ATCCTGGCCGGGAGTTTCTTGCCCCTAAATGTGGCTGCTTCGAAGGAGGGCGGTGGCGA

CACCTT

Supplementary Note 3 | Alignment of locus 10 kb downstream of the t_MSE region. Dore= D. orena, D_ = dark haplogroup, L_= light haplogroup, ECAM = erecta from Cameroon, EGAB = erecta from Gabon.

>Dore
ATAGATCATAGATTGATTTCATGATAGTCATTGGGAATGTTT-
AAGTAACGACGCGATGGACGA-----------
ATGGCTGCCCCAACGGATCTACTTGTAGAGGAGCGGCAGGCGGCAGTGCGGCTCCGTCT
CGCTGGGATTGTCGGAGTACAGGGAGATGGTCCTGGCACTGAGATCGAAGATGCCCACG
GCAATGGTCTTGACCACCTCGTCGCAGCTGTTGTTCTCCCGCCAGACGCAGTAGACTCC
GCCGGACACATCGCCCAGCATGTGGCGCACATCCTGCTCGCTCATCGGCGGATTGTAGG
CTCCAAAGGTTTTCATCCGGGAGATGCTCGAGCTGATCATCAGATCGTTGGCCTGCTCCT
GACGGATGCGATCGAATCTGCA----------ATACACGTGTATACT---------ATACTTTCA---
AACAC------
TGCACCCACTGGTTAACATGGTAGTTGTGCTCGCCCAGCGGCACCTCCTTGATGTTCAAA
TACGACTCATTCTTGCGCTCCGGCGACGGAGCCATCTCCACATTGTAGCACATCTGGCGG
GGATCCCTGTTCGGCGTTGGCAAGTAGCTGGGATTAGTAAGACAATTTGAT--
TGCACAGTTAAACGTACGCCAGGAAGGTAAAGTTGATGGAGCAGGCATCCGCTGCACC
CACTCCCGCATCCCTGAGCACCCGGAACGCATCGTCCACGTTGCTGGTGGCCAACAGAG
CTCGGGTGAGGAAGTGTCGCGCTGGTGG-ACAAAAGAATGCAATA-
AAAAACGTAACTTTTT--------TTTTTT--
GTGTACGTACGGGTTTTGCCGCTGCGCAACAGCTCCGCACTGATGGTGTTAATGCTGAA
GACCAGTCCGTGGTGATTCTGGCTCATGGTGTATCCGGGCAGGTGGCCGGCGTAGC
>L_EGAB_C136
ATAGATCATAGATGGATTTCATGTTAGTCATTGGGAATATTT-
AAGCAAAGACGCGATGGATGATCGAGACTTCCATGGCTGCCCCAACGGATCTACTTGTA
GAGGAGCGGCAGGCGGCAGTGTGGCTCCGTCTCGCTGGGATTGTCGGAGTACAGAGAG
ATGGTCCTGGCACTGAGATCGAAGATGCCCACGGCGATGGTCTTGACCACCTCGTCGCA
GCTGTTGTTCTCCCGCCAGACGCAGTAGACTCCGCCGGACACATCGCCCAGCATGTGGC
GCACATCCTGCTCGCTCATCGGCGGATTGTAGGCTCCGAAGGTTTTCATCCTGGAGATGC
TCGAGCTGATCATCAGATCGTTGGCCTGCTCCTGACGGATGCGATCGAATCTGCAATATA
CATGCATACACGTGTATACTGCATATGAAATACTTTCATGCAACACTTTTCATGCACTCAC
TGGTTAACATGGTAGTTGTGCTCGCCCAGCGGCACCTCCTTGATGTTCAAATAGGACTCA
TTCTTGCGCTCCGGCGACGGAGCCATCTCCACATTGTAGCACATCTGGCGGGGATCCCTG
TTGGGCATTGGCAAGCAGCTCGGATTAGTAAAACAAAATGATGGTGCACAGATAAACGT
ACGCCAGGAAGGTAAAGTTGACGGAGCAGGCATCCGCTGCACCCACTCCCGCATCCTT
GAGCACCCGGAACGCATCGTCCACGTTGCTGGTGGCCAACAGAGCTCGGGTGATGAAG
TGTCGCGCTGGTGG-ATAAATGCATTCAATG-AAAACCGTCCCTTTTT--AAGGGATTCTTT-
-
GGGCACGTACGGGTTCTGCCGCTGCGCAAGAGCTCCGCACTGATGGTGTTAATGCTGAA
GACCAGTCCGTGGTGATTCTGGCTCATGGTGTATCCGGGCAGGTGTCCGGCGTAGC
>D_EGAB_N2
ATAGATCATAGATGGATTTCATGTTAGTCATTGGGAATATTT-
AAGCAAAGACGCGATGGATGATCGAGACTTCCATGGCTGCCCCAACGGATCTACTTGTA
GAGGAGCGGCAGGCGGCAGTGTGGCTCCGTCTCGCTGGGATTGTCGGAGTACAGGGAG
ATGGTCCTGGCACTGAGATCGAAGATGCCCACGGCGATGGTCTTGACCACCTCGTCGCA
GCTGTTGTTCTCCCGCCAGACGCAGTAGACTCCGCCGGACACATCGCCCAGCATGTGGC
GCACATCCTGCTCGCTCATCGGCGGATTGTAGGCTCCGAAGGTTTTCATCCTGGAGATGC

TCGAGCTGATCATCAGATCGTTGGCCTGCTCCTGACGGATGCGATCGAATCTGCAATATA
CATGCATACACGTGTATACTGCATATGAAATACTTTCATGCAACACTTTTCATGCACTCAC
TGGTTAACATGGTAGTTGTGCTCGCCCAGCGGCACCTCCTTGATGTTCAAATAGGACTCA
TTCTTGCGCTCCGGCGACGGAGCCATCTCCACATTGTAGCACATCTGGCGGGGATCCCTG
TTGGGCATTGGCAAGCAGCTCGGATTAGTAAAACAAAATGATGGTGCACAGATAAACGT
ACGCCAGGAAGGTAAAGTTGACGGAGCAGGCATCCGCTGCACCCACTCCCGCATCCTT
GAGCACCCGGAACGCATCGTCCACGTTGCTGGTGGCCAACAGAGCTCGGGTGATGAAG
TGTCGCGCTGGTGG-ATAAATGCATTCAATG-AAAACCGTCCCTTTTT--AAGGGATTCTTT-
-
GGGCACGTACGGGTTCTGCCGCTGCGCAAGAGCTCCGCACTGATGGTGTTAATGCTGAA
GACCAGTCCGTGGTGATTCTGGCTCATGGTGTATCCGGGCAGGTGTCCGGCGTAGC
>L_EGAB_C3
ATAGATCATAGATGGATTTCATGTTAGTCATTGGGAATATTT-
AAGCAAAGACGCGATGGATGATCGAGACTTCCATGGCTGCCCCAACGGATCTACTTGTA
GAGGAGCGGCAGGCGGCAGTGTGGCTCCGTCTCGCTGGGATTGTCGGAGTACAGAGAG
ATGGTCCTGGCACTGAGATCGAAGATGCCCACGGCGATGGTCTTGACCACCTCGTCGCA
GCTGTTGTTCTCCCGCCAGACGCAGTAGACTCCGCCGGACACATCGCCCAGCATGTGGC
GCACATCCTGCTCGCTCATCGGCGGATTGTAGGCTCCGAAGGTTTTCATCCTGGAGATGC
TCGAGCTGATCATCAGATCGTTGGCCTGCTCCTGACGGATGCGATCGAATCTGCAATATA
CATGCATACACGTGTATACTGCATATGAAATACTTTCATGCAACACTTTTCATGCACTCAC
TGGTTAACATGGTAGTTGTGCTCGCCCAGCGGCACCTCCTTGATGTTCAAATAGGACTCA
TTCTTGCGCTCCGGCGACGGAGCCATCTCCACATTGTAGCACATCTGGCGGGGATCCCTG
TTGGGCATTGGCAAGCAGCTCGGATTAGTAAAACAAAATGATGGTGCACAGATAAACGT
ACGCCAGGAAGGTAAAGTTGACGGAGCAGGCATCCGCTGCACCCACTCCCGCATCCTT
GAGCACCCGGAACGCATCGTCCACGTTGCTGGTGGCCAACAGAGCTCGGGTGATGAAG
TGTCGCGCTGGTGG-ATAAATGCATTCAATG-AAAACCGTCCCTTTTT--AAGGGATTCTTT-
-
GGGCACGTACGGGTTCTGCCGCTGCGCAAGAGCTCCGCACTGATGGTGTTAATGCTGAA
GACCAGTCCGTGGTGATTCTGGCTCATGGTGTATCCGGGCAGGTGTCCGGCGTAGC
>D_ECAM13
ATAGATCATAGATGGATTTCATGTTAGTCATTGGGAATATTT-
AAGCAAAGACGCGATGGATGATCGAGACTTCCATGGCTGCCCCAACGGATCTACTTGTA
GAGGAGCGGCAGGCGGCAGTGTGGCTCCGTCTCGCTGGGATTGTCGGAGTACAGAGAG
ATGGTCCTGGCACTGAGATCGAAGATGCCCACGGCGATGGTCTTGACCACCTCGTCGCA
GCTGTTGTTCTCCCGCCAGACGCAGTAGACTCCGCCGGACACATCGCCCAGCATGTGGC
GCACATCCTGCTCGCTCATCGGCGGATTGTAGGCTCCGAAGGTTTTCATCCTGGAGATGC
TCGAGCTGATCATCAGATCGTTGGCCTGCTCCTGACGGATGCGATCGAATCTGCAATATA
CATGCATACACGTGTATACTGCATATGAAATACTTTCATGCAACACTTTTCATGCACTCAC
TGGTTAACATGGTAGTTGTGCTCGCCCAGCGGCACCTCCTTGATGTTCAAATAGGACTCA
TTCTTGCGCTCCGGCGACGGAGCCATCTCCACATTGTAGCACATCTGGCGGGGATCCCTG
TTGGGCATTGGCAAACAGCTCGGATTAGTAAAACAAAATGATGGTGCACAGATAAACGT
ACGCCAGGAAGGTAAAGTTGACGGAGCAGGCATCCGCTGCACCCACTCCCGCATCCTT
GAGCACCCCGAACGCATCGTCCACGTTGCTGGGGGCCAACAGAGCTCGGGGAATGAAG
TGTCCCGCTGGTGG-ATAAATGCATTCAATG-AAAACCGTCCCTTTTT--AAGGGATTCTTT-
GGGGCACGTACGGGTTCTGCCCCTGCGCAAGAGCTCCGCACTGATGGTGTTAATGCTAA
AGACCAGTCCGTGGGGATTCTGGCTCATGGTGTATCCGGGCAGGTGTCCGGCGTAGC
>L_ECAM15
ATAGATCATAGATGGATTTCATGTTAGTCATTGGGAATATTT-
AAGCAAAGACGCGATGGATGATCGAGACTTCCATGGCTGCCCCAACGGATCTACTTGTA
GAGGAGCGGCAGGCGGCAGTGTGGCTCCGTCTCGCTGGGATTGTCGGAGTACAGGGAG

ATGGTCCTGGCACTGAGATCGAAGATGCCCACGGCGATGGTCTTGACCACCTCGTCGCA
GCTGTTGTTCTCCCGCCAGACGCAGTAGACTCCGCCGGACACATCGCCCAGCATGTGGC
GCACATCCTGCTCGCTCATTGGCGGATTGTAGGCTCCGAAGGTTTTCATCCTGGAGATGC
TCGAGCTGATCATCAGATCGTTGGCCTGCTCCTGACGGATGCGATCGAATCTGCAATATA
CATGCATACACGTGTATACTGCATATGAAATACTTTCATGCAACACTTTTCATGCACTCAC
TGGTTAACATGGTAGTTGTGCTCGCCCAGCGGCACCTCCTTGATGTTCAAATAGGACTCA
TTCTTGCGCTCCGGCGACGGAGCCATCTCCACATTGTAGCACATCTGGCGGGGATCCCTG
TTGGGCATTGGCAAGCAGCTCGGATTAGTAAAACAAAATGATGGTGCACAGATAAACGT
ACGCCAGGAAGGTAAAGTTGACGGAGCAGGCATCCGCTGCACCCACTCCCGCATCCTT
GAGCACCCGGAACGCATCGTCCACGTTGCTGGTGGCCAACAGAGCTCGGGTGATGAAG
TGTCGCGCTGGTGG-ATAAATGCATTCAATG-AAAACCGTCCCTTTTTT-
AAGGGATTCTTT--
GGGCACGTACGGGTTCTGCCGCTGCGCAAGAGCTCCGCACTGATGGTGTTAATGCTGAA
AACCAGTCCGTGGTGATTCTGGCTCATGGTGTATCCGGGCAGGTGTCCGGCGTAGC
>L_ECAM16
NNNNNNNNNNNNNNNNNNNNNNNNNNNNNNNNNNNNNNNNNNNNNNNNNNNNNNNNNN
NNNNNNNNNNNNNNNACTTCCATGGCTGCCCCAACGGATCTACTTGTCGAGGAGCGGCA
GGCGGCAGTGTGGCTCCGTCTCGCTGGGATTGTCGGAGTACAGAGAGATGGTCCTGGCA
CTGAGATCGAAGATGCCCACGGCGATGGTCTTGACCACCTCGTCGCAGCTGTTGTTCTC
CCGCCAGACGCAGTAGACTCCGCCGGACACATCGCCCAGCATGTGGCGCACATCCTGCT
CGCTCATCGGCGGATTGTAGGCTCCGAAGGTTTTCATCCTGGAGATGCTCTAGCTGATCA
TCAGATCGTTGGCCTGCTCCTGACGGATGCGATCGAATCTGCAATATACATGCATACACG
TGTATACTGCATATGAAATACTTTCATGCAACACTTTTCATGCACTCACTGGTTAACATGG
TAGTTGTGCTCGCCCAGCGGCACCTCCTTGATGTTCAAATAGGACTCATTCTTGCGCTCC
GGCGACGGAGCCATCTCCACATTGTAGCACATCTGGCGGGGATCCCTGTTGGGCATTGG
CAAGCAGCTCGCATTAGTAAAACAAAATGATGGTGCACAGATAAACGTACGCCAGGAA
GGTAAAGTTGACGGAGCAGGCATCCGCTGCACCCACTCCCGCATCCTTGAGCACCCGGA
AAGCATCGTCCACGTTGCTGGGGGCCAACAAAACTCGGGTGATGAAGTGTCCCCCTGG
TGGGATAAATGCATTCAATG-AAAACCGTCCCTTTTT--AAGGGATTCTTT--
GGGCACGTCCGGGTTCTGCCGCTGCCCAAGAGCTCCGCACTGATGGTGTTAATGCTAAA
AACCAGTCCGGGGGGATTCTGGCTCATGGTGTATCCGGGCAGGTGTCCGGCGTAGC
>D_ECAM19
ATAGATCATAGATGGATTTCATGTTAGTCATTGGGAATATTT-
AAGCAAAGACGCGATGGATGATCGAGACTTCCATGGCTGCCCCAACGGATCTACTTGTA
GAGGAGCGGCAGGCGGCAGTGTGGCTCCGTCTCGCTGGGATTGTCGGAGTACAGAGAG
ATGGTCCTGGCACTGAGATCGAAGATGCCCACGGCGATGGTCTTGACCACCTCGTCGCA
GCTGTTGTTCTCCCGCCAGACGCAGTAGACTCCGCCGGACACATCGCCCAGCATGTGGC
GCACATCCTGCTCGCTCATCGGCGGATTGTAGGCTCCGAAGGTTTTCATCCTGGAGATGC
TCGAGCTGATCATCAGATCGTTGGCCTGCTCCTGACGGATGCGATCGAATCTGCAATATA
CATGCATACACGTGTATACTGCATATGAAATACTTTCATGCAACACTTTTCATGCACTCAC
TGGTTAACATGGTAGTTGTGCTCGCCCAGCGGCACCTCCTTGATGTTCAAATAGGACTCA
TTCTTGCGCTCCGGCGACGGAGCCATCTCCACATTGTAGCACATCTGGCGGGGATCCCTG
TTGGGCATTGGCAAGCAGCTCGGATTAGTAAAACAAAATGATGGTGCACAGATAAACGT
ACGCCAGGAAGGTAAAGTTGACGGAGCAGGCATCCGCTGCACCCACTCCCGCATCCTT
GAGCACCCGGAACGCATCGTCCACGTTGCTGGTGGCCAACAGAGCTCGGGTGATGAAG
TGTCGCGCTGGTGG-ATAAATGCATTCAATG-AAAACCGTCCCTTTTT--
AAGGGATTCTTTTGGGGCACGTACGGGTTCTGCCGCTGCGCAAGAGCTCCGCACTGATG
GTGTTAATGCTGAAGACCAGTCCGTGGTGATTCTGGCTCATGGTGTATCCGGGCAGGTGT
CCGGCGTAGC
>L_ECAM20

NNNNNNNNNNNNNNNNNNNNNNNNNNNNNNNNNNNNNNNNNNNNNNNNNNNNNNNNNNNN
NNNNNNNNNNNNNNNACTTCCATGGCTGCCCCAACGGATCTACTTGTAGAGGAGCGGCA
GGCGGCAGNGTGGCTCCGTCTCGCTGGGATTGTCGGAGTACAGGGAGATGGTCCTGGC
ACTGAGATCGAAGATGCCCACGGCGATGGTCTTGACCACCTCGTCGCAGCTGTTGTTCT
CCCGCCAGACGCAGTAGACTCCGCCGGACACATCGCCCAGCATGTGGCGCACATCCTGC
TCGCTCATCGGCGGATTGTAGGCTCCGAAGGTTTTCATCCTGGAGATGCTCGAGCTGATC
ATCAGATCGTTGGCCTGCTCCTGACGGATGCGATCGAATCTGCAATATACATGCATACAC
GTGTATACTGCATATGAAATACTTTCATGCAACACTTTTCATGCACTCACTGGTTAACATG
GTAGTTGTGCTCGCCCAGCGGCACCTCCTTGATGTTCAAATAGGACTCATTCTTGCGCTC
CGGCGACGGAGCCATCTCCACATTGTAGCACATCTGGCGGGGATCCCTGTTGGGCATTG
GCAAGCAGCTCGGATTAGTAAAACAAAATGATGGTGCACAGATAAACGTACGCCAGGA
AGGTAAAGTTGACGGAGCAGGCATCCGCTGCACCCACTCCCGCATCCTTGAGCACCCGG
AACGCATCGTCCACGTTGCTGGTGGCCAACAGAGCTCGGGTGATGAAGTGTCGCGCTG
GTGG-ATAAATGCATTCAATG-AAAACCGTCCCTTTTT--AAGGGATTCTTT--
GGGCACGTACGGGTTCTGCCGCTGCGCAAGAGCTCCGCACTGTGGGTGTTAATGCTGAA
GACCAGTCCGTGGTGATTCTGGCTCATGGTGTATCCGGGCAGGGGTCCGGCGTAAC
>D_ECAM21b
ATAGATCATAGATGGATTTCATGTTAGTCATTGGGAATATTT-
AAGCAAAGACGCGATGGATGATCGAGACTTCCATGGCTGCCCCAACGGATCTACTTGTA
GAGGAGCGGCAGGCGGCAGTGTGGCTCCGTCTCGCTGGGATTGTCGGAGTACAGAGAG
ATGGTCCTGGCACTGAGATCGAAGATGCCCACGGCGATGGTCTTGACCACCTCGTCGCA
GCTGTTGTTCTCCCGCCAGACGCAGTAGACTCCGCCGGACACATCGCCCAGCATGTGGC
GCACATCCTGCTCGCTCATCGGCGGATTGTAGGCTCCGAAGGTTTTCATCCTGGAGATGC
TCGAGCTGATCATCAGATCGTTGGCCTGCTCCTGACGGATGCGATCGAATCTGCAATATA
CATGCATACACGTGTATACTGCATATGAAATACTTTCATGCAACACTTTTCATGCACTCAC
TGGTTAACATGGTAGTTGTGCTCGCCCAGCGGCACCTCCTTGATGTTCAAATAGGACTCA
TTCTTGCGCTCCGGCGACGGAGCCATCTCCACATTGTAGCACATCTGGCGGGGATCCCTG
TTGGGCATTGGCAAGCAGCTCGGATTAGTAAAACAAAATGATGGTGCACAGATAAACGT
ACGCCAGGAAGGTAAAGTTGACGGAGCAGGCATCCGCTGCACCCACTCCCGCATCCTT
GAGCACCCGGAACGCATCGTCCACGTTGCTGGTGGCCAACAGAGCTCGGGTGATGAAG
TGTCGCCCTGGTGG-ATAAATGCATTCAATG-AAAACCGTCCCTTTTT--
AAGGGATTCTTTTGGGGCACGTACGGGTTCTGCCGCTGCGCAAGAGCTCCGCACTGATG
GTGTTAATGCTGAAGACCAGTCCGTGGTGATTCTGGCTCATGGTGTATCCGGGCAGGTGT
CCGGCGTAGC
>L_ECAM22b
ATAGATCATAGATGGATTTCATGTTAGTCATTGGGAATATTT-
AAGCAAAGACGCGATGGATGATCGAGACTTCCATGGCTGCCCCAACGGATCTACTTGTA
GAGGAGCGGCAGGCGGCAGTGTGGCTCCGTCTCGCTGGGATTGTCGGAGTACAGAGAG
ATGGTCCTGGCACTGAGATCGAAGATGCCCACGGCGATGGTCTTGACCACCTCGTCGCA
GCTGTTGTTCTCCCGCCAGACGCAGTAGACTCCGCCGGACACATCGCCCAGCATGTGGC
GCACATCCTGCTCGCTCATCGGCGGATTGTAGGCTCCGAAGGTTTTCATCCTGGAGATGC
TCGAGCTGATCATCAGATCGTTGGCCTGCTCCTGACGGATGCGATCGAATCTGCAATATA
CATGCATACACGTGTATACTGCATATGAAATACTTTCATGCAACACTTTTCATGCACTCAC
TGGTTAACATGGTAGTTGTGCTCGCCCAGCGGCACCTCCTTGATGTTCAAATAGGACTCA
TTCTTGCGCTCCGGCGACGGAGCCATCTCCACATTGTAGCACATCTGGCGGGGATCCCTG
TTGGGCATTGGCAAGCAGCTCGGATTAGTAAAACAAAATGATGGTGCACAGATAAACGT
ACGCCAGGAAGGTAAAGTTGACGGAGCAGGCATCCGCTGCACCCACTCCCGCATCCTT
GAGCACCCGGAACGCATCGTCCACGTTGCTGGTGGCCAACAGAGCTCGGGTGATGAAG
TGTCGCGCTGGTGG-ATAAATGCATTCAATG-AAAACCGTCCCTTTTT--AAGGGATTCTTT-
-

GGGCACGTACGGGTTCTGCCGCTGCGCAAGAGCTCCGCACTGATGGTGTTAATGCTGAA
GACCAGTCCGTGGTGATTCTGGCTCATGGTGTATCCGGGCAGGTGTCCGGCGTACC
>D_ECAM24b
ATAGATCATAGATGGATTTCATGTTAGTCATGGGGAATATTT-
AAGCAAAGACGCGATGGATGATCGAGACTTCCATGGCTGCCCCAACGGATCTACTTGTA
GAGGAGCGGCAGGCGGCAGTGTGGCTCCGTCTCGCTGGGATTGTCGGAGTACAGAGAG
ATGGTCCTGGCACTGAGATCGAAGATGCCCACGGCGATGGTCTTGACCACCTCGTCGCA
GCTGTTGTTCTCCCGCCAGACGCAGTAGACTCCGCCGGACACATCGCCCAGCATGTGGC
GCACATCCTGCTCGCTCATCGGCGGATTGTAGGCTCCGAAGGTTTTCATCCTGGAAATGC
TCGAGCTGATCATCAGATCGTTGGCCTGCTCCTGACGGATGCGATCGAATCTGCAATATA
CATGCATACACGTGTA---
TGCATATGAAATACTTTCATGCAACACTTTTCATGCACTCACTGGTTAACATGGTAGTTGT
GCTCGCCCAGCGGCACCTCCTTGATGTTCAAATAGGACTCATTCTTGCGCTCCGGCGAC
GGAGCCATCTCCACATTGTAGCACATCTGGCGGGGATCCCTGTTGGGCATTGGCAAGCA
GCTCGCATTAGTAAAACAAAATGATGGTGCACAGATAAACGTACGCCAGGAAGGTAAAG
TTGACGGAGCAGGCATCCGCTGCACCCACTCCCGCATCCTTGAGCACCCGGAACGCATC
GTCCACGTTGCTGGTGGCCAACAGAGCTCGGGTAATGAAGTGTCGCGCTGGTGG-
ATAAATGCATTCAATG-AAAACCGTCCCTTTTT--AAGGGATTCTTT--
GGGCACGTACGGGTTCTGCCGCTGCGCAAGAGCTCCGCACTGATGGTGTTAATGCTGAA
GACCAGTCCGTGGTGATTCTGGCTCATGGTGTATCCGGGCAGGTGTCCGGCGTAGC
>L_ECAM25
NNNNNNNNNNNNNNNNNNNNNNNNNNNNNNNNNNNNNNNNNNNNNNNNNNNNNNNNNN
NNNNNNNNNNNNNNNACTTCCATGGCTGCCCCAACGGATCTACTTGTAGAGGAGCGGCA
GGCGGCAGTGTGGCTCCGTCTCGCTGGGATTGTCGGAGTACAGGGAGATGGTCCTGGCA
CTGAGATCGAAGATGCCCACGGCGATGGTCTTGACCACCTCGTCGCAGCTGTTGTTCTC
CCGCCAGACGCAGTAGACTCCGCCGGACACATCGCCCAGCATGTGGCGCACATCCTGCT
CGCTCATCGGCGGATTGTAGGCTCCGAAGGTTTTCATCCTGGAGATGCTCGAGCTGATCA
TCAGATCGTTGGCCTGCTCCTGACGGATGCGATCGAATCTGCAATATACATGCATACACG
TGTATACTGCATATGAAATACTTTCATGCAACACTTTTCATGCACTCACTGGTTAACATGG
TAGTTGTGCTCGCCCAGCGGCACCTCCTTGATGTTCAAATAGGACTCATTCTTGCGCTCC
GGCGACGGAGCCATCTCCACATTGTAGCACATCTGGCGGGGATCCCTGTTGGGCATTGG
CAAGCAGCTCGGATTAGTAAAACAAAATGATGGTGCACAGATAAACGTACGCCAGGAA
GGTAAAGTTGACGGAGCAGGCATCCGCTGCACCCACTCCCGCATCCTTGAGCACCCGGA
ACGCATCGTCCACGTTGCTGGTGGCCAACAGAGCTCGGGTGATGAAGTGTCGCGCTGGT
GG-ATAAATGCATTCAATG-AAAACCGTCCCTTTTTT-AAGGGATTCTTT--
GGGCACGTACGGGTTCTGCCGCTGCGCAAGAGCTCCGCACTGATGGTGTAAATGCTGAA
GACCAGTCCGGGGGGATTCTGGCTCATGGTGTATCCGGGCAGGTGTCCGGCGTAGC
>D_ECAM26
NNNNNNNNNNNNNNNNNNNNNNNNNNNNNNNNNNNNNNNNNNNNNNNNNNNNNNNNNN
NNNNNNNNNNNNNNNACTTCCATGGCTGCCCCAACGGATCTACTTGTAGAGGAGCGGCA
GGCGGCAGTGTGGCTCCGTCTCGCTGGGATTGTCGGAGTACAGAGAGATGGTCCTGGCA
CTGAGATCGAAGATGCCCACGGCGATGGTCTTGACCACCTCGTCGCAGCTGTTGTTCTC
CCGCCAGACGCAGTAGACTCCGCCGGACACATCGCCCAGCATGTGGCGCACATCCTGCT
CGCTCATCGGCGGATTGTAGGCTCCGAAGGTTTTCATCCTGGAGATGCTCGAGCTGATCA
TCAGATCGTTGGCCTGCTCCTGACGGATGCGATCGAATCTGCAATATACATGCATACACG
TGTATACTGCATATGAAATACTTTCATGCAACACTTTTCATGCACTCACTGGTTAACATGG
TAGTTGTGCTCGCCCAGCGGCACCTCCTTGATGTTCAAATAGGACTCATTCTTGCGCTCC
GGCGACGGAGCCATCTCCACATTGTAGCACATCTGGCGGGGATCCCTGTTGGGCATTGG
CAAGCAGCTCGGATTAGTAAAACAAAATGATGGTGCACAGATAAACGTACGCCAGGAA
GGTAAAGTTGACGGAGCAGGCATCCGCTGCACCCACTCCCGCATCCTTGAGCACCCGGA

ACGCATCGTCCACGTTGCTGGTGGCCAACAGAGCTCGGGTGATGAAGTGTCGCGCTGGT
GG-ATAAATGCATTCAATG-AAAACCGTCCCTTTTT--AAGGGATTCTTT--
GGGCACGTACGGGTTCTGCCGCTGCGCAAGAGCTCCGCACTGATGGTGTTAATGCTGAA
GACCAGTCCGGGGTGATTCTGGCTCATGGGGTATCCGGGCAGGTGTCCGGCGTAGC
>D_ECAM27
NNNNNNNNNNNNNNNNNNNNNNNNNNNNNNNNNNNNNNNNNNNNNNNNNNNNNNNNNNNN
NNNNNNNNNNNNNNNNACTTCCATGGCTGCCCCAACGGATCTACTTGTAGAGGAGCGGCA
GGCGGCAGTGTGGCTCCGTCTCGCTGGGATTGTCGGAGTACAGAGAGATGGTCCTGGCA
CTGAGATCGAAGATGCCCACGGCGATGGTCTTGACCACCTCGTCGCAGCTGTTGTTCTC
CCGCCAGACGCAGTAGACTCCGCCGGACACATCGCCCAGCATGTGGCGCACATCCTGCT
CGCTCATCGGCGGATTGTAGGCTCCGAAGGTTTTCATCCTGGAGATGCTCGAGCTGATCA
TCAGATCGTTGGCCTGCTCCTGACGGATGCGATCGAATCTGCAATATACATGCATACACG
TGTATACTGCATATGAAATACTTTCATGCAACACTTTTCATGCACTCACTGGTTAACATGG
TAGTTGTGCTCGCCCAGCGGCACCTCCTTGATGTTCAAATAGGACTCATTCTTGCGCTCC
GGCGACGGAGCCATCTCCACATTGTAGCACATCTGGCGGGGATCCCTGTTGGGCATTGG
CAAGCAGCTCGGATTAGTAAAACAAAATGATGGTGCACAGATAAACGTACGCCAGGAA
GGTAAAGTTGACGGAGCAGGCATCCGCTGCACCCACTCCCGCATCCTTGAGCACCCGGA
ACGCATCGTCCACGTTGCTGGTGGCCAACAGAGCTCGGGTGATGAAGTGTCGCGCTGGT
GG-ATAAATGCATTCAATG-AAAACCGTCCCTTTTT---AGGGATTCTTT--
GGGCACGTACGGGTTCTGCCGCTGCGCAAGAGCTCCGCACTGATGGTGTTAATGCTGAA
GACCAGTCCGTGGTGATTCTGGCTCATGGTGTATCCGGGCAGGTGTCCGGCGTAGC
>L_ECAM28
NNNNNNNNNNNNNNNNNNNNNNNNNNNNNNNNNNNNNNNNNNNNNNNNNNNNNNNNNNNN
NNNNNNNNNNNNNNNNACTTCCATGGCTGCCCCAACGGATCTACTTGTAGAGGAGCGGCA
GGCGGCAGTGTGGCTCCGTCTCGCTGGGATTGTCGGAGTACAGGGAGATGGTCCTGGCA
CTGAGATCGAAGATGCCCACGGCGATGGTCTTGACCACCTCGTCGCAGCTGTTGTTCTC
CCGCCAGACGCAGTAGACTCCGCCGGACACATCGCCCAGCATGTGGCGCACATCCTGCT
CGCTCATCGGCGGATTGTAGGCTCCGAAGGTTTTCATCCTGGAGATGCTCGAGCTGATCA
TCAGATCGTTGGCCTGCTCCTGACGGATGCGATCGAATCTGCAATATACATGCATACACG
TGTATACTGCATATGAAATACTTTCATGCAACACTTTTCATGCACTCACTGGTTAACATGG
TAGTTGTGCTCGCCCAGCGGCACCTCCTTGATGTTCAAATAGGACTCATTCTTGCGCTCC
GGCGACGGAGCCATCTCCACATTGTAGCACATCTGGCGGGGATCCCTGTTGGGCATTGG
CAAGCAGCTCGGATTAGTAAAACAAAATGATGGTGCACAGATAAACGTACGCCAGGAA
GGTAAAGTTGACGGAGCAGGCATCCGCTGCACCCACTCCCGCATCCTTGAGCACCCGGA
ACGCATCGTCCACGTTGCTGGTGGCCAACAGAGCTCGGGTGATGAAGTGTCGCGCTGGT
GG-ATAAATGCATTCAATG-AAAACCGTCCCTTTTTT-AAGGGATTCTTT--
GGGCACGTACGGGTTCTGCCGCTGCGCAAGAGCTCCGCACTGTGGGTGTTAATGCTGAA
GACCAGTCCGTGGTGATTCTGGCTCATGGTGTATCCGGGCAGGTGTCCGGCGTAGC
>D_ECAM31
ATAGATCATAGATGGATTTCATGTTAGTCATTGGGAATATTT-
AAGCAAAGACGCGATGGATGATCGAGACTTCCATGGCTGCCCCAACGGATCTACTTGTA
GAGGAGCGGCAGGCGGCAGTGTGGCTCCGTCTCGCTGGGATTGTCGGAGTACAGAGAG
ATGGTCCTGGCACTGAGATCGAAGATGCCCACGGCGATGGTCTTGACCACCTCGTCGCA
GCTGTTGTTCTCCCGCCAGACGCAGTAGACTCCGCCGGACACATCGCCCAGCATGTGGC
GCACATCCTGCTCGCTCATCGGCGGATTGTAGGCTCCGAAGGTTTTCATCCTGGAGATGC
TCGAGCTGATCATCAGATCGTTGGCCTGCTCCTGACGGATGCGATCGAATCTGCAATATA
CATGCATACACGTGTATACTGCATATGAAATACTTTCATGCAACACTTTTCATGCACTCAC
TGGTTAACATGGTAGTTGTGCTCGCCCAGCGGCACCTCCTTGATGTTCAAATAGGACTCA
TTCTTGCGCTCCGGCGACGGAGCCATCTCCACATTGTAGCACATCTGGCGGGGATCCCTG
TTGGGCATTGGCAAGCAGCTCGGATTAGTAAAACAAAATGATGGTGCACAGATAAACGT

ACGCCAGGAAGGTAAAGTTGACGGAGCAGGCATCCGCTGCACCCACTCCCGCATCCTT
GAGCACCCGGAACGCATCGTCCACGTTGCTGGTGGCCAACAGAGCTCGGGTGATGAAG
TGTCGCGCTGGTGG-ATAAATGCATTCAATG-AAAACCGTCCCTTTTT--AAGGGATTCTTT-
-
GGGCACGTACGGGTTCTGCCGCTGCGCAAGAGCTCCGCACTGATGGTGTTAATGCTGAA
GACCAGTCCGTGGTGATTCTGGCTCATGGGGTATCCGGGCAGGTGTCCGGCGTAGC
>L_ECAM38
NNNNNNNNNNNNNNNNNNNNNNNNNNNNNNNNNNNNNNNNNNNNNNNNNNNNNNNNN
NNNNNNNNNNNNNNNACTTCCATGGCTGCCCCAACGGATCTACTTGTAGAGGAGCGGCA
GGCGGCAGTGTGGCTCCGTCTCGCTGGGATTGTCGGAGTACAGGGAGATGGTCCTGGCA
CTGAGATCGAAGATGCCCACGGCGATGGTCTTGACCACCTCGTCGCAGCTGTTGTTCTC
CCGCCAGACGCAGTAGACTCCGCCGGACACATCGCCCAGCATGTGGCGCACATCCTGCT
CGCTCATCGGCGGATTGTAGGCTCCGAAGGTTTTCATCCTGGAGATGCTCGAGCTGATCA
TCAGATCGTTGGCCTGCTCCTGACGGATGCGATCGAATCTGCAATATACATGCATACACG
TGTATACTGCATATGAAATACTTTCATGCAACACTTTTCATGCACTCACTGGTTAACATGG
TAGTTGTGCTCGCCCAGCGGCACCTCCTTGATGTTCAAATAGGACTCATTCTTGCGCTCC
GGCGACGGAGCCATCTCCACATTGTAGCACATCTGGCGGGGATCCCTGTTGGGCATTGG
CAAGCAGCTCGGATTAGTAAAACAAAATGATGGTGCACAGATAAACGTACGCCAGGAA
GGTAAAGTTGACGGAGCAGGCATCCGCTGCACCCACTCCCGCATCCTTGAGCACCCGGA
ACGCATCGTCCACGTTGCTGGTGGCCAACAGAGCTCGGGTGATGAAGTGTCGCGCTGGT
GG-ATAAATGCATTCAATG-AAAACCGTCCCTTTTT--AAGGGATTCTTT-
GGGGCACGTACGGGTTCTGCCGCTGCGCAAGAGCTCCGCACTGATGGTGTTAATGCTGA
AGACCAGTCCGTGGGGATTCTGGCTCATGGTGTATCCGGGCAGGTGTCCGGCGTAGC
>L_EGAB6A
ATAGATCATAGATGGATTTCATTTTAGTCATTGGGAATATTTTAAGCAAAGACGCGATGGA
TGATCGAGACTTCCATGGCTGCCCCAACGGATCTACTTGTAGAGGAGCGGCAGGCGGCA
GTGTGGCTCCGTCTCGCTGGGATTGTCGGAGTACAGAGAGATGGTCCTGGCACTGAGAT
CGAAGATGCCCACGGCGATGGTCTTGACCACCTCGTCGCAGCTGTTGTTCTCCCGCCAG
ACGCAGTAGACTCCGCCGGACACATCGCCCAGCATGTGGCGCACATCCTGCTCGCTCAT
CGGCGGATTGTAGGCTCCGAAGGTTTTCATCCTGGAGATGCTCGAGCTGATCATCAGATC
GTTGGCCTGCTCCTGACGGATGCGATCGAATCTGCAATATACATGCATACACGTGTATACT
GCATATGAAATACTTTCATGCAACACTTTTCATGCACTCACTGGTTAACATGGTAGTTGTG
CTCGCCCAGCGGCACCTCCTTGATGTTCAAATAGGACTCATTCTTGCGCTCCGGCGACG
GAGCCATCTCCACATTGTAGCACATCTGGCGGGGATCCCTGTTGGGCATTGGCAAGCAG
CTCGGATTAGTAAAACAAAATGATGGTGCACAGATAAACGTACGCCAGGAAGGTAAAGT
TGACGGAGCAGGCATCCGCTGCACCCACTCCCGCATCCTTGAGCACCCGGAACGCATCG
TCCACGTTGCTGGTGGCCAACAGAGCTCGGGTGATGAAGTGTCGCGCTGGTGG-
ATAAATGCATTCAATG-AAAACCGTCCCTTTTT--AAGGGATTCTTT--
GGGCACGTACGGGTTCTGCCGCTGCGCAAGAGCTCCGAACTGATGGTGTTAATGCTGAA
GACCAGTCCGTGGTGATTCTGGCTCATGGTGTATCCGGGCAGGTGTCCGGCGTAGC
>L_EGAB6B
ATAGATCATAGATGGATTTCATGTTAGTCATTGGGAATATTT-
AAGCAAAGACGCGATGGATGATCGAGACTTCCATGGCTGCCCCAACGGATCTACTTGTA
GAGGAGCGGCAGGCGGCAGTGTGGCTCCGTCTCGCTGGGATTGTCGGAGTACAGAGAG
ATGGTCCTGGCACTGAGATCGAAGATGCCCACGGCGATGGTCTTGACCACCTCGTCGCA
GCTGTTGTTCTCCCGCCAGACGCAGTAGACTCCGCCGGACACATCGCCCAGCATGTGGC
GCACATCCTGCTCGCTCATCGGCGGATTGTAGGCTCCGAAGGTTTTCATCCTGGAGATGC
TCGAGCTGATCATCAGATCGTTGGCCTGCTCCTGACGGATGCGATCGAATCTGCAATATA
CATGCATACACGTGTATACTGCATATGAAATACTTTCATGCAACACTTTTCATGCACTCAC
TGGTTAACATGGTAGTTGTGCTCGCCCAGCGGCACCTCCTTGATGTTCAAATAGGACTCA

TTCTTGCGCTCCGGCGACGGAGCCATCTCCACATTGTAGCACATCTGGCGGGGATCCCTG
TTGGGCATTGGCAAGCAGCTCGGATTAGTAAAACAAAATGATGGTGCACAGATAAACGT
ACGCCAGGAAGGTAAAGTTGACGGAGCAGGCATCCGCTGCACCCACTCCCGCATCCTT
GAGCACCCGGAACGCATCGTCCACGTTGCTGGTGGCCAACAGAGCTCGGGTGATGAAG
TGTCGCGCTGGTGG-ATAAATGCATTCAATG-AAAACCGTCCCTTTTT--AAGGGATTCTTT-
-
GGGCACGTACGGGTTCTGCCGCTGCGCAAGAGCTCCGCACTGATGGTGTTAATGCTGAA
GACCAGTCCGTGGTGATTCTGGCTCATGGTGTATCCGGGCAGGTGTCCGGCGTAGC
>D_EGAB7
ATAGATCATAGATGGATTTCATGTTAGTCATTGGGAATATTT-
AAGCAAAGACGCGATGGATGATCGAGACTTCCATGGCTGCCCCAACGGATCTACTTGTA
GAGGAGCGGCAGGCGGCAGTGTGGCTCCGTCTCGCTGGGATTGTCGGAGTACAGAGAG
ATGGTCCTGGCACTGAGATCGAAGATGCCCACGGCGATGGTCTTGACCACCTCGTCGCA
GCTGTTGTTCTCCCGCCAGACGCAGTAGACTCCGCCGGACACATCGCCCAGCATGTGGC
GCACATCCTGCTCGCTCATCGGCGGATTGTAGGCTCCGAAGGTTTTCATCCTGGAGATGC
TCGAGCTGATCATCAGATCGTTGGCCTGCTCCTGACGGATGCGATCGAATCTGCAATATA
CATGCATACACGTGTATACTGCATATGAAATACTTTCATGCAACACTTTTCATGCACTCAC
TGGTTAACATGGTAGTTGTGCTCGCCCAGCGGCACCTCCTTGATGTTCAAATAGGACTCA
TTCTTGCGCTCCGGCGACGGAGCCATCTCCACATTGTAGCACATCTGGCGGGGATCCCTG
TTGGGCATTGGCAAGCAGCTCGGATTAGTAAAACAAAATGATGGTGCACAGATAAACGT
ACGCCAGGAAGGTAAAGTTGACGGAGCAGGCATCCGCTGCACCCACTCCCGCATCCTT
GAGCACCCGGAACGCATCGTCCACGTTGCTGGTGGCCAACAGAGCTCGGGTGATGAAG
TGTCGCGCTGGTGG-ATAAATGCATTCAATG-AAAACCGTCCCTTTTT--AAGGGATTCTTT-
-
GGGCACGTACGGGTTCTGCCGCTGCGCAAGAGCTCCGAACTGATGGTGTTAATGCTGAA
GACCAGTCCGTGGTGATTCTGGCTCATGGTGTATCCGGGCAGGTGTCCGGCGTAGC
>L_EGAB150A
ATAGATCATAGATGGATTTCATGTTAGTCATTGGGAATATTT-
AAGCAAAGACGCGATGGATGATCGAGACTTCCATGGCTGCCCCAACGGATCTACTTGTA
GAGGAGCGGCAGGCGGCAGTGTGGCTCCGTCTCGCTGGGATTGTCGGAGTACAGAGAG
ATGGTCCTGGCACTGAGATCGAAGATGCCCACGGCGATGGTCTTGACCACCTCGTCGCA
GCTGTTGTTCTCCCGCCAGACGCAGTAGACTCCGCCGGACACATCGCCCAGCATGTGGC
GCACATCCTGCTCGCTCATCGGCGGATTGTAGGCTCCGAAGGTTTTCATCCTGGAGATGC
TCGAGCTGATCATCAGATCGTTGGCCTGCTCCTGACGGATGCGATCGAATCTGCAATATA
CATGCATACACGTGTATACTGCATATGAAATA-
TTTCATGCAACACTTTTCATGCACTCACTGGTTAACATGGTAGTTGTGCTCGCCCAGCGG
CACCTCCTTGATGTTCAAATAGGACTCATTCTTGCGCTCCGGCGACGGAGCCATCTCCAC
ATTGTAGCACATCTGGCGGGGATCCCTGTTGGGCATTGGCAAGCAGCTCGGATTAGTAA
AACAAAATGATGGTGCACAGATAAACGTACGCCAGGAAGGTAAAGTTGACGGAGCAGG
CATCCGCTGCACCCACTCCCGCATCCTTGAGCACCCGGAACGCATCGTCCACGTTGCTG
GTGGCCAACAGAGCTCGGGTGATGAAGTGTCGCGCTGGTGG-ATAAATGCATTCAATG-
AAAACCGTCCCTTTTT-AAAGGGATTCTTT--
GGGCACGTACGGGTTCTGCCGCTGCGCAAGAGCTCCGCACTGATGGTGTTAATGCTGAA
AACCAGTCCGTGGTGATTCTGGCTCATGGTGTATCCGGGCAGGTGTCCGGCGTAGC
>L_EGAB150B
ATAGATCATAGATGGATTTCATGTTAGTCATTGGGAATATTT-
AAGCAAAGACGCGATGGATGATCGAGACTTCCATGGCTGCCCCAACGGATCTACTTGTA
GAGGAGCGGCAGGCGGCAGTGTGGCTCCGTCTCGCTGGGATTGTCGGAGTACAGAGAG
ATGGTCCTGGCACTGAGATCGAAGATGCCCACGGCGATGGTCTTGACCACCTCGTCGCA
GCTGTTGTTCTCCCGCCAGACGCAGTAGACTCCGCCGGACACATCGCCCAGCATGTGGC

GCACATCCTGCTCGCTCATCGGCGGATTGTAGGCTCCGAAGGTTTTCATCCTGGAGATGC
TCGAGCTGATCATCAGATCGTTGGCCTGCTCCTGACGGATGCGATCGAATCTGCAATATA
CATGCATACACGTGTATACTGCATATGAAATA-
TTTCATGCAACACTTTTCATGCACTCACTGGTTAACATGGTAGTTGTGCTCGCCCAGCGG
CACCTCCTTGATGTTCAAATAGGACTCATTCTTGCGCTCCGGCGACGGAGCCATCTCCAC
ATTGTAGCACATCTGGCGGGGATCCCTGTTGGGCATTGGCAAGCAGCTCGGATTAGTAA
AACAAAATGATGGTGCACAGATAAACGTACGCCAGGAAGGTAAAGTTGACGGAGCAGG
CATCCGCTGCACCCACTCCCGCATCCTTGAGCACCCGGAACGCATCGTCCACGTTGCTG
GTGGCCAACAGAGCTCGGGTGATGAAGTGTCGCGCTGGTGG-ATAAATGCATTCAATG-
AAAACCGTCCCTTTTT--AAGGGATTCTTT--
GGGCACGTACGGGTTCTGCCGCTGCGCAAGAGCTCCGCACTGATGGTGTTAATGCTGAA
AACCAGTCCGTGGTGATTCTGGCTCATGGTGTATCCGGGCAGGTGTCCGGCGTAGC
>L_EGAB152A
ATAGATCATAGATGGATTTCATGTTAGTCATNGGGAATATTT-
AAGCAAAGACGCGATGGATGATCGAGACTTCCATGGCTGCCCCAACGGATCTACTTGTA
GAGGAGCGGCAGGCGGCAGTGTGGCTCCGTCTCGCTGGGATTGTCGGAGTACAGGGAG
ATGGTCCTGGCACTGAGATCGAAGATGCCCACGGCGATGGTCTTGACCACCTCGTCGCA
GCTGTTGTTCTCCCGCCAGACGCAGTAGACTCCGCCGGACACATCGCCCAGCATGTGGC
GCACATCCTGCTCGCTCATCGGCGGATTGTAGGCTCCGAAGGTTTTCATCCTGGAGATGC
TCGAGCTGATCATCAGATCGTTGGCCTGCTCCTGACGGATGCGATCGAATCTGCAATATA
CATGCATACACGTGTATACTGCATATGAAATACTTTCATGCAACACTTTTCATGCACTCAC
TGGTTAACATGGTAGTTGTGCTCGCCCAGCGGCACCTCCTTGATGTTCAAATAGGACTCA
TTCTTGCGCTCCGGCGACGGAGCCATCTCCACATTGTAGCACATCTGGCGGGGATCCCTG
TTGGGCATTGGCAAGCAGCTCGGATTAGTAAAACAAAATGATGGTGCACAGTTAAACGT
ACGCCAGGAAGGTAAAGTTGACGGAGCAGGCATCCGCTGCACCCACTCCCGCATCCTT
GAGCACCCGGAACGCATCGTCCACGTTGCTGGTGGCCAACAGAGCTCGGGTGATGAAG
TGTCGCGCTGGTGG-ATAAATGCATTCAATG-AAAACCGTCCCTTTTT--AAGGGATTCTTT-
-
GGGCACGTACGGGTTCTGCCGCTGCGCAAGAGCTCCGCACTGATGGTGTTAATGCTGAA
GACCAGTCCGTGGTGATTCTGGCTCATGGTGTATCCGGGCAGGTGTCCGGCGTAGC
>L_EGAB152B
ATAGATCATAGATGGATTTCATGTTAGTCATTGGGAATATTT-
AAGCAAAGACGCGATGGATGATCGAGACTTCCATGGCTGCCCCAACGGATCTACTTGTA
GAGGAGCGGCAGGCGGCAGTGTGGCTCCGTCTCGCTGGGATTGTCGGAGTACAGAGAG
ATGGTCCTGGCACTGAGATCGAAGATGCCCACGGCGATGGTCTTGACCACCTCGTCGCA
GCTGTTGTTCTCCCGCCAGACGCAGTAGACTCCGCCGGACACATCGCCCAGCATGTGGC
GCACATCCTGCTCGCTCATCGGCGGATTGTAGGCTCCGAAGGTTTTCATCCTGGAGATGC
TCGAGCTGATCATCAGATCGTTGGCCTGCTCCTGACGGATGCGATCGAATCTGCAATATA
CATGCATACACGTGTATACTGCATATGAAATACTTTCATGCAACACTTTTCATGCACTCAC
TGGTTAACATGGTAGTTGTGCTCGCCCAGCGGCACCTCCTTGATGTTCAAATAGGACTCA
TTCTTGCGCTCCGGCGACGGAGCCATCTCCACATTGTAGCACATCTGGCGGGGATCCCTG
TTGGGCATTGGCAAGCAGCTCGGATTAGTAAAACAAAATGATGGTGCACAGATAAACGT
ACGCCAGGAAGGTAAAGTTGACGGAGCAGGCATCCGCTGCACCCACTCCCGCATCCTT
GAGCACCCGGAACGCATCGTCCACGTTGCTGGTGGCCAACAGAGCTCGGGTGATGAAG
TGTCGCGCTGGTGG-
ATAAATGCATTCAATGAAAAACCGTCCCCTTTTTAAAGGGATTCTTT--
GGGCACGTACGGGTTCTGCCGCTGCGCAAGAGCTCCGCACTGTGGGTGTTAATGCTGAA
GACCAGTCCGTGGTGATTCTGGCTCATGGTGTATCCGGGCAGGTGTCCGGCGTAGC
>D_EGAB153A
ATAGATCATAGATGGATTTCATGTTAGTCATNGGGAATATTT-

AAGCAAAGACGCGATGGATGATCGAGACTTCCATGGCTGCCCCAACGGATCTACTTGTA
GAGGAGCGGCAGGCGGCAGTGTGGCTCCGTCTCGCTGGGATTGTCGGAGTACAGGGAG
ATGGTCCTGGCACTGAGATCGAAGATGCCCACGGCGATGGTCTTGACCACCTCGTCGCA
GCTGTTGTTCTCCCGCCAGACGCAGTAGACTCCGCCGGACACATCGCCCAGCATGTGGC
GCACATCCTGCTCGCTCATCGGCGGATTGTAGGCTCCGAAGGTTTTCATCCTGGAGATGC
TCGAGCTGATCATCAGATCGTTGGCCTGCTCCTGACGGATGCGATCGAATCTGCAATATA
CATGCATACACGTGTATACTGCATATGAAATACTTTCATGCAACACTTTTCATGCACTCAC
TGGTTAACATGGTAGTTGTGCTCGCCCAGCGGCACCTCCTTGATGTTCAAATAGGACTCA
TTCTTGCGCTCCGGCGACGGAGCCATCTCCACATTGTAGCACATCTGGCGGGGATCCCTG
TTGGGCATTGGCAAGCAGCTCGGATTAGTAAAACAAAATGATGGTGCACAGATAAACGT
ACGCCAGGAAGGTAAAGTTGACGGAGCAGGCATCCGCTGCACCCACTCCCGCATCCTT
GAGCACCCGGAACGCATCGTCCACGTTGCTGGTGGCCAACAGAGCTCGGGTGATGAAG
TGTCGCGCTGGTGG-ATAAATGCATTCAATG-AAAACCGTCCCTTTTT--AAGGGATTCTTT-
-
GGGCACGTACGGGTTCTGCCGCTGCGCAAGAGCTCCGCACTGATGGTGTTAATGCTGAA
GACCAGTCCGTGGTGATTCTGGCTCATGGTGTATCCGGGCAGGTGTCCGGCGTAGC
>D_EGAB153B
ATAGATCATAGATGGATTTCATGTTAGTCATTGGGAATATTT-
AAGCAAAGACGCGATGGATGATCGAGACTTCCATGGCTGCCCCAACGGATCTACTTGTA
GAGGAGCGGCAGGCGGCAGTGTGGCTCCGTCTCGCTGGGATTGTCGGAGTACAGGGAG
ATGGTCCTGGCACTGAGATCGAAGATGCCCACGGCGATGGTCTTGACCACCTCGTCGCA
GCTGTTGTTCTCCCGCCAGACGCAGTAGACTCCGCCGGACACATCGCCCAGCATGTGGC
GCACATCCTGCTCGCTCATCGGCGGATTGTAGGCTCCGAAGGTTTTCATCCTGGAGATGC
TCGAGCTGATCATCAGATCGTTGGCCTGCTCCTGACGGATGCGATCGAATCTGCAATATA
CATGCATACACGTGTATACTGCATATGAAATACTTTCATGCAACACTTTTCATGCACTCAC
TGGTTAACATGGTAGTTGTGCTCGCCCAGCGGCACCTCCTTGATGTTCAAATAGGACTCA
TTCTTGCGCTCCGGCGACGGAGCCATCTCCACATTGTAGCACATCTGGCGGGGATCCCTG
TTGGGCATTGGCAAGCAGCTCGGATTAGTAAAACAAAATGATGGTGCACAGATAAACGT
ACGCCAGGAAGGTAAAGTTGACGGAGCAGGCATCCGCTGCACCCACTCCCGCATCCTT
GAGCACCCGGAACGCATCGTCCACGTTGCTGGTGGCCAACAGAGCTCGGGTGATGAAG
TGTCGCGCTGGTGG-ATAAATGCATTCAATGAAAAACCGTCCCTTTTT--
AAGGGATTCTTT-
GGGGCACGTACGGGTTCTGCCGCTGCGCAAGAGCTCCGCACTGATGGTGTTAATGCTGA
AAACCAGTCCGTGGTGATTCTGGCTCATGGTGTATCCGGGCAGGTGTCCGGCGTAGC
>D_EGAB154B
NNNNNNNNNNNNNNNNNNNNNNNNNNNNNNNNNNNNNNNNNNNNNNNNNNNNNNNNNN
NNNNNNNNNNNNNNNACTTCCATGGCTGCCCCAACGGATCTACTTGTAGAGGAGCGGCA
GGCGGCAGTGTGGCTCCGTCTCGCTGGGATTGTCGGAGTACAGAGAGATGGTCCTGGCA
CTGAGATCGAAGATGCCCACGGCGATGGTCTTGACCACCTCGTCGCAGCTGTTGTTCTC
CCGCCAGACGCAGTAGACTCCGCCGGACACATCGCCCAGCATGTGGCGCACATCCTGCT
CGCTCATCGGCGGATTGTAGGCTCCGAAGGTTTTCATCCTGGAGATGCTCGAGCTGATCA
TCAGATCGTTGGCCTGCTCCTGACGGATGCGATCGAATCTGCAATATACATGCATACACG
TGTATACTGCATATGAAATACTTTCATGCAACACTTTTCATGCACTCACTGGTTAACATGG
TAGTTGTGCTCGCCCAGCGGCACCTCCTTGATGTTCAAATAGGACTCATTCTTGCGCTCC
GGCGACGGAGCCATCTCCACATTGTAGCACATCTGGCGGGGATCCCTGTTGGGCATTGG
CAAGCAGCTCGGATTAGTAAAACAAAATGATGGTGCACAGATAAACGTACGCCAGGAA
GGTAAAGTTGACGGAGCAGGCATCCGCTGCACCCACTCCCGCATCCTTGAGCACCCGGA
ACGCATCGTCCACGTTGCTGGTGGCCAACAGAGCTCGGGTGATGAAGTGTCGCGCTGGT
GG-ATAAATGCATTCAATG-AAAACCGTCCCTTTTT--AAGGGATTCTTT-
GGGGCACGTACGGGTTCTGCCGCTGCGCAAGAGCTCCGCACTGATGGTGTTAATGCTGA

AGACCAGTCCGGGGTGATTCTGGCTCATGGTGTATCCGGGCAGGTGTCCGGCGTAGC

Supplementary Note 4 | Alignment of the t_MSE region in nine species of the melanogaster subgroup including the light and dark haplotypes that were used in the GFP reporter experiment. Sequences are aligned according to the D. melanogaster genome (reverse relative to D. erecta).

>melanogaster
GCGTTCCAACACCCCGTCTAATCTAAGATCTATTTATTGCACTTTTTTTTTCCTAGCTTCGT
AAGTCG-AT-AGT--
CAGTTTTCAGATTTAAGCGATAAATAAATAAATGTTTGTAAACACTTAAAGAAATGTTATT
CAATTTCTAGGAATCAAGTTGTTAGAAG-TAATAAACAAAAGGTTAACTTATTTGTTT----
ATTTATTTATTTGTTT-----------CAACTCAATCCTAGC-AGTTGGAAA-
GCACCAAGACCACTTGAAAA------
TTATAATGTAATAAAAACGCAAATTTGAATAGTTGAAGTAATAAAAAAAA-
GAGACTGTCTAATTAGTATGCATAT--
TATGATCTTGAATTCTCAAATTAGTTTATTTAAACACTAAAATCTAATATTCTAATGGTGCA
AGAGTA---
AAATGCACTCATTTTTGTTATTGACTTCAACTTTGGATACCAACCGGTTCACTTGGCAAT
GGGAATTTAAGTATACTACTTAGTGCAATTATCCTAAGTCTTGATTCTATCTAT--------
AAAAC-------GTATAATATATTT-----------------------------
ACATTCTGATTATTTATTATTATTTCAAATACGGAAATCGTGT-
TTGGTACATTCATACATGGACTACTGTTCTCAGTGTTTTTGAGGAAGTCAGTTAGGCTAAT
GGATTTCTCAGTTGTTTGTTGGCCCATTTAAAGGGA----
GATCCGCCGCAAGGATAAGGCCTGTCC--ATCCAT---
AAGGGTTTCACTTCCGAGCAGTCAACGATGAGCGGCCATCTGGGTCGGGTCCTGCAGTT
CCACCTGCGGCTCTACCAGGTGCTCGGC
>mauritiana
GTGTCCCAACACCCCGCCTAATCTAAGATCTATTTATTGCA--
TTTTTTTTCGTAGCTTCGTAAGTCG-AT-
AGTATCAGTTTTAAGATTTAAGCGATAAATAAAGAAATGCTTGTAAACACTCAAAGAAAT
GTTATTCGATTTCTAGGAATCAAGTTGTTA-----------------GCTAAACTTATTTGTTT------------
--ATGTTTATGT-----CTCAACACAATCCTAGC-AGTTGGAAA-
GCACCAAGACCACTTGAAAA------
TTATAAAGTAATAAAAACGCAAATCTGAATAGTTGAAGTATT-AAAAAAA-
GAGACTGTCTAATTAGTATACAAGT--
TATGATCTTGAATTCGCAAATTAGTTTATTTAAACACTAAAATCTAATATTCTAATGGTGCA
AGAGTA---AAATGCACTCATTTTTGTTATTGACTTCAACTTTGGATAAT-
ACCGGTTCACTTGGCACTGGGAACTTAAGTATACTACTTAATGCAATTATCCTAAGCCTT
GATTCTATCTAT--------AAAAC-------GTATAACATATTT----------------
ACAAGCATTCTTTAGTTTCTAATTGTTTATTATTGTTTTAAATACGGAAATCGAGT------------
-----
TGGATTACTGTTCTCAGTGTTTTTGAGGAAGTCACTTAGGCTAATGGATTTGCCAGTTGT
TTGGTGGCCCATTTAAAGGAA----GATCTGCCGCAGGGATAAGGCCTGTCC--ATCCA---
AAAGGGTTTCGCTTCCGAGCAGTCAACGATGAGCGGCCGTCTGGGTCGGGTCCTGCAG
TTCCACCTGCGGCTCTACCAGGTGCTCGGC
>simulans
GTGTCCCAACACCCCGTCTAATCTAAGATCTATTTATTGCA--
TTTTTTTTCGTAGCTTCGTAAGTCG-AT-

AGTATCAGTTTTAAGATTTAAGCGATAAATAAAGAAATGTTTGTAAACACTCAAAGAAAT
GTTATTCGATTTCTAAGAATCAAGTTGTTA-----------------GCTAAACTTATTTGTTT------------
--ATGTCTATGT-----CTCAACACAATCCTAGC-AGTTGGAAA-
GCACCAAGACCACTTGAAAA------TTATAAGGTAAT-
AAAACGCAAATCTGAATAGTTGAAGTATTAAAAAAAA-
GAGACTGTCTAATTAGTATACAAAT--
TATGATCTTGAATTCGCAAATTAGTTTATTTAAACACTAAAATCTAATATTCTAATGGTGCA
AGAGTA---AAATGCACTCATTTTTGTTATTGACTTCAACTTTGGATAAT-
ACCGGTTCACTTGGCAATGGGAACTTAAGTATACTACTTAATGCAATTATCCTAAGCCTTG
ATTATATCTAT--------AAAAC-------GTATAACATATTT----------------
ACAAGCATTCTTTAGTTTCTAATTGTTTATTATTATTTTAAATACGGAAATCGAGT------------
-----
TGGATTGCTGTTCTCAGTGTTTTTGAGGAAGTCACTTAGGCTAATGGATTTGCCAGTTGT
TTGGTGGCCCATTTAAAGGTA----GATCTGCCGCAGGGATAAGACCTGTCC--ATCCA---
AAAGGGTTTCGCTTCCGAGCAGTCAACGATGAGCGGCCGTCTGGGTCGGGTCCTGCAG
TTCCACCTGCGGCTCTACCAGGTGCTCGGC
>sechellia
GTGTCCCAACACCCCGTCTAATCTAAGATCTATTTATTAC---
TTTTTTTTCGTAGCTTCGTAAGTCG-AT-
AGTATCAGTTTTAAGATTTAAGCGATAAATAAAGAAATGTTTGTAAACACTCAAAGAAAT
GTT---AAACTTATTTGCTT--------------ATGTTTATGT-----
CTCAACGCAATCCTATCCAGTTGGAAA-GCACCAAGACCACTTGAAAA------
TTATAAAGTAATAAAAACGCAACTCTGAATAGTTGAAGTATTAAAAAAAAAGAGACTGT
CTAATTAGTATACAAAT---
TATGATCTTGAATTCGCAAATTAGTTTATTTAAACACTAAAATCTAATATTCTAATGGTGCA
AGAGTA---AAATGCACTCATTTTTGTTATTGACTTCAACTTTGGATAAT-
ACCGGTTTACTTGGCAATGGGAACTTA----
TACTACTTAATGCAATTATCCTAAGCCTTGATTATATTTAT--------AAAAC-------
GTATAACATATTT-----------------ACAAGCA--------
TTTCTAATTGTTTATTATTATTTTAAATACGGAAATCGAGT-----------------
TGGATTACTGTTCTCAGTGTTTTTGAGGAAGTCACTTAGGCTAATGGATTTGCCAGTTGT
TTGGTGGCCCATTTAAAGGAA----GATCTGCCGCAGGGATAAGGCCTGTCC--ATCCA---
AAAGGGTTTCGCTTTCGAGCAGTCAACGATGAGCGGCCGTCTGGGTCGGGTCCTGCAG
TTCCACCTGCGGCTCTACCAGGTGCTCGGC
>erecta "light"
GTGTACCAGCCCCCCGCCTAATTTAAGATCTATTTATTGCA--
TTTTTTTTCGTAGCTTCGTAAGTTG-AT-
AGTATCAGTTTTAAGATTTAAGCGATAAATAAAGAAATGTTTGTAAGCACTCAGCGAAAT
GTTTTTACAC----------------TTTTATAGGTAATTGGCAAAAGGCAAACTCCTTTGAAA----
TATC-----AATGTG-------TCTTTCAACACAATTCTAGC-
AGTTCGAAACGCACAAAACCCACTTGGAAA------TTATCTAGTGATAAAATCGC------
AAATAGTTTGAGTAAT-AAAAAAA-GAGACTGTCCACTTAATATACAAAT----------------------
---------------------GATGATCTTGAATTCGCAAATTAGTTTATTTAACTAAT-
AAATTTAATATTCTAATGGTCCAAGAACATTTTAATGCACTTATTTTAGTTATTAGTTGCAA
CTTTAGAT----ACCGGTTCATTTAGC-
ATTGGAATTTAAGTACACTACTAAATGCAATTATCCTAAGCCTTGATTCTGCCTATAAAAT
TATAAAACATATAATGTA-----------------------
GACACAGACAATCTTTACTTTCTGGTGGTTTGTGTTCATTT-AAATACGGAAATCAAGG----
-----
AAGCTACATGGAGTACTGTTCTCAGTGTTTTTCACAAATTCACTTAGGCTAATGGATTTG

TCAGTTGTTTGGTGGCCCATTTGAAGGCAGAT-
GATCGGCCGCTGGGATAAGGCCTGTCCCTGTCCATCTAAAGGGTTTCGCCTTCGCGTAGT
CAACAATGAGCGGCCGTCTGGGTCGGGTGCTGCAGTTCCACTTGCGGCTCTACCAGGTG
CTCGGC
>erecta "dark"
GTGTACCAGCCCCCCCCCTAATCTAAGATCTATTTATTGCA--
TTTTTTTTCGTAGCTTCGTAAGTTG-AT-
AGTATCAGTTTTAAGATTTAAGCGATAAATAAAGAAATGTTTGTAAGCACTCAACGAAAT
GTTTTTAGATCTTCCGGAAAACTACATTTTATTGGTAATTGGCAAAAGGCAAACTCCTTT
GAAATGTGTATCAATTTAATGTTCATGTT-TCTTTCAACACAATTCTAGT-
AGTTCGAAATGCACAAAAAGCACTAGGAAA------
TTATCTAGTAATAAAAACGAAAATTTGAATAGTTGGAGTAAT-AAAAAAA-
GAGACTGTCCAATTAATATAC-AAT--
TATGATCTTGAATTCGCAAAATAGTTTAATTAAACACCAAAAATTAGTATTCTAATGTTCT
TGGACTATTTTAATGCACTTATTTTT-TTATTGATCGCAACTGTAGAT----
ACCGGTTCATTTAGC-
ATTGGAATTTAAGTACACTACTAAATGCAATTATCCTAAGCCTTGATTCTGCCTATAAAAT
TATAAAACATGAAATGTA------------------------
GATACAGGCAATATTTACATTCTGGTCGTTTATATTCATTT-ACATACGGAAATCAAGG------
----
ATTACTGTTCTCAGTGTTTTTCACAAATTCACTTAGGCTAATGGATTTGTCAGTTGTTTGG
TGGCCCATTTGAAGGGAGAT-GATC-------
GGGATAAGGCCTGTCCCTGTCCATGTAAAGGGTTTCGCCTTCGCGTAGTCAACAATGAG
CGGCCGTCTGGGTCGGGTGCTGCAGTTCCACTTGCGGCTCTACCAGGTGCTCGGC
>orena
GTGTACCAGCACCCCGCCTAATCTAAGATCTATTTATTGCA--TTTTTTTTCGTAGC-
TCGTAAGTGG-AT-
AGCATCAGTTTTAAGAGTTAAGCGATAAATAAAGAAAAGTTTGTAACCACTCAACAAAA
TGTTTTCACATTTTCCGGAAAACTACATTTTATAGGTAATTGGCAAGAGACAAACTCCTT
TGAAA----TATCAATTTAATGATCATGTT-TCTTTCAACGCAATTCTAGC-
AGTTCGAAATGCAAAAAAGGCACTTGGAAA------
TTATCTAGTAATAAAAACGCAAATTTCAATAGTTGGAGTAAT-AAAAAAA-
GAGACTGGTTAATTAATATACTAATATATATATACTATACACTATACTATATATTGTATACTAA
TATACTATGATCTTGAATTCGCAAATTAGTTTAATTAAACACCAAAAATTAGTATTCTAATG
TGCTTGGACCATTTTAATGCACTTATTTTT-TTATTGATCGCAACTTTAGGA----
TCCGGTCCATTTAGC-
ATTGGAATTTAAGTGCACTACTAAATGCAATTATCCTAAGCCTTGATTCCACCTATAAAAT
TATAAAACATACAGTGTA------------------------
GATACAGGCAATCTTTAGTTTCTGGTCGCTTATATTCATTT-AAGTACGGAAATCAAGG-----
----
AAGCTACATGGATTACTGTTCTCAGTGTTTTTGACAAATTCACTTAGGCTAATGGATTCGT
CAGTTGTTTGGTGGCCCATTT-AAGGG-GA--GATCGGCCACTGGGATAAGG------
CGTGTCCATCTGAAGGGTTTCGCTTTCGCGTAGTCAACAATGAGCGGCCGTCTGGGTCG
GGTGCTGCAGTTCCACTTGCGGCTATACCAGGTGCTCGGA
>teissieri
GTGTACCAAGACCGCGCCTAATCTAAGATCTATTTATTGCA--
TTTTTTTTCGTAGCTTCGTAAGTTGTATTAGTATCAGTTTTAAGATTTAAGCGATAAATAAA
GAAATGTTTACAGACACTCAAAGAAATGTTGTTACACTTTCCGAAAAC-------------
TAATTTT------TAAAAGTAATTTGAAA----TATCAATTTAATGTTCATGTTCTTTTT-------------
G-AGTTTGAAGTGCACAAAACCCACTTGGAAAAACCACTTATCGAGTATTAA---------

AAAAAATAGTTGCAGTAAT--AAAAAA-GAGACTGTTCAATTAATATACAAAT---
TATGATCTTGAATTCGCAAATTAGTTGATTTAAACACTAAAAATAAATATTCTAATGGTCC
AAGAGTG---AAATGCACCTATTTTTGTTATTGATTTCAACTTTGGAT----
ACCGGTTCACTCAGCCATGGCAATTTAATACTACTACTAAATGCAATTATCCTAAGCCTTC
ATTCTACTTATAAAACTATAAACCTATAAAACTATAAAACTATATAGCGTAGACTGTGGGC
ACAAGCAAT-TTTAGTTTCTGGTTATTTATATTCTCTT-CAATACGGAAATCGAGG---------
CAGGTACATGGACTACTGTTCTCAGTGTTTTTGAAAAAGCCACTTAGGCTAATGGATTTC
TCAGTTGTTCGGTGGCCCATTTAAAGGGAGATCGGTCG----CGGGGATAAGG------
CATGTCCATCTAAAAGGTTTCGCGTCCGAGTAGTCAACGATGAGTGGCCGTCTGGGTCG
GGTTCTGCAGTTCCACCTGCGGCTCTACCAGGTGCTCGGC
>yakuba
GTGTACCAACAGCCCGCCTAATCTAAGATCTATTTATTGCA--
TTTTTTTTCGTAGCTTCGTAAGTTGTATTAGTAACAGTTTTAAGATTTAAGCGATAAATAA
AGAAATGTTAATAGACACTGAAAGAAATGTTATAACACTTTTCGAAAAA-------------
TAATTGACACACGTCAAACTCCTTTGAAA----TATCAATGAAATGTTCATGT--TTTCT--------------C-AGTTTATAGTGCACAAACACCACTTGGAAAAACCACTTATCGAGTAATAA---------
AAAAAATAGTTGCGGTAAT--AAAAAA-GAGACTGTCCAATGAATATACAAAT---
TATGATCTTGAATTCGCAAATTGGTTGATTTAAGCACTAAAAATAAATATTCTAATGGTCC
AGGAGTG---AAATGCACCTATTTTTGTTATTGATTTCAACTTTAGAT----
ACCGGTTCACTCAGCCATGGGAATTTAAGTATGCTACTAAATGCAATTATCCTAAGCCTTC
ATTCTACTTATTAAACTATAAAACTATAAAACGT----------------
AGACTGTAGGTACAATCAATCTGTAGTTTCTAGTTATTTATATACTTAT-
AAATACGGAAATCGAGA---------
AAGGTACATGGATTACGGTTCTCTGTGTATTTGAGAAAACCACTTAGGCTAATGGATTCG
TC-----------GGTCCTTTTAAAGGTAGATTGACC-----CAGGGATAAGT------
ACTAGCCATCTAATGGGTCTCGCTTTCGCGTAGTCAACGATGAGTGGCCGTCTGGGTCGG
GTTCTGCAGTTCCACCTGCGGCTCTACCAGGTGCTCGGC
>santomea
GTGTACCAACAGCCCGCCTAATCTAAGATCTATTTATTGCA--
TTTTTTTTCGTAGCTTCGTAAGTTGTATTAGTATCAGTTTTAAGATTTAAGCGATAAATAAA
GAAATGTTAATAGACACTGAAAGAAATGTTATTACACTTTTCGGAAAA-------------
TAGTTGACACAAATCAAACTCCTTTGGAA----TATCGATTAAATGTTCATGT--TTTCT--------------C-AGTTTATAGTGCACAAACACCACTTGGGAAAACCACTTATCGAGTAATAA------------ATATAGTTGCGGAAAT--AAAAAA-GAGACTGTCCAATAAATATACAAAT---
TATGATCTTGAATTCGAAAATTGGTTGATTTAAGCACTAAAAATAAATATTCTAATGGTCC
AAGAGTG---AAATGCACCTATTTTTGTTATTGATTTCAACCTTAGAT----
ACCGGTTCACTCAGCCATGGGAATTTAAGTATGCTACTAAATGCAATTATCCTAAGCCTTC
ATTCTACTTATAAAACCATTAAACTATAAAACGT----------------
AGACTGTAGGTACAATCAATCTGTAGTTTCTGGTTATTTATATACTTAT-
AAATACGGAAATCGAGA---------
AAGGTACATGGATCACTGTTCTCGGTGTATTTGAGAAAACCACTTAGGCTAATGGATTCG
TC-----------GGTCCTTTAAAAGGGAGATAGACC-----CAGGGATAAGT------
ACTAGCCATCTAAAGGGTCTAGCTTTCGCGTAGTCAACGATGAGTGGCCGTCTGGGTCG
GGTTCTGCAGTTCCACCTGCGGCTCTACCAGGTGCTCGGC
